# Supplementary material for: Synthesis of Oxidized 3β,3′β-Disteryl Ethers and Search after High-Temperature Treatment of Sterol-Rich Samples
Source: Int J Mol Sci. 2021 Sep 27;22(19):10421. doi: 10.3390/ijms221910421 (PMC8508662; doi:10.3390/ijms221910421)
Supplement: Supplementary file 1 [file ijms-22-10421-s001.zip › ijms-1391754-supplementary.pdf]

Supplementary data
Synthesis of oxidized 3β,3'β-disteryl ethers and search after high-temperature treatment of sterol-rich samples

Authors: Adam Zmysłowski[1], Jerzy Sitkowski[1], Katarzyna Bus[1], Katarzyna Michalska[1], Arkadiusz Szterk[2]
[1]National Medicines Institute, 30/34 Chełmska, 00-725 Warsaw, Poland.
[2]Center for Translationale Medicine Warsaw University for Life Sciences, Nowoursynowska 100, 02-797 Warsaw, Poland
Transfer of Science Sp. z o.o. Janiszowska 14, 02-264 Warszawa

Correspondence:
Adam Zmysłowski MSc, National Medicines Institute, Department of Spectrometric Methods, 30/34 Chełmska, 00-725 Warsaw, Poland
Email: a.zmyslowski@nil.gov.pl

1.1. Chemical Synthesis

3β,3'β-Dicholesteryl ether (**1**). The synthesis was performed as already described [4]. A solution that contained cholesterol (3.48 g, 9.0 mmol) and MK10 (2.7 g) in CH_2Cl_2 (250 mL) was stirred at refluxing temperature until the substrate disappeared (TLC, solvent: n-Hex:EtOAc 3:1 *v/v*). After cooling, the catalyst was removed via filtration, and the solvent was evaporated under reduced pressure. 3β,3'β-Dicholesteryl ether was purified by crystallization (n-Hex:EtOAc 10:1 *v/v*) and using DCVC with n-Hex:Et_2O (95:5 *v/v*) as a mobile phase to give the title compound (2.421 g, yield: 71%) as a white solid. The ^1H and ^{13}C NMR spectral data are listed in Table S8 and match the expected values [4].

3β,3'β-Disitosteryl ether (**2**). The synthesis was performed as already described [4]. A solution that contained β-sitosterol (3.71 g; 9.0 mmol) and MK10 (2.7 g) in CH_2Cl_2 (250 mL) was stirred at refluxing temperature until the substrate disappeared (TLC, solvent: n-Hex:EtOAc 3:1 *v/v*). After cooling, the catalyst was removed via filtration, and the solvent was evaporated under reduced pressure. 3β,3'β-Disitosteryl ether was purified by crystallization (n-Hex:EtOAc 10:1 *v/v*) and using DCVC with n-Hex:Et_2O (95:5 *v/v*) as a mobile phase to give the title compound (2.531 g, yield: 69%) as a white solid. The ^1H and ^{13}C NMR spectral data are listed in Table S9 and match the expected values [4].

3β,3'β-Distigmasteryl ether (**3**). The synthesis was performed as already described [4]. A solution that contained stigmasterol (3.73 g; 9.0 mmol) and MK10 (2.7 g) in CH_2Cl_2 (250 mL) was stirred at refluxing temperature until the substrate disappeared (TLC, solvent: n-Hex:EtOAc 3:1 *v/v*). After cooling, the catalyst was removed via filtration, and the solvent was evaporated under reduced pressure. 3β,3'β-Distigmasteryl ether was purified by crystallization (n-Hex:EtOAc 10:1 *v/v*) and using DCVC with n-Hex:Et_2O (95:5 *v/v*) as a mobile phase to give the title compound (2.614 g, yield: 72%) as a white solid. The ^1H and ^{13}C NMR spectral data are listed in Table S10 and match the expected values [4].

Cholest-5-en-7-one-3β-yl-cholesterol ether (7-ketocholesteryl-cholesterol ether) (**4**).
Method I. A solution that contained cholesterol (772 mg, 2.0 mmol), 7-ketocholesterol (**22**, 401 mg, 1.0 mmol), and MK10 (0.6 g) in CH_2Cl_2 (120 mL) was stirred at refluxing temperature until the cholesterol disappeared (TLC, solvent: n-Hex:EtOAc 3:1 *v/v*). After cooling, the catalyst was removed

via filtration, and the solvent was evaporated under reduced pressure. The white crystals were purified using DCVC with n-Hex:EtOAc (15:1 *v/v*) as a mobile phase to give the title compound (251 mg, yield: 67%) as a white solid.

Method II. Chromium trioxide (CrO_3) (0.50 g, 5.0 mmol) was suspended in 50 mL of dry CH_2Cl_2 and stirred for 30 min at −20°C. 3,5-Dimethylpyrazole (481 mg, 5.0 mmol) was added in one portion, and the reaction mixture was stirred for 30 min at −20°C. 3β,3β'-Dicholesteryl ether (**1**, 755 mg, 1 mmol) was added, and the mixture was stirred at −20°C for 1 h and then allowed to warm to room temperature. The solvent was evaporated in a rotavapor and redissolved in n-Hex:EtOAc (3:1 *v/v*), and the brown suspension was filtered through a short pad of Celite and silica gel. The solvent was evaporated. The yellow crystals were purified by DCVC using n-Hex:EtOAC (15:1 *v/v*) as a mobile phase to give the title compound (431 mg, yield: 56%) as a white solid.

The ^{1}H and ^{13}C NMR spectral data are listed in Table S8 and match the expected values [4].

Sitost-5-en-7-one-3β-yl-sitosterol ether (7-ketositosteryl-sitosterol ether) (**5**).

Method I. A solution that contained β-sitosterol (824 mg, 2.0 mmol), 7-ketositosterol (**23**, 428 mg, 1.0 mmol), and MK10 (0.6 g) in CH_2Cl_2 (120 mL) was stirred at refluxing temperature until β-sitosterol disappeared (TLC, solvent: n-Hex:EtOAc 3:1 *v/v*). After cooling, the catalyst was removed via filtration, and the solvent was evaporated under reduced pressure. The white crystals were purified using DCVC with n-Hex:EtOAc (15:1 *v/v*) as a mobile phase to give the title compound (264 mg, yield: 65%) as a white solid.

Method II. Chromium trioxide (CrO_3) (0.5 g, 5.0 mmol) was suspended in 50 mL of dry CH_2Cl_2 and stirred for 30 min at −20°C. 3,5-Dimethylpyrazole (481 mg, 5.0 mmol) was added in one portion, and the reaction mixture was stirred for 30 min at −20°C. 3β,3β'-Disitosteryl ether (**2**, 755 mg, 1 mmol) was added, and the mixture was stirred at −20°C for 1 h and then allowed to warm to room temperature. The solvent was evaporated in a rotavapor and redissolved in n-Hex:EtOAc (3:1 *v/v*), and the brown suspension was filtered through a short pad of Celite and silica gel. The solvent was evaporated. The yellow crystals were purified by DCVC using n-Hex:EtOAC (15:1 *v/v*) as a mobile phase to give the title compound (448 mg, yield: 54%) as a white solid.

The ^{1}H and ^{13}C NMR spectral data are listed in Table S9 and match the expected values [4].

Stigmast-5-en-7-one-3β-yl-stigmasterol ether (7-ketostigmasteryl-stigmasterol ether) (**6**).

Method I. A solution that contained stigmasterol (828 mg, 2.0 mmol), 7-ketostigmasterol (**24**, 426 mg, 1.0 mmol), and MK10 (0.6 g) in CH_2Cl_2 (120 mL) was stirred at refluxing temperature until the stigmasterol disappeared (TLC, solvent: n-Hex:EtOAc 3:1 *v/v*). After cooling, the catalyst was removed via filtration, and the solvent was evaporated under reduced pressure. The white crystals were purified using DCVC with n-Hex:EtOAc (15:1 *v/v*) as a mobile phase to give the title compound (461 mg, yield: 56%) as a white solid.

Method II. Chromium trioxide (CrO_3) (0.5 g, 5.0 mmol) was suspended in 50 mL of dry CH_2Cl_2 and stirred for 30 min at −20°C. 3,5-Dimethylpyrazole (481 mg, 5.0 mmol) was added in one portion, and the reaction mixture was stirred for 30 min at −20°C. 3β,3β'-Distigmasteryl ether (**3**, 755 mg, 1 mmol) was added, and the mixture was stirred at −20°C for 1 h and then allowed to warm to room temperature. The solvent was evaporated in a rotavapor and redissolved in n-Hex:EtOAc (3:1 *v/v*), and the brown suspension was filtered through a short pad of Celite and silica gel. The solvent was evaporated. The yellow crystals were purified by DCVC using n-Hex:EtOAC (15:1 *v/v*) as a mobile phase to give the title compound (461 mg, yield: 56%) as a white solid.

The ^{1}H and ^{13}C NMR spectral data are listed in Table S10 and match the expected values [4].

3β,3’β-Di(cholest-5-en-7-one-3β-yl) ether (di(7-ketocholesteryl) ether) (**7**). Chromium trioxide (CrO_3) (1.5 g, 15.0 mmol) was suspended in dry CH_2Cl_2 and stirred for 30 min at −20°C. 3,5-Dimethylpyrazole (1.442 g, 15.0 mmol) was added in one portion, and the reaction mixture was stirred for 30 min at −20°C. 3β,3β’-Dicholesteryl ether (**1**, 755 mg, 1 mmol) was added, and the mixture was stirred at −20°C for 1 h and then allowed to warm to room temperature. The solvent was evaporated in a rotavapor and redissolved in n-Hex:EtOAc (3:1 *v/v*), and the brown suspension was filtered through a short pad of Celite and silica gel. The solvent was evaporated. The yellow crystals were purified by DCVC using n-Hex:EtOAC (15:1 *v/v*) as a mobile phase to give the compound (621 mg, yield: 79%). The ^1H and ^{13}C NMR spectral data are listed in Table S11.

3β,3’β-Di(sitost-5-en-7-one-3β-yl) ether (di(7-ketositosteryl) ether) (**8**). Chromium trioxide (CrO_3) (1,5 g, 15.0 mmol) was suspended in dry CH_2Cl_2 and stirred for 30 min at −20°C. 3,5-Dimethylpyrazole (1.44 g, 15.0 mmol) was added in one portion, and the reaction mixture was stirred for 30 min at −20°C. 3β,3β’-Disitosteryl ether (**2**, 806 mg, 1 mmol) was added, and the mixture was stirred at −20°C for 1 h and then allowed to warm to room temperature overnight. The solvent was evaporated in a rotavapor and redissolved in n-Hex:EtOAc (3:1 *v/v*), and the brown suspension was filtered through a short pad of Celite and silica gel. The solvent was evaporated. The yellow crystals were purified by DCVC using n-Hex:EtOAC (15:1 *v/v*) as a mobile phase to give the compound (608 mg, yield: 73%). The ^1H and ^{13}C NMR spectral data are listed in Table S11.

3β,3’β-Di(stigmast-5-en-7-one-3β-yl) ether (di(7-ketostigmasteryl) ether) (**9**). Chromium trioxide (CrO_3) (1,5 g, 15.0 mmol) was suspended in dry CH_2Cl_2 and stirred for 30 min at −20°C. 3,5-Dimethylpyrazole (1.44 g, 15.0 mmol) was added in one portion, and the reaction mixture was stirred for 30 min at −20°C. 3β,3β’-Distigmasteryl ether (**3**, 805 mg, 1 mmol) was added, and the mixture was stirred at −20°C for 1 h and then allowed to warm to room temperature overnight. The solvent was evaporated in a rotavapor and redissolved in n-Hex:EtOAc (3:1 *v/v*), and the brown suspension was filtered through a short pad of Celite and silica gel. The solvent was evaporated. The yellow crystals were purified by DCVC using n-Hex:EtOAC (6:1 *v/v*) as a mobile phase to give the title compound (647 mg, yield: 78%) as a white solid. The ^1H and ^{13}C NMR spectral data are listed in Table S11.

Cholest-5-en-3β,7α-diol-cholesterol ether (7α-hydroxycholesteryl-cholesterol ether) (**10**). A solution of L-Selectride (1 M in THF, 0.25 mL, 0.25 mmol) was added dropwise to a solution of 7-ketocholesteryl-cholesterol ether (**4**, 154 mg, 0.20 mmol) in THF (7.5 mL) at −78°C. The resulting mixture was stirred at −78°C for 1.5 h. Water (0.25 mL), 6 M NaOH (0.25 mL), and 30% H_2O_2 (0.25 mL) were added successively to the reaction mixture to give a clean and transparent mixture. The dry ice was removed, and the reaction mixture was allowed to reach room temperature. Water (5 mL) was added, and the mixture was extracted with CH_2Cl_2 (2 × 10 mL) and saturated NaCl solution and then dried over anhydrous $MgSO_4$. The resulting precipitate was filtered, washed with water and dried under reduced pressure. The crude product was purified by DCVC using n-Hex:EtOAc (15:1 *v/v* to 10:1 *v/v*) as a mobile phase to give the title compound (97 mg, yield: 63%) as a white solid. The ^1H and ^{13}C NMR spectral data are listed in Table S12.

Sitost-5-en-3β,7α-diol-sitosterol ether (7α-hydroxysitosteryl-sitosterol ether) (**11**). A solution of L-Selectride (1 M in THF, 0.25 mL, 0.25 mmol) was added dropwise to a solution of 7-ketositosteryl-sitosterol ether (**5**, 154 mg, 0.20 mmol) in THF (7.5 mL) at −78°C. The resulting mixture was stirred at −78°C for 1.5 h. Water (0.25 mL), 6 M NaOH (0.25 mL), and 30% H_2O_2 (0.25 mL) were added successively to the reaction mixture to give a clean and transparent mixture. The dry ice was removed, and the reaction mixture was allowed to reach room temperature. Water (5 mL) was added, and the

mixture was extracted with CH_2Cl_2 (2 × 10 mL) and saturated NaCl solution and then dried over anhydrous $MgSO_4$. The resulting precipitate was filtered, washed with water and dried under reduced pressure. The crude product was purified by DCVC using n-Hex:EtOAc (15:1 *v/v* to 10:1 *v/v*) as a mobile phase to give the title compound (105 mg, yield: 64%) as a white solid. The ^{1}H and ^{13}C NMR spectral data are listed in Table S12.

Stigmast-5-en-3β,7α-diol-stigmasterol ether (7α-hydroxystigmasteryl-stigmasterol ether) (**12**). A solution of L-Selectride (1 M in THF, 0.25 mL, 0.25 mmol) was added dropwise to a solution of 7-ketostigmasteryl-stigmasterol ether (**6**, 165 mg, 0.20 mmol) in THF (7.5 mL) at −78°C. The resulting mixture was stirred at −78°C for 1.5 h. Water (0.25 mL), 6 M NaOH (0.25 mL), and 30% H_2O_2 (0.25 mL) were added successively to the reaction mixture to give a clean and transparent mixture. The dry ice was removed, and the reaction mixture was allowed to reach room temperature. Water (5 mL) was added, and the mixture was extracted with CH_2Cl_2 (2 × 10 mL) and saturated NaCl solution and then dried over anhydrous $MgSO_4$. The resulting precipitate was filtered, washed with water and dried under reduced pressure. The crude product was purified by DCVC using n-Hex:EtOAc (15:1 *v/v* to 10:1 *v/v*) as a mobile phase to give the title compound (101 mg, yield: 61%) as a white solid. The ^{1}H and ^{13}C NMR spectral data are listed in Table S12.

Cholest-5-en-3β,7β-diol-cholesterol ether (7β-hydroxycholesteryl-cholesterol ether) (**13**). $CeCl_3{}^x7H_2O$ (149 mg, 0.4 mmol) was dissolved in 10 mL of a mixture CH_2Cl_2/MeOH (1:1 *v*/*v*), and 7-ketocholesteryl-cholesterol ether (**4**, 154 mg, 0.2 mmol) in CH_2Cl_2 (2 mL) was added at room temperature. $NaBH_4$ (15 mg, 0.4 mmol) was added in small portions. The mixture was stirred at room temperature for 15 min, hydrolysed with a 10% aqueous HCl solution (2 mL), and then extracted successively with CH_2Cl_2 (3 × 25 mL). The organic layer was dried over $MgSO_4$, and the solvent was removed under reduced pressure. The crude product was purified by DCVC using n-Hex:EtOAc (10:1 *v/v*) as a mobile phase to give the title compound (114 mg, yield: 74%) as a white solid. The ^{1}H and ^{13}C NMR spectral data are listed in Table S13.

Sito-5-en-3β,7β-diol-sitosterol ether (7β-hydroxysitosteryl-sitosterol ether) (**14**). $CeCl_3{}^x7H_2O$ (149 mg, 0.4 mmol) was dissolved in 10 mL of a mixture CH_2Cl_2/MeOH (1:1 *v*/*v*), and 7-ketositosteryl-sitosterol ether (**5**, 164 mg, 0.2 mmol) in CH_2Cl_2 (2 mL) was added at room temperature. $NaBH_4$ (15 mg, 0.4 mmol) was added in small portions. The mixture was stirred at room temperature for 15 min, hydrolysed with a 10% aqueous HCl solution (2 mL), and then extracted successively with CH_2Cl_2 (3 × 25 mL). The organic layer was dried over $MgSO_4$, and the solvent was removed under reduced pressure. The crude product was purified by DCVC using n-Hex:EtOAc (10:1 *v/v*) as a mobile phase to give the title compound (124 mg, yield: 75%) as a white solid. The ^{1}H and ^{13}C NMR spectral data are listed in Table S13.

Stigma-5-en-3β,7β-diol-stigmasterol ether (7β-hydroxystigmasteryl-stigmasterol ether) (**15**). $CeCl_3{}^x7H_2O$ (149 mg, 0.4 mmol) was dissolved in 10 mL of a mixture CH_2Cl_2/MeOH (1:1 *v*/*v*), and 7-ketositmgatseryl-stigmasterol ether (**6**, 165 mg, 0.2 mmol) in CH_2Cl_2 (2 mL) was added at room temperature. $NaBH_4$ (15 mg, 0.4 mmol) was added in small portions. The mixture was stirred at room temperature for 15 min, hydrolysed with a 10% aqueous HCl solution (2 mL), and then extracted successively with CH_2Cl_2 (3 × 25 mL). The organic layer was dried over $MgSO_4$, and the solvent was removed under reduced pressure. The crude product was purified by DCVC using n-Hex:EtOAc (10:1 *v/v*) as a mobile phase to give the title compound (119 mg, yield: 72%) as a white solid. The ^{1}H and ^{13}C NMR spectral data are listed in Table S13.

(5,6α-epoxycholesteryl)-cholesterol ether (**16**). To a solution of 3β,3β'-dicholesteryl ether (**1**, 378 mg, 0.5 mmol) in 100 mL CH_2Cl_2 at 0°C, a solution of mCPBA (149 mg, 0.6 mmol) in 10 mL CH_2Cl_2 was added and mixed for 4 h at 0°C. The reaction progression was checked by TLC (10:1 *v/v* n-Hex:EtOAc). After 4 h at 0°C, the reaction was quenched with sat. aq K_2CO_3 (100 mL), and the aqueous layer was extracted with CH_2Cl_2. The combined organic layers were dried over anhydrous Na_2SO_4, and the solvent was evaporated under reduced pressure. The crude product was purified by DCVC using n-Hex:Et_2O (95:5 *v/v*) as a mobile phase to give the title compound (201 mg, yield: 52%) as a white solid. The ^1H and ^{13}C NMR spectral data are listed in Table S14.

(5,6α-epoxysitosteryl)-sitosterol ether (**17**). To a solution of 3β,3β'-disitosteryl ether (**2**, 402 mg, 0.5 mmol) in 100 mL CH_2Cl_2 at 0°C, a solution of mCPBA (149 mg, 0.6 mmol) in 10 mL CH_2Cl_2 was added and mixed for 4 h at 0°C. The reaction progression was checked by TLC (10:1 *v/v* n-Hex:EtOAc). After 4 h at 0°C, the reaction was quenched with sat. aq K_2CO_3 (100 mL), and the aqueous layer was extracted with CH_2Cl_2. The combined organic layers were dried over anhydrous Na_2SO_4, and the solvent was evaporated under reduced pressure. The crude product was purified by DCVC using n-Hex:Et_2O (95:5 *v/v*) as a mobile phase to give the title compound (211 mg, yield: 51%) as a white solid. The ^1H and ^{13}C NMR spectral data are listed in Table S14.

(5,6α-epoxysitgmasteryl)-stigmasterol ether (**18**). To a solution of 3β,3β'-distigmasteryl ether (**3**, 403 mg, 0.5 mmol) in CH_2Cl_2 at 0°C, a solution of mCPBA (149 mg, 0.6 mmol) in 10 mL CH_2Cl_2 was added dropwise and mixed for 4 h at 0°C. The reaction progression was checked by TLC (10:1 *v/v* n-Hex:EtOAc). After 4 h at 0°C, the reaction was quenched with sat. aq K_2CO_3 (100 mL), and the aqueous layer was extracted with CH_2Cl_2. The combined organic layers were dried over anhydrous Na_2SO_4, and the solvent was evaporated under reduced pressure. The crude product was purified by DCVC using n-Hex:Et_2O (95:5 *v/v*) as a mobile phase to give the title compound (221 mg, yield: 54%) as a white solid. The ^1H and ^{13}C NMR spectral data are listed in Table S14.

(5,6β-epoxycholesteryl)-cholesterol ether (**19**). To a solution of 3β,3β'-dicholesteryl ether (**1**, 378 mg, 0.5 mmol) in 15 mL CH_2Cl_2 and 15 mL *t*-BuOH, $Cu(MnO_4)_2$ (182 mg, 0.6 mmol) was added and stirred for 1 h. The reaction progression was checked by TLC (n-Hex:EtOAc 10:1 *v/v*). After the reaction, the mixture was added to 50 mL of n-Hex, filtered through a short silica pad and rinsed with n-Hex:EtOAc 3:1 (*v/v*). The solvent was evaporated, and the crude product was purified by DCVC using n-Hex:Et_2O (95:5 *v/v*) as a mobile phase to give the title compound (231 mg, yield: 60%) as a white solid. The ^1H and ^{13}C NMR spectral data are listed in Table S15.

(5,6β-epoxysitosteryl)-sitosterol ether (**20**). To a solution of 3β,3β'-disitosteryl ether (**2**, 402 mg, 0.5 mmol) of 15 mL CH_2Cl_2 and 15 mL *t*-BuOH, $Cu(MnO_4)_2$ (182 mg, 0.6 mmol) was added and stirred for 1 h. The reaction progression was checked by TLC (n-Hex:EtOAc 10:1 *v/v*). After the reaction, the mixture was added to 50 mL of n-Hex, filtered through a short silica pad and rinsed with n-Hex:EtOAc 3:1 (*v/v*). The solvent was evaporated, and the crude product was purified by DCVC using n-Hex:Et_2O (95:5 *v/v*) as a mobile phase to give the title compound (257 mg, yield: 63%) as a white solid. The ^1H and ^{13}C NMR spectral data are listed in Table S15.

(5,6β-epoxystigmasteryl)-stigmasterol ether (**21**). To a solution of 3β,3β'-distigmasteryl ether (**3**, 403 mg, 0.5 mmol) in 15 mL CH_2Cl_2 and 15 mL *t*-BuOH, $Cu(MnO_4)_2$ (182 mg, 0.6 mmol) was added and stirred for 1 h. The reaction progression was checked by TLC (n-Hex:EtOAc 10:1 *v/v*). After the reaction, the mixture was added to 50 mL of n-Hex, filtered through a short silica pad and rinsed with n-Hex:EtOAc 3:1 (*v/v*). The solvent was evaporated, and the crude product was purified by DCVC

using n-Hex:Et_2O (95:5 *v/v*) as a mobile phase to give the title compound (261 mg, yield: 64%) as a white solid. The ^{1}H and ^{13}C NMR spectral data are listed in Table S15.

Cholest-5-en-3β-yl acetate (Cholesteryl acetate) (**22**). Pyridine (593 mg, 7.5 mmol) and a solution of acetyl chloride (393 mg, 5.0 mmol) in CH_2Cl_2 (4 mL) were added to a solution of cholesterol (1.93 g, 5.0 mmol) in CH_2Cl_2 (40 mL). The reaction mixture was stirred at room temperature for 1 h. Water (10 mL) was added to the residue, and the mixture was extracted with CH_2Cl_2 (3 × 50 mL). The organic layer was washed sequentially with a 10% aqueous HCl solution (50 mL) of brine, dried over $MgSO_4$, and filtered. The filtrate was evaporated off, and the crude product was purified using DCVC with a mixture of n-Hex:EtOAc (6:1 *v/v*) as the mobile phase to give the title compound (1.964 g, yield: 92%) as a white solid. NMR data matched expected values [18]. ^{1}H NMR (500 MHz, $CDCl_3$, δ ppm): 0.67 (s, 3H, -CH3), 0.87 (m, 3H, -CH3), 0.92 (d, 3H, J = 6.5 Hz, -CH3), 1.01 (s, 3H, -CH3), 2.05 (s, 3H, -CH3), 2.32 (d, J = 7.0 Hz, 2H), 4.61 (m, 1H), 5.37 (s, 1H, =CH-), 1.06–2.53 (29H, methyl- and methylene- of steroid structure). ^{13}C NMR (125 MHz, $CDCl_3$, δ ppm): 170.50 (C=O), 139.63, 122.63, 73.97, 56.67, 56.13, 50.02, 42.30, 39.72, 39.51, 38.11, 36.98, 36.58, 36.17, 35.78, 31.88, 31.84, 28.22, 28.00, 27.76, 24.27, 23.81, 22.81, 22.55, 21.42, 21.02, 19.29, 18.70, 11.84.

Sitost-5-en-3β-yl acetate (β-Sitosteryl acetate) (**23**). Pyridine (593 mg, 7.5 mmol) and a solution of acetyl chloride (393 mg, 5.0 mmol) in CH_2Cl_2 (4 mL) were added to a solution of β-sitosterol (2.08 g, 5.0 mmol) in CH_2Cl_2 (40 mL). The reaction mixture was stirred at room temperature for 1 h. Water (10 mL) was added to the residue, and the mixture was extracted with CH_2Cl_2 (3 × 50 mL). The organic layer was washed sequentially with a 10% aqueous HCl solution (50 mL) of brine, dried over $MgSO_4$, and filtered. The filtrate was evaporated off, and the crude product was purified using DCVC with a mixture of n-Hex:EtOAc (6:1 *v/v*) as the mobile phase to give the title compound (2.078 g, yield: 91%) as a white solid. NMR data matched expected values [18]. ^{1}H NMR (500 MHz, $CDCl_3$, δ ppm) 5.38 (m, 1H), 4.60 (m, 1H), 2.32 (m, 2H), 2.03(s, 3H), 1.02 (s, 3H), 0.93 (d, J = 6.5 Hz, 3H), 0.85 (t, J = 7.3 Hz, 3H), 0.83 (d, J = 6.8 Hz, 3H), 0.81 (d, J = 6.8 Hz, 3H), 0.68 (s, 3H). ^{13}C NMR (125 MHz, $CDCl_3$, δ ppm) 170.51, 139.64, 122.63, 73.97, 56.68, 56.03, 50.03, 45.83, 42.30, 39.71, 38.11, 36.99, 36.58, 36.14, 33.93, 31.89, 31.85, 29.14, 28.23, 27.76, 26.07, 24.28, 23.06, 21.42, 21.02, 19.81, 19.30, 19.03, 18.77, 11.97, 11.84.

Stigmast-5-en-3β-yl acetate (Stigmasteryl acetate) (**24**). Pyridine (593 mg, 7.5 mmol) and a solution of acetyl chloride (393 mg, 5.0 mmol) in CH_2Cl_2 (4 mL) were added to a solution of stigmasterol (2.07 g, 5.0 mmol) in CH_2Cl_2 (40 mL). The reaction mixture was stirred at room temperature for 1 h. Water (10 mL) was added to the residue, and the mixture was extracted with CH_2Cl_2 (3 × 50 mL). The organic layer was washed sequentially with a 10% aqueous HCl solution (50 mL) of brine, dried over $MgSO_4$, and filtered. The filtrate was evaporated off, and the crude product was purified using DCVC with a mixture of n-Hex:EtOAc (6:1 *v/v*) as the mobile phase to give the title compound (2.116 g, yield: 93%) as a white solid. NMR data matched expected values [19]. ^{1}H NMR (500 MHz, $CDCl_3$, δ ppm) 5.38 (bd, J = 5.0 Hz, 1H), 5.15 (dd, J = 15.2, 8.6 Hz, 1H), 5.01 (dd, J = 15.2, 8.7 Hz, 1H), 4.61 (m, 1H), 2.33 (bs, 1H), 2.31 (bs, 1H), 2.07–1.92 (m, 3H), 2.03 (s, 3H), 1.88–1.83 (m, 2H), 1.75–1.64 (m, 1H), 1.63–1.36 (m, 9H), 1.30–0.90 (m, 8H), 1.02 (d, J = 6.6 Hz, 3H), 1.02 (s, 3H), 0.85 (d, J = 6.5 Hz, 3H), 0.81 (t, J = 7.4 Hz, 3H), 0.80 (d, J = 6.4 Hz, 3H), 0.70 (s, 3H). ^{13}C NMR (125 MHz, $CDCl_3$, δ ppm) 170.50, 139.64, 138.30, 129.27, 122.62, 73.96, 56.77, 55.93, 51.22, 50.04, 42.20, 40.48, 39.62, 38.11, 36.99, 36.59, 31.87, 31.85, 28.90, 27.76, 25.39, 24.34, 21.42, 21.21, 21.07, 21.00, 19.30, 18.97, 12.23, 12.03.

Cholest-5-en-7-one-3β-yl acetate (7-ketocholesterol acetate) (**25**). Chromium trioxide (CrO_3) (3.75 g, 37.5 mmol) was suspended in 150 mL of dry CH_2Cl_2 and stirred for 30 min at −20°C. 3,5-Dimethylpyrazole (3,61 g, 37.55 mmol) was added in one portion, and the reaction mixture was stirred for 30 min at −20°C. Cholesteryl acetate (**28**, 1.07 g, 2.50 mmol) was added, and the mixture was stirred at −20°C for 2 h and allowed to warm to room temperature. The solvent was evaporated in a rotavapor and redissolved in CH_2Cl_2, 100 mL of n-Hex:EtOAc (3:1 *v/v*) was added, and the brown suspension was filtered through a short pad of Celite and silica gel. The solvent was evaporated. The yellow crystals were purified by DCVC using n-Hex:EtOAC (6:1 *v/v*) as a mobile phase to give the compound (698 mg, yield: 63%). The ^1H and ^{13}C NMR spectral data matched the expected values [20]. ^1H NMR (500 MHz, $CDCl_3$, δ ppm) 5.70 (d, J = 1.6 Hz, 1H), 4.71 (m, 1H), 2.60-2.35 (m, 3H), 2.22 (br t, J = 9.0 Hz, 1H), 2.07-1.82 (m, 4H), 2.04 (s, 3H), 1.71-1.47 (m, 5H), 1.40-0.99 (m, 13H), 1.20 (s, 3H), 0.91 (d, J = 6.5 Hz, 3H), 0.87 (d, J = 6.6 Hz, 3H), 0.85 (d, J = 6.6 Hz, 3H), 0.68 (s, 3H). ^{13}C NMR (125 MHz, $CDCl_3$, δ ppm) 201.91, 170.26, 163.81, 126.70, 72.19, 54.77, 49.94, 49.80, 45.40, 43.10, 39.46, 38.65, 38.30, 37.73, 36.16, 35.7, 28.52, 27.98, 27.34, 26.29, 23.81, 22.79, 22.54, 21.24, 21.16, 18.85, 17.25, 11.96.

Sitost-5-en-7-one-3β-yl acetate (7-ketositosteryl acetate) (**26**). Chromium trioxide (CrO_3) (3.75 g, 37.5 mmol) was suspended in 150 mL of dry CH_2Cl_2 and stirred for 30 min at −20°C. 3,5-Dimethylpyrazole (3,61 g, 37.55 mmol) was added in one portion, and the reaction mixture was stirred for 30 min at −20°C. β-Sitosteryl acetate (**29**, 1.14 g, 2.50 mmol) was added, and the mixture was stirred at −20°C for 2 h and allowed to warm to room temperature. The solvent was evaporated in a rotavapor and redissolved in CH_2Cl_2, 100 mL of n-Hex:EtOAc (3:1 *v/v*) was added, and the brown suspension was filtered through a short pad of Celite and silica gel. The solvent was evaporated. The yellow crystals were purified by DCVC using n-Hex:EtOAC (6:1 *v/v*) as a mobile phase to give the compound (710 mg, yield: 60%). The ^1H and ^{13}C NMR spectral data matched the expected values [21]. ^1H NMR (500 MHz, $CDCl_3$, δ ppm) 5.70 (bd, J = 1.7 Hz, 1H), 4.72 (m, 1H), 2.60-2.35 (m, 3H), 2.23 (br t, J = 9.0 Hz, 1H), 2.05-1.85 (m, 3H), 2.05 (s, 3H), 1.75–1.45 (m, 6H), 1.40–0.95 (m, 14H), 1.21 (s, 3H), 0.93 (d, J = 6.6 Hz, 3H), 0.85 (t, J = 7.5 Hz, 3H), 0.83 (d, J = 6.7 Hz, 3H), 0.81 (d, J = 6.7 Hz, 3H) 0.69 (s, 3H). ^{13}C NMR (125 MHz, $CDCl_3$, δ ppm) 201.93, 170.26, 163.81, 126.70, 72.20, 54.67, 49.94, 49.80, 45.81, 45.41, 43.10, 38.64, 38.30, 37.73, 36.08, 35.99, 33.92, 29.11, 28.54, 27.35, 26.30, 26.07, 23.04, 21.24, 21.16, 19.78, 19.03, 18.91, 17.24, 11.96 (x2).

Stigmast-5-en-7-one-3β-yl acetate (7-ketostigmasteryl acetate) (**27**). Chromium trioxide (CrO_3) (3.75 g, 37.5 mmol) was suspended in 150 mL of dry CH_2Cl_2 and stirred for 30 min at −20°C. 3,5-Dimethylpyrazole (3.61 g, 37.55 mmol) was added in one portion, and the reaction mixture was stirred for 30 min at −20°C. Stigmasteryl acetate (**30**, 1.14 g, 2.50 mmol) was added, and the mixture was stirred at −20°C for 2 h and allowed to warm to room temperature. The solvent was evaporated in a rotavapor and redissolved in CH_2Cl_2, 100 mL of n-Hex:EtOAc (3:1 *v/v*) was added, and the brown suspension was filtered through a short pad of Celite and silica gel. The solvent was evaporated. The yellow crystals were purified by DCVC using n-Hex:EtOAC (6:1 *v/v*) as a mobile phase to the give compound (725 mg, yield: 61%). The ^1H and ^{13}C NMR spectral data matched the expected values [20]. ^1H NMR (500 MHz, $CDCl_3$, δ = ppm) 5.70 (d, J = 1.5 Hz, 1H), 5.18 (dd, J = 15.0 Hz, J = 8.5 Hz, 1H), 5.03 (dd, J = 15.0 Hz, J = 8.5 Hz, 1H), 4.72 (m, 1H), 2.55 (m, 1H), 2.47 (m, 1H), 2.38 (m, 1H), 2.10-1.92 (m, 4H), 2.05 (s, 3H), 1.83-1.48 (m, 8H), 1.47-1.10 (m, 13H), 1.21 (s, 3H), 1.04 (d, J = 6.7 Hz, 3H), 0.86 (d, J = 6.5 Hz, 3H), 0.81 (t, J = 7.3 Hz, 3H), 0.80 (d, J = 6.5 Hz, 3H), 0.70 (s,3H). ^{13}C NMR (125 MHz, $CDCl_3$, δ ppm) 201.83, 170.26, 163.79, 138.06, 129.48, 126.68, 72.20, 54.67, 51.19, 50.01, 49.81, 45.39, 42.99, 40.23, 38.53, 38.31, 37.74, 36.00, 31.87, 29.02, 27.35, 26.38, 25.35, 21.40, 21.24, 21.14, 21.04, 18.99, 17.24, 12.23, 12.18.

5-Cholesten-3β-ol-7-one (7-ketocholesterol) (**28**). Na_2CO_3 (318 mg, 3.0 mmol) was added at RT to a stirred solution of cholest-5-en-7-one-3β-yl acetate (**31**, 665 mg, 1.5 mmol) in 100 mL MeOH/H_2O (9:1 *v/v*). The resulting mixture was stirred at RT for 15 h. The organic solvents were evaporated, and the residue was diluted with water and extracted with EtOAc. The organic layer was washed with water and brine, dried over Na_2SO_4 and concentrated *in vacuo.* The resulting white solid was purified by DCVC using n-Hex:EtOAC (3:1 *v/v*) as a mobile phase to give the compound (458 mg, yield: 76%). The ^{1}H and ^{13}C NMR spectral data matched the expected values [22] and are listed in Table S1.

5-Sitosten-3β-ol-7-one (7-ketositosterol) (**29**). Na_2CO_3 (318 mg, 3.0 mmol) was added at RT to a stirred solution of sitost-5-en-7-one-3β-yl acetate (**32**, 705 mg, 1.5 mmol) in 100 mL MeOH/H_2O (9:1 *v/v*). The resulting mixture was stirred at RT for 15 h. The organic solvents were evaporated, and the residue was diluted with water and extracted with EtOAc. The organic layer was washed with water and brine, dried over Na_2SO_4 and concentrated *in vacuo*. The resulting white solid was purified by DCVC using n-Hex:EtOAC (3:1 *v/v*) as a mobile phase to give the compound (486 mg, yield: 75%). The ^{1}H and ^{13}C NMR spectral data matched the expected values [21] and are listed in Table S2.

5-Stigmasten-3β-ol-7-one (7-ketostigmasterol) (**30**). Na_2CO_3 (318 mg, 3.0 mmol) was added at RT to a stirred solution of stigmast-5-en-7-one-3β-yl acetate (**33**, 705 mg, 1.5 mmol) in 100 mL MeOH/H_2O (9:1 *v/v*). The resulting mixture was stirred at RT for 15 h. The organic solvents were evaporated, and the residue was diluted with water and extracted with EtOAc. The organic layer was washed with water and brine, dried over Na_2SO_4 and concentrated *in vacuo*. The resulting white solid was purified by DCVC using n-Hex:EtOAC (3:1 *v/v*) as a mobile phase to give the compound (508 mg, yield: 79%). The ^{1}H and ^{13}C NMR spectral data matched the expected values [20] and are listed in Table S2.

Cholesta-3,5-diene (cholestadiene) (**31**). Zinc(II) sulfate (180 mg, 1.0 mmol) was added to cholesterol (965 mg, 2.50 mmol), and the mixture was ground together into a fine powder with a mortar and pestle and transferred to a vial, which was sealed with a rubber stopper. The reaction was conducted at 220°C for 2 h. After the reaction, the product was dissolved in n-Hex and purified by DCVC using n-Hex as a mobile phase to give the compound (426 mg, yield: 46%). The ^{1}H and ^{13}C NMR spectral data are listed in Table S1. The product was a mixture of the isomers of cholesta-2,4-diene and cholesta-3,5-diene in a ratio, and because of an inability to resolve isomers, it was used as is.

Table S1. Mass spectrometry parameters during the analysis of the oxidized 3β,3'β-dicholesteryl ethers.

Compound	SIM	fragmentor	Cell accelerator Voltage	Capillary Voltage [V]	Corona Current [µA]	Nebulizer
**di-(7-ketocholesteryl) ether** di(7-kCh) (**7**)	784.0 383.3	120	1	6000	2	40
**7α-hydroxycholesteryl-cholesterol ether** 7α-hCh-Ch (**13**) / **7β-hydroxycholesteryl-cholesterol ether** 7β-hCh-Ch (**10**)	369.4 385.3	80	2	5400	2	30
**7-ketocholesteryl-cholesterol ether** 7-kCh-Ch (**4**)	385.3 770.0	120	3	6000	2	30
**7-ketositgmasteryl-stigmasterol ether (IS)** 7-kStig-Stig (**6**)	822.0	120	2	6000	2	30
**5,6α-epoxycholesteryl-cholesterol ether** 5,6α-epoxyCh-Ch (/ **5,6β-epoxycholesteryl-cholesterol ether** 5,6β-epoxyCh-Ch	369.4 403.3	80	2	5400	2	30

Table S2. Mass spectrometry parameters during the analysis of the oxidized 3β,3'β-disitosteryl ethers and 3β,3'β-distigmasteryl ethers.

Compound	SIM	fragmentor	Cell accelerator Voltage	Capillary Voltage [V]	Corona Current [μA]	Nebulizer
**di-(7-ketositosteryl) ether** di(7-kSito) (**8**)	840.1 411.5	120	1	6000	2	40
**di-(7-ketostigmasteryl) ether** di(7-kStig) (**9**)	836.1 409.4	120	1	6000	2	40
**7α-hydroxysitosteryl-sitosterol ether** 7α-hSito-Sito (**11**) / **7β-hydroxysitosteryl-sitosterol ether** 7β-hSito-Sito (**14**)	397.6 413.6	80	2	5400	2	30
**7α-hydroxystigmasteryl-stigmasterol ether** 7α-hStig-Stig (**12**) / **7β-hydroxystigmasteryl-stigmasterol ether** 7β-hStig-Stig (**15**)	395.4 411.4	80	2	5400	2	30
**7-ketositosteryl-sitosterol ether** 7-kSito-Sito (**5**)	826.3 429.9	120	2	5400	2	30
**7-ketositgmasteryl-stigmasterol ether** 7-kStig-Stig (**6**)	822.3 427.8	120	2	5400	2	30
**7-ketocholesteryl-cholesterol ether (IS)** 7-kCh-Ch (**4**)	770.0	120	3	5400	2	30
**5,6α-epoxysitosteryl-sitosterol ether** 5,6α-epoxySito-Sito (**23**) / **5,6β-epoxysitosteryl-sitosterol ether** 5,6β-epoxySito-Sito (**26**)	397.6 413.6	120	2	5400	2	30
**5,6α-epoxystigmasteryl-stigmasterol ether** 5,6α-epoxyStig-Stig (**24**) / **5,6β-epoxystigmasteryl-stigmasterol ether** 5,6β-epoxyStig-Stig (**27**)	395.4 411.4	120	2	5400	2	30

Table S3. Instrumental conditions of ICP-MS.

Parameter	Value
RF Power	1550 W
Plasma argon flow rate	15 L/min
Auxiliary argon flow rate	0.9 L/min
Nebulizer argon flow rate	1.07 L/min
Cell gas flow rate	4.3 mL/min
Dwell time	0.3 s
Sweeps	100
Number of readings per replicate	3
Conditions	Oxide 156/140 and doubly charged 70/140 < 2%

Table S4. Measurements of metal content in MK10, butter, cod liver oil, rapeseed oil and corn oil.

	**Montmorillonite K 10 (MK10)**			**Butter**	**Cod-liver oil**	**Rapeseed oil**	**Corn Oil**	
	Mean±SD	Unit		Mean±SD	Mean±SD	Mean	Mean	Unit
**^{7}Li**	12.86±0.40	**ppm**	**^{7}Li**	305.66±48.80	27.06±7.55	145.32±57.19	425.44±1.58	
**^{9}Be**	447.48±70.85	**ppb**	**^{9}Be**	< LOD	< LOD	< LOD	< LOD	
**^{11}B**	9.10±0.57	**ppm**	**^{11}B**	381.75±23.55	341.66±19.89	171.27±2.90	1223.11±643.62	
**^{23}Na***	787.75±61.59	**ppm**	**^{23}Na**	1148.37±106.16	488.13±304.06	690.11±40.84	2115.85±282.39	
**^{24}Mg**	0.551±0.017	**%**	**^{24}Mg**	493.36±34.57	57.38±9.01	7060.31±367.03	1537.64±80.22	
**^{27}Al**	4.13±0.13	**%**	**^{27}Al**	153.30±2.99	211.62±165.91	71.28±14.35	376.46±168.09	
**^{39}K***	650.91±45.64	**ppm**	**^{39}K**	3587.17±305.75	426.50±198.59	790.99±89.71	1226.52±65.68	
**^{43}Ca***	0.12±0.03	**%**	**^{43}Ca**	594.33±46.84	54.93±14.67	3952.15±232.37	250.96±53.63	
**^{51}V**	23.69±0.82	**ppm**	**^{51}V**	12.87±0.06	63.99±34.80	55.28±55.48	0.61±0.03	
**^{52}Cr**	17.76±0.72	**ppm**	**^{52}Cr**	3.95±1.25	1.34±0.08	10.59±6.75	5.25±1.99	
**^{55}Mn**	36.10±1.25	**ppm**	**^{55}Mn**	2.09±0.50	1.31±0.21	330.43±23.45	16.98±0.95	
**^{56}Fe**	1.23±0.02	**%**	**^{56}Fe**	76.84±0.90	74.78±40.91	390.40±147.33	271.26±2.51	
**^{59}Co**	849.47±52.82	**ppb**	**^{59}Co**	1.14±0.48	0.36±0.19	0.39±0.05	0.52±0.12	
**^{60}Ni**	3.66±0.35	**ppm**	**^{60}Ni**	30.29±15.72	7.30±6.10	6.83±2.23	24.68±17.28	
**^{63}Cu**	1.79±0.13	**ppm**	**^{63}Cu**	25.40±0.38	28.89±5.24	69.09±2.16	103.82±31.27	
**^{66}Zn**	1.24±0.43	**ppm**	**^{66}Zn**	175.47±16.00	41.12±34.45	307.25±38.22	117.95±55.34	
**^{71}Ga**	11.72±0.37	**ppm**	**^{71}Ga**	0.28±0.17	0.17±0.06	0.12±0.05	0.35±0.01	
**^{75}As**	4.46±0.21	**ppb**	**^{75}As**	0.86±0.41	0.37±0.33	0.72±0.23	1.03±0.49	
**^{78}Se**	< LOD	**ppb**	**^{78}Se**	2.55±1.16	3.08±0.08	2.70±1.04	2.29±0.25	
**^{85}Rb**	40.26±1.42	**ppm**	**^{85}Rb**	3.05±0.22	0.22±0.17	0.65±0.10	0.71±0.14	
**^{88}Sr**	15.12±0.53	**ppm**	**^{88}Sr**	5.82±1.74	5.01±1.63	150.99±11.05	16.95±1.61	
**^{95}Mo**	304.68±40.12	**ppb**	**^{95}Mo**	3.00±1.28	0.55±0.08	0.98±0.34	2.65±1.91	
**^{101}Ru**	< LOD	**ppb**	**^{101}Ru**	0.26±0.10	0.06±0.04	0.03±0.00	0.11±0.02	
**^{103}Rh**	0.03±0.04	**ppb**	**^{103}Rh**	0.23±0.09	0.04±0.02	< LOD	0.06±0.01	
**^{107}Ag**	190.36±12.34	**ppb**	**^{107}Ag**	9.74±1.41	4.45±0.05	27.55±17.74	2.20±0.17	
**^{111}Cd**	32.86±0.82	**ppb**	**^{111}Cd**	2.69±2.77	0.26±0.06	0.24±0.04	1.06±1.20	
**^{118}Sn**	2.57±0.03	**ppm**	**^{118}Sn**	2.60±2.36	0.530.14±	1.69±1.12	3.45±1.95	**ppb**
**^{121}Sb**	626.57±2.00	**ppb**	**^{121}Sb**	0.66±0.15	0.22±0.12	0.43±0.03	0.43±0.11	
**^{133}Cs**	3.35±0.06	**ppm**	**^{133}Cs**	0.23±0.09	0.09±0.04	0.03±0.00	0.13±0.00	
**^{137}Ba**	108.98±4.13	**ppm**	**^{137}Ba**	12.99±0.80	42.90±56.23	128.92±53.85	48.23±14.89	
**^{139}La**	2.94±0.05	**ppm**	**^{139}La**	6.47±0.09	4.452.27±	6.37±2.90	27.23±6.90	
**^{140}Ce**	5.76±0.20	**ppm**	**^{140}Ce**	1.10±0.63	1.96±0.26	0.91±0.14	1.96±0.88	
**^{141}Pr**	682.29±21.87	**ppb**	**^{141}Pr**	0.16±0.05	0.05±0.02	0.09±0.01	0.09±0.01	
**^{146}Nd**	2.70±0.09	**ppm**	**^{146}Nd**	0.20±0.08	0.09±0.04	0.36±0.02	0.18±0.02	
**^{147}Sm**	541.89±19.53	**ppb**	**^{147}Sm**	0.16±0.07	0.05±0.02	0.10±0.02	0.09±0.00	
**^{153}Eu**	102.52±4.44	**ppb**	**^{153}Eu**	0.14±0.05	0.04±0.02	0.04±0.00	0.08±0.00	
**^{157}Gd**	521.55±28.80	**ppb**	**^{157}Gd**	1.05±0.12	1.13±1.03	0.64±0.15	0.72±0.49	
**^{163}Dy**	438.89±17.39	**ppb**	**^{163}Dy**	0.15±0.05	0.03±0.01	0.12±0.02	0.080.01±	
**^{165}Ho**	83.91±3.38	**ppb**	**^{165}Ho**	0.15±0.05	0.03±0.01	0.02±0.00	0.07±0.00	
**^{166}Er**	253.73±10.21	**ppb**	**^{166}Er**	0.15±0.04	0.03±0.01	0.06±0.01	0.08±0.00	
**^{169}Tm**	37.35±1.73	**ppb**	**^{169}Tm**	0.14±0.05	0.03±0.01	0.01±0.00	0.07±0.00	
**^{172}Yb**	268.25±10.94	**ppb**	**^{172}Yb**	0.13±0.04	0.04±0.02	0.05±0.01	0.08±0.00	
**^{175}Lu**	38.75±1.64	**ppb**	**^{175}Lu**	0.14±0.06	0.03±0.01	0.01±0.00	0.06±0.00	
**^{189}Os**	< LOD	**ppb**	**^{189}Os**	2.17±1.20	0.45±0.02	0.62±0.13	1.14±0.43	
**^{193}Ir**	6.13±0.89	**ppb**	**^{193}Ir**	0.22±0.10	0.01±0.01	0.04±0.07	0.09±0.07	
**^{195}Pt**	18.76±1.26	**ppb**	**^{195}Pt**	0.36±0.11	0.09±0.03	0.11±0.08	0.13±0.02	
**^{197}Au**	< LOD	**ppb**	**^{197}Au**	2.54±1.11	0.43±0.02	0.91±0.23	1.65±0.29	
**^{201}Hg**	< LOD	**ppb**	**^{201}Hg**	4.96±1.10	1.38±0.01	2.68±0.87	3.86±0.63	
**^{205}Tl**	218.58±9.41	**ppb**	**^{205}Tl**	0.93±0.30	0.06±0.06	0.07±0.04	0.34±0.21	
**^{208}Pb**	2.25±0.10	**ppm**	**^{208}Pb**	50.61±10.78	23.43±13.50	42.07±22.01	42.61±17.12	
**^{232}Th**	5.13±0.33	**ppm**	**^{232}Th**	0.56±0.33	0.05±0.03	1.56±1.71	1.72±0.28	
**^{238}U**	264.47±14.42	**ppb**	**^{238}U**	0.22±0.12	0.16±0.11	0.62±0.02	0.18±0.03	

*The measurements were performed using AAS method.

Table S5. Validation parameters for LC-CAD method

	Range	Equation	R^2 (n=12)	LOD [µg/mL]	LOQ [µg/mL]
**Cholesterol**	0.35-25 mg/mL	$y=-0.1017x^2+6.8241x+9.2389$	0.995	16.2	53.9
**3β,3'β-dicholesteryl ether**	0.04-3.5 mg/mL	$y=-2.2896x^2+19.2679x+1.3320$	0.996	13.2	42.8
**Cholesta-3,5-diene**	0.10-10 mg/mL	$y=-0.3056x^2+6.7769x+1.9084$	0.995	4.1	13.7

Table S6. Validation parameters for LC-MS analysis of studied oxidized 3β,3'β-dicholesteryl ethers.

	Range	Equation	R^2 (n=8)	LOD [ng/mL]	LOQ [ng/mL]	Recovery	
**di-(7-ketocholesteryl) ether**	25-475 ng/ml	y=0.0068x+0.0316	0.9995	3.3	11.1	50 ng/ml	89,59%
						100 ng/ml	99,18%
						250 ng/ml	99,28%
**5,6α-epoxycholesteryl-cholesterol ether**		y=0.0012x+0.0150	0.9978	7.1	23.7	50 ng/ml	98,08%
						100 ng/ml	98,41%
						250 ng/ml	89,02%
**5,6β-epoxycholesteryl-cholesterol ether**		y=0.0019x+0.0220	0.9987	8.4	27.9	50 ng/ml	101,09%
						100 ng/ml	106,15%
						250 ng/ml	109,53%
**7-ketocholesteryl-cholesterol ether**		y=0.0045x+0.2053	0.9981	5.5	18.4	50 ng/ml	89,77%
						100 ng/ml	85,16%
						250 ng/ml	111,25%
**7α-hydroxycholesteryl-cholesterol ether**		y=0.0075x+0.0906	0.9990	6.7	22.3	50 ng/ml	97,52%
						100 ng/ml	100,77%
						250 ng/ml	100,14%
**7β-hydroxycholesteryl-cholesterol ether**		y=0.0123x+0.1620	0.9985	7.6	25.5	50 ng/ml	105,03%
						100 ng/ml	89,10%
						250 ng/ml	100,99%

Table S7. Validation parameters for LC-MS analysis of studied oxidized 3β,3'β-dicholesteryl ethers.

	Range	Equation	R^2 (n=8)	LOD [ng/mL]	LOQ [ng/mL]	Recovery	
**di-(7-ketositosteryl) ether** di(7-kSito) (**8**)	25-475 ng/ml	y=0.0067x-0.1948	0.9961	7.4	24.9	50 ng/ml	108.13%
						100 ng/ml	100.24%
						250 ng/ml	91.77%
**di-(7-ketostigmasteryl) ether** di(7-kStig) (**9**)		y=0.0030x-0.0604	09962	5.9	19.5	50 ng/ml	94.52%
						100 ng/ml	92.43%
						250 ng/ml	86.34%
**7α-hydroxysitosteryl-sitosterol ether** 7α-hSito-Sito (**11**)		y=0.0068x-0.1465	0.9978	10.1	33.5	50 ng/ml	95.40%
						100 ng/ml	103.17%
						250 ng/ml	79.44%
**7α-hydroxystigmasteryl-stigmasterol ether** 7α-hStig-Stig (**12**)		y=0.0045x-0.0497	0.9987	8.8	29.3	50 ng/ml	96.32%
						100 ng/ml	87.13%
						250 ng/ml	92.88%
**7β-hydroxysitosteryl-sitosterol ether** 7β-hSito-Sito (**14**)		y=0.0056x-0.1025	0.9973	11.0	36.7	50 ng/ml	89.34%
						100 ng/ml	106.78%
						250 ng/ml	81.47%
**7β-hydroxystigmasteryl-stigmasterol ether** 7β-hStig-Stig (**15**)		y=0.0030x+0.0071	0.9995	9.0	29.9	50 ng/ml	82.67%
						100 ng/ml	83.10%
						250 ng/ml	95.73%
**7-ketositosteryl-sitosterol ether** 7-kSito-Sito (**5**)		y=0.0046x+0.0607	0.9992	6.1	20.4	50 ng/ml	86.19%
						100 ng/ml	82.03%
						250 ng/ml	75.55%
**7-ketositgmasteryl-stigmasterol ether** 7-kStig-Stig (**6**)		y=0.0037x+0.0276	0.9995	8.5	28.1	50 ng/ml	97.00%
						100 ng/ml	90.60%
						250 ng/ml	84.31%
**5,6α-epoxysitosteryl-sitosterol ether** 5,6α-epoxySito-Sito (**23**)		y=0.0103x-0.1273	0.9975	6.4	21.2	50 ng/ml	86.98%
						100 ng/ml	100.66%
						250 ng/ml	84.40%
**5,6α-epoxystigmasteryl-stigmasterol ether** 5,6α-epoxyStig-Stig (**24**)		y=0.0071x-0.0048	0.9982	3.4	11.4	50 ng/ml	90.80%
						100 ng/ml	86.69%
						250 ng/ml	94.54%
**5,6β-epoxysitosteryl-sitosterol ether** 5,6β-epoxySito-Sito (**26**)		y=0.0073x-0.0992	0.9991	9.2	30.6	50 ng/ml	85.42%
						100 ng/ml	95.14%
						250 ng/ml	86.58%
**5,6β-epoxystigmasteryl-stigmasterol ether** 5,6β-epoxyStig-Stig (**27**)		y=0.0053x+0.0107	0.9986	10.3	34.4	50 ng/ml	87.01%
						100 ng/ml	88.80%
						250 ng/ml	94.99%

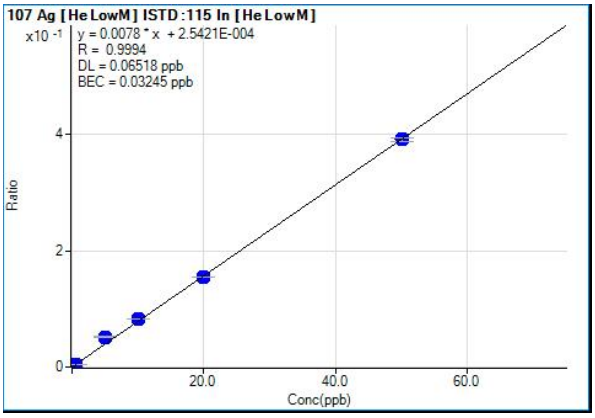
107 Ag [He LowM] ISTD:115 In [He LowM]
x10 -1
y = 0.0078 * x + 2.5421E-004
R = 0.9994
DL = 0.06518 ppb
BEC = 0.03245 ppb
Ratio
Conc(ppb)

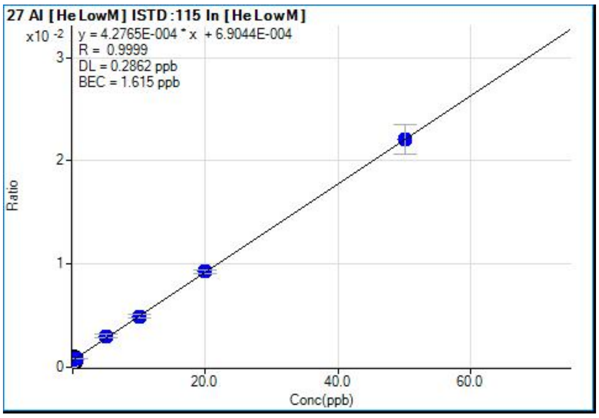
27 Al [He LowM] ISTD:115 In [He LowM]
x10 -2
y = 4.2765E-004 * x + 6.9044E-004
R = 0.9999
DL = 0.2862 ppb
BEC = 1.615 ppb
Ratio
Conc(ppb)

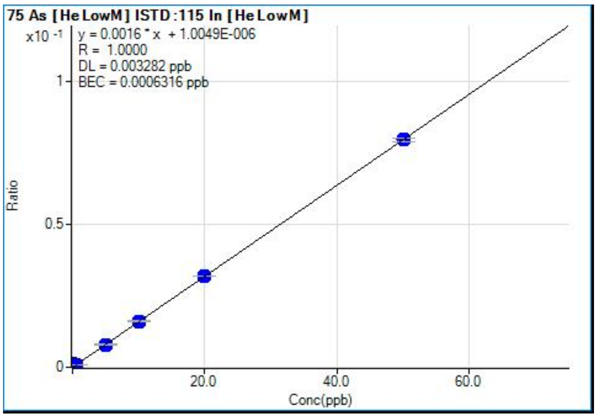
75 As [He LowM] ISTD:115 In [He LowM]
x10 -1
y = 0.0016 * x + 1.0049E-006
R = 1.0000
DL = 0.003282 ppb
BEC = 0.0006316 ppb
Ratio
Conc(ppb)

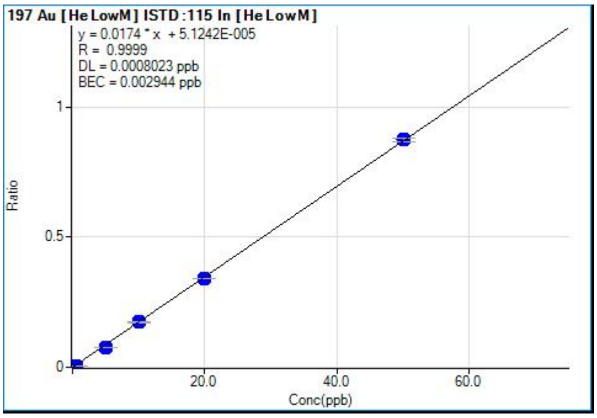
197 Au [He LowM] ISTD:115 In [He LowM]
y = 0.0174 * x + 5.1242E-005
R = 0.9999
DL = 0.0008023 ppb
BEC = 0.002944 ppb
Ratio
Conc(ppb)

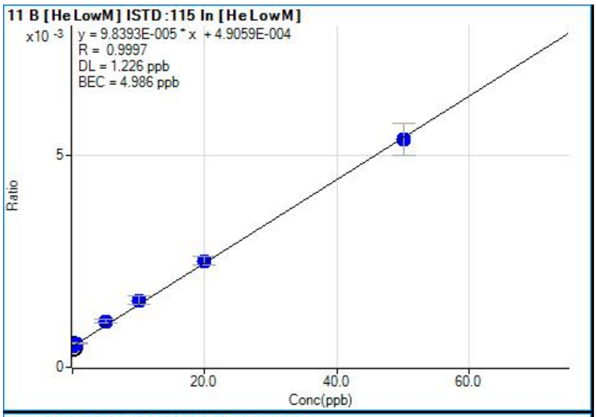
11 B [He LowM] ISTD:115 In [He LowM]
x10 -3
y = 9.8393E-005 * x + 4.9059E-004
R = 0.9997
DL = 1.226 ppb
BEC = 4.986 ppb
Ratio
Conc(ppb)

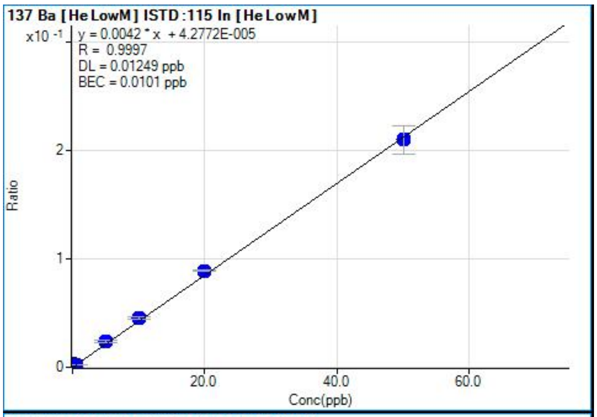
137 Ba [He LowM] ISTD:115 In [He LowM]
x10 -1
y = 0.0042 * x + 4.2772E-005
R = 0.9997
DL = 0.01249 ppb
BEC = 0.0101 ppb
Ratio
Conc(ppb)

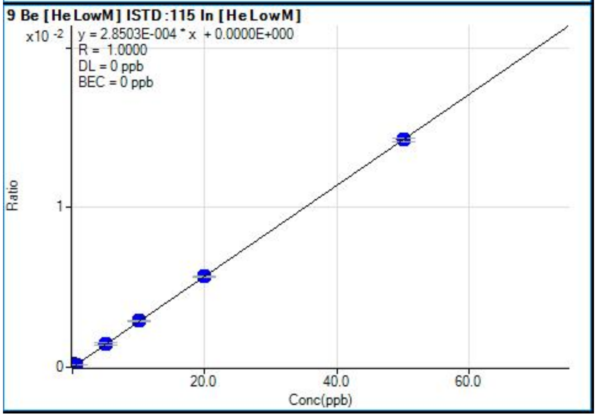
9 Be [He LowM] ISTD:115 In [He LowM]
x10 -2
y = 2.8503E-004 * x + 0.0000E+000
R = 1.0000
DL = 0 ppb
BEC = 0 ppb
Ratio
Conc(ppb)

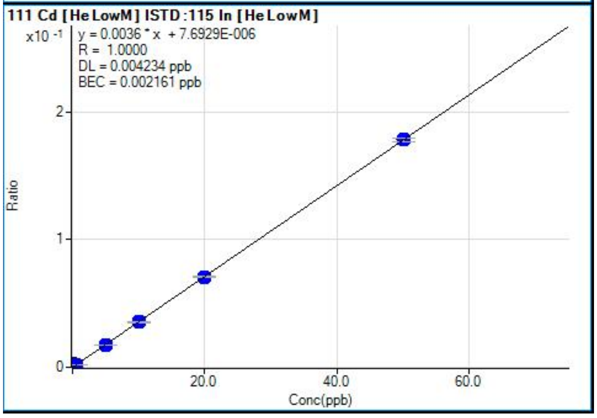
111 Cd [He LowM] ISTD:115 In [He LowM]
x10 -1
y = 0.0036 * x + 7.6929E-006
R = 1.0000
DL = 0.004234 ppb
BEC = 0.002161 ppb
Ratio
Conc(ppb)

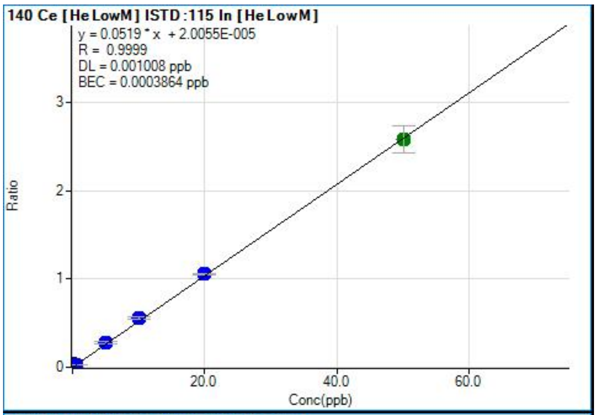
140 Ce [He LowM] ISTD :115 In [He LowM]
y = 0.0519 * x + 2.0055E-005
R = 0.9999
DL = 0.001008 ppb
BEC = 0.0003864 ppb
Ratio
Conc(ppb)

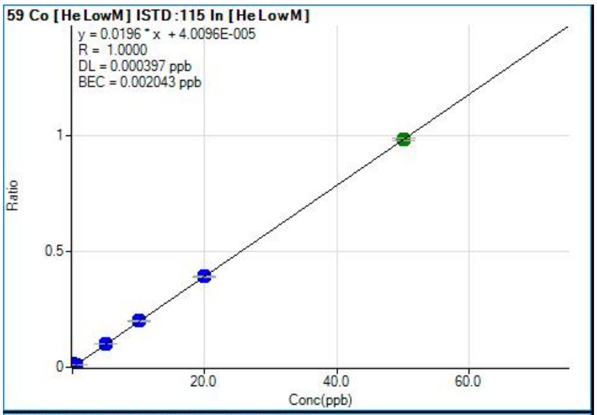
59 Co [He LowM] ISTD :115 In [He LowM]
y = 0.0196 * x + 4.0096E-005
R = 1.0000
DL = 0.000397 ppb
BEC = 0.002043 ppb
Ratio
Conc(ppb)

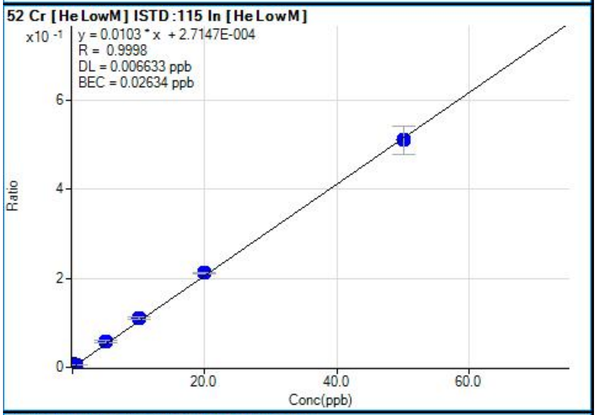
52 Cr [He LowM] ISTD :115 In [He LowM]
x10 -1
y = 0.0103 * x + 2.7147E-004
R = 0.9998
DL = 0.006633 ppb
BEC = 0.02634 ppb
Ratio
Conc(ppb)

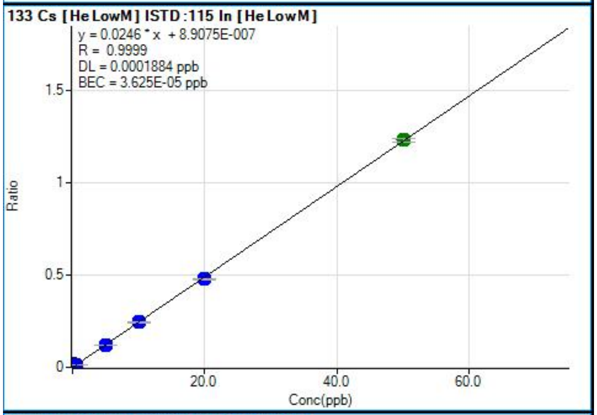
133 Cs [He LowM] ISTD :115 In [He LowM]
y = 0.0246 * x + 8.9075E-007
R = 0.9999
DL = 0.0001884 ppb
BEC = 3.625E-05 ppb
Ratio
Conc(ppb)

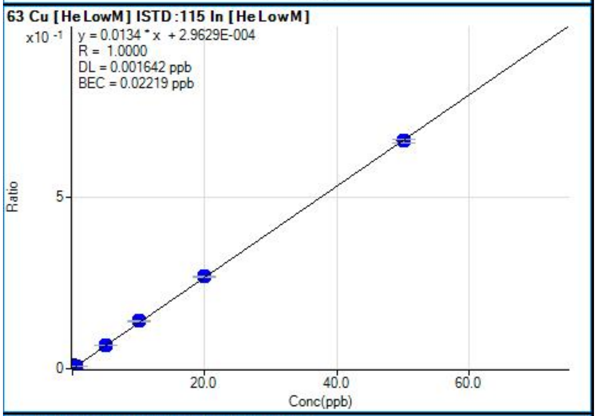
63 Cu [He LowM] ISTD :115 In [He LowM]
x10 -1
y = 0.0134 * x + 2.9629E-004
R = 1.0000
DL = 0.001642 ppb
BEC = 0.02219 ppb
Ratio
Conc(ppb)

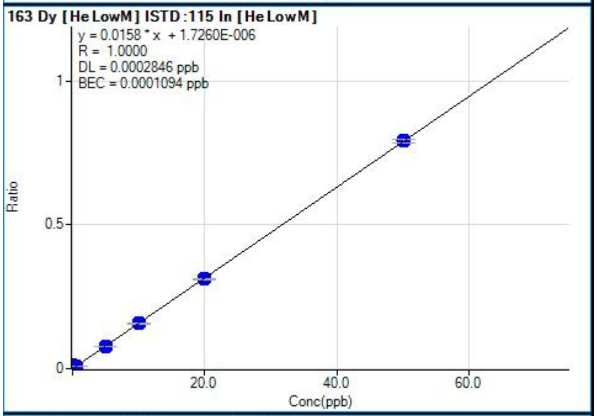
163 Dy [He LowM] ISTD :115 In [He LowM]
y = 0.0158 * x + 1.7260E-006
R = 1.0000
DL = 0.0002846 ppb
BEC = 0.0001094 ppb
Ratio
Conc(ppb)

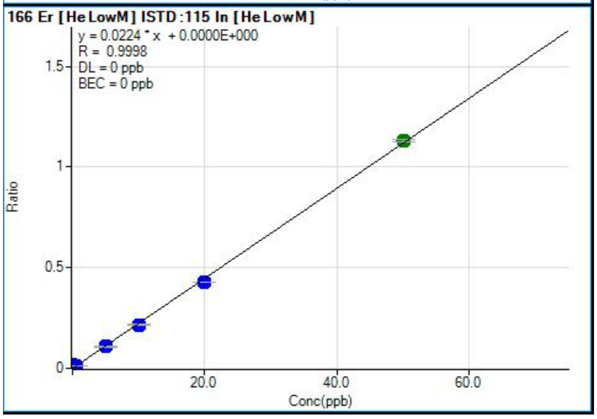
166 Er [He LowM] ISTD :115 In [He LowM]
y = 0.0224 * x + 0.0000E+000
R = 0.9998
DL = 0 ppb
BEC = 0 ppb
Ratio
Conc(ppb)

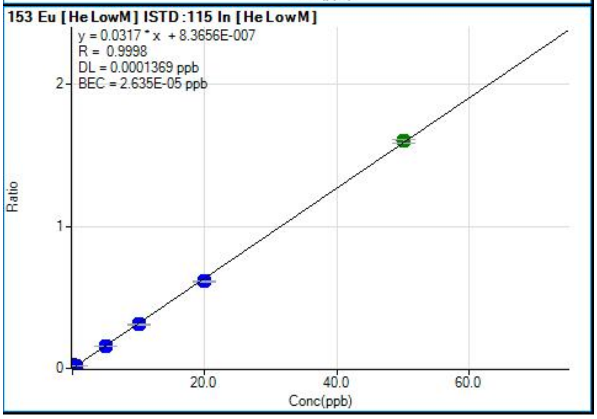
153 Eu [He LowM] ISTD :115 In [He LowM]
y = 0.0317 * x + 8.3656E-007
R = 0.9998
DL = 0.0001369 ppb
BEC = 2.635E-05 ppb
Ratio
Conc(ppb)

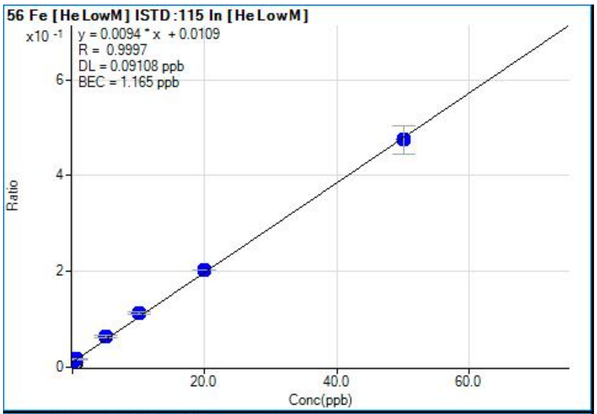
56 Fe [He LowM] ISTD:115 In [He LowM]
y = 0.0094 * x + 0.0109
R = 0.9997
DL = 0.09108 ppb
BEC = 1.165 ppb
x10 -1
Ratio
Conc(ppb)

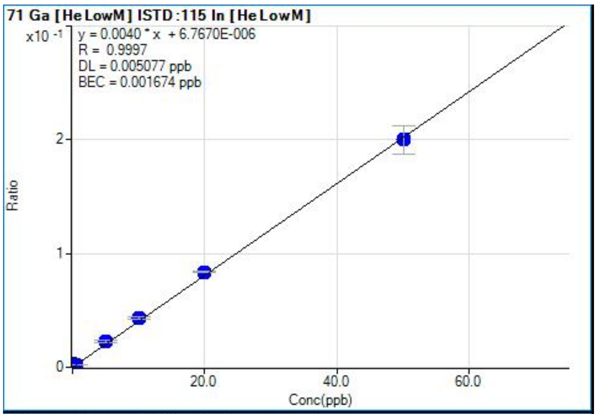
71 Ga [He LowM] ISTD:115 In [He LowM]
y = 0.0040 * x + 6.7670E-006
R = 0.9997
DL = 0.005077 ppb
BEC = 0.001674 ppb
x10 -1
Ratio
Conc(ppb)

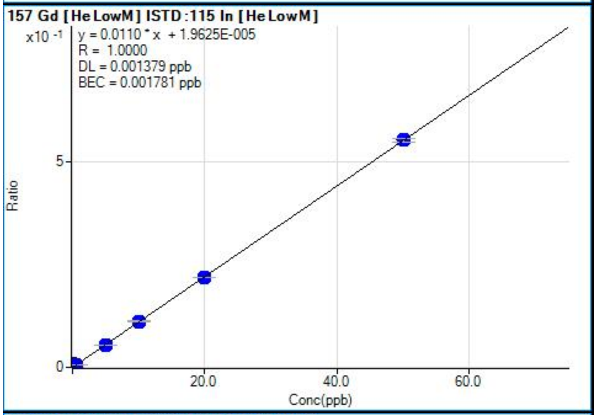
157 Gd [He LowM] ISTD:115 In [He LowM]
y = 0.0110 * x + 1.9625E-005
R = 1.0000
DL = 0.001379 ppb
BEC = 0.001781 ppb
x10 -1
Ratio
Conc(ppb)

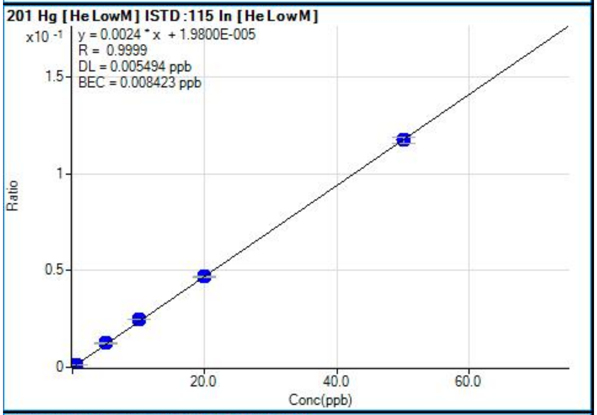
201 Hg [He LowM] ISTD:115 In [He LowM]
y = 0.0024 * x + 1.9800E-005
R = 0.9999
DL = 0.005494 ppb
BEC = 0.008423 ppb
x10 -1
Ratio
Conc(ppb)

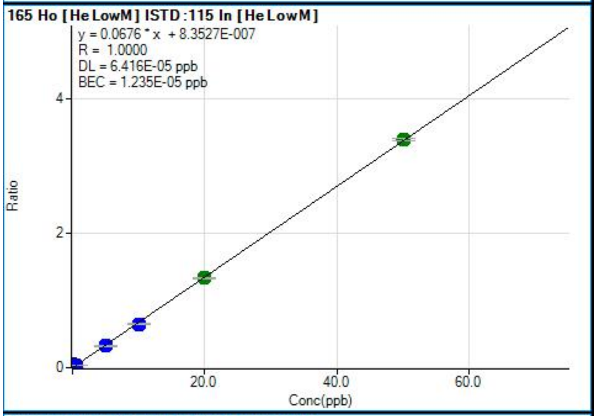
165 Ho [He LowM] ISTD:115 In [He LowM]
y = 0.0676 * x + 8.3527E-007
R = 1.0000
DL = 6.416E-05 ppb
BEC = 1.235E-05 ppb
Ratio
Conc(ppb)

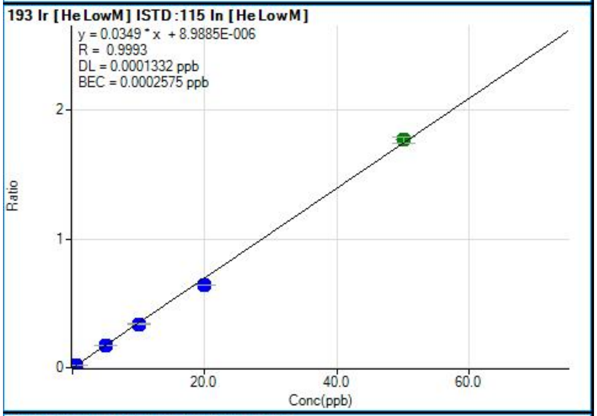
193 Ir [He LowM] ISTD:115 In [He LowM]
y = 0.0349 * x + 8.9885E-006
R = 0.9993
DL = 0.0001332 ppb
BEC = 0.0002575 ppb
Ratio
Conc(ppb)

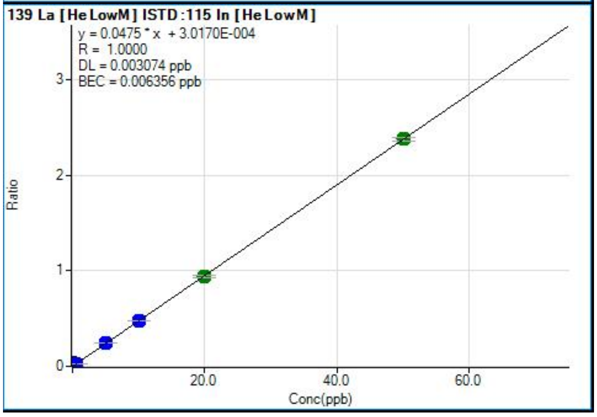
139 La [He LowM] ISTD:115 In [He LowM]
y = 0.0475 * x + 3.0170E-004
R = 1.0000
DL = 0.003074 ppb
BEC = 0.006356 ppb
Ratio
Conc(ppb)

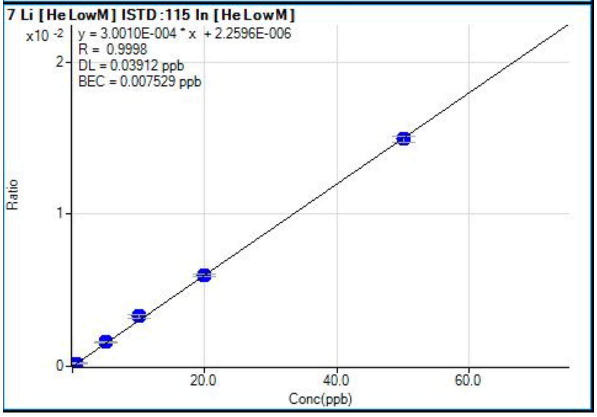
7 Li [He LowM] ISTD:115 In [He LowM]
y = 3.0010E-004 * x + 2.2596E-006
R = 0.9998
DL = 0.03912 ppb
BEC = 0.007529 ppb
x10 -2
Ratio
Conc(ppb)

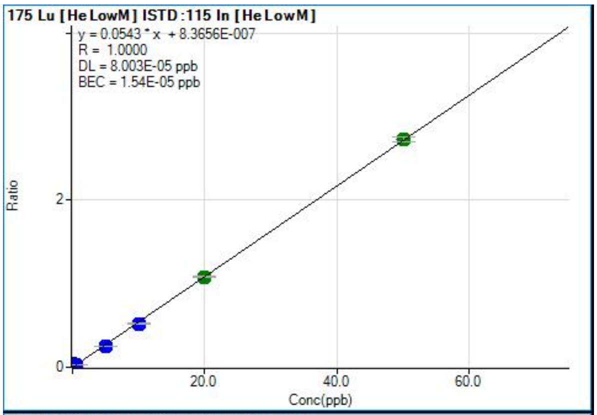
175 Lu [He LowM] ISTD :115 In [He LowM]
y = 0.0543 * x + 8.3656E-007
R = 1.0000
DL = 8.003E-05 ppb
BEC = 1.54E-05 ppb
Ratio
Conc(ppb)

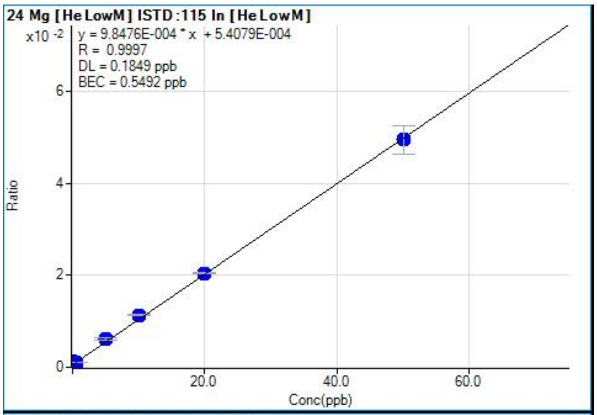
24 Mg [He LowM] ISTD :115 In [He LowM]
x10 -2
y = 9.8476E-004 * x + 5.4079E-004
R = 0.9997
DL = 0.1849 ppb
BEC = 0.5492 ppb
Ratio
Conc(ppb)

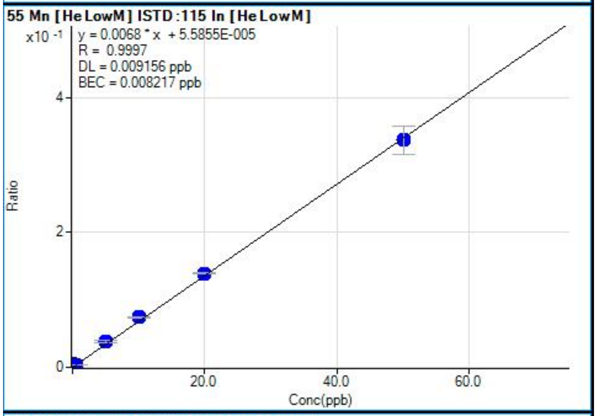
55 Mn [He LowM] ISTD :115 In [He LowM]
x10 -1
y = 0.0068 * x + 5.5855E-005
R = 0.9997
DL = 0.009156 ppb
BEC = 0.008217 ppb
Ratio
Conc(ppb)

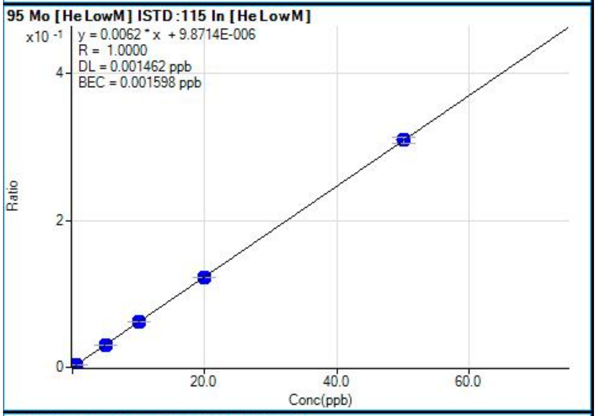
95 Mo [He LowM] ISTD :115 In [He LowM]
x10 -1
y = 0.0062 * x + 9.8714E-006
R = 1.0000
DL = 0.001462 ppb
BEC = 0.001598 ppb
Ratio
Conc(ppb)

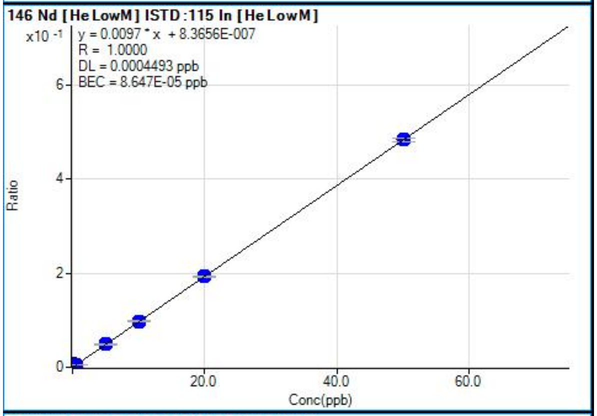
146 Nd [He LowM] ISTD :115 In [He LowM]
x10 -1
y = 0.0097 * x + 8.3656E-007
R = 1.0000
DL = 0.0004493 ppb
BEC = 8.647E-05 ppb
Ratio
Conc(ppb)

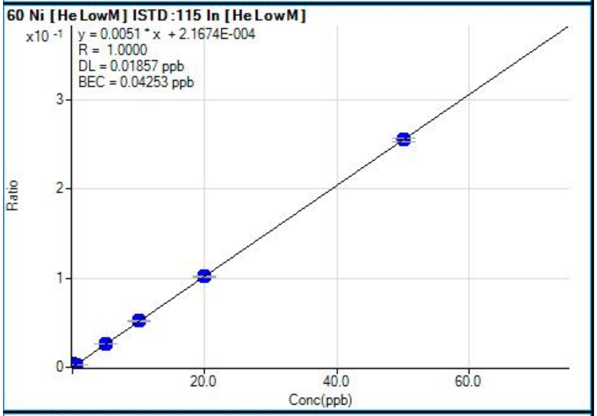
60 Ni [He LowM] ISTD :115 In [He LowM]
x10 -1
y = 0.0051 * x + 2.1674E-004
R = 1.0000
DL = 0.01857 ppb
BEC = 0.04253 ppb
Ratio
Conc(ppb)

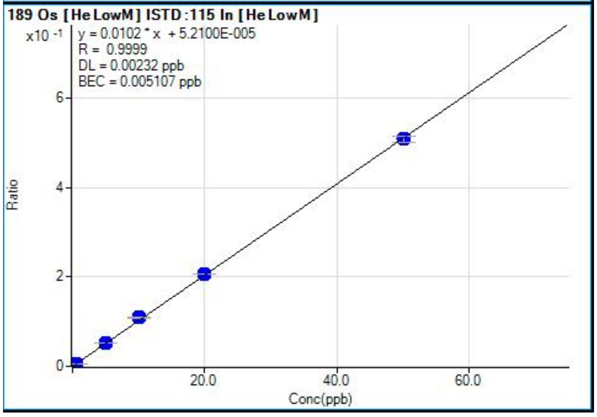
189 Os [He LowM] ISTD :115 In [He LowM]
x10 -1
y = 0.0102 * x + 5.2100E-005
R = 0.9999
DL = 0.00232 ppb
BEC = 0.005107 ppb
Ratio
Conc(ppb)

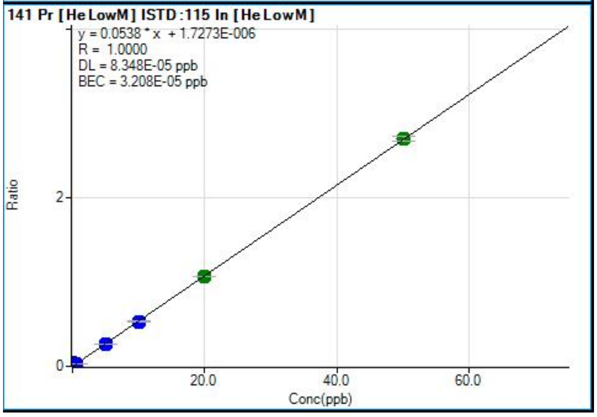
141 Pr [He LowM] ISTD :115 In [He LowM]
y = 0.0538 * x + 1.7273E-006
R = 1.0000
DL = 8.348E-05 ppb
BEC = 3.208E-05 ppb
Ratio
Conc(ppb)

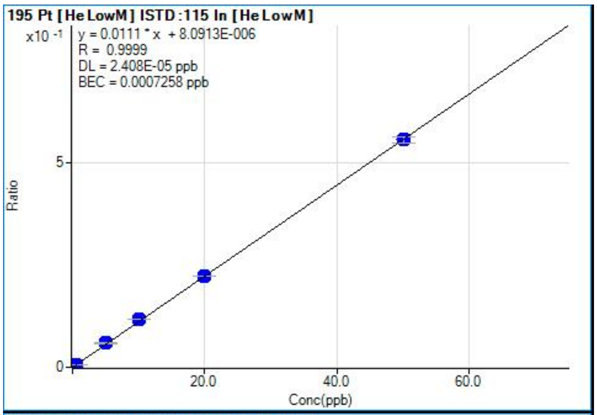
195 Pt [He LowM] ISTD :115 In [He LowM]
x10 -1
y = 0.0111 * x + 8.0913E-006
R = 0.9999
DL = 2.408E-05 ppb
BEC = 0.0007258 ppb
Ratio
Conc(ppb)

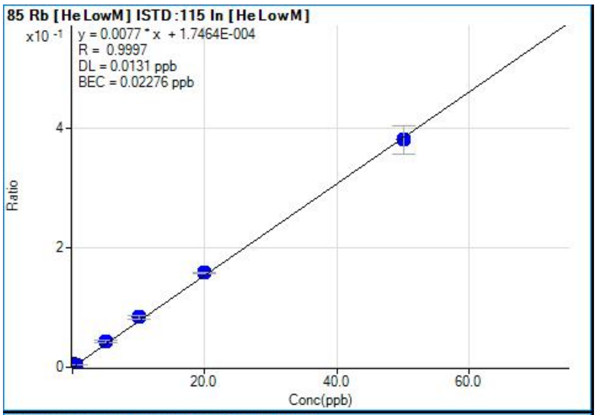
85 Rb [He LowM] ISTD :115 In [He LowM]
x10 -1
y = 0.0077 * x + 1.7464E-004
R = 0.9997
DL = 0.0131 ppb
BEC = 0.02276 ppb
Ratio
Conc(ppb)

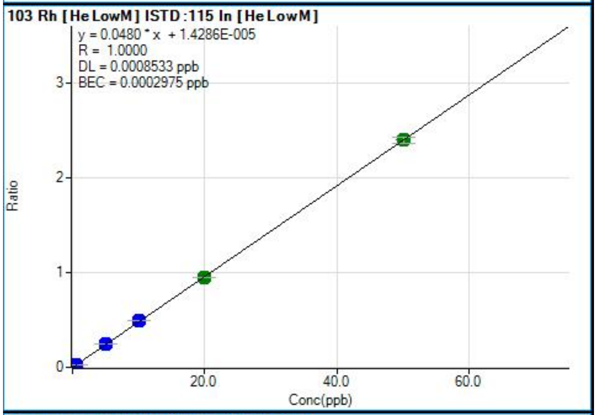
103 Rh [He LowM] ISTD :115 In [He LowM]
y = 0.0480 * x + 1.4286E-005
R = 1.0000
DL = 0.0008533 ppb
BEC = 0.0002975 ppb
Ratio
Conc(ppb)

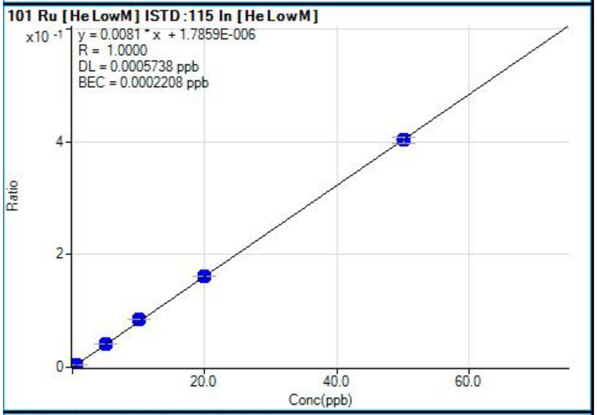
101 Ru [He LowM] ISTD :115 In [He LowM]
x10 -1
y = 0.0081 * x + 1.7859E-006
R = 1.0000
DL = 0.0005738 ppb
BEC = 0.0002208 ppb
Ratio
Conc(ppb)

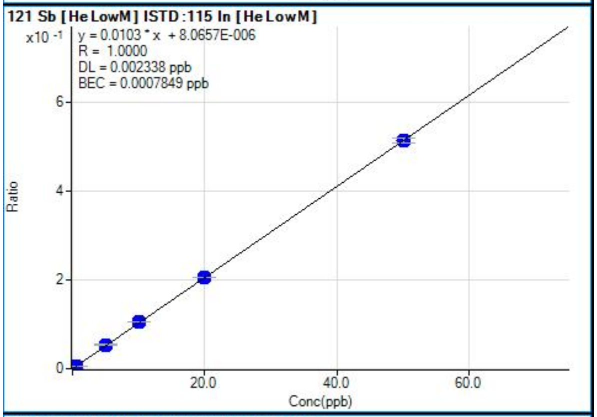
121 Sb [He LowM] ISTD :115 In [He LowM]
x10 -1
y = 0.0103 * x + 8.0657E-006
R = 1.0000
DL = 0.002338 ppb
BEC = 0.0007849 ppb
Ratio
Conc(ppb)

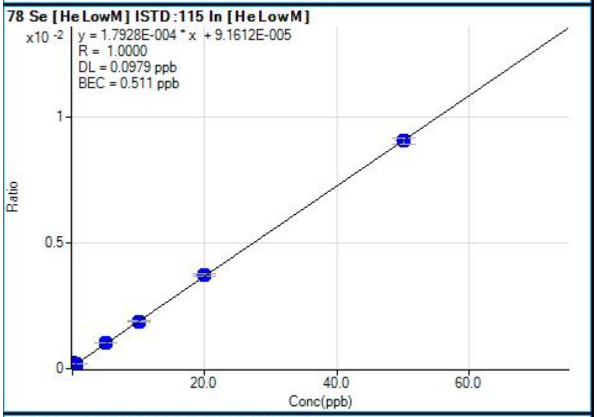
78 Se [He LowM] ISTD :115 In [He LowM]
x10 -2
y = 1.7928E-004 * x + 9.1612E-005
R = 1.0000
DL = 0.0979 ppb
BEC = 0.511 ppb
Ratio
Conc(ppb)

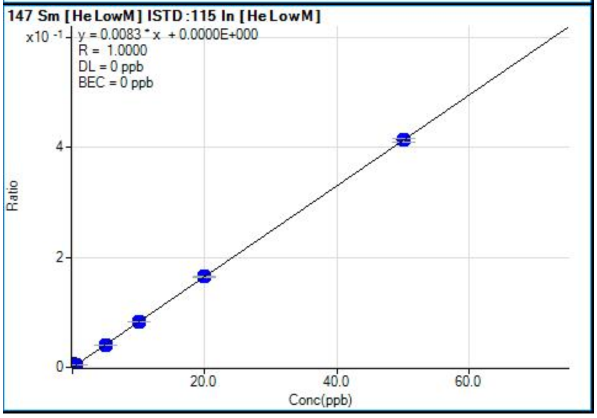
147 Sm [He LowM] ISTD :115 In [He LowM]
x10 -1
y = 0.0083 * x + 0.0000E+000
R = 1.0000
DL = 0 ppb
BEC = 0 ppb
Ratio
Conc(ppb)

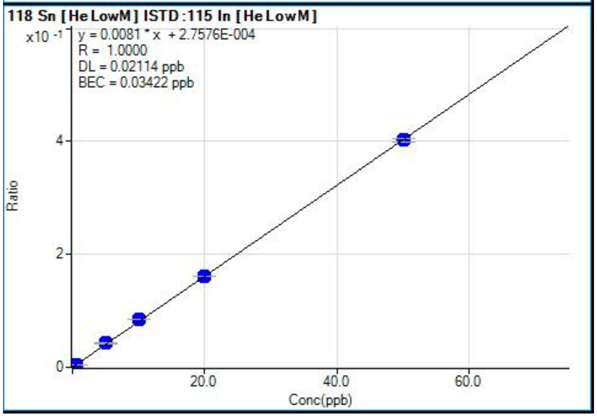
118 Sn [He LowM] ISTD :115 In [He LowM]
x10 -1
y = 0.0081 * x + 2.7576E-004
R = 1.0000
DL = 0.02114 ppb
BEC = 0.03422 ppb
Ratio
Conc(ppb)

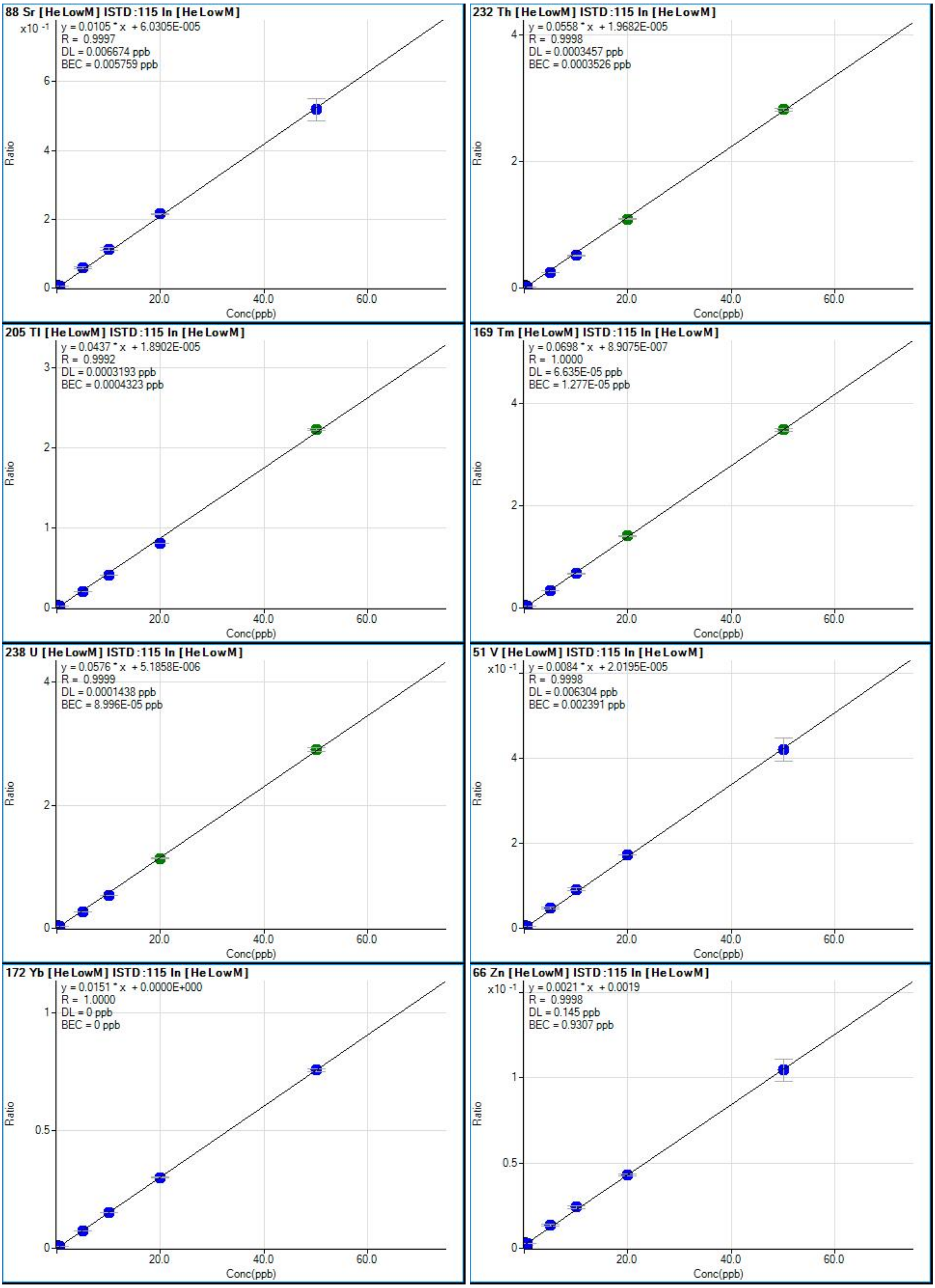

Fig S1. Linearity curves for all 54 metals measured by ICP-MS.

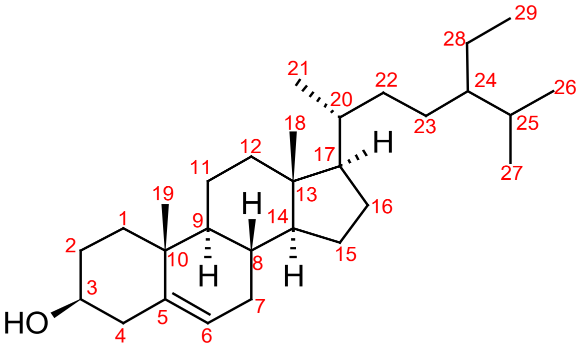

Figure S2. Steroids numbering system

Atom No.	3β,3’β-dicholesteryl ether (1)		Cholest-5-en-7-one-3β-yl-cholesterol ether (7-ketocholesteryl-cholesterol ether) (4)				5-Cholesten-3β-ol-7-one (28)		Cholesta-3,5-diene (31)	
			native sterol part		*7-keton part*					
	^{1}H NMR δ (ppm)	^{13}C δ (ppm)	^{1}H NMR δ (ppm)	^{13}C δ (ppm)	^{1}H NMR δ (ppm)	^{13}C δ (ppm)	^{1}H NMR δ (ppm)	^{13}C δ (ppm)	^{1}H NMR δ (ppm)	^{13}C δ (ppm)
**1**	1.04, 1.84	37.43	1.04, 1.86	37.31	1.16, 1.94	36.47	1.20, 1.95	36.34	1.16, 1.31	33.78
**2**	1.82, 1.49	29.43	1.82, 1.50	29.30	1.93, 1.59	29.14	1.94, 1.62	31.17	2.18, 2.10	23.05
**3**	3.28	76.34	3.29	76.87	3.41	75.05	3.67	70.49	5.59	124.99
**4**	2.28, 2.22	40.06	2.27, 2.23	39.98	2.49, 2.37	39.90	2.51, 2.40	41.81	5.93	128.99
**5**	-	141.35	-	140.96	-	165.63	-	165.18	-	141.47
**6**	5.33	121.33	5.34	121.66	5.67	125.93	5.69	126.08	5.39	123.15
**7**	1.52, 1.97	31.96	1.51, 1.97	31.94	-	202.33	-	202.35	1.68, 2.16	31.77
**8**	1.44	31.90	1.45	31.89	2.23	45.39	2.24	45.40	1.62	31.78
**9**	0.91	50.25	0.92	50.22	1.48	50.01	1.50	49.92	1.00	48.39
**10**	-	36.86	-	36.83	-	38.59	-	38.27	-	35.18
**11**	1.45, 1.49	21.06	1.45, 1.49	21.06	1.57, 1.57	21.19	1.54, 1.57	21.22	1.55, 1.41	20.96
**12**	1.15, 2.01	39.80	1.15, 2.01	39.77	1.12, 2.03	38.73	1.13, 2.03	38.70	1.18, 2.02	39.82
**13**	-	42.32	-	42.32	-	43.09	-	43.09	-	42.46
**14**	0.98	56.79	0.99	56.77	1.33	49.97	1.30	49.96	1.05	56.96
**15**	1.06, 1.57	24.29	1.06, 1.57	24.29	1.24, 2.40	26.31	1.24, 2.40	26.31	1.08, 1.59	24.18
**16**	1.82, 1.26	28.23	1.82, 1.26	28.23	1.89, 1.27	28.55	1.89, 1.27	28.54	1.85, 1.27	28.24
**17**	1.08	56.16	1.09	56.15	1.08	54.80	1.08	54.78	1.10	56.16
**18**	0.67	11.85	0.676	11.85	0.679	11.96	0.68	11.96	0.71	11.97
**19**	1.00	19.39	1.00	19.37	1.18	17.27	1.20	17.30	0.96	18.77
**20**	1.37	35.78	1.37	35.78	1.37	35.70	1.37	35.70	1.38	35.81
**21**	0.91	18.71	0.91	18.72	0.92	18.87	0.92	18.86	0.92	18.71
**22**	1.33, 0.99	36.19	1.34, 1.00	36.18	1.34, 1.00	36.18	1.34, 1.01	36.17	1.34, 1.00	36.19
**23**	1.33, 1.14	23.82	1.34, 1.14	23.82	1.34, 1.14	23.82	1.34, 1.13	23.82	1.34, 1.14	23.84
**24**	1.12, 1.12	39.52	1.12, 1.12	39.52	1.12, 1.12	39.47	1.12, 1.12	39.47	1.12, 1.12	39.52
**25**	1.52	28.01	1.52	28.01[a]	1.51	27.99[a]	1.51	27.99	1.52	28.01
**26**	0.86	22.56	0.86	22.56[b]	0.86	22.55[b]	0.86	22.55	0.86	22.56
**27**	0.87	22.81	0.87	22.81[c]	0.87	22.80[c]	0.87	22.80	0.87	22.81

[a, b, c] Groups of resonances ambiguously assigned (could be interchanged within the group)

Table S8. ^{1}H and ^{13}C NMR chemical shifts of synthesized **1**, **4**, **28** and **31** steroids

Atom No.	3β,3'β-disitosteryl ether (2)		Sitost-5-en-7-one-3β-yl-sitosterol ether (7-ketositosteryl-sitosterol ether) (5) *native sterol part*		*7-keton part*		5-Sitosten-3β-ol-7-one (29)	
	^{1}H NMR δ (ppm)	^{13}C δ (ppm)	^{1}H NMR δ (ppm)	^{13}C δ (ppm)	^{1}H NMR δ (ppm)	^{13}C δ (ppm)	^{1}H NMR δ (ppm)	^{13}C δ (ppm)
**1**	1.03, 1.84	37.42	1.04, 1.85	37.31	1.16, 1.94	36.47	1.21, 1.95	36.34
**2**	1.82, 1.49	29.43	1.82, 1.50	29.30	1.93, 1.58	29.13	1.94, 1.62	31.16
**3**	-	76.34	3.29	76.87	3.41	75.05	3.68	70.49
**4**	2.28, 2.22	40.06	2.27, 2.23	39.98	2.49, 2.37	39.90	2.51, 2.40	41.80
**5**	-	141.35	-	140.96	-	165.64	-	165.16
**6**	5.33	121.32	5.34	121.65	5.67	125.92	5.69	126.08
**7**	1.52, 1.97	31.96	1.53, 1.97	31.94	-	202.34	-	202.36
**8**	1.43	31.90	1.45	31.89	2.23	45.39	2.24	45.40
**9**	0.92	50.25	0.92	50.21	1.48	50.01	1.51	49.92
**10**	-	36.85	-	36.83	-	38.59	-	38.27
**11**	1.44, 1.49	21.06	1.49, 1.45	21.05	1.57, 1.57	21.19	1.57, 1.57	21.20
**12**	1.16, 2.01	39.79	1.16, 2.01	39.76	1.13, 2.03	38.72	1.13, 2.04	38.68
**13**	-	42.32	-	42.32	-	43.08	-	43.08
**14**	0.99	56.79	0.99	56.76	1.33	49.97	1.33	49.95
**15**	1.06, 1.57	24.30	1.06, 1.57	24.29	1.24, 2.40	26.32	1.26, 2.41	26.31
**16**	1.84, 1.26	28.24	1.84, 1.26	28.23	1.91, 1.28	28.55	1.91, 1.27	28.54
**17**	1.10	56.05	1.10	56.05	1.09	54.70	1.10	54.69
**18**	0.68	11.85	0.68	11.96	0.68	11.85	0.68	11.96
**19**	1.00	19.39	1.00	19.36	1.18	17.26	1.20	17.30
**20**	1.36	36.13	1.36	36.13	1.36	36.06	1.36	36.07
**21**	0.92	18.78	0.92	18.77	0.92	18.92	0.93	18.91
**22**	1.32, 1.01	33.94	1.33, 1.02	33.94	1.33, 1.02	33.94	1.34, 1.03	33.93
**23**	1.16	26.06	1.16	26.07	1.16	26.07	1.16	26.07
**24**	0.93	45.83	0.93	45.83[a]	0.93	45.81[a]	0.93	45.81
**25**	1.66	29.14	1.67	29.12	1.67	29.12	1.67	29.11
**26**	0.81	19.03	0.81	19.03	0.81	19.03	0.81	19.03
**27**	0.83	19.81	0.83	19.78	0.83	19.78	0.83	19.79
**28**	1.25	23.06	1.25	23.06[b]	1.25	23.04[b]	1.25	23.04
**29**	0.85	11.98	0.85	11.97	0.85	11.97	0.85	11.96

[a, b] Groups of resonances ambiguously assigned (could be interchanged within the group)

Table S9. ^{1}H and ^{13}C NMR chemical shifts of synthesized **2**, **5** and **29** steroids

Atom No.	**3β,3'β-distigmasteryl ether** (**3**)		**Stigmast-5-en-7-one-3β-yl-stigmasterol ether** (7-ketostigmasteryl-stigmasterol ether) (**6**) *native sterol part*		*7-keton part*		**5-Stigmasten-3β-ol-7-one** (**30**)	
	**^{1}H NMR δ (ppm)**	**^{13}C δ (ppm)**	**^{1}H NMR δ (ppm)**	**^{13}C δ (ppm)**	**^{1}H NMR δ (ppm)**	**^{13}C δ (ppm)**	**^{1}H NMR δ (ppm)**	**^{13}C δ (ppm)**
**1**	1.04, 1.84	37.41	1.04, 1.86	37.31	1.17, 1.94	36.47	1.21, 1.96	36.34
**2**	1.82, 1.49	29.43	1.82, 1.50	29.31	1.93, 1.59	29.14	1.94, 1.62	31.15
**3**	3.28	76.33	3.29	76.87	3.41	75.05	3.67	70.47
**4**	2.28, 2.22	40.04	2.27, 2.23	39.98	2.50, 2.37	39.90	2.51, 2.40	41.81
**5**	-	141.32	-	140.95	-	165.62	-	165.17
**6**	5.33	121.30	5.34	121.65	5.67	125.91	5.69	126.05
**7**	1.52, 1.96	31.93	1.51, 1.97	31.93	-	202.25	-	202.27
**8**	1.45	31.89	1.45	31.88	2.23	45.36	2.24	45.37
**9**	0.92	50.26	0.92	50.23	1.49	50.02	1.51	49.93
**10**	-	36.85	-	36.83	-	38.61	-	38.28
**11**	1.45, 1.50	21.04	1.49, 1.49	21.04	1.57, 1.57	21.17	1.58, 1.58	21.19
**12**	1.18, 1.99	39.68	1.18, 1.99	39.66	1.15, 2.00	38.60	1.15, 2.01	38.57
**13**	-	42.20	-	42.21	-	42.97	-	42.97
**14**	1.01	56.88	1.01	56.86	1.34	50.04	1.35	50.02
**15**	1.04, 1.55	24.35	1.03, 1.55	24.35	1.24, 2.38	26.40	1.24, 2.38	26.39
**16**	1.70, 1.25	28.91	1.70, 1.26	28.91	1.77, 1.28	29.04	1.77, 1.28	29.03
**17**	1.15	55.95	1.15	55.95	1.14	54.70	1.14	54.68
**18**	0.69	12.03	0.70	12.03	0.70	12.18	0.70	12.18
**19**	1.00	19.37	1.00	19.37	1.18	17.26	1.20	17.30
**20**	2.04	40.47	2.04	40.47	2.05	40.23	2.05	40.23
**21**	1.02	21.20	1.02	21.21	1.03	21.41	1.03	21.40
**22**	5.15	138.31	5.15	138.30	5.17	138.09	5.17	138.07
**23**	5.02	129.24	5.02	129.27	5.03	129.46	5.03	129.47
**24**	1.53	51.22	1.53	51.23[a]	1.53	51.21[a]	1.53	51.20
**25**	1.53	31.86	1.53	31.87	1.53	31.87	1.53	31.87
**26**	0.80	18.96	0.79	18.99[b]	0.79	18.97[b]	0.80	18.99
**27**	0.85	21.07	0.85	21.08[c]	0.85	21.04[c]	0.85	21.04
**28**	1.42, 1.16	25.39	1.42, 1.17	25.40[d]	1.42, 1.17	25.35[d]	1.42, 1.17	25.35
**29**	0.80	12.23	0.80	12.24	0.80	12.24	0.80	12.23

[a, b, c, d] Groups of resonances ambiguously assigned (could be interchanged within the group)

Table S10. ^{1}H and ^{13}C NMR chemical shifts of synthesized **3**, **6** and **30** steroids

Atom No.	3β,3’β-di(cholest-5-en-7-one-3β-yl) ether (di(7-ketocholesteryl) ether) (**7**)		3β,3’β-di(sitost-5-en-7-one-3β-yl) ether (di(7-ketositosteryl) ether) (**8**)		3β,3’β-di(stigmast-5-en-7-one-3β-yl) ether (di(7-ketostigmasteryl) ether) (**9**)	
	**^{1}H NMR δ (ppm)**	**^{13}C δ (ppm)**	**^{1}H NMR δ (ppm)**	**^{13}C δ (ppm)**	**^{1}H NMR δ (ppm)**	**^{13}C δ (ppm)**
**1**	1.04, 1.86	37.31	1.16, 1.95	36.33	1.16, 1.95	36.34
**2**	1.82, 1.50	29.30	1.93, 1.57	28.94	1.93, 1.59	28.94
**3**	3.29	76.87	3.41	75.62	3.41	75.62
**4**	2.27, 2.23	39.98	2.47, 2.38	39.88	2.48, 2.38	39.89
**5**	-	140.96	-	165.16	-	165.13
**6**	5.34	121.66	5.67	126.07	5.67	126.06
**7**	1.51, 1.97	31.94	-	202.24	-	202.14
**8**	1.45	31.89	2.23	45.39	2.23	45.37
**9**	0.92	50.22	1.48	49.97	1.49	49.99
**10**	-	36.83	-	38.54	-	38.55
**11**	1.45, 1.49	21.06	1.56, 1.56	21.19	1.57, 1.57	21.18
**12**	1.15, 2.01	39.77	1.12, 2.03	38.69	1.14, 2.00	38.58
**13**	-	42.32	-	43.08	-	42.97
**14**	0.99	56.77	1.33	49.95	1.34	50.02
**15**	1.06, 1.57	24.29	1.24, 2.40	26.31	1.23, 2.37	26.39
**16**	1.82, 1.26	28.23	1.90, 1.27	28.54	1.77, 1.27	29.03
**17**	1.09	56.15	1.09	54.68	1.14	54.69
**18**	0.676	11.85	0.68	11.96	0.69	12.18
**19**	1.00	19.37	1.18	17.26	1.19	17.27
**20**	1.37	35.78	1.35	36.06	2.05	40.23
**21**	0.91	18.72	0.92	18.91	1.02	21.40
**22**	1.34, 1.00	36.18	1.33, 1.02	33.93	5.17	138.07
**23**	1.34, 1.14	23.82	1.15	26.06	5.03	129.48
**24**	1.12, 1.12	39.52	0.92	45.81	1.53	51.20
**25**	1.52	28.01[a]	1.66	29.11	1.53	31.87
**26**	0.86	22.56[b]	0.81	19.03	0.79	18.99
**27**	0.87	22.81[c]	0.83	19.78	0.84	21.04
**28**	-	-	1.24	23.04	1.41, 1.15	25.35
**29**	-	-	0.84	11.96	0.80	12.23

[a, b, c] Groups of resonances ambiguously assigned (could be interchanged within the group)

Table S11. ^{1}H and ^{13}C NMR chemical shifts of synthesized **7**, **8** and **9** steroids

Atom No.	Cholest-5-en-3β,7α-diol-cholesterol ether **(7α-hydroxycholesteryl-cholesterol ether)** (**10**)				Sitost-5-en-3β,7α-diol-sitosterol ether **(7α-hydroxysitosteryl-sitosterol ether)** (**11**)				Stigmast-5-en-3β,7α-diol-stigmasterol ether **(7α-hydroxystigmasteryl-stigmasterol ether)** (**12**)			
	sterol part		*7-hydroxysterol part*		*sterol part*		*7-hydroxysterol part*		*sterol part*		*7-hydroxysterol part*	
	**^{1}H NMR δ (ppm)**	**^{13}C δ (ppm)**	**^{1}H NMR δ (ppm)**	**^{13}C δ (ppm)**	**^{1}H NMR δ (ppm)**	**^{13}C δ (ppm)**	**^{1}H NMR δ (ppm)**	**^{13}C δ (ppm)**	**^{1}H NMR δ (ppm)**	**^{13}C δ (ppm)**	**^{1}H NMR δ (ppm)**	**^{13}C δ (ppm)**
**1**	1.04, 1.86	37.31	1.16, 1.94	36.47	1.03, 1.84	37.39	1.07, 1.85	37.20	1.04, 1.85	37.39	1.08, 1.86	37.19
**2**	1.82, 1.50	29.30	1.93, 1.59	29.14	1.81, 1.48	29.38	1.84, 1.50	29.20	1.83, 1.49	29.39	1.85, 1.51	29.21
**3**	3.29	76.87	3.41	75.05	3.27	76.51	3.34	75.85	3.27	76.50	3.34	75.84
**4**	2.27, 2.23	39.98	2.49, 2.37	39.90	2.28, 2.21	39.99	2.33, 2.26	39.90	2.27, 2.21	39.99	2.33, 2.25	39.90
**5**	-	140.96	-	165.63	-	141.17	-	146.80	-	141.15	-	146.75
**6**	5.34	121.66	5.67	125.93	5.33	121.46	5.58	123.52	5.33	121.45	5.58	123.51
**7**	1.51, 1.97	31.94	-	202.33	1.52, 1.97	31.96	3.85	65.40	1.50, 1.97	31.94	3.84	65.35
**8**	1.45	31.89	2.23	45.39	1.44	31.90	1.45	37.51	1.45	31.89	1.46	37.50
**9**	0.92	50.22	1.48	50.01	0.91	50.23	1.21	42.32	0.92	50.25	1.22	42.34
**10**	-	36.83	-	38.59	-	36.84	-	37.73	-	36.85	-	37.73
**11**	1.45, 1.49	21.06	1.57, 1.57	21.19	1.45, 1.49	21.06	1.48, 1.52	20.69	1.45, 1.50	21.04	1.48, 1.53	20.68
**12**	1.15, 2.01	39.77	1.12, 2.03	38.73	1.15, 2.00	39.78	1.16, 1.99	39.19	1.17, 1.98	39.69	1.19, 1.97	39.10
**13**	-	42.32	-	43.09	-	42.32	-	42.14	-	42.21	-	42.04
**14**	0.99	56.77	1.33	49.97	0.98	56.78	1.43	49.45	1.01	56.88	1.44	49.51
**15**	1.06, 1.57	24.29	1.24, 2.40	26.31	1.06, 1.57	24.29	1.13, 1.70	24.29	1.03, 1.55	24.35	1.12, 1.67	24.36
**16**	1.82, 1.26	28.23	1.89, 1.27	28.55	1.83, 1.26	28.24	1.90, 1.30	28.28	1.70, 1.24	28.92	1.77, 1.27	28.94
**17**	1.09	56.15	1.08	54.80	1.10	56.05	1.17	55.71	1.14	55.96	1.21	55.77
**18**	0.676	11.85	0.679	11.96	0.673	11.85	0.679	11.62	0.69	12.04	0.70	11.81
**19**	1.00	19.37	1.18	17.27	0.996	19.37	0.982	18.22	1.00	19.38	0.98	18.22
**20**	1.37	35.78	1.37	35.70	1.35	36.15	1.35	36.13	2.04	40.48	2.04	40.44
**21**	0.91	18.72	0.92	18.87	0.917	18.77	0.929	18.80	1.02	21.21	1.03	21.21
**22**	1.34, 1.00	36.18	1.34, 1.00	36.18	1.31, 1.00	33.94	1.31, 1.00	33.92	5.15	138.32	5.16	138.23
**23**	1.34, 1.14	23.82	1.34, 1.14	23.82	1.15	26.08	1.15	26.08	5.01	129.25*	5.01	129.32*
**24**	1.12, 1.12	39.52	1.12, 1.12	39.47	0.92	45.82	0.92	45.82	1.52	51.23	1.52	51.23
**25**	1.52	28.01[a]	1.51	27.99[a]	1.66	29.14	1.66	29.14	1.52	31.87	1.52	31.87
**26**	0.86	22.56[b]	0.86	22.55[b]	0.81	19.03	0.81	19.03	0.79	18.98	0.79	18.98
**27**	0.87	22.81[c]	0.87	22.80[c]	0.83	19.81	0.83	19.81	0.84	21.08	0.84	21.08
**28**	-	-	-	-	1.24	23.05	1.24	23.05	1.41, 1.16	25.40	1.41, 1.16	25.40
**29**	-	-	-	-	0.84	11.97	0.84	11.97	0.80	12.25	0.80	12.25

[a, b, c] Groups of resonances ambiguously assigned (could be interchanged within the group)

Table S12. ^{1}H and ^{13}C NMR chemical shifts of synthesized **10**, **11** and **12** steroids

Atom No.	**Cholest-5-en-3β,7β-diol-cholesterol ether** (7β-hydroxycholesteryl-cholesterol ether) (**13**) *sterol part* ^{1}H NMR δ (ppm)	*sterol part* ^{13}C δ (ppm)	*7-hydroxysterol part* ^{1}H NMR δ (ppm)	*7-hydroxysterol part* ^{13}C δ (ppm)	**Sito-5-en-3β,7β-diol-sitosterol ether** (7β-hydroxysitosteryl-sitosterol ether) (**14**) *sterol part* ^{1}H NMR δ (ppm)	*sterol part* ^{13}C δ (ppm)	*7-hydroxysterol part* ^{1}H NMR δ (ppm)	*7-hydroxysterol part* ^{13}C δ (ppm)	**Stigma-5-en-3β,7β-diol-stigmasterol ether** (7β-hydroxystigmasteryl-stigmasterol ether) (**15**) *sterol part* ^{1}H NMR δ (ppm)	*sterol part* ^{13}C δ (ppm)	*7-hydroxysterol part* ^{1}H NMR δ (ppm)	*7-hydroxysterol part* ^{13}C δ (ppm)
**1**	1.03, 1.84	37.39	1.01, 1.84	37.09	1.03, 1.84	37.39	1.01, 1.84	37.09	1.04, 1.85	37.39	1.01, 1.84	37.09
**2**	1.82, 1.49	29.37	1.84, 1.50	29.37	1.83, 1.49	29.37	1.83, 1.49	29.37	1.83, 1.49	29.38	1.85, 1.51	29.38
**3**	3.27	76.49	3.29	75.97	3.27	76.50	3.29	75.98	3.27	76.49	3.29	75.98
**4**	2.27, 2.23	40.06	2.31, 2.23	39.58	2.27, 2.22	40.06	2.32, 2.23	39.58	2.27, 2.21	40.06	2.32, 2.23	39.57
**5**	-	141.26	-	144.02	-	141.26	-	144.01	-	141.25	-	143.97
**6**	5.33	121.41	5.26	125.13	5.33	121.41	5.27	125.13	5.33	121.39	5.26	125.11
**7**	1.52, 1.97	31.95	3.83	73.41	1.52, 1.97	31.96	3.83	73.42	1.50, 1.96	31.94	3.83	73.41
**8**	1.43	31.89	1.38	40.94	1.44	31.90	1.38	40.95	1.45	31.89	1.39	40.91
**9**	0.91	50.24	1.01	48.35	0.91	50.24	1.01	48.35	0.92	50.26	1.02	48.35
**10**	-	36.85	-	36.77	-	36.85	-	36.77	-	36.86	-	36.78
**11**	1.45, 1.49	21.05	1.45, 1.49	21.05	1.45, 1.49	21.05	1.45, 1.51	21.05	1.45, 1.50	21.04	1.46, 1.53	21.04
**12**	1.15, 2.01	39.79	1.13, 2.01	39.58	1.15, 2.00	39.78	1.13, 2.01	39.57	1.18, 1.98	39.68	1.16, 1.99	39.47
**13**	-	42.32	-	42.92	-	42.32	-	42.93	-	42.21	-	42.82
**14**	0.99	56.79	1.14	55.99	0.98	56.79	1.14	55.99	1.01	56.88	1.15	56.06
**15**	1.06, 1.57	24.28	1.42, 1.80	26.38	1.06, 1.57	24.29	1.42, 1.81	26.39	1.04, 1.54	24.35	1.42, 1.78	26.46
**16**	1.82, 1.25	28.22	1.87, 1.30	28.54	1.83, 1.25	28.24	1.89, 1.31	28.55	1.70, 1.24	28.91	1.77, 1.31	29.16
**17**	1.08	56.15	1.07	55.46	1.10	56.05	1.09	55.38	1.14	55.96	1.13	55.30
**18**	0.67	11.85	0.69	11.81	0.673	11.85	0.688	11.81	0.69	12.04	0.71	12.02
**19**	1.00	19.38	1.04	19.14	0.997	19.38	1.036	19.14	1.00	19.38	1.04	19.14
**20**	1.37	35.77	1.37	35.72	1.35	36.13	1.35	36.09	2.04	40.48	2.04	40.33
**21**	0.91	18.71	0.92	18.77	0.918	18.78	0.925	18.83	1.02	21.21	1.03	21.29
**22**	1.33, 0.99	36.19[a]	1.33, 0.99	36.20[a]	1.32, 1.01	33.94	1.32, 1.01	33.97	5.15	138.31	5.16	138.13
**23**	1.33, 1.13	23.82[b]	1.33, 1.13	23.81[b]	1.15	26.07	1.15	26.07	5.01	129.26	5.03	129.46
**24**	1.12, 1.12	39.50[c]	1.12, 1.12	39.51[c]	0.92	45.83	0.92	45.83	1.52	51.23	1.52	51.23
**25**	1.51	28.01	1.51	28.01	1.66	29.14	1.66	29.14	1.52	31.87	1.52	31.87
**26**	0.86	22.55	0.86	22.55	0.81	19.02	0.81	19.02	0.79	18.99[a]	0.79	18.97[a]
**27**	0.87	22.81	0.87	22.81	0.83	19.81	0.83	19.81	0.84	21.06[b]	0.84	21.08[b]
**28**	-	-	-	-	1.24	23.06	1.24	23.06	1.41, 1.16	25.40[c]	1.41, 1.16	25.37[c]
**29**	-	-	-	-	0.84	11.97	0.84	11.97	0.80	12.24	0.80	12.24

[a, b, c] Groups of resonances ambiguously assigned (could be interchanged within the group)

Table S13. ^{1}H and ^{13}C NMR chemical shifts of synthesized **13**, **14** and **15** steroids

Atom No.	(5,6α-epoxycholesteryl)-cholesterol ether (16)				(5,6α-epoxysitosteryl)-sitosterol ether (17)				(5,6α-epoxystigmasteryl)-stigmasterol ether (18)			
	sterol part		*5,6-epoxysterol part*		*sterol part*		*5,6-epoxysterol part*		*sterol part*		*5,6-epoxysterol part*	
	**^{1}H NMR δ (ppm)**	**^{13}C δ (ppm)**	**^{1}H NMR δ (ppm)**	**^{13}C δ (ppm)**	**^{1}H NMR δ (ppm)**	**^{13}C δ (ppm)**	**^{1}H NMR δ (ppm)**	**^{13}C δ (ppm)**	**^{1}H NMR δ (ppm)**	**^{13}C δ (ppm)**	**^{1}H NMR δ (ppm)**	**^{13}C δ (ppm)**
**1**	1.00, 1.82	37.36	1.32, 1.68	32.53	1.00, 1.82	37.36	1.32, 1.67	32.53	1.01, 1.82	37.36	1.33, 1.68	32.54
**2**	1.79, 1.45	29.35	1.91, 1.58	29.03	1.79, 1.44	29.34	1.91, 1.57	29.04	1.80, 1.45	29.35	1.91, 1.58	29.05
**3**	3.25	76.74	3.64	73.36	3.24	76.74	3.64	73.36	3.24	76.75	3.64	73.36
**4**	2.28, 2.20	40.07	2.04, 1.28	37.95	2.27, 2.20	40.07	2.04, 1.28	37.95	2.28, 2.20	40.07	2.04, 1.28	37.95
**5**	-	141.15	-	65.80	-	141.14	-	65.80	-	141.14	-	65.80
**6**	5.32	121.44	2.87	59.43	5.32	121.44	2.87	59.42	5.32	121.42	2.87	59.41
**7**	1.51, 1.96	31.94	1.47, 1.90	28.92	1.51, 1.96	31.94	1.47, 1.90	28.92	1.51, 1.96	31.93	1.47, 1.90	28.91
**8**	1.44	31.89	1.36	29.88	1.44	31.89	1.35	29.88	1.44	31.89	1.36	29.88
**9**	0.90	50.22	1.24	42.62	0.90	50.21	1.23	42.61	0.91	50.23	1.25	42.64
**10**	-	36.80	-	35.11	-	36.80	-	35.11	-	36.81	-	35.12
**11**	1.44, 1.48	21.05	1.24, 1.36	20.61	1.43, 1.47	21.05	1.24, 1.36	20.61	1.45, 1.49	21.04	1.25, 1.38	20.60
**12**	1.15, 2.00	39.78	1.12, 1.94	39.43	1.15, 2.00	39.77	1.10, 1.94	39.43	1.17, 1.98	39.68	1.13, 1.92	39.33
**13**	-	42.31	-	42.33	-	42.31	-	42.33	-	42.20	-	42.22
**14**	0.99	56.76	0.96	56.87	0.98	56.76	0.96	56.87	1.01	56.86	0.97	56.96
**15**	1.05, 1.57	24.28	0.97, 1.55	24.04	1.06, 1.57	24.29	0.97, 1.55	24.05	1.03, 1.55	24.35	0.98, 1.53	24.11
**16**	1.82, 1.24	28.22	1.81, 1.22	28.07	1.83, 1.24	28.24	1.82, 1.22	28.09	1.70, 1.25	28.91	1.68, 1.23	28.74
**17**	1.08	56.14	1.05	55.87	1.09	56.04	1.06	55.78	1.14	55.95	1.11	55.65
**18**	0.67	11.84	0.60	11.84	0.67	11.84	0.60	11.84	0.69	12.03	0.62	12.03
**19**	0.98	19.37	1.05	15.88	0.98	19.37	1.04	15.88	0.99	19.37	1.05	15.88
**20**	1.36	35.77	1.34	35.74	1.35	36.13	1.32	36.13	2.04	40.48	2.01	40.44
**21**	0.91	18.70	0.88	18.63	0.91	18.77	0.89	18.70	1.02	21.20	0.99	21.15
**22**	1.33, 0.98	36.18	1.33, 0.98	36.14	1.31, 1.00	33.94	1.31, 1.00	33.90	5.15	138.33	5.13	138.23
**23**	1.32, 1.13	23.82	1.32, 1.13	23.82	1.15	26.08	1.15	26.08	5.02	129.24	4.99	129.30
**24**	1.11, 1.11	39.50	1.11, 1.11	39.50	0.92	45.82	0.92	45.82	1.52	51.23	1.52	51.20
**25**	1.51	28.00	1.51	28.00	1.66	29.14	1.66	29.14	1.53	31.87	1.53	31.86
**26**	0.86	22.55	0.86	22.55	0.81	19.03	0.81	19.03	0.80	18.97	0.80	18.97
**27**	0.86	22.80	0.86	22.80	0.83	19.81	0.83	19.81	0.85	21.07	0.85	21.07
**28**					1.24	23.05	1.24	23.05	1.42, 1.16	25.40	1.42, 1.16	25.38
**29**					0.84	11.97	0.84	11.97	0.80	12.24	0.80	12.23

[a, b, c] Groups of resonances ambiguously assigned (could be interchanged within the group)

Table S14. ^{1}H and ^{13}C NMR chemical shifts of synthesized **16**, **17** and **18** steroids

Atom No.	(5,6β-epoxycholesteryl)-cholesterol ether (19) sterol part ^1H NMR δ (ppm)	sterol part ^{13}C δ (ppm)	5,6-epoxysterol part ^1H NMR δ (ppm)	5,6-epoxysterol part ^{13}C δ (ppm)	(5,6β-epoxysitosteryl)-sitosterol ether (20) sterol part ^1H NMR δ (ppm)	sterol part ^{13}C δ (ppm)	5,6-epoxysterol part ^1H NMR δ (ppm)	5,6-epoxysterol part ^{13}C δ (ppm)	(5,6β-epoxystigmasteryl)-stigmasterol ether (21) sterol part ^1H NMR δ (ppm)	sterol part ^{13}C δ (ppm)	5,6-epoxysterol part ^1H NMR δ (ppm)	5,6-epoxysterol part ^{13}C δ (ppm)
**1**	1.01, 1.83	37.37	1.22, 1.94	37.22	1.01, 1.83	37.38	1.21, 1.94	37.22	1.01, 1.83	37.38	1.22, 1.94	37.22
**2**	1.79, 1.47	29.33	1.78, 1.40	28.70	1.79, 1.47	29.34	1.77, 1.40	28.71	1.79, 1.47	29.34	1.78, 1.40	28.72
**3**	3.24	76.60	3.45	73.94	3.23	76.75	3.44	73.95	3.23	76.59	3.44	73.95
**4**	2.27, 2.23	39.99	2.03, 1.39	39.98	2.26, 2.21	40.00	2.03, 1.39	39.99	2.26, 2.21	40.00	2.03, 1.39	39.99
**5**	-	141.24	-	63.09	-	141.25	-	63.06	-	141.24	-	63.06
**6**	5.33	121.43	3.03	63.63	5.33	121.42	3.03	63.61	5.33	121.41	3.03	63.59
**7**	1.52, 1.97	31.95	1.18, 2.07	32.65	1.52, 1.97	31.95	1.18, 2.07	32.66	1.51, 1.97	31.94	1.18, 2.07	32.65
**8**	1.44	31.89	1.47	29.78	1.44	31.89	1.47	29.78	1.44	31.89	1.48	29.77
**9**	0.91	50.23	0.59	51.26	0.91	50.23	0.59	51.27	0.91	50.26	0.59	51.29
**10**	-	36.84	-	35.17	-	36.84	-	35.18	-	36.85	-	35.19
**11**	1.44, 1.48	21.04	1.37, 1.37	21.97	1.44, 1.48	21.05	1.40, 1.36	21.97	1.44, 1.49	21.03	1.38, 1.38	21.96
**12**	1.15, 2.00	39.77	1.05, 1.95	39.86	1.15, 2.00	39.77	1.05, 1.95	39.85	1.17, 1.98	39.68	1.07, 1.93	39.76
**13**	-	42.31	-	42.29	-	42.32	-	42.29	-	42.21	-	42.18
**14**	0.99	56.78	0.85	56.27	0.98	56.78	0.85	56.28	1.00	56.88	0.87	56.38
**15**	1.06, 1.57	24.28	1.05, 1.57	24.18	1.05, 1.56	24.29	1.05, 1.56	24.19	1.04, 1.55	24.35	1.04, 1.55	24.35
**16**	1.82, 1.24	28.22	1.80, 1.24	28.14	1.82, 1.25	28.24	1.82, 1.24	28.17	1.69, 1.25	28.91	1.68, 1.24	28.91
**17**	1.08	56.15	1.05	56.21	1.09	56.05	1.06	56.11	1.14	55.96	1.10	56.02
**18**	0.67	11.84	0.63	11.75	0.67	11.84	0.63	11.75	0.69	12.03	0.65	11.94
**19**	0.99	19.37	0.98	17.05	0.99	19.37	0.98	17.05	0.99	19.37	0.98	17.05
**20**	1.37	35.77	1.35	35.71	1.35	36.13	1.32	36.07	2.03	40.47	2.02	40.44
**21**	0.91	18.71	0.89	18.67	0.92	18.77	0.89	18.73	1.02	21.21	0.99	21.16
**22**	1.33, 0.98	36.18	1.33, 0.98	36.13	1.31, 1.00	33.94	1.31, 1.00	33.89	5.14	138.31	5.13	138.22
**23**	1.32, 1.13	23.81	1.32, 1.13	23.81	1.15	26.05	1.15	26.05	5.01	129.26	5.01	129.33
**24**	1.11, 1.11	39.50	1.11, 1.11	39.50	0.92	45.82	0.92	45.82	1.52	51.23	1.52	51.23
**25**	1.51	28.00	1.51	28.00	1.66	29.14	1.66	29.14	1.52	31.87	1.52	31.87
**26**	0.86	22.55	0.86	22.55	0.81	19.03	0.81	19.03	0.79	18.97	0.79	18.97
**27**	0.86	22.80	0.86	22.80	0.83	19.80	0.83	19.80	0.84	21.08	0.84	21.08
**28**					1.24	23.05	1.24	23.05	1.41 1.16	25.40	1.41, 1.16	25.40
**29**					0.84	11.97	0.84	11.97	0.80	12.24	0.80	12.24

[a, b, c] Groups of resonances ambiguously assigned (could be interchanged within the group)

Table S15. ^1H and ^{13}C NMR chemical shifts of synthesized **19**, **20** and **21** steroids

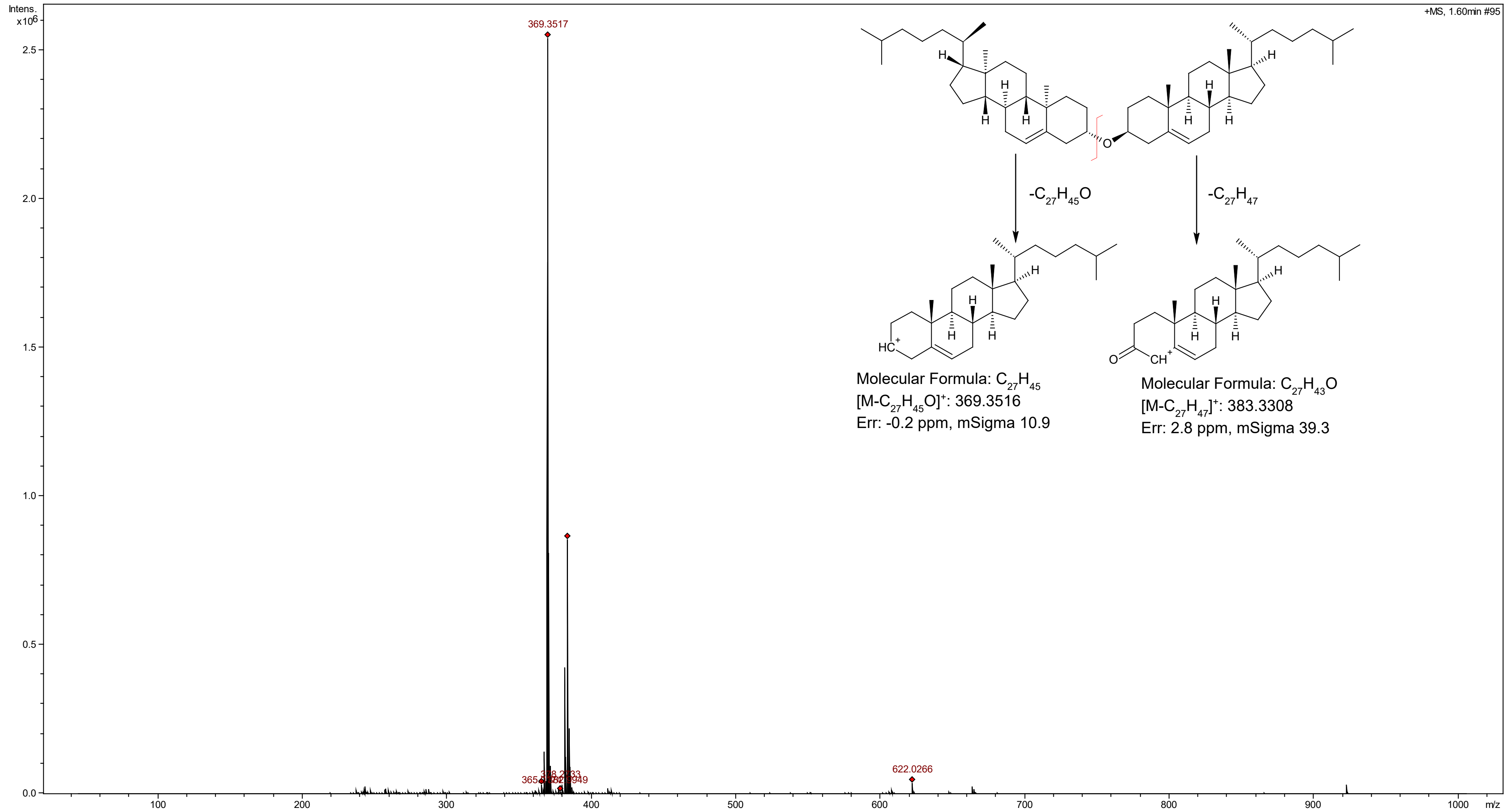

Figure S3. Mass spectrum of 3β,3'β-dicholesteryl ether (**1**)

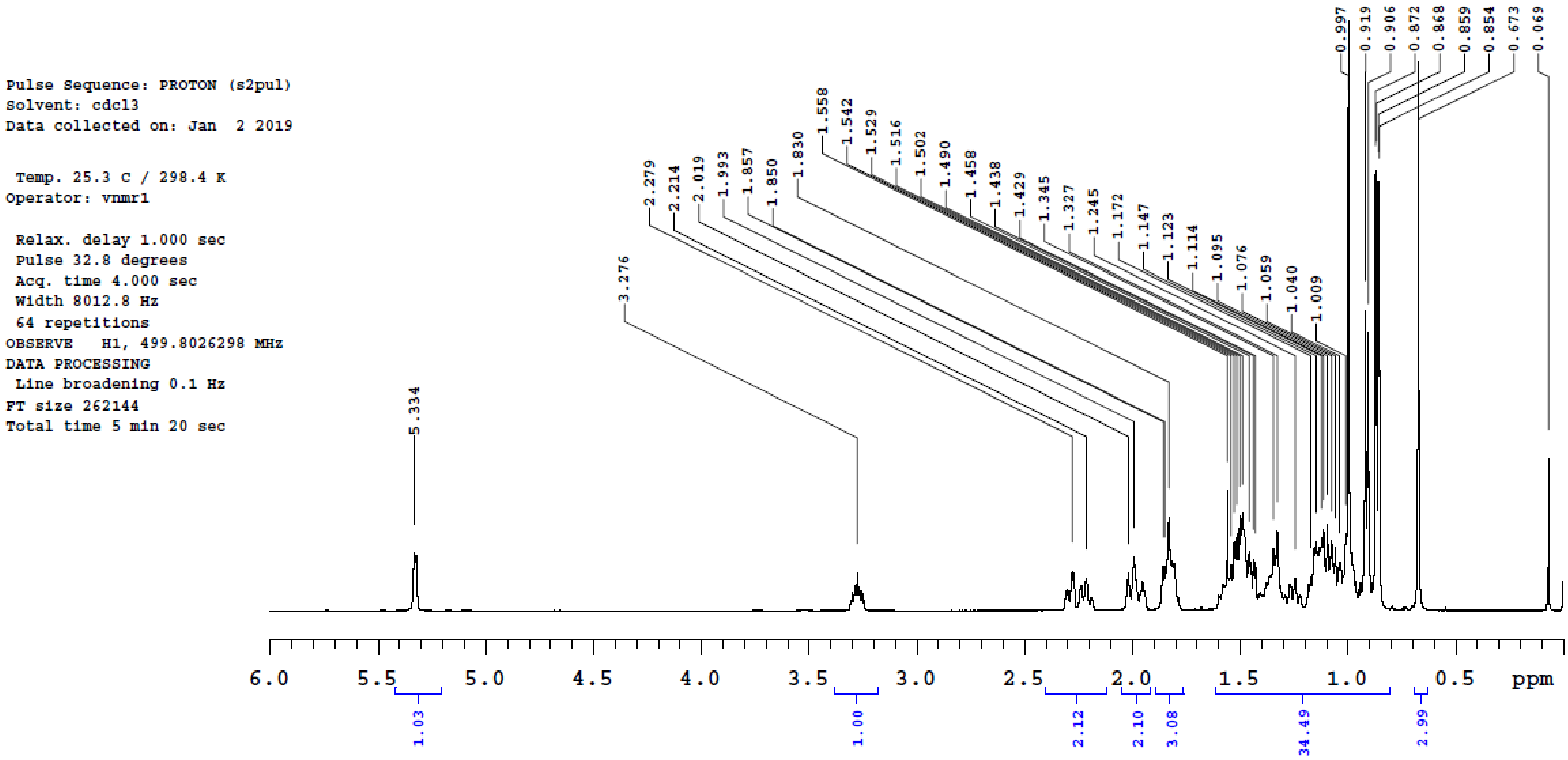

Figure S4. ^{1}H spectrum of 3β,3'β-dicholesteryl ether (**1**) in $CDCl_3$

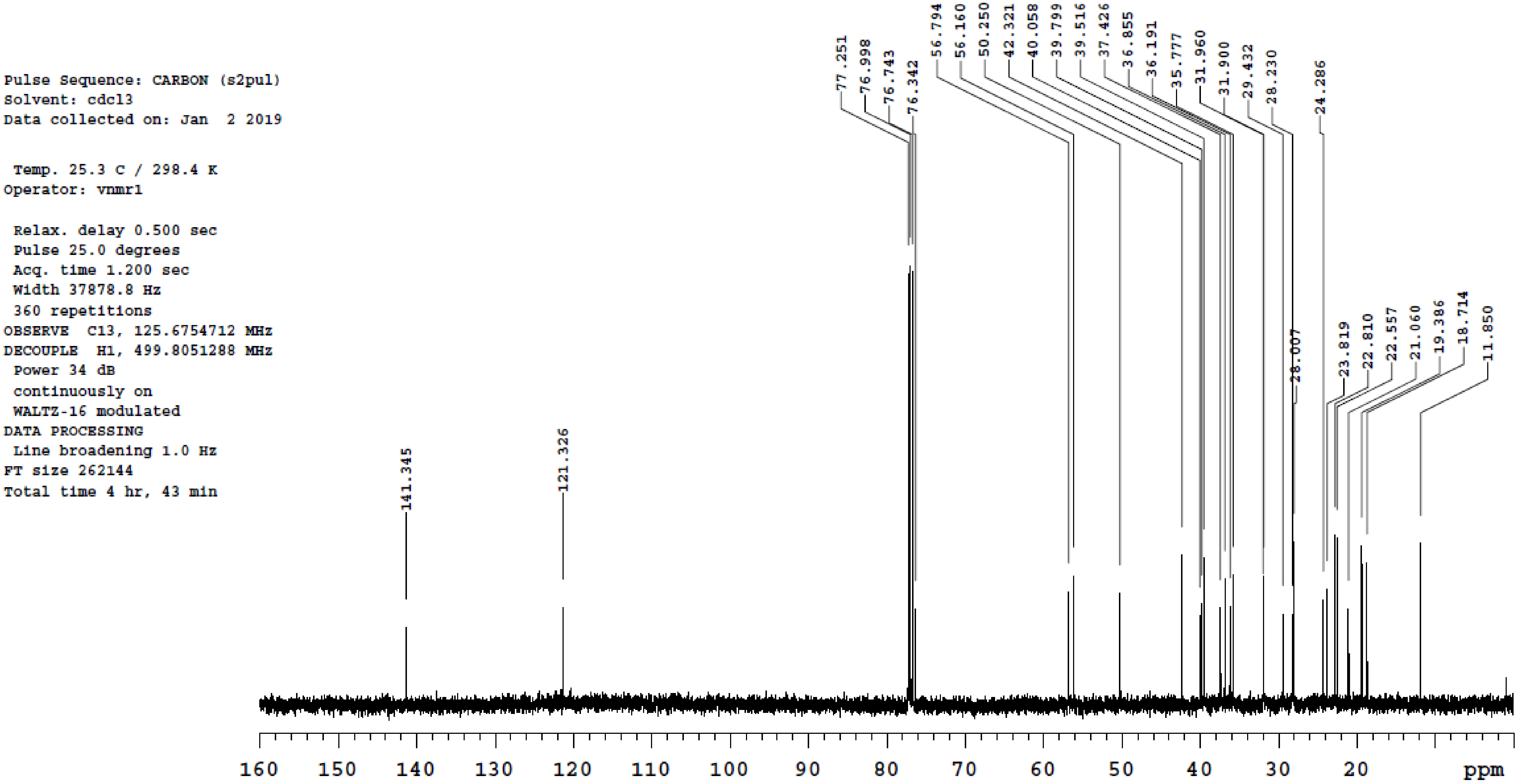

Figure S5. ^{13}C spectrum of 3β,3'β-dicholesteryl ether (**1**) in $CDCl_3$

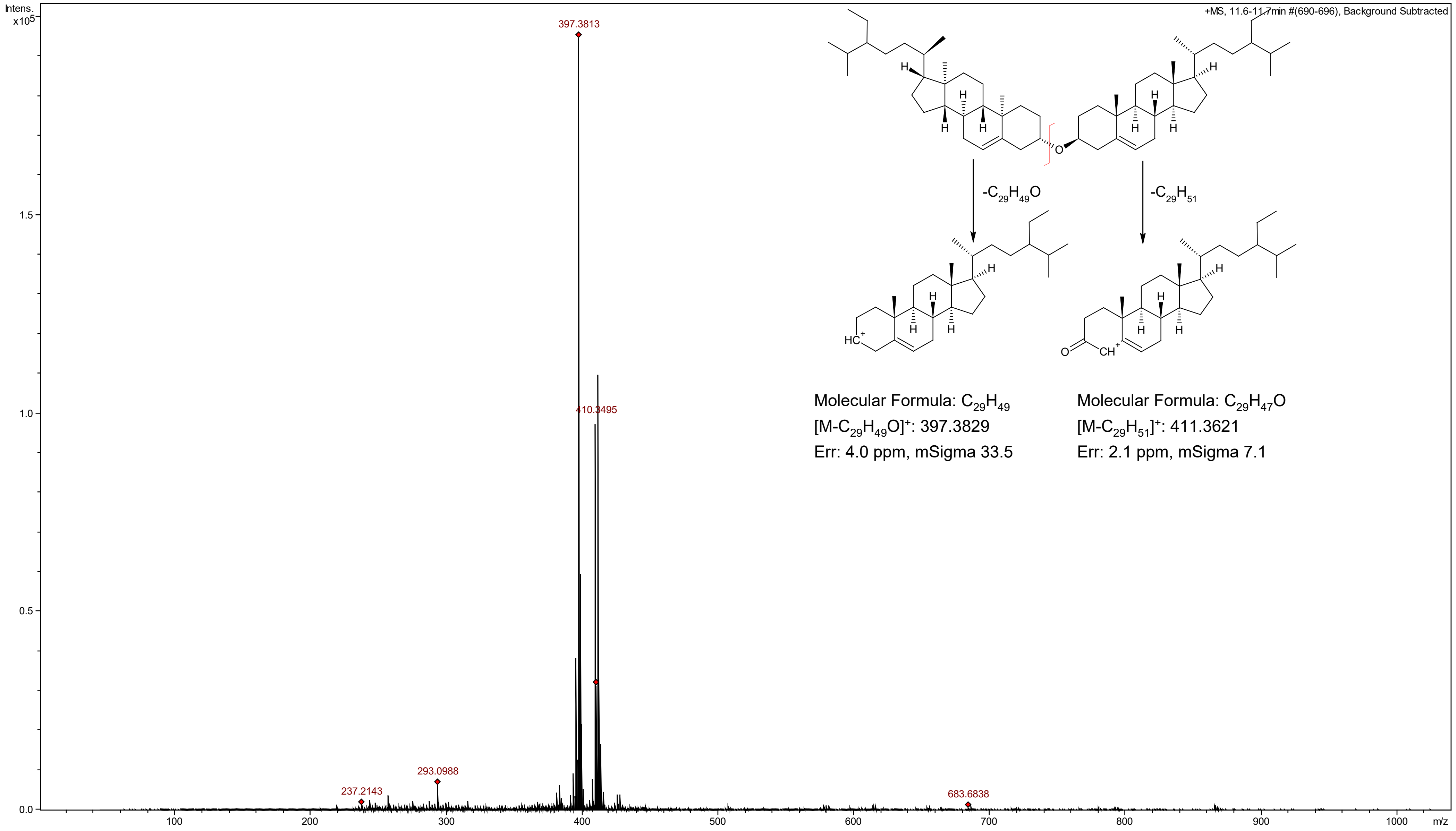

Figure S6. Mass spectrum of 3β,3’β-disitosteryl ether (**2**)

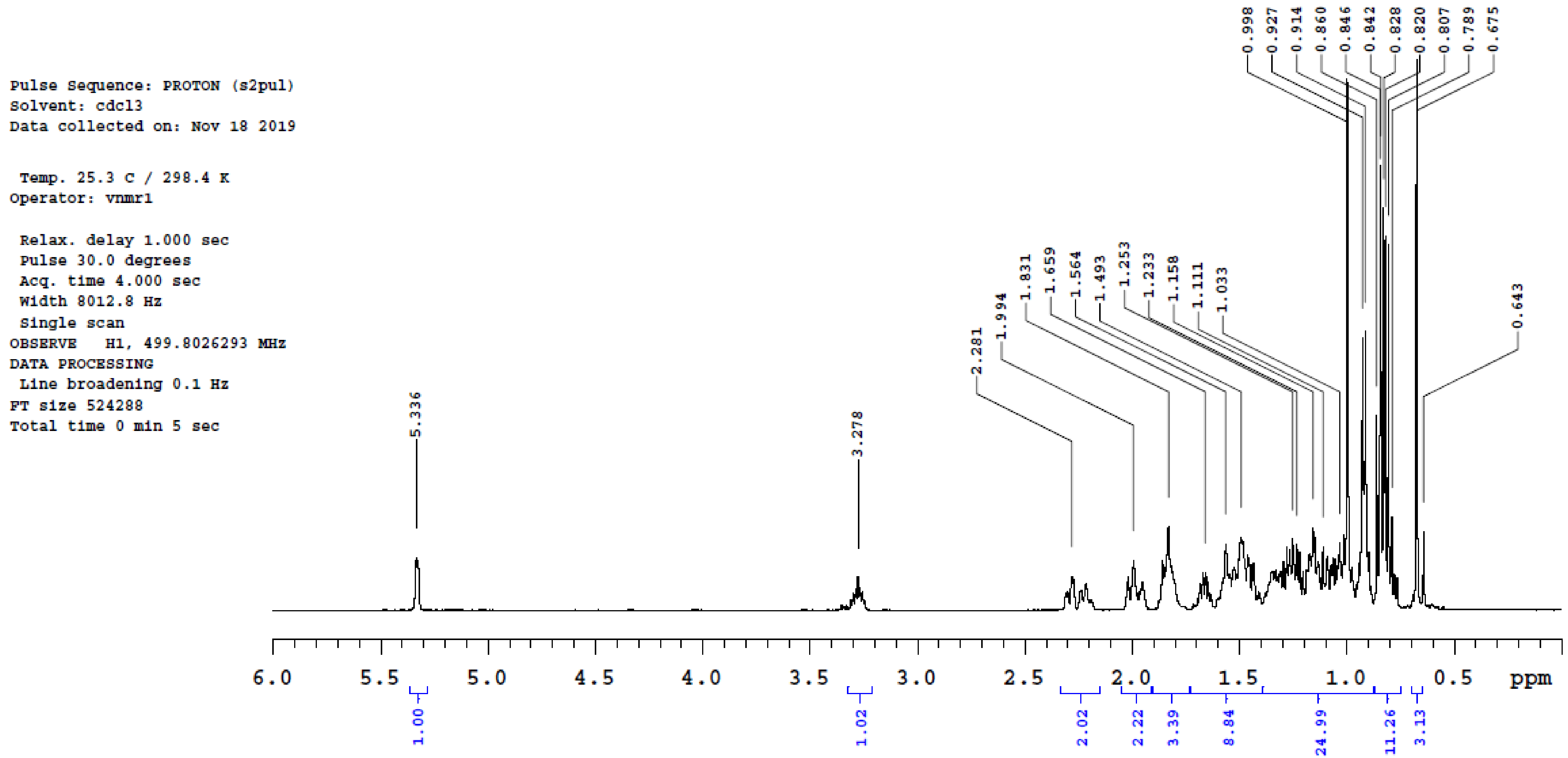

Figure S7. ^{1}H spectrum of 3β,3’β-disitosteryl ether (**2**) in $CDCl_3$

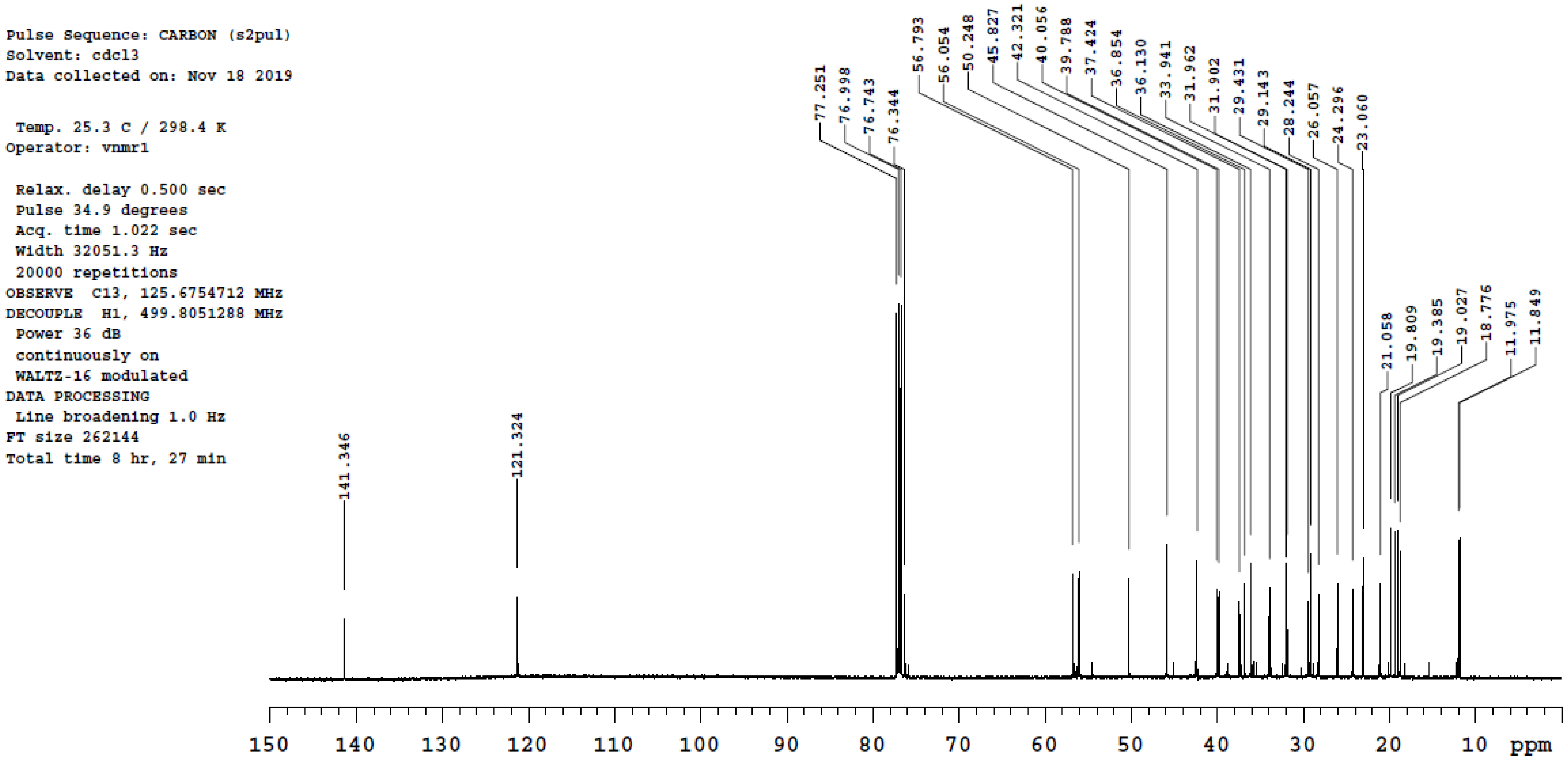

Figure S8. ^{13}C spectrum of 3β,3'β-disitosteryl ether (**2**) in $CDCl_3$

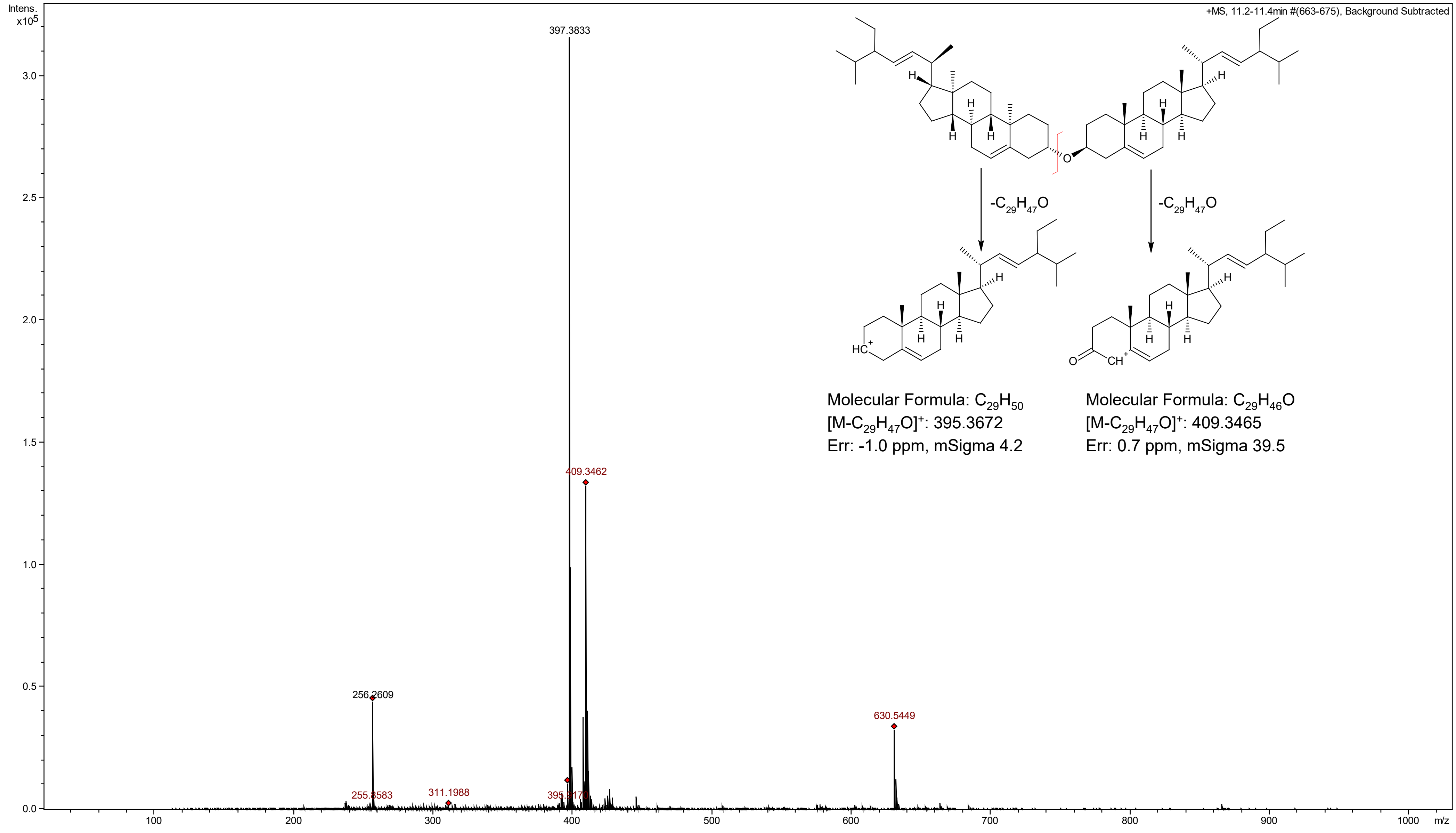

Figure S9. Mass spectrum of 3β,3'β-distigmasteryl ether (**3**)

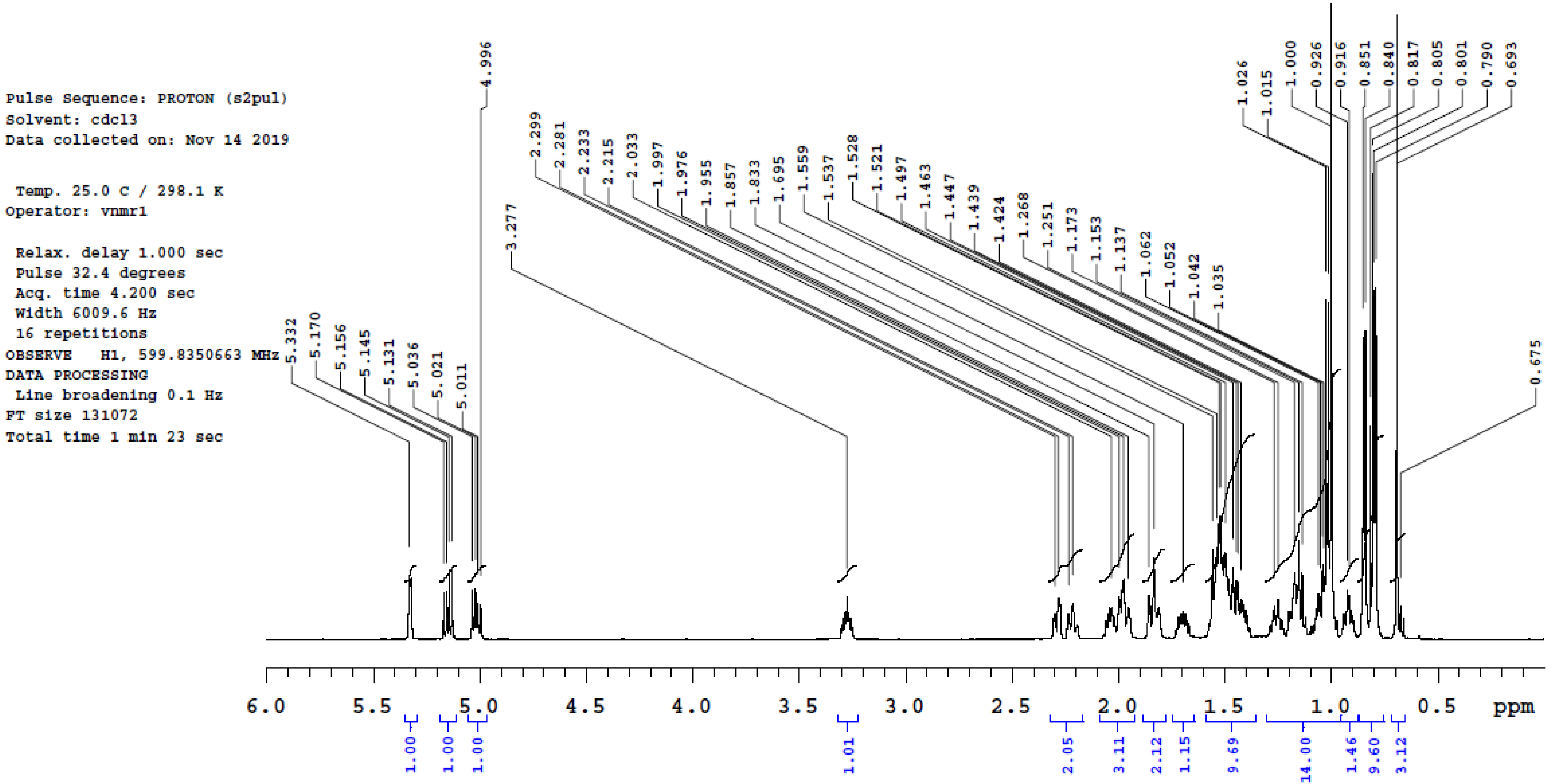

Figure S10. ^{1}H spectrum of 3β,3’β-distigmasteryl ether (**3**) in $CDCl_3$

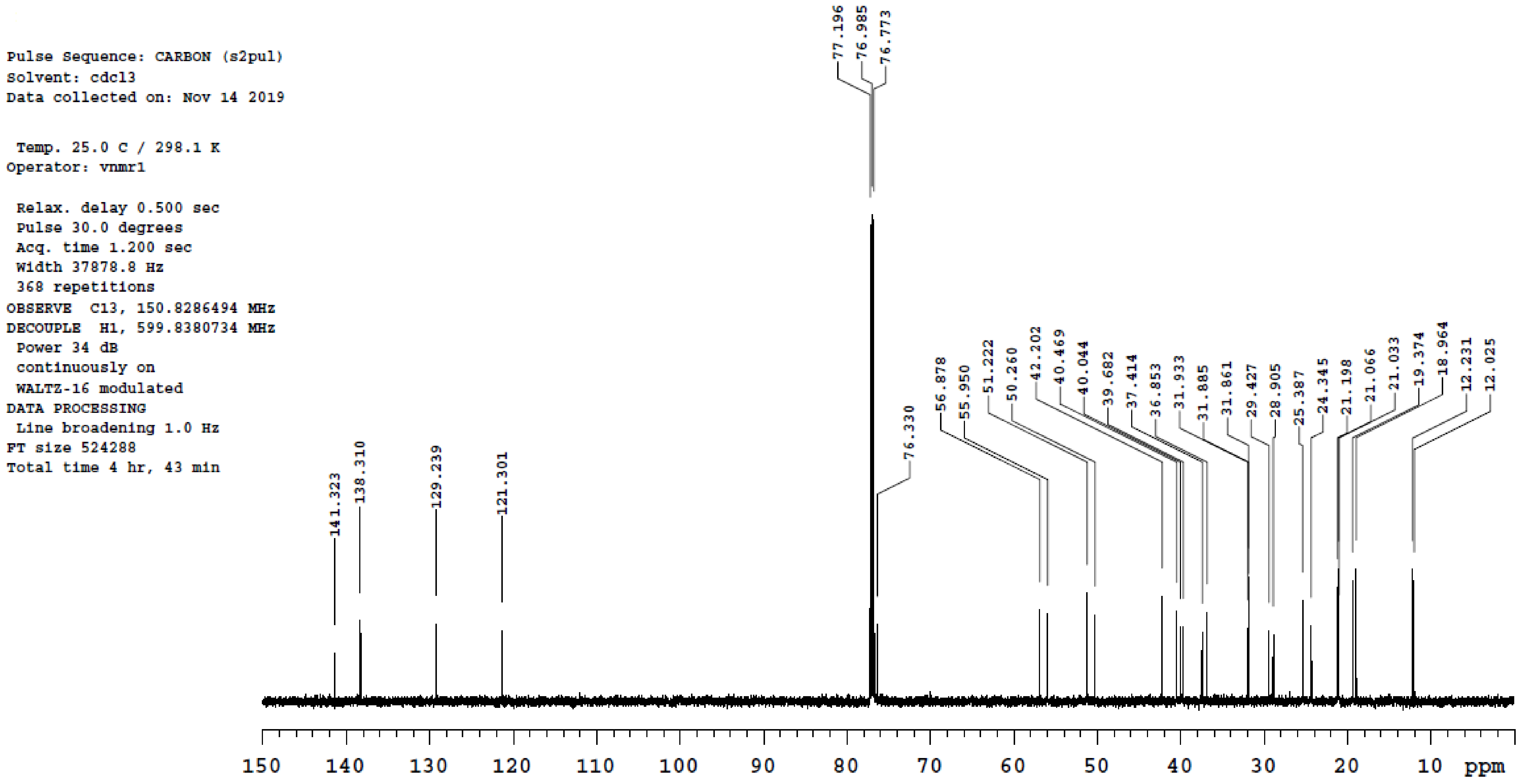

Figure S11. ^{13}C spectrum of 3β,3’β-distigmasteryl ether (**3**) in $CDCl_3$

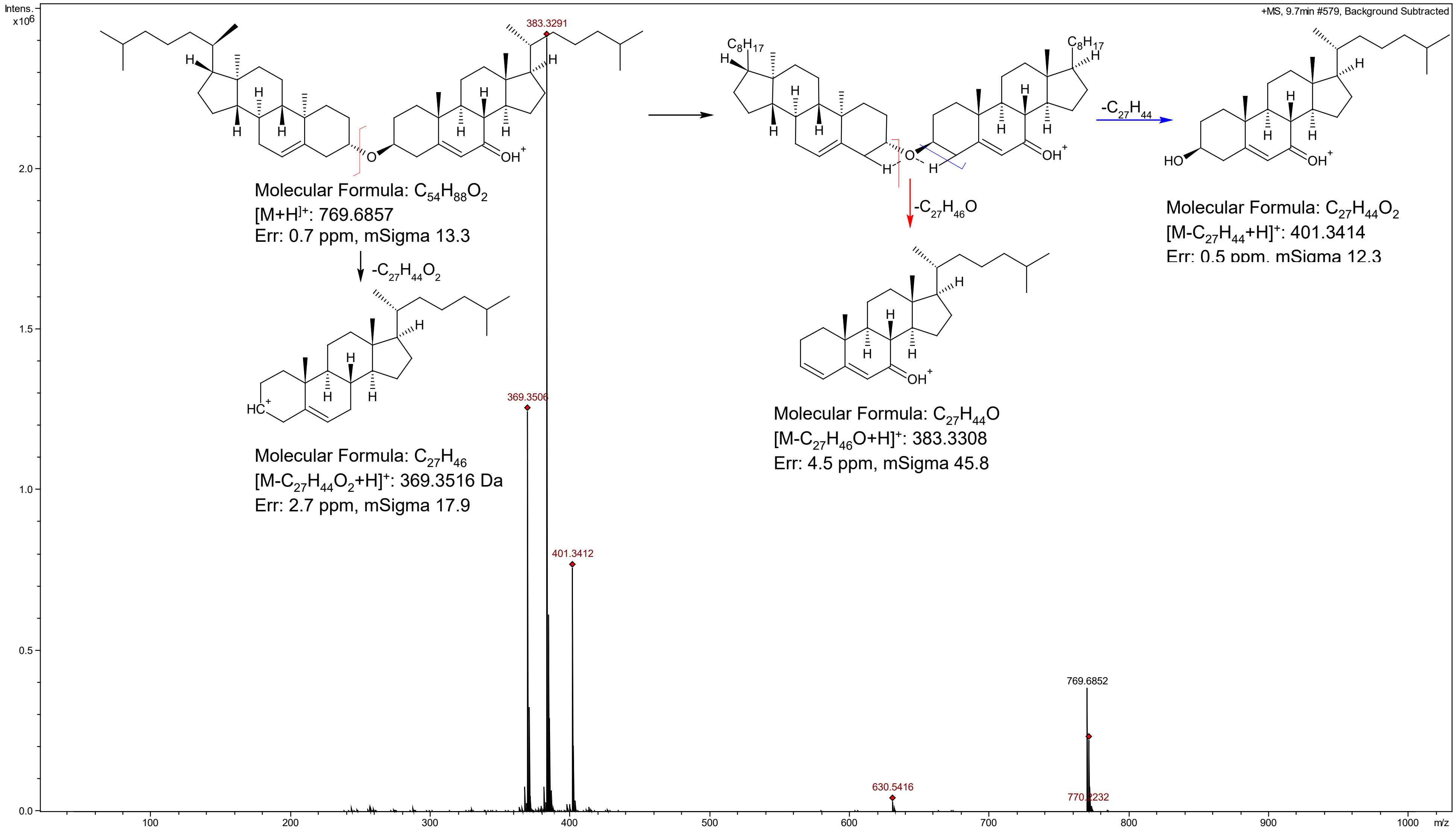

Figure S12. Mass spectrum of cholest-5-en-7-one-3β-yl-cholesterol ether (7-ketocholesteryl-cholesterol ether) (**4**)

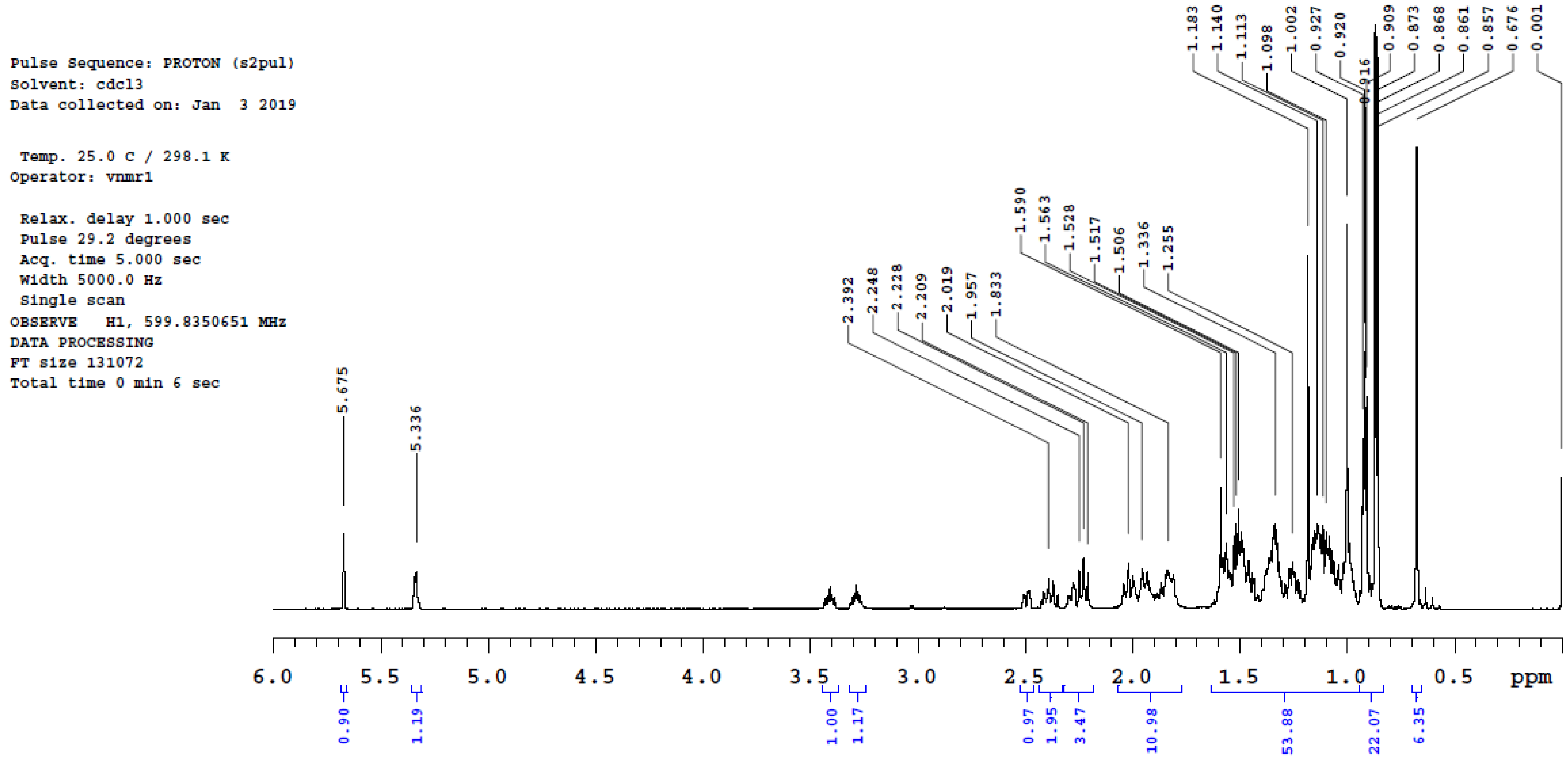

Figure S13. ^{1}H spectrum of cholest-5-en-7-one-3β-yl-cholesterol ether (7-ketocholesteryl-cholesterol ether) (**4**) in $CDCl_3$

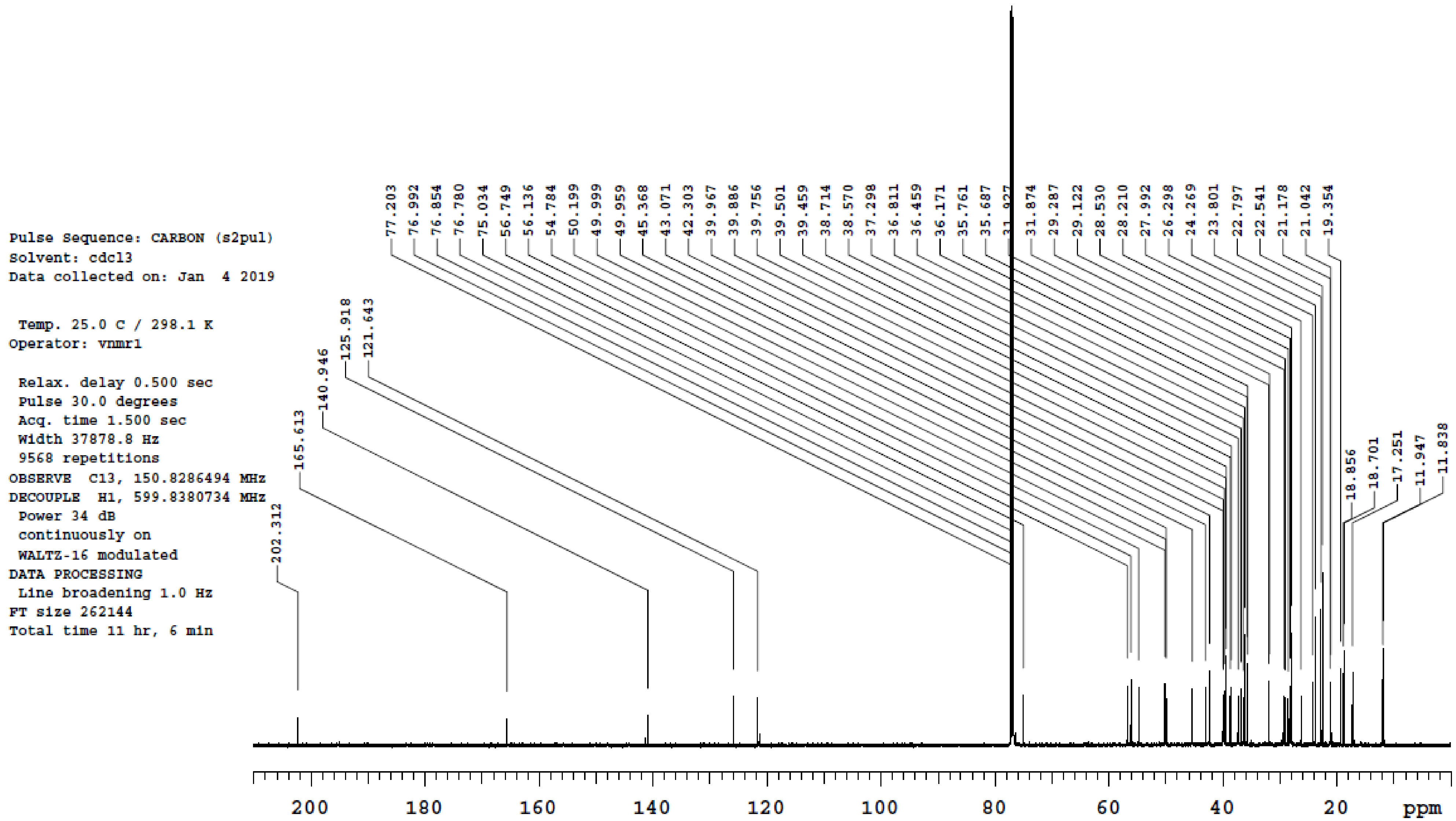

Figure S14. ^{13}C spectrum of cholest-5-en-7-one-3β-yl-cholesterol ether (7-ketocholesteryl-cholesterol ether) (**4**) in $CDCl_3$

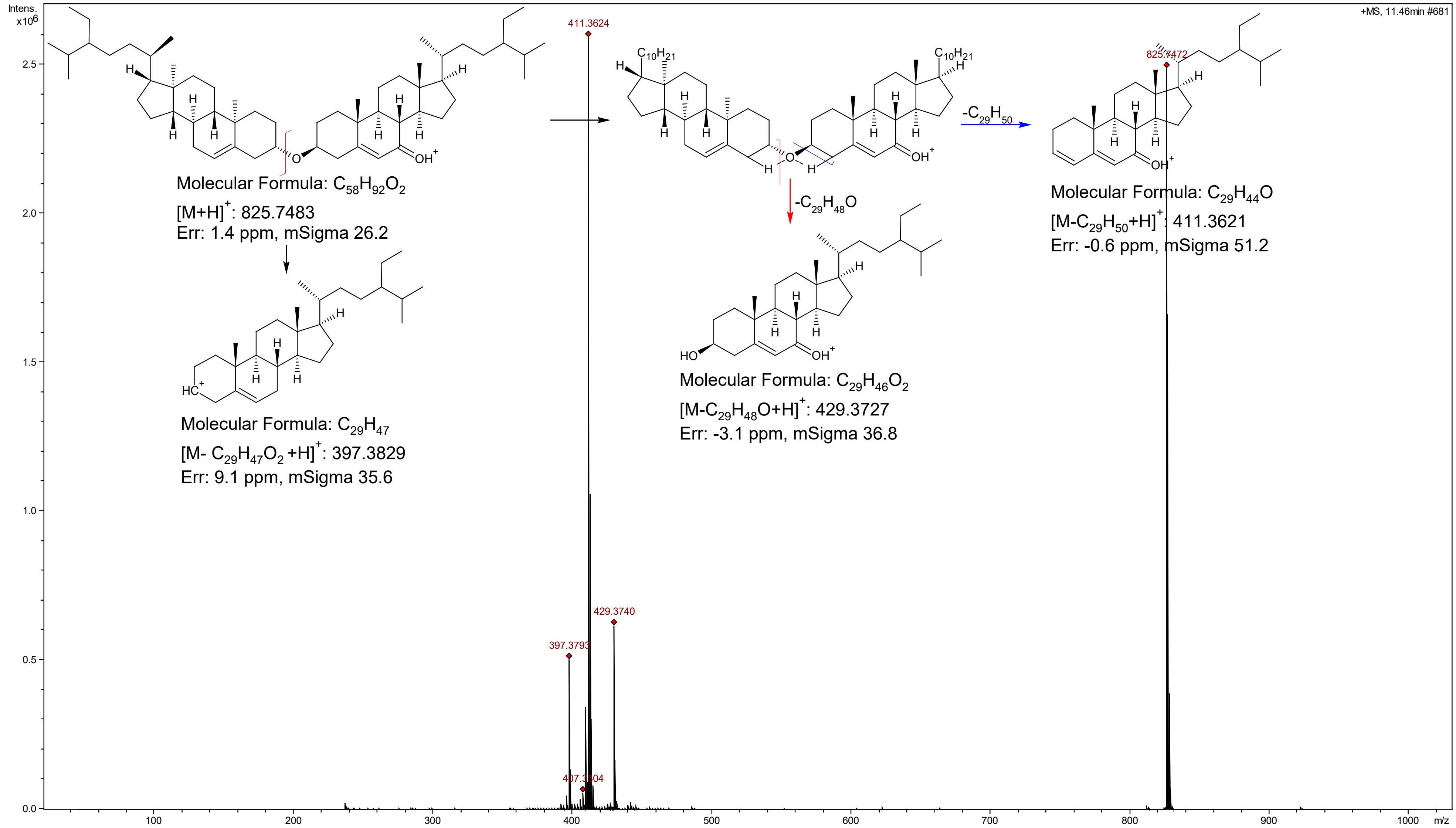

Figure S15. Mass spectrum of sitost-5-en-7-one-3β-yl-sitosterol ether (7-ketositosteryl-sitosterol ether) (**5**)

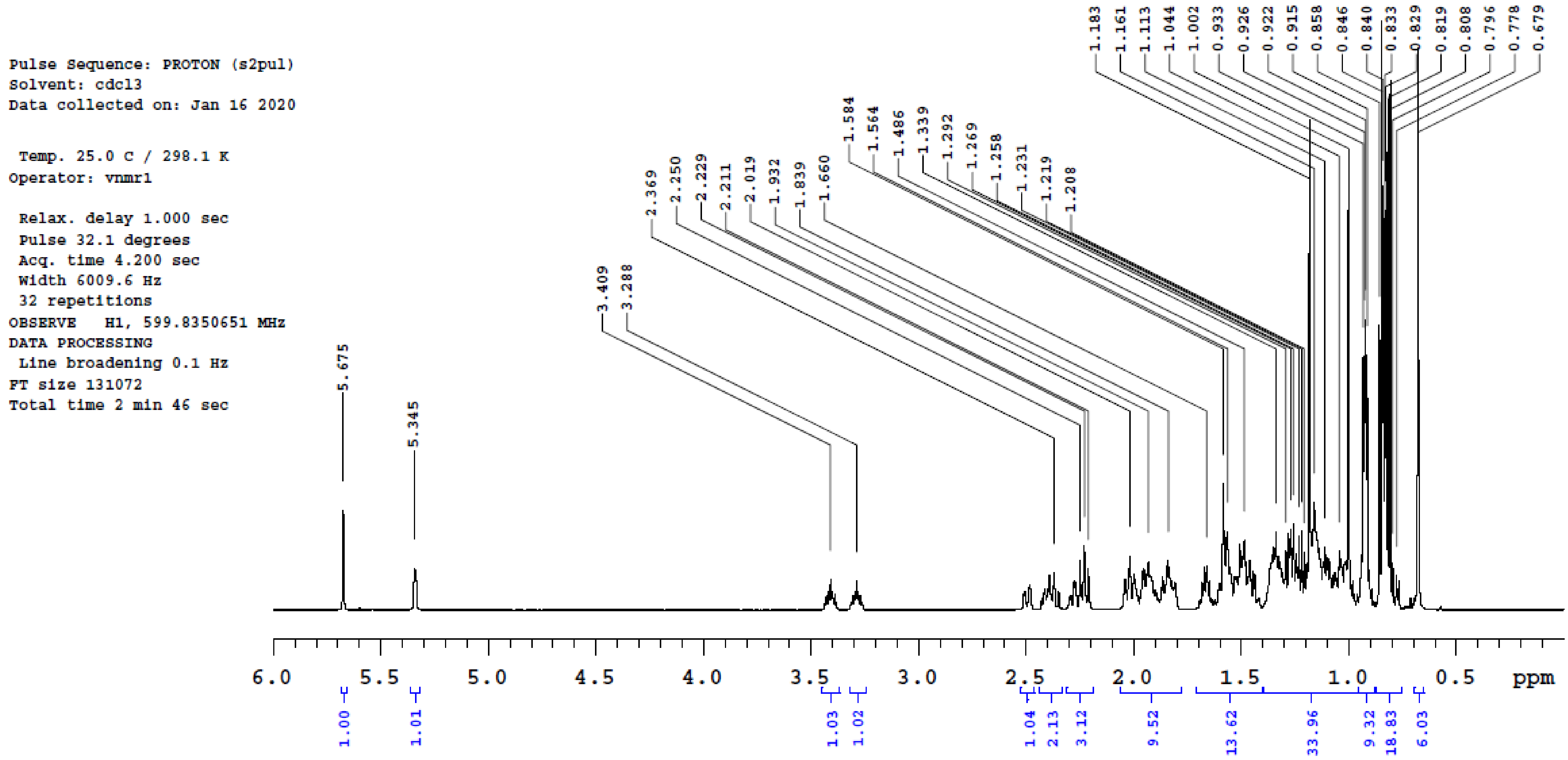

Figure S16. ^{1}H spectrum of sitost-5-en-7-one-3β-yl-sitosterol ether (7-ketositosteryl-sitosterol ether) (**5**) in $CDCl_3$

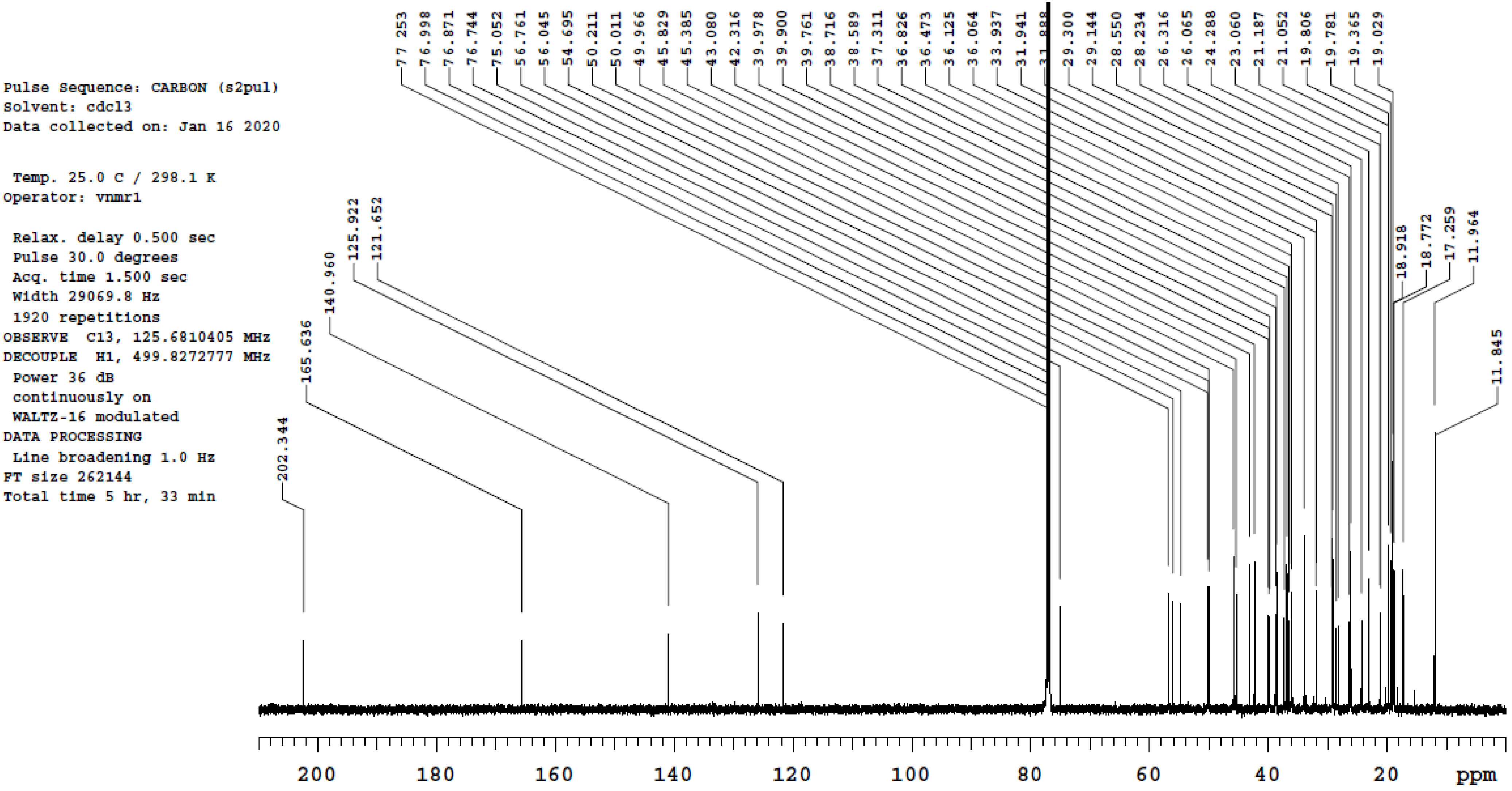

Figure S17. ^{13}C spectrum of sitost-5-en-7-one-3β-yl-sitosterol ether (7-ketositosteryl-sitosterol ether) (**5**) in $CDCl_3$

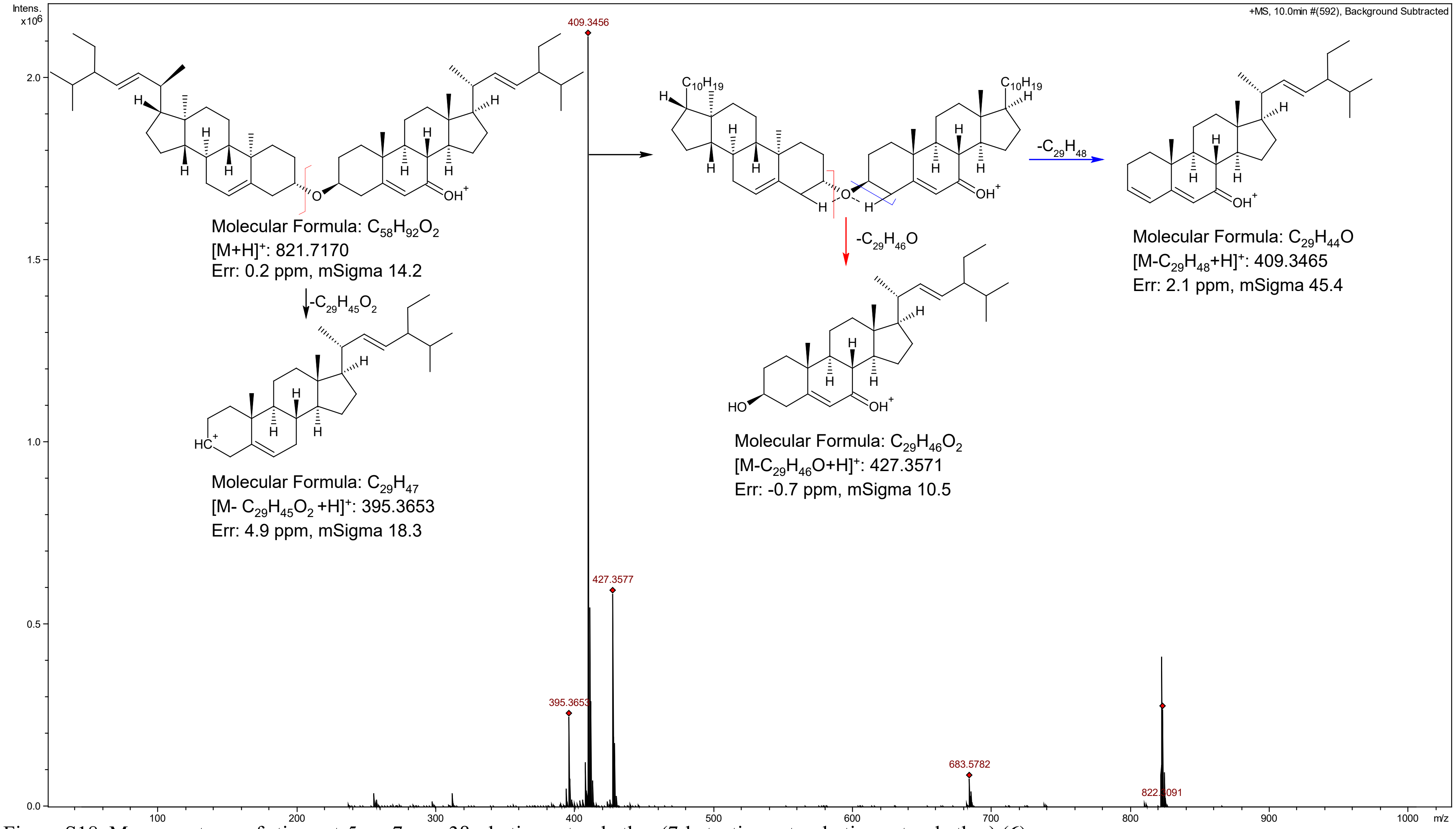

Figure S18. Mass spectrum of stigmast-5-en-7-one-3β-yl-stigmasterol ether (7-ketostigmasteryl-stigmasterol ether) (**6**)

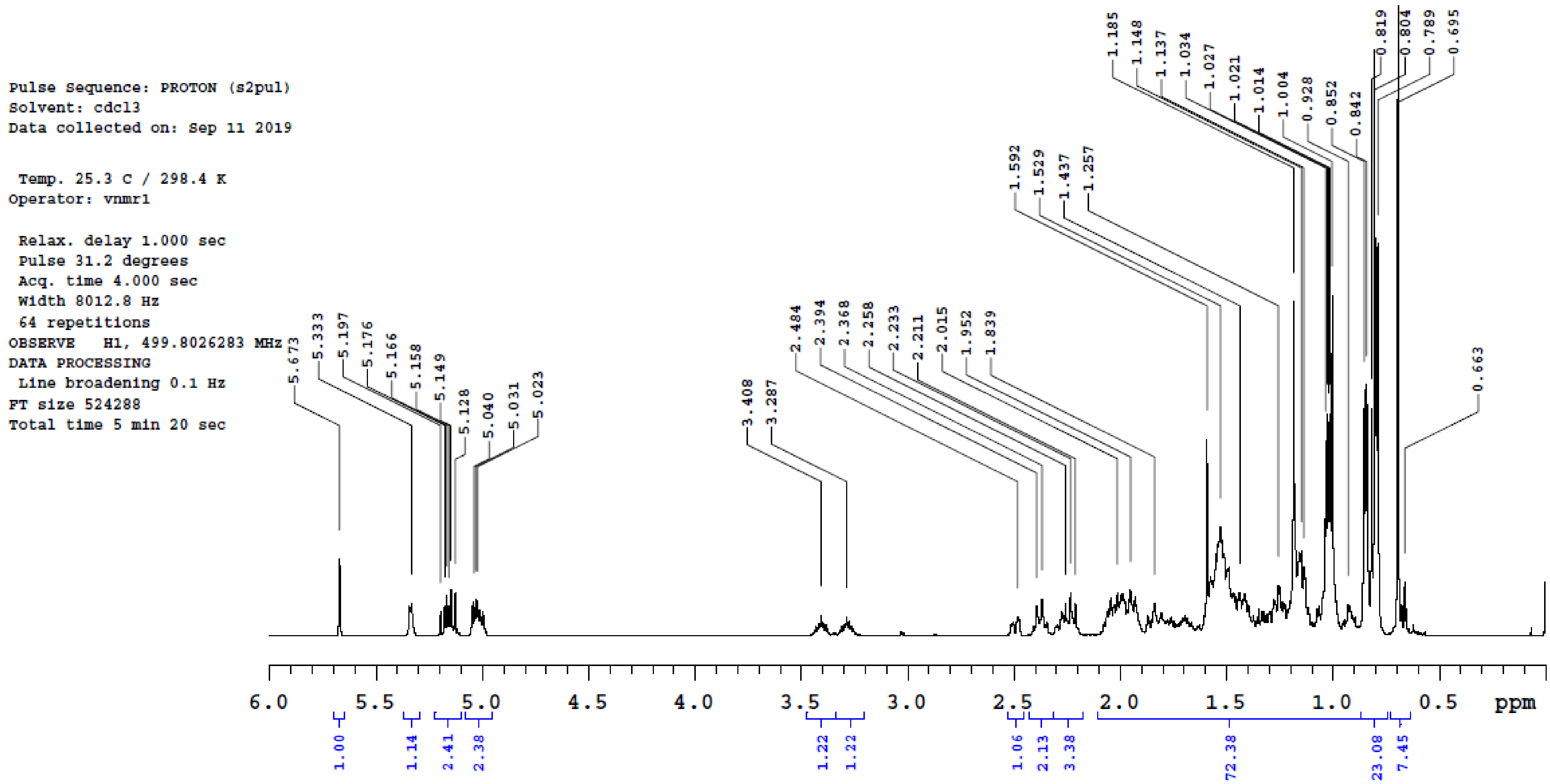

Figure S19. ^{1}H spectrum of stigmast-5-en-7-one-3β-yl-stigmasterol ether (7-ketostigmasteryl-stigmasterol ether) (**6**) in $CDCl_3$

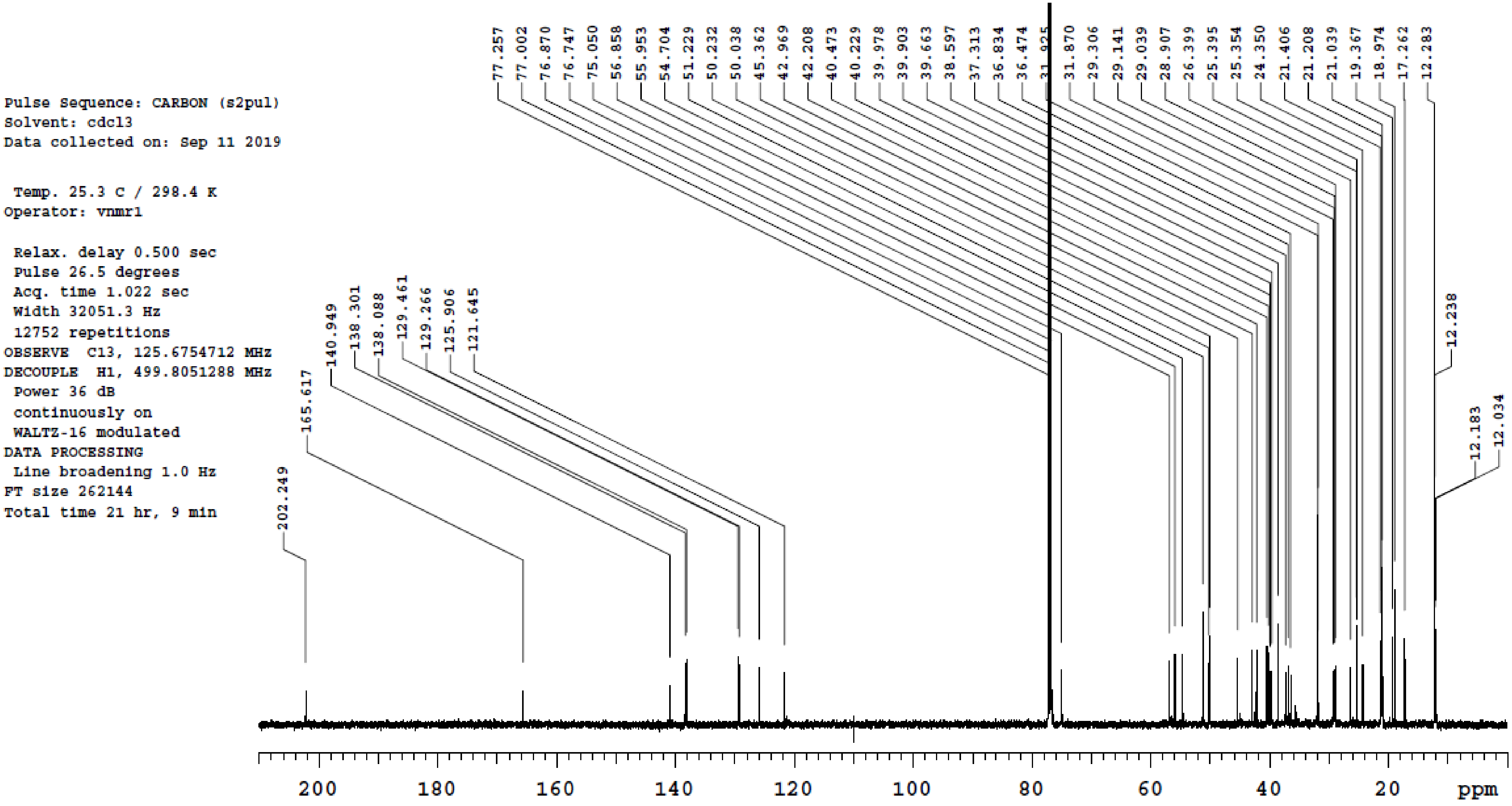

Figure S20. ^{13}C spectrum of stigmast-5-en-7-one-3β-yl-stigmasterol ether (7-ketostigmasteryl-stigmasterol ether) (**6**) in $CDCl_3$

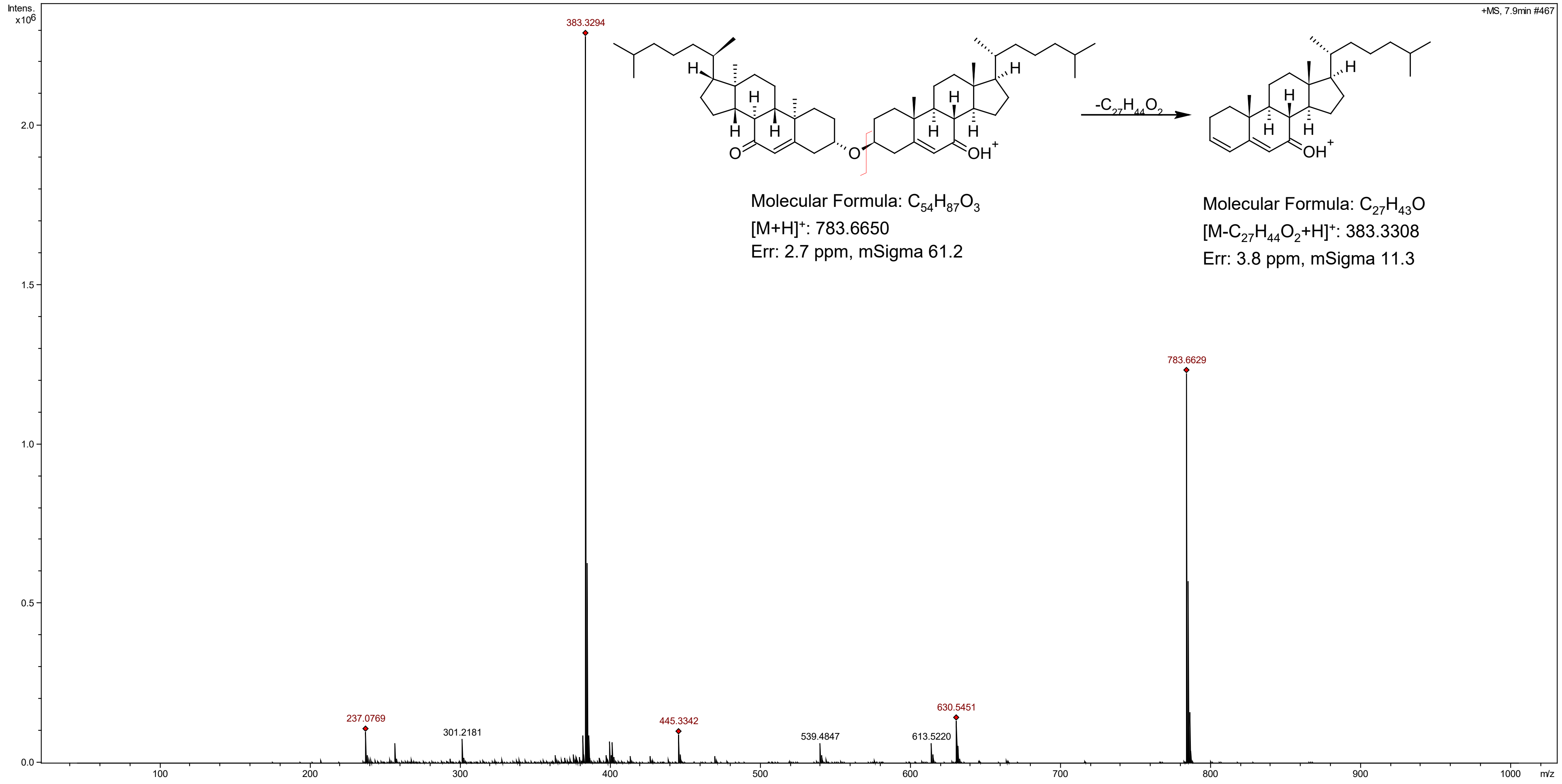

Figure S21. Mass spectrum of 3β,3'β-di(cholest-5-en-7-one-3β-yl) ether (**7**)

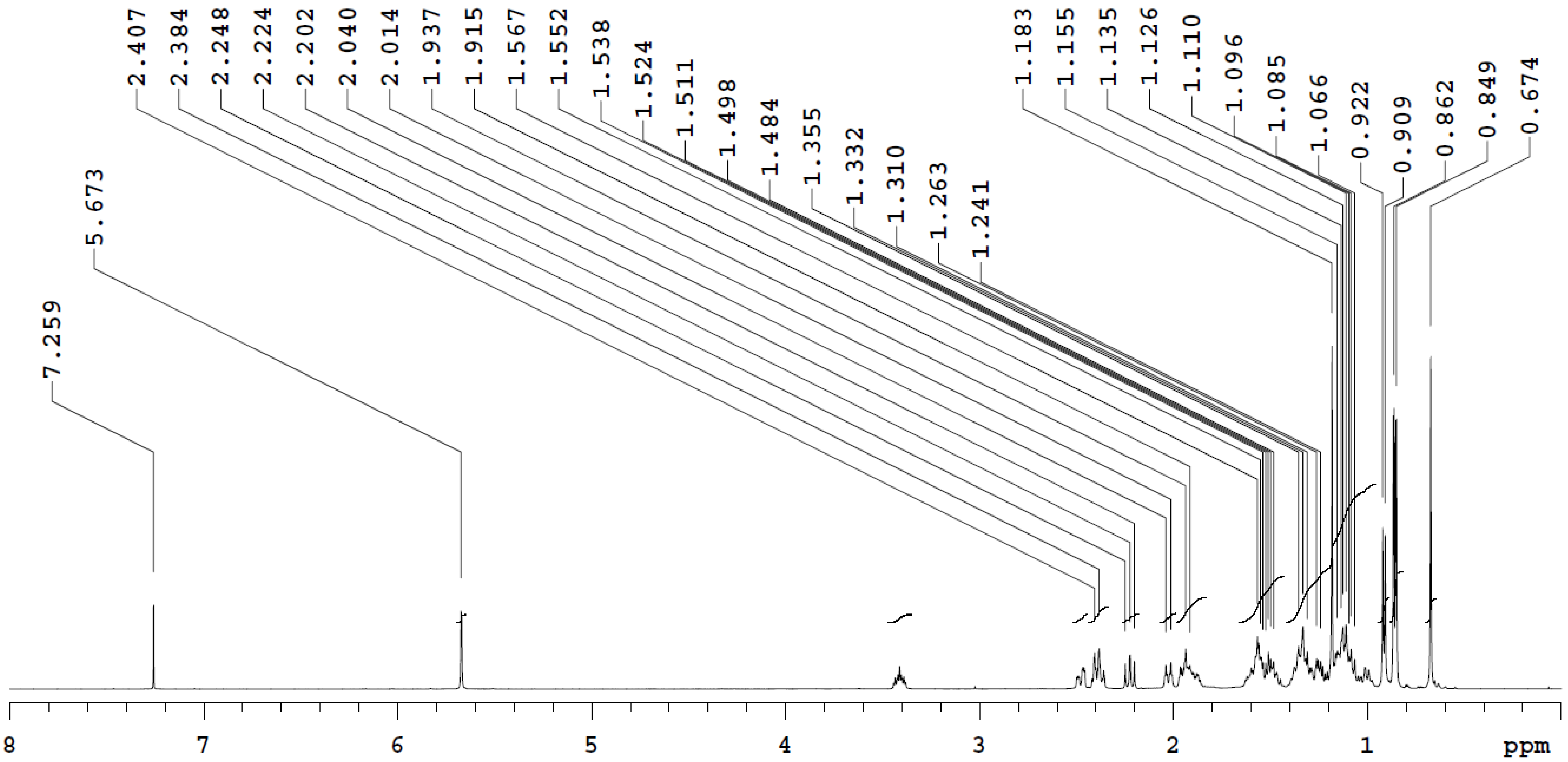

Figure S22. ^{1}H spectrum of 3β,3’β-di(cholest-5-en-7-one-3β-yl) ether (**7**) in $CDCl_3$

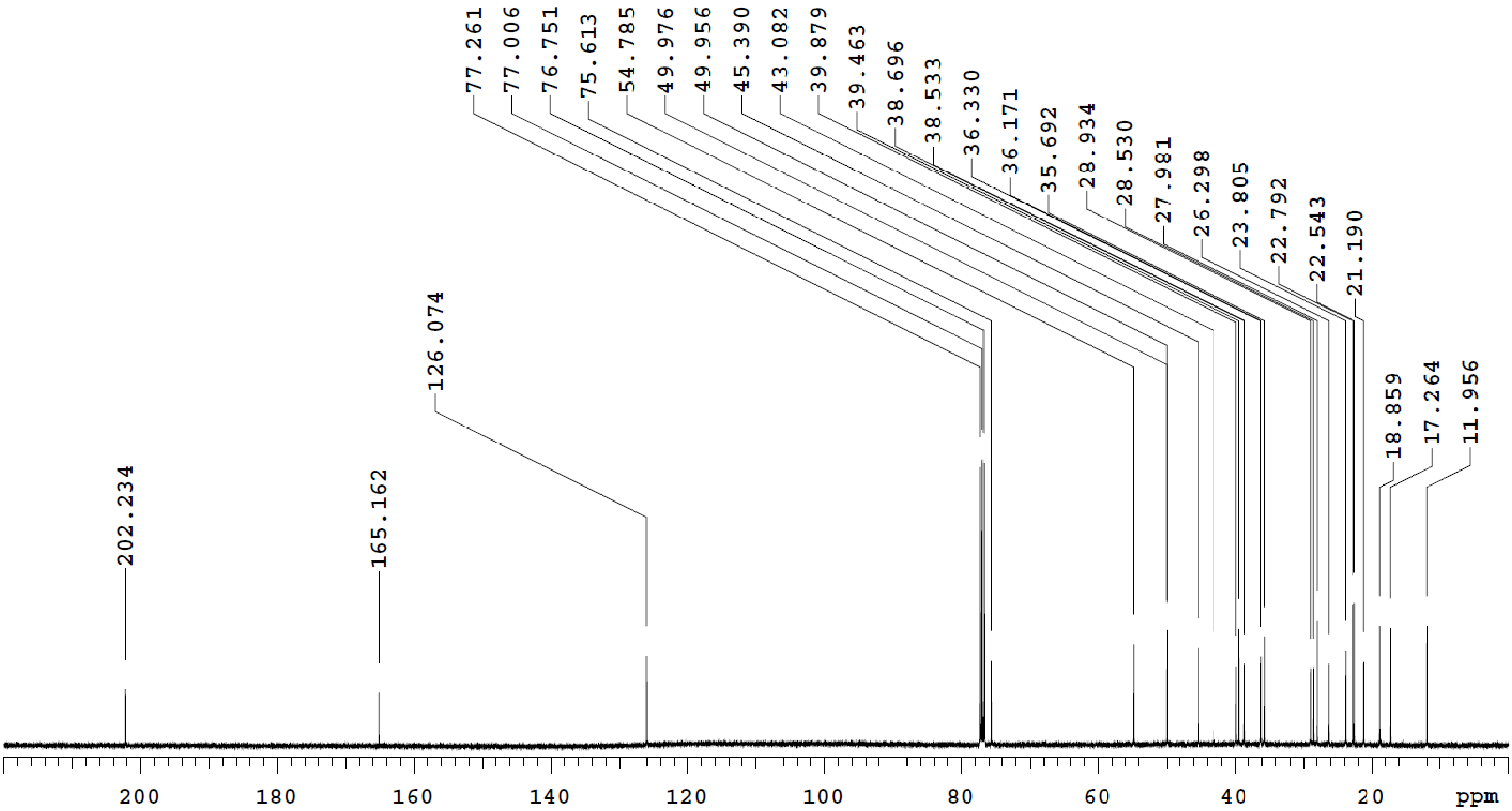

Figure S23. ^{13}C spectrum of 3β,3'β-di(cholest-5-en-7-one-3β-yl) ether (**7**) in $CDCl_3$

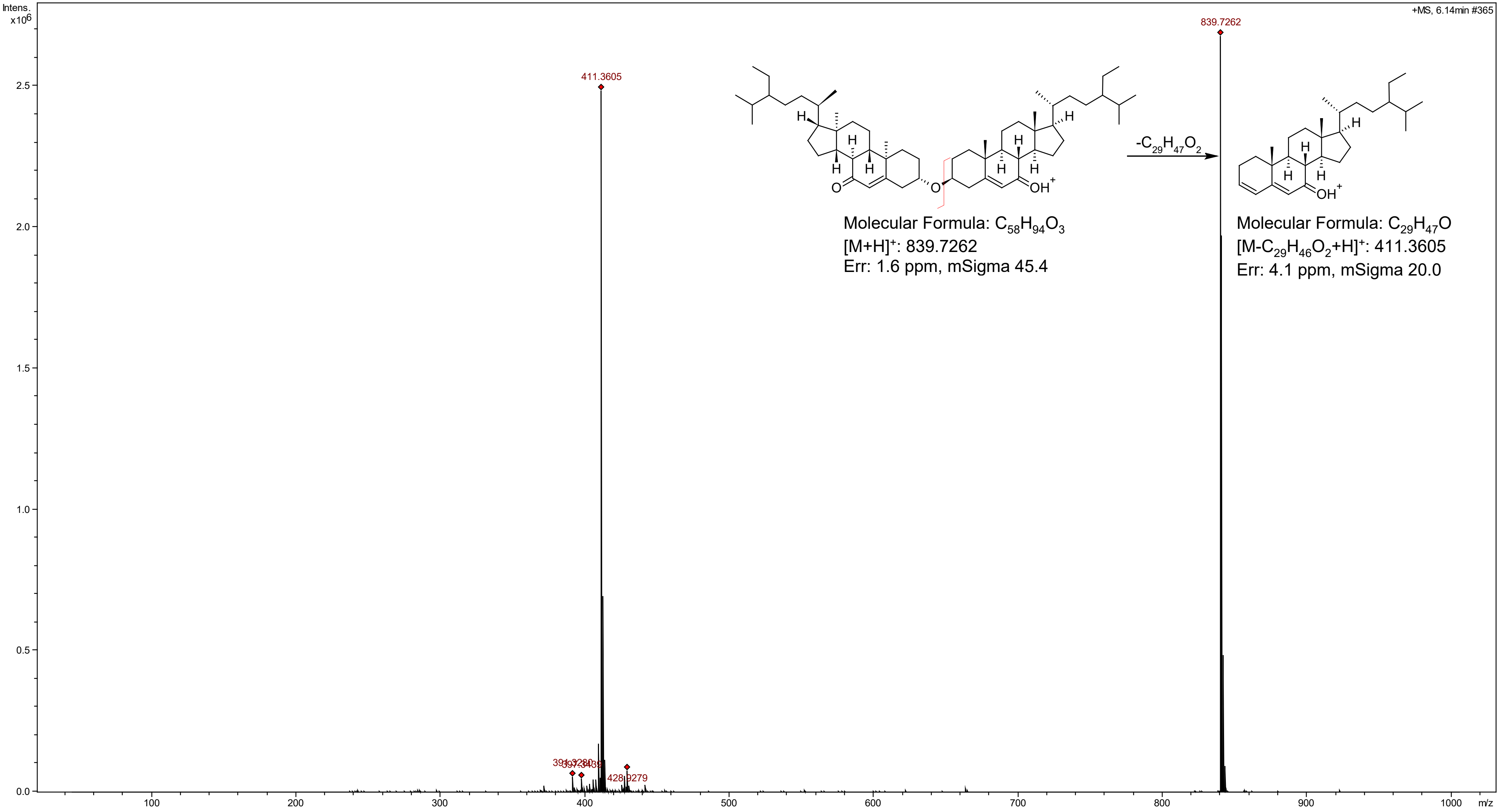

Figure S24. Mass spectrum of 3β,3’β-di(sitoste-5-en-7-one-3β-yl) ether (**8**)

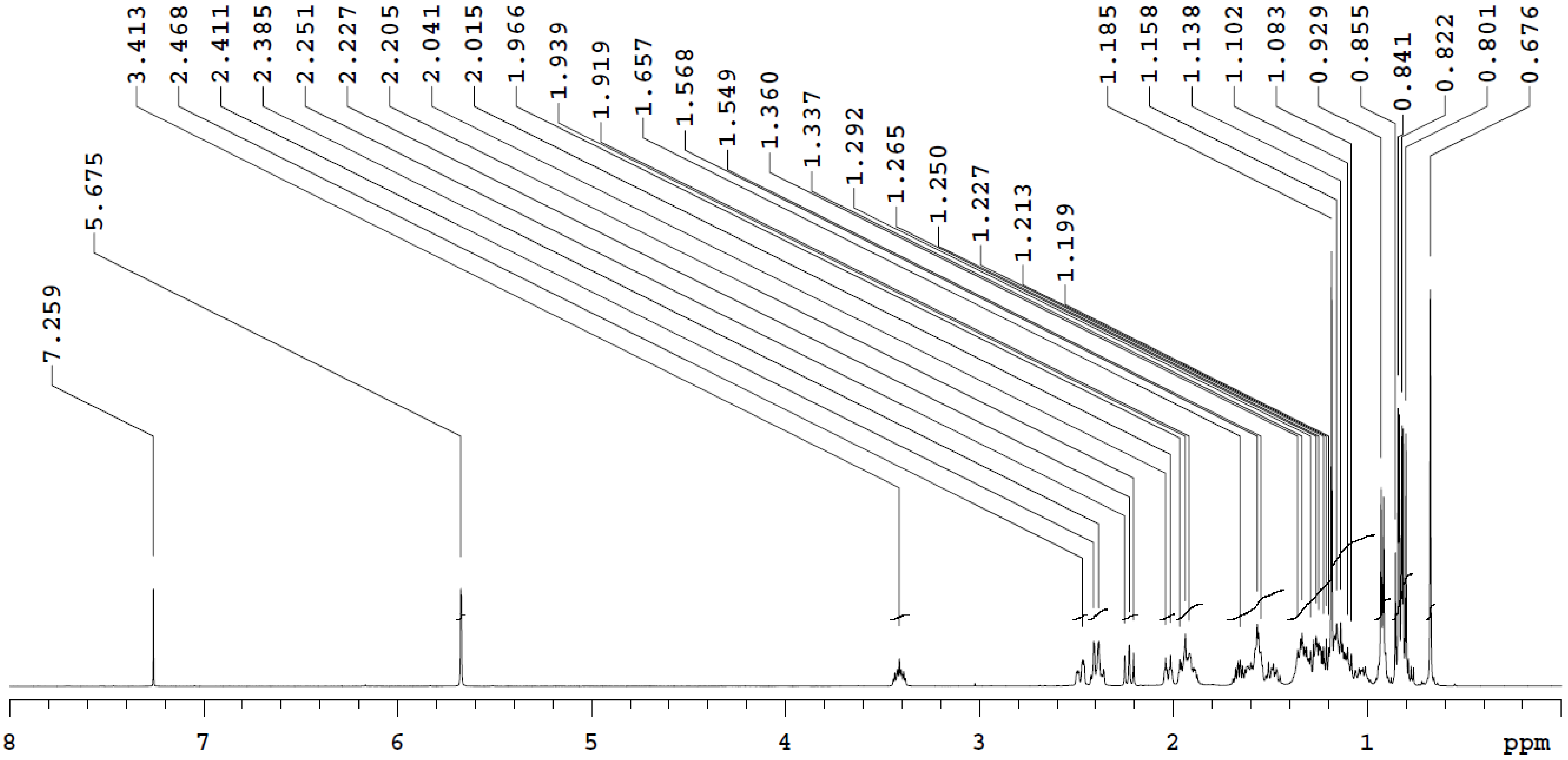

Figure S25. ^{1}H spectrum of 3β,3'β-di(sitoste-5-en-7-one-3β-yl) ether (**8**) in $CDCl_3$

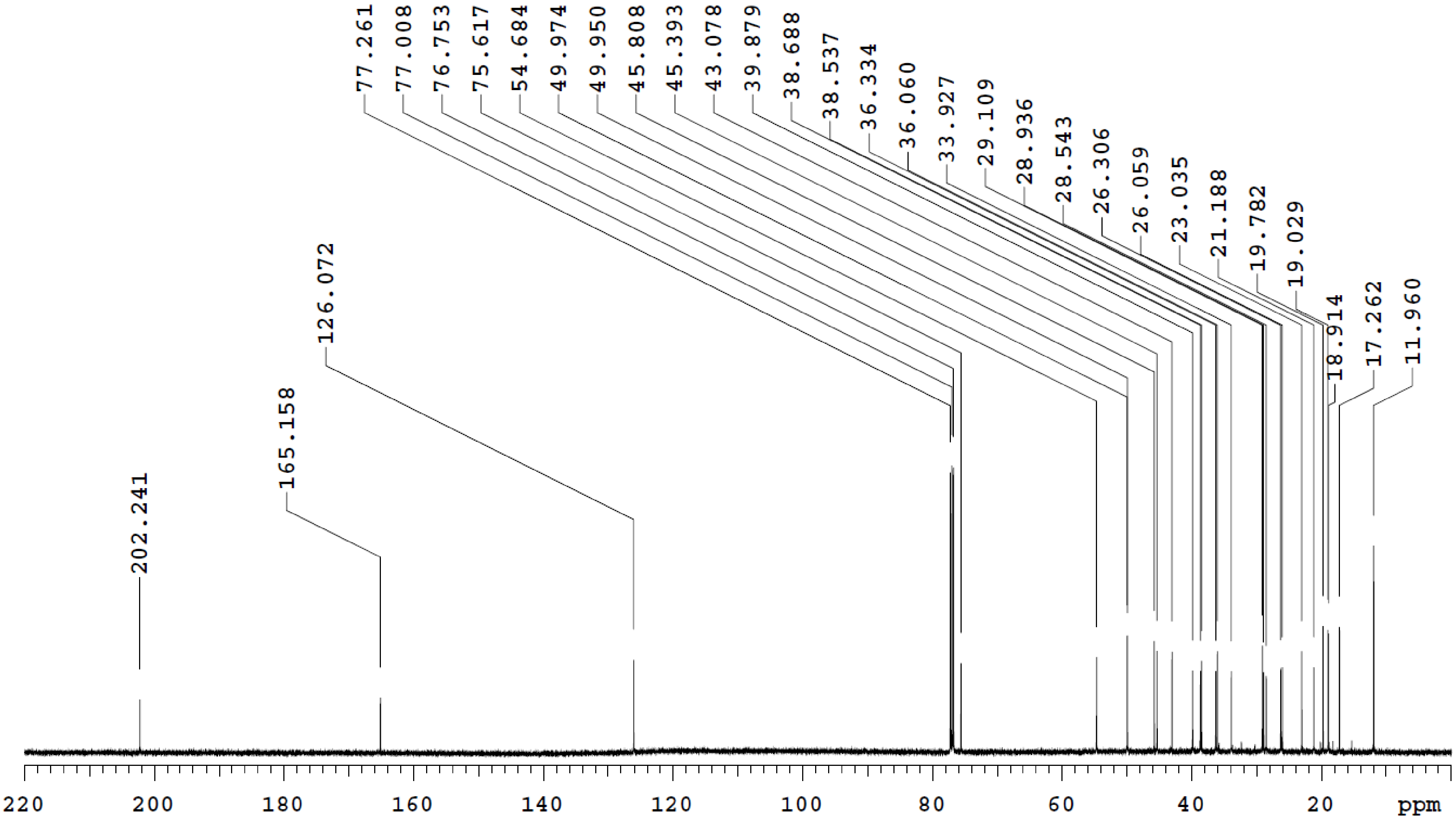

Figure S26. ^{13}C spectrum of 3β,3’β-di(sitost-5-en-7-one-3β-yl) ether (**8**) in $CDCl_3$

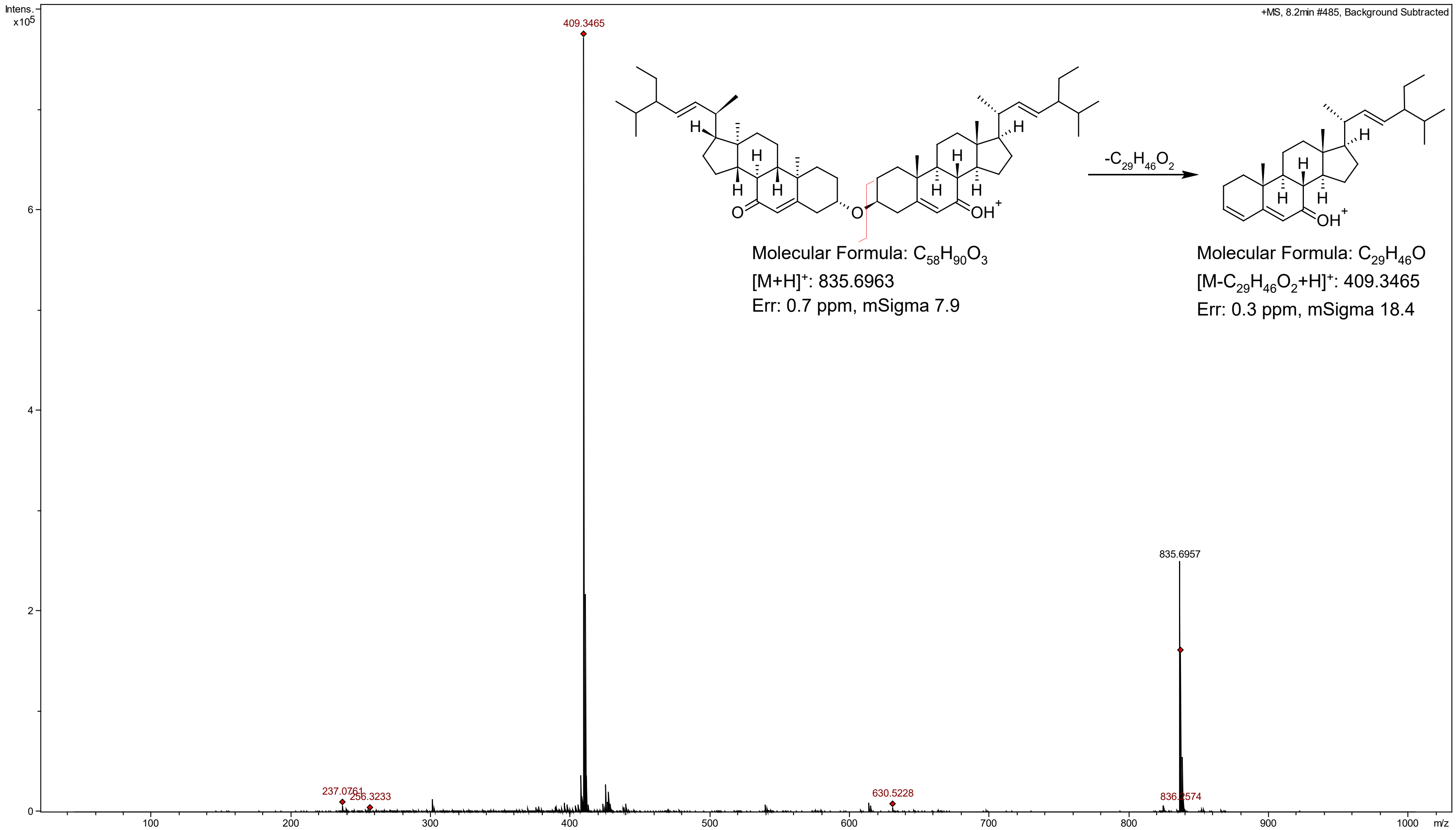

Figure S27. Mass spectrum of 3β,3'β-di(stigmast-5-en-7-one-3β-yl) ether (**9**)

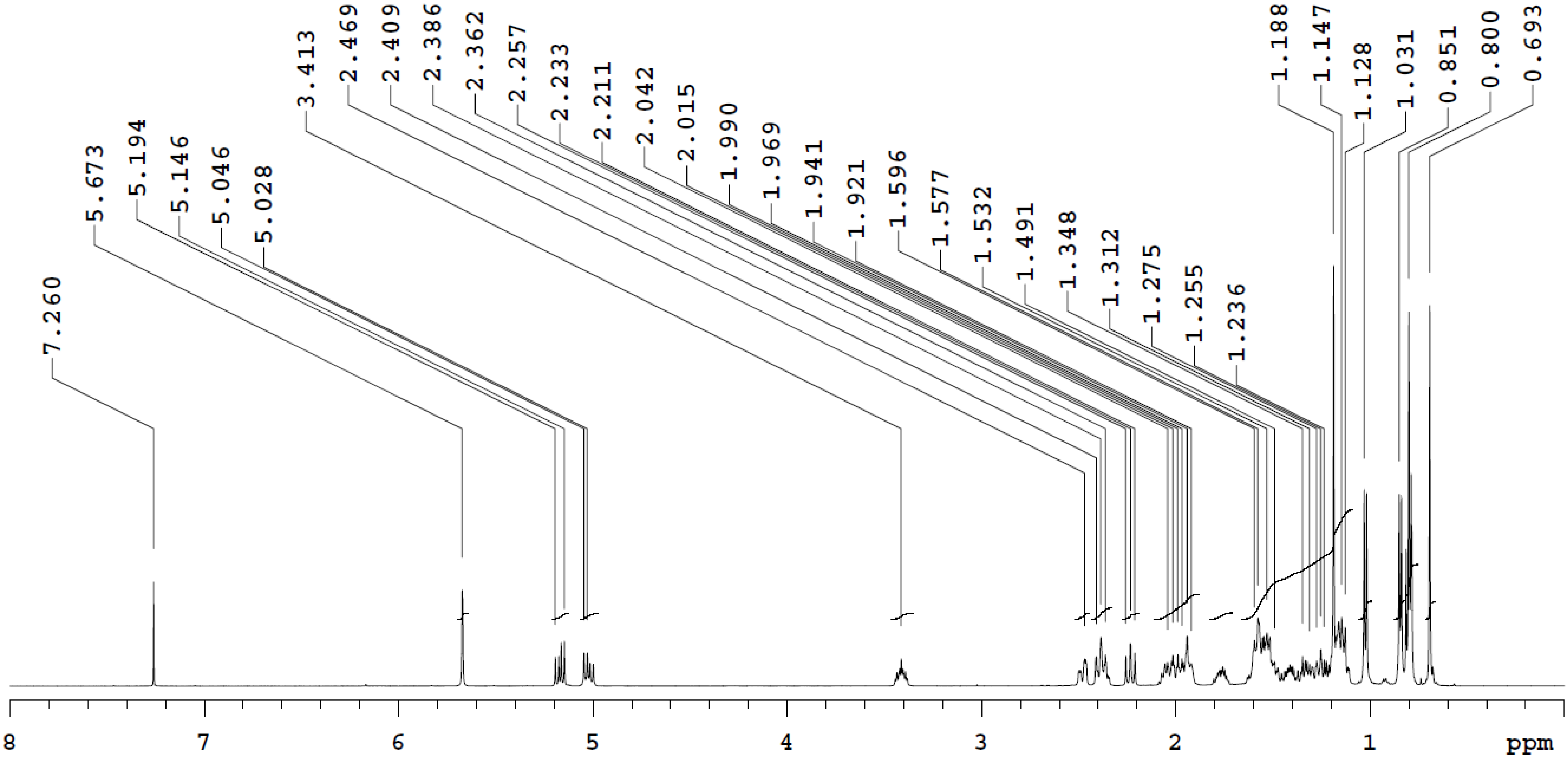

Figure S28. ^{1}H spectrum of 3β,3'β-di(stigmast-5-en-7-one-3β-yl) ether (**9**) in $CDCl_3$

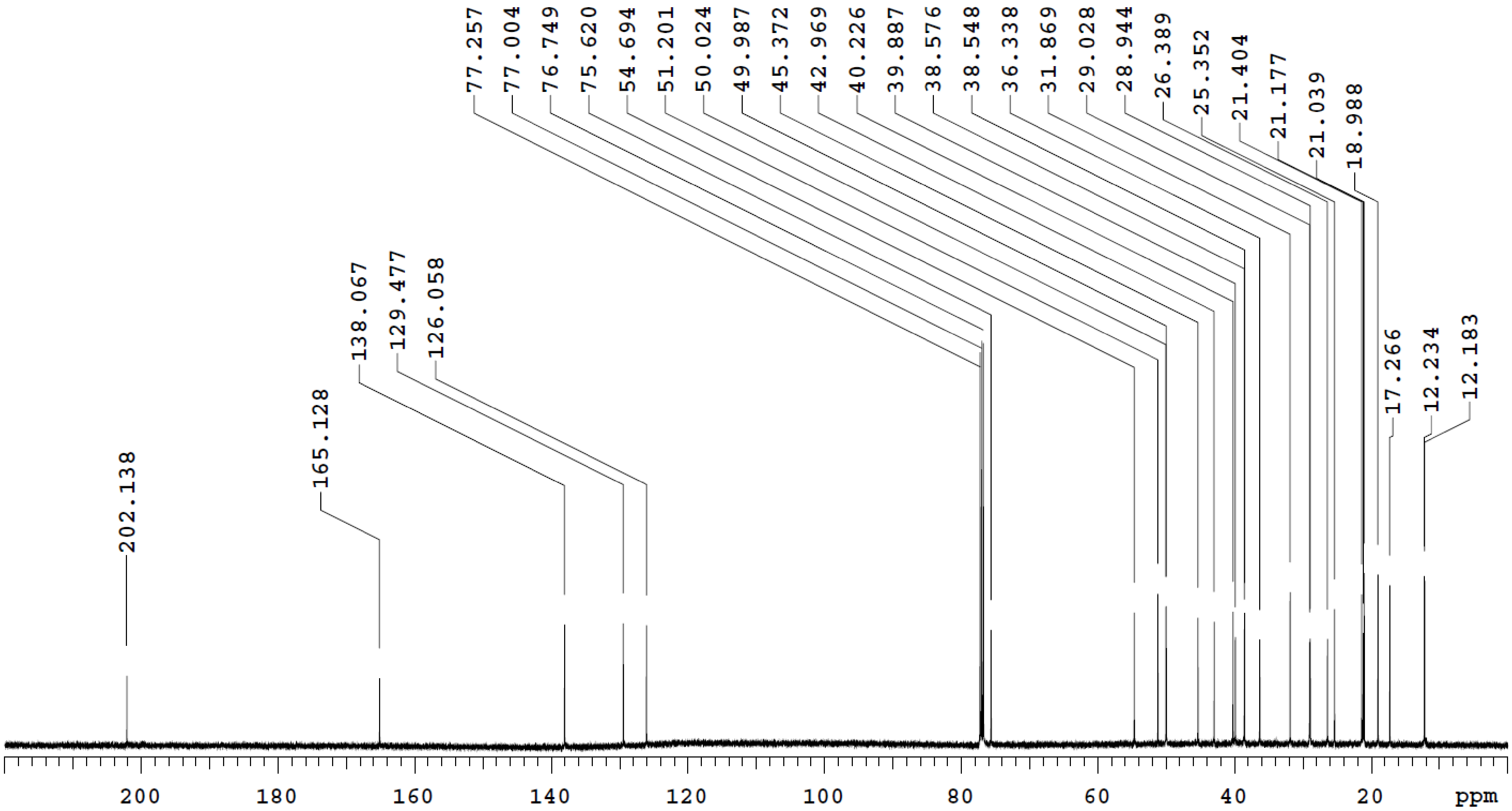

Figure S29. ^{13}C spectrum of 3β,3'β-di(stigmast-5-en-7-one-3β-yl) ether (**9**) in $CDCl_3$

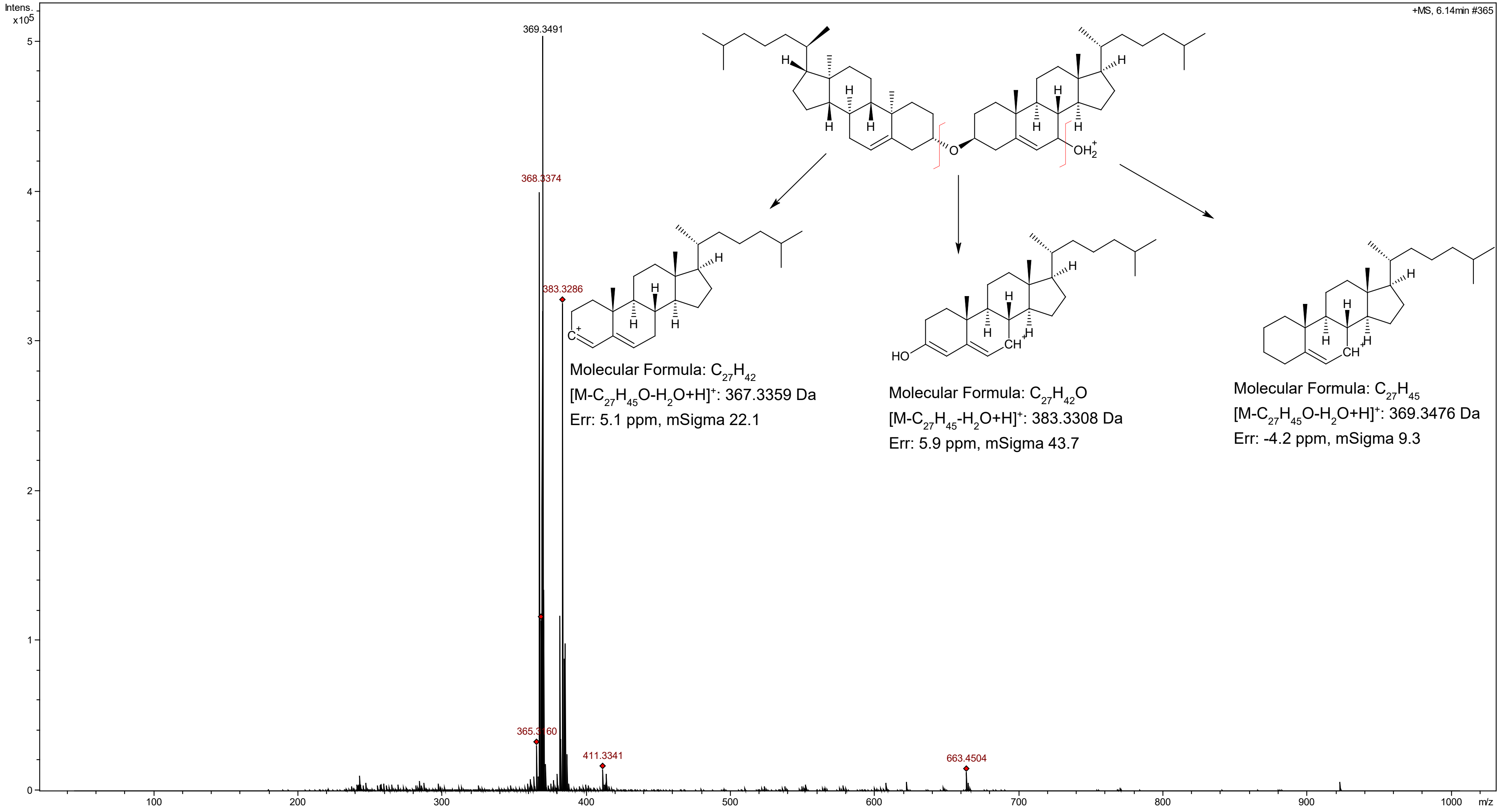

Figure S30. Mass spectrum of cholest-5-en-3β,7α-diol-cholesterol ether (7α-hydroxycholesteryl-cholesterol ether) (**10**)

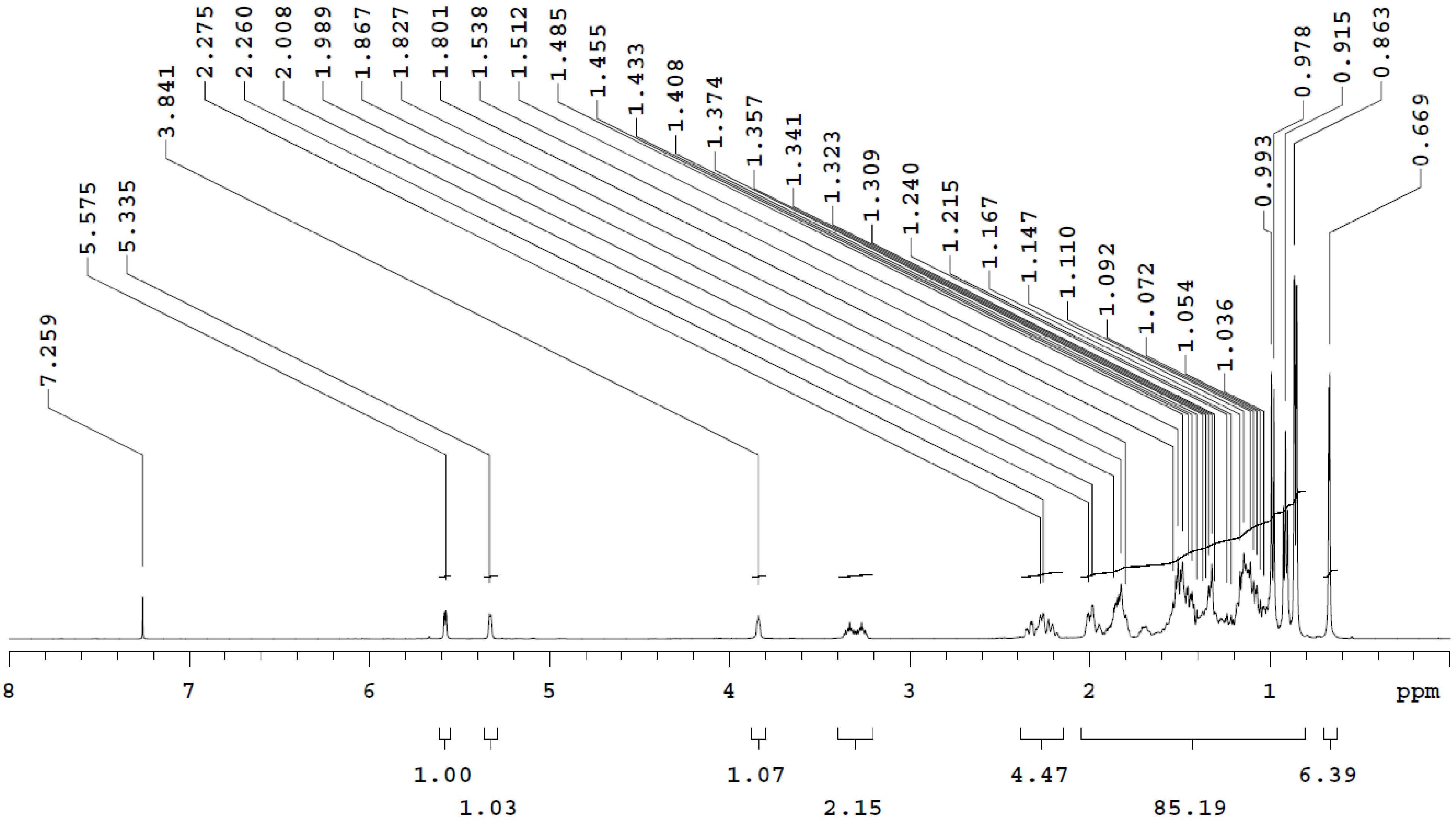

Figure S31. ^{1}H spectrum of cholest-5-en-3β,7α-diol-cholesterol ether (7α-hydroxycholesteryl-cholesterol ether) (**10**) in $CDCl_3$

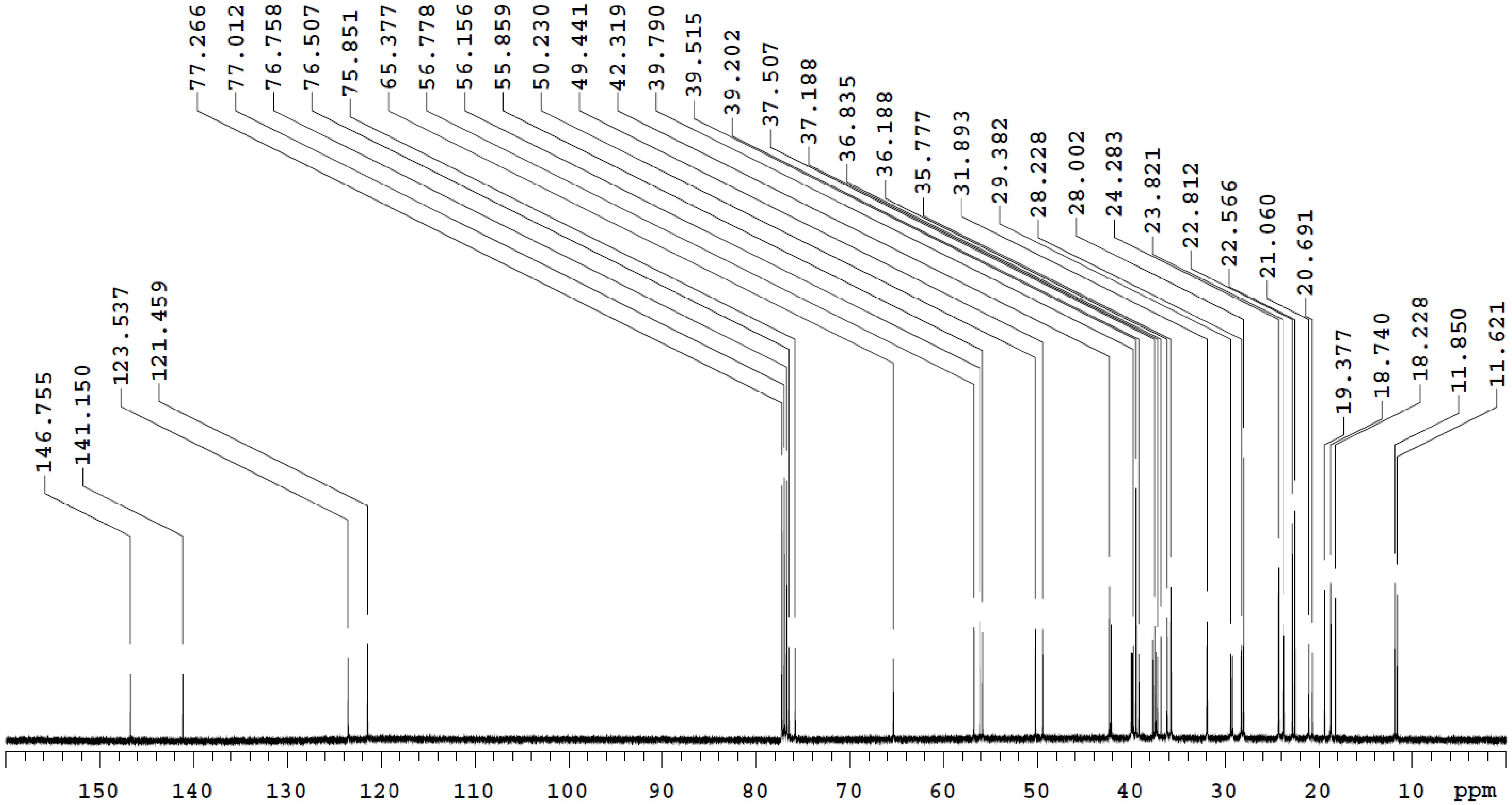

Figure S32. ^{13}C spectrum of cholest-5-en-3β,7α-diol-cholesterol ether (7α-hydroxycholesteryl-cholesterol ether) (**10**) in $CDCl_3$

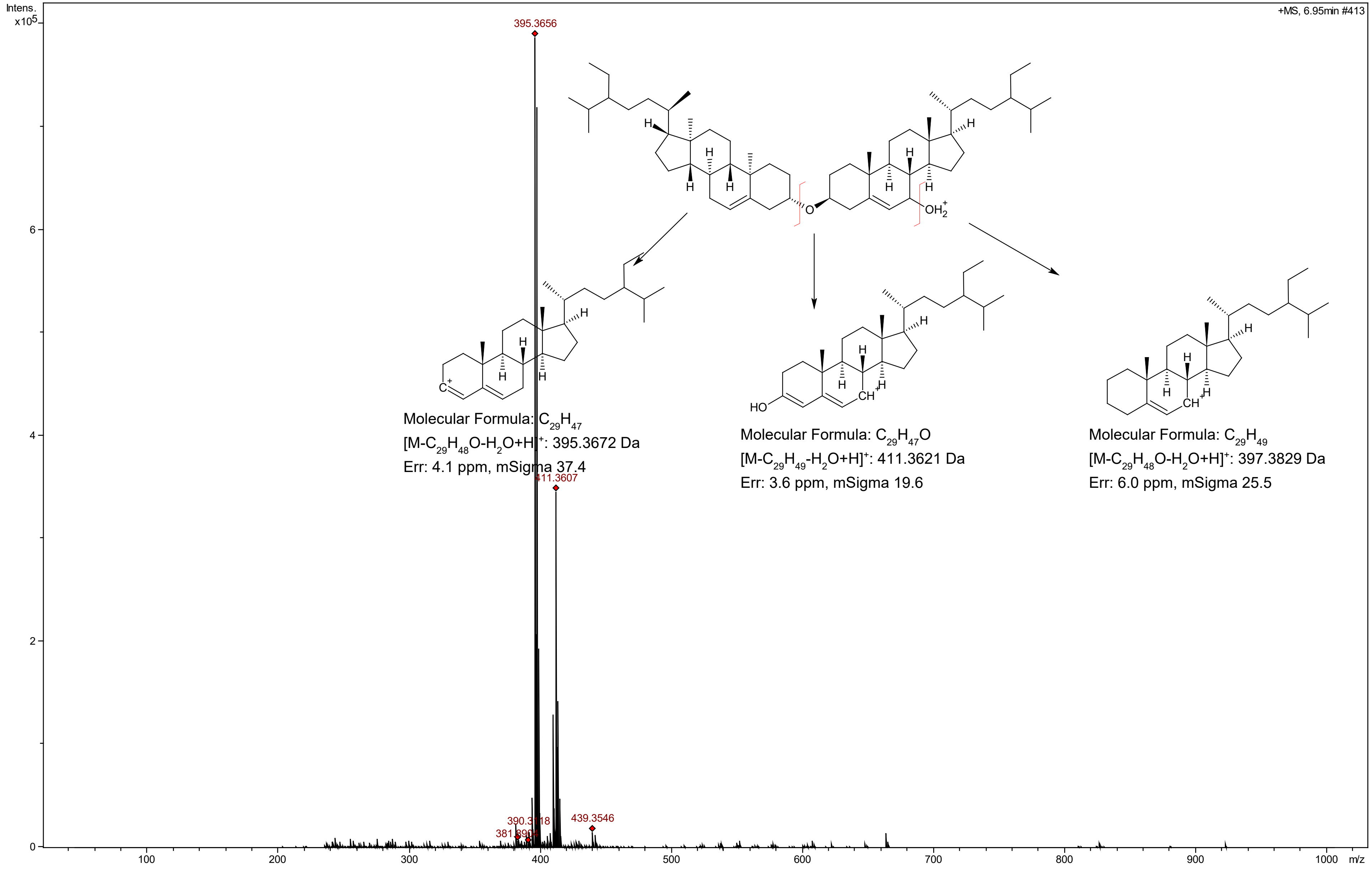

Figure S33. Mass spectrum of sitost-5-en-3β,7α-diol-sitosterol ether (7α-hydroxysitosteryl-sitosterol ether) (**11**).

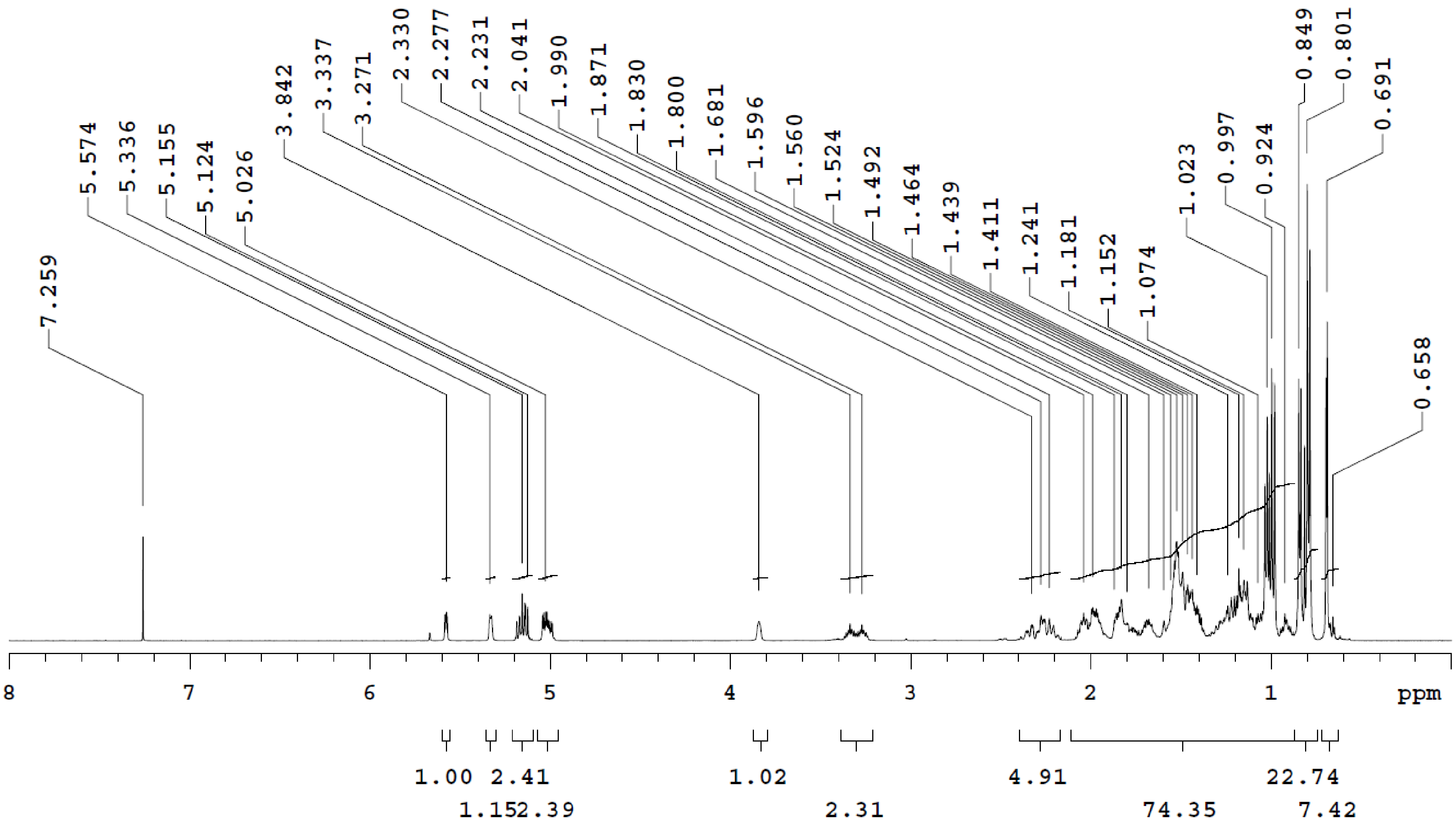

Figure S34. ^{1}H spectrum of sitost-5-en-3β,7α-diol-sitosterol ether (7α-hydroxysitosteryl-sitosterol ether) (**11**) in $CDCl_3$.

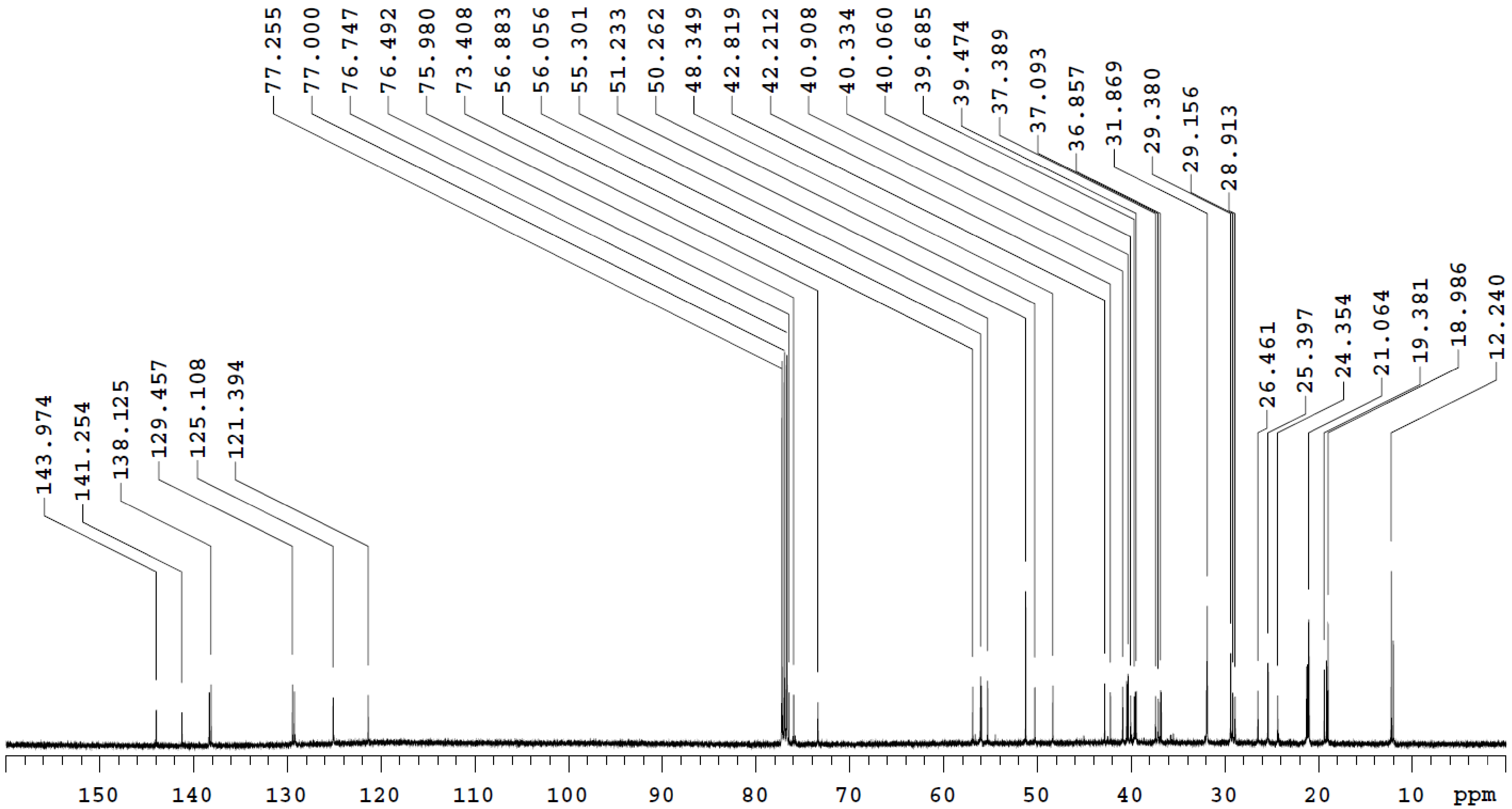

Figure S35. ^{13}C spectrum of sitost-5-en-3β,7α-diol-sitosterol ether (7α-hydroxysitosteryl-sitosterol ether) (**11**) in $CDCl_3$

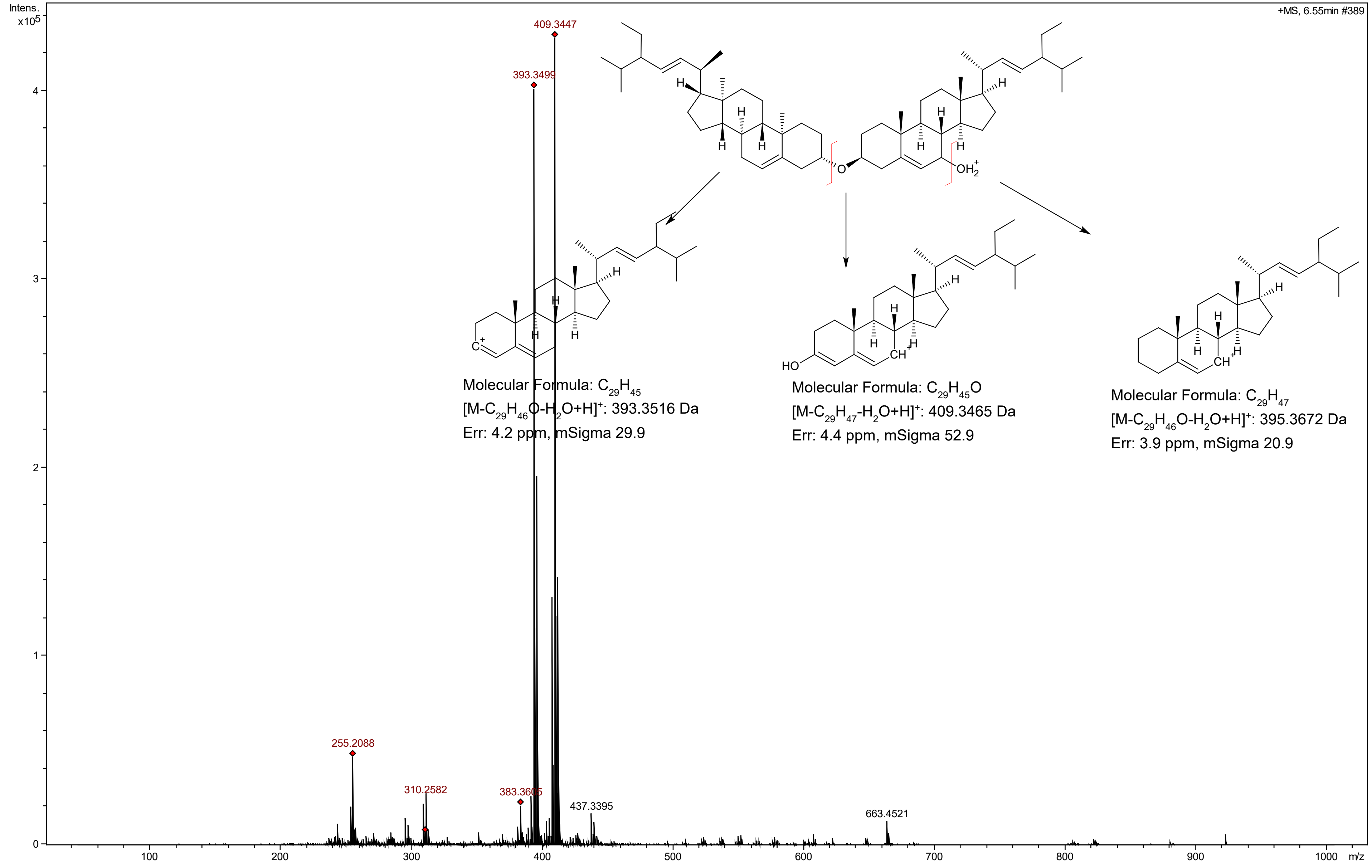

Figure S36. Mass spectrum of stigmast-5-en-3β,7α-diol-stigmasterol ether (7α-hydroxystigmasteryl-stigmasterol ether) (**12**).

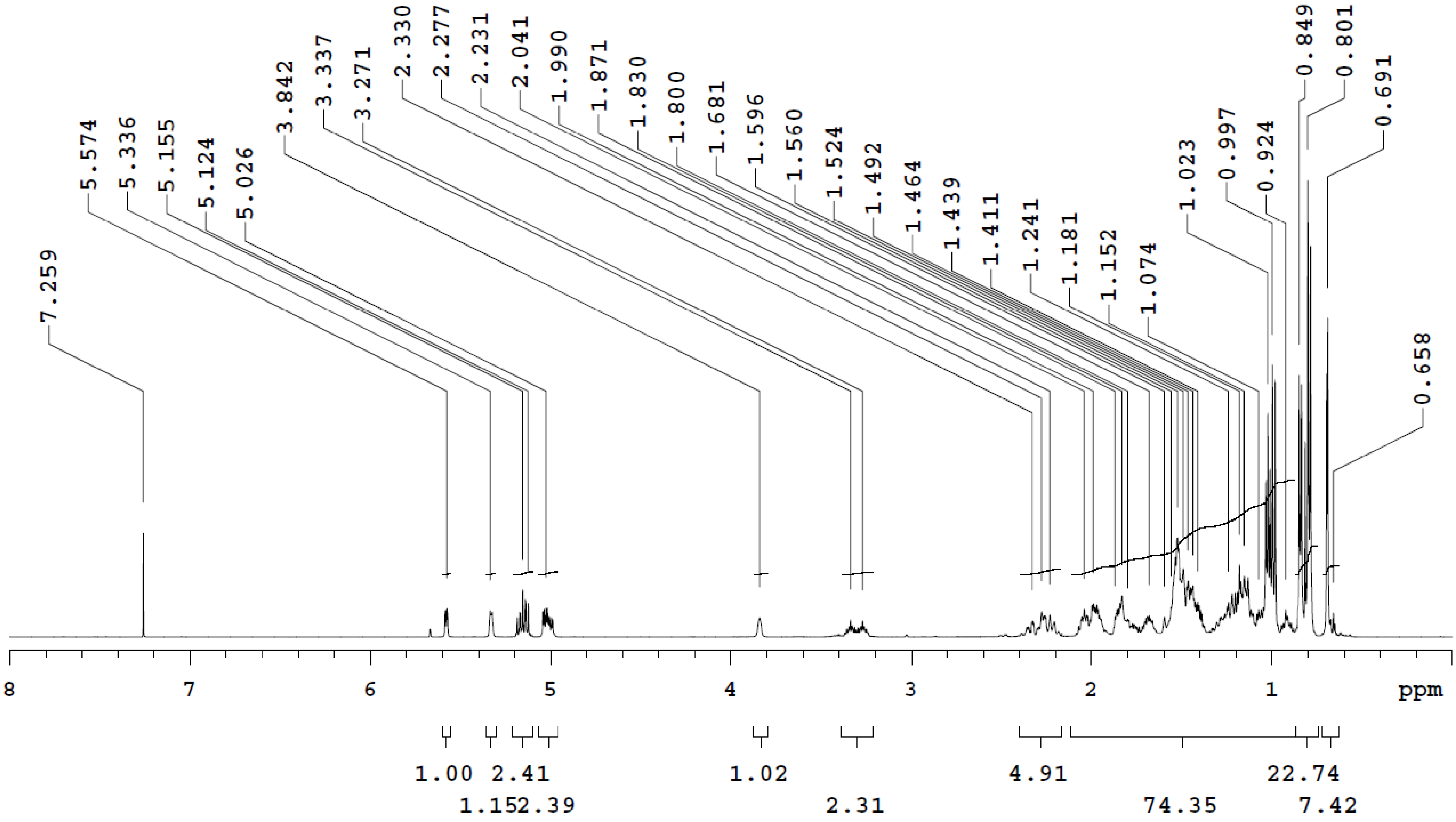

Figure S37. ^{1}H spectrum of stigmast-5-en-3β,7α-diol-stigmasterol ether (7α-hydroxystigmasteryl-stigmasterol ether) (**12**) in $CDCl_3$.

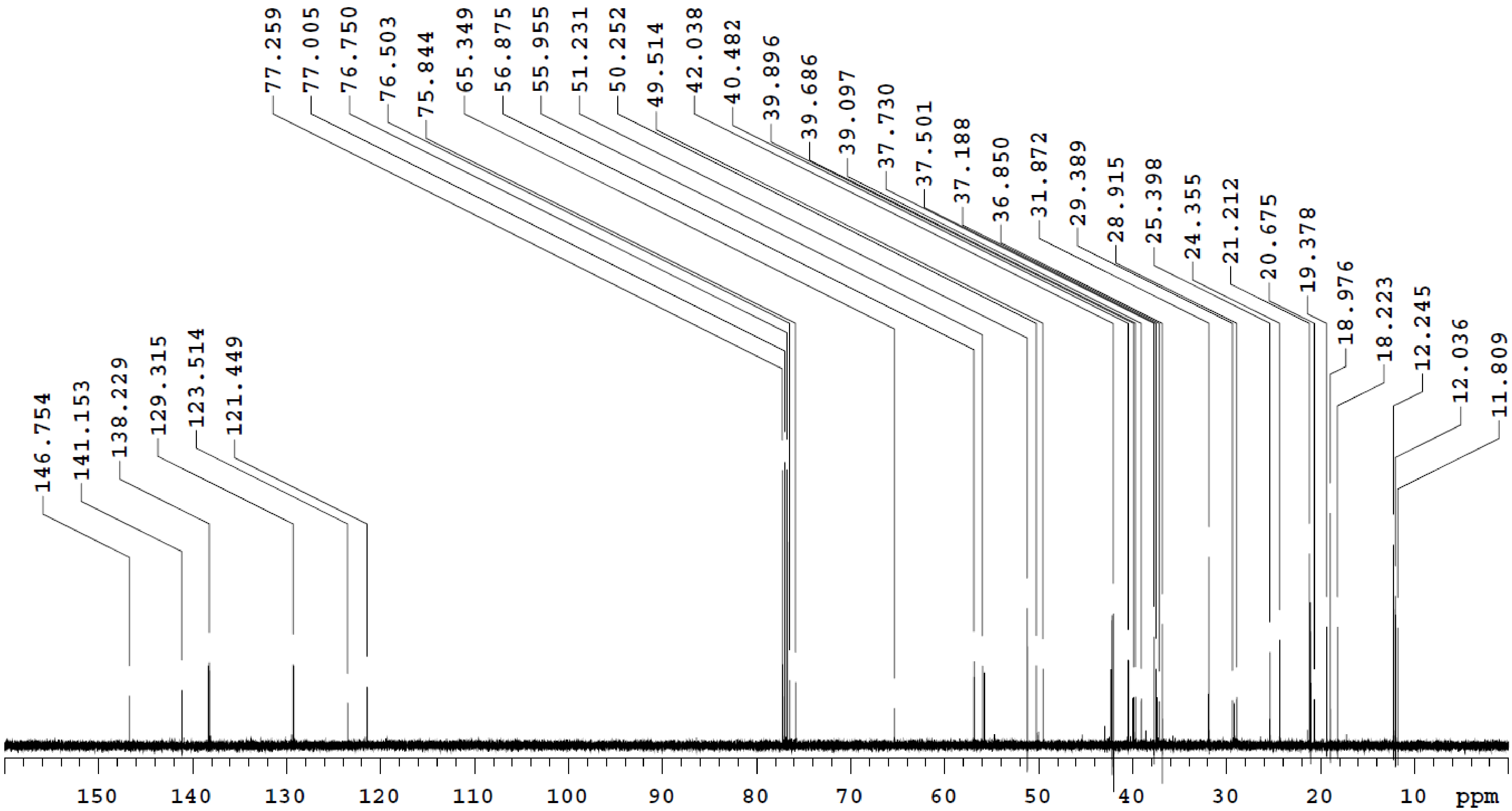

Figure S38. ^{13}C spectrum of stigmast-5-en-3β,7α-diol-stigmasterol ether (7α-hydroxystigmasteryl-stigmasterol ether) (**12**) in $CDCl_3$.

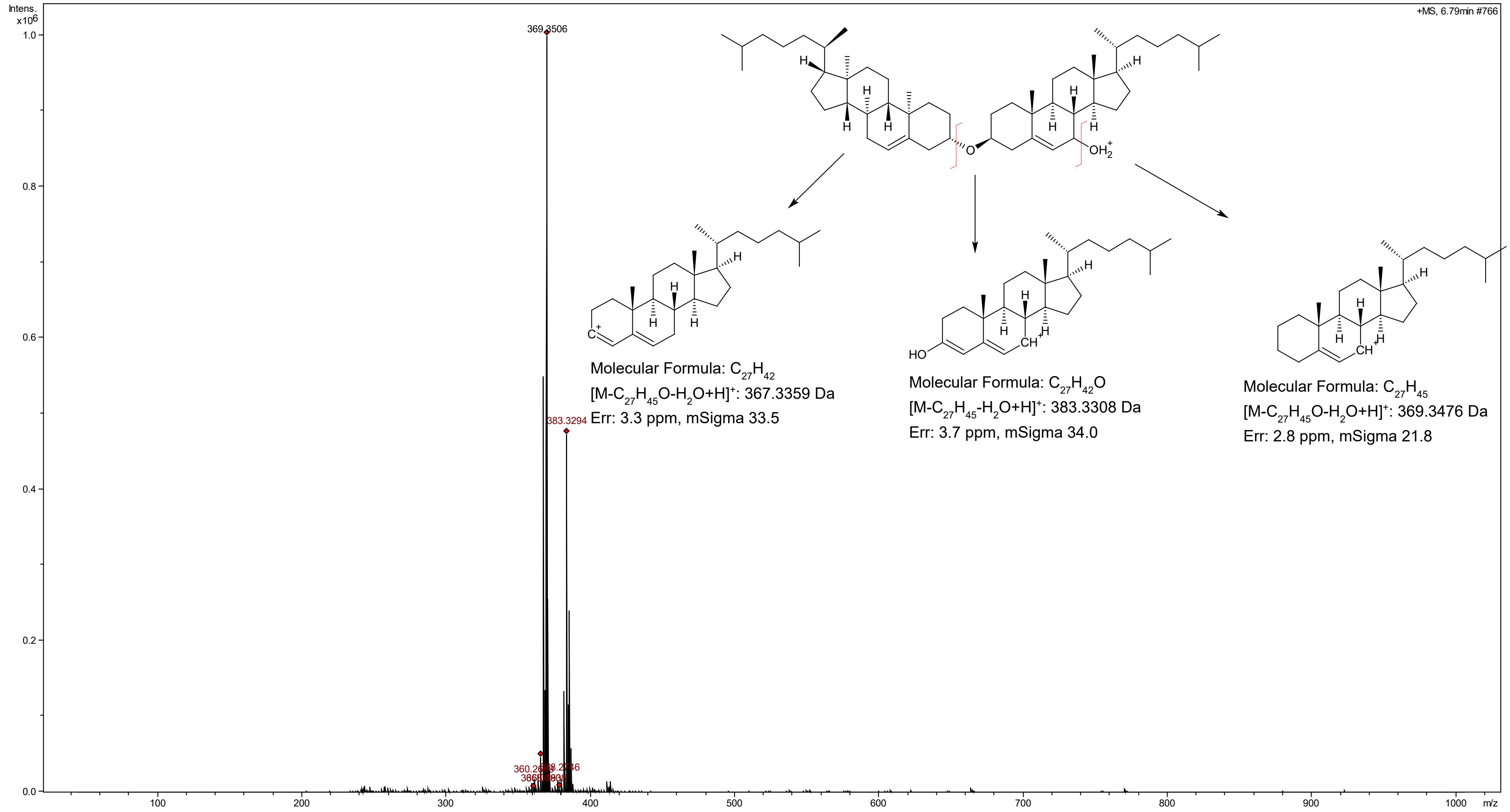

Figure S39. Mass spectrum of cholest-5-en-3β,7β-diol-cholesterol ether (7β-hydroxycholesteryl-cholesterol ether) (**13**).

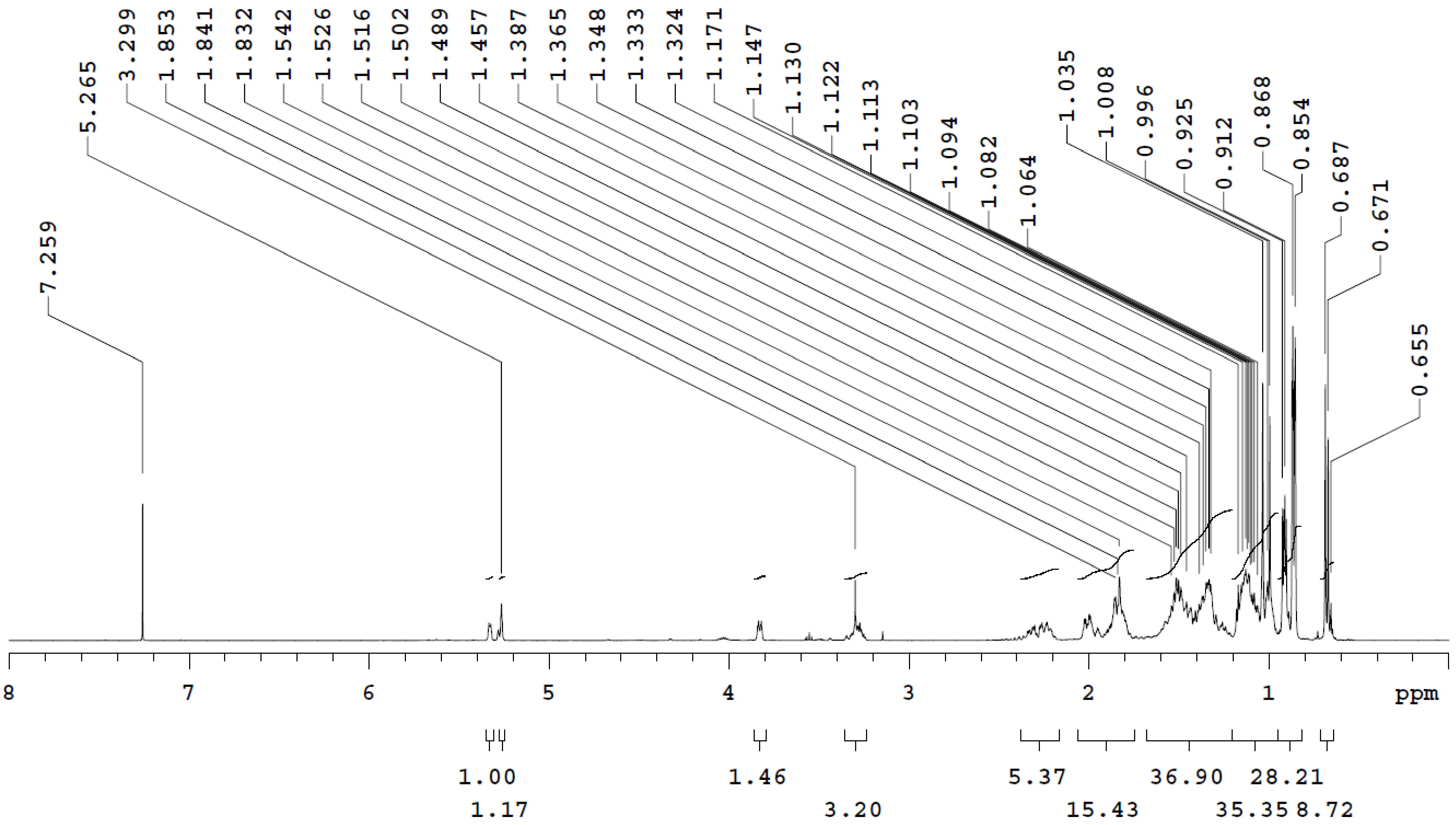

Figure S40. ^{1}H spectrum of cholest-5-en-3β,7β-diol-cholesterol ether (7β-hydroxycholesteryl-cholesterol ether) (**13**) in $CDCl_3$.

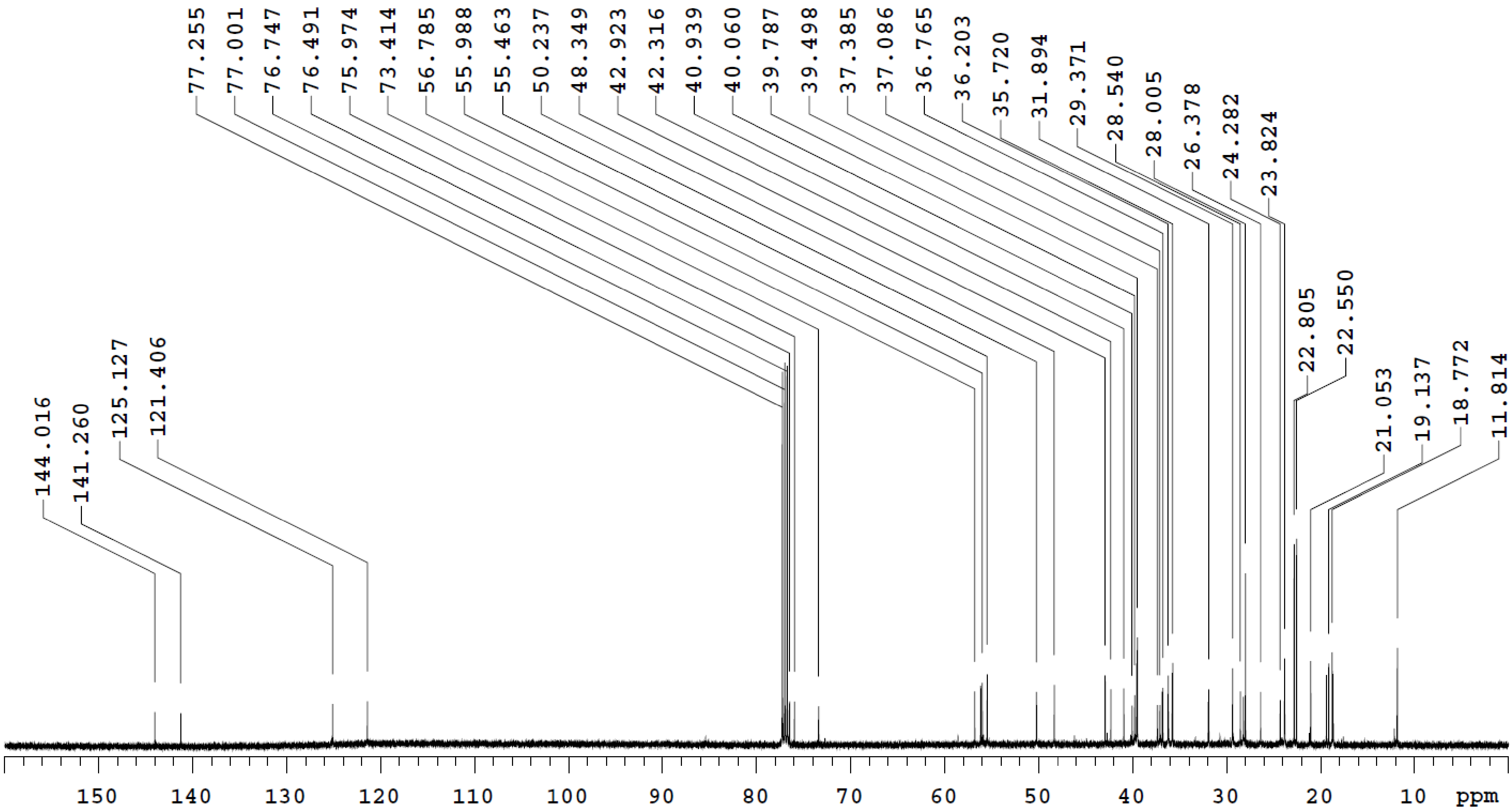

Figure S41. ^{13}C spectrum of cholest-5-en-3β,7β-diol-cholesterol ether (7β-hydroxycholesteryl-cholesterol ether) (**13**) in $CDCl_3$.

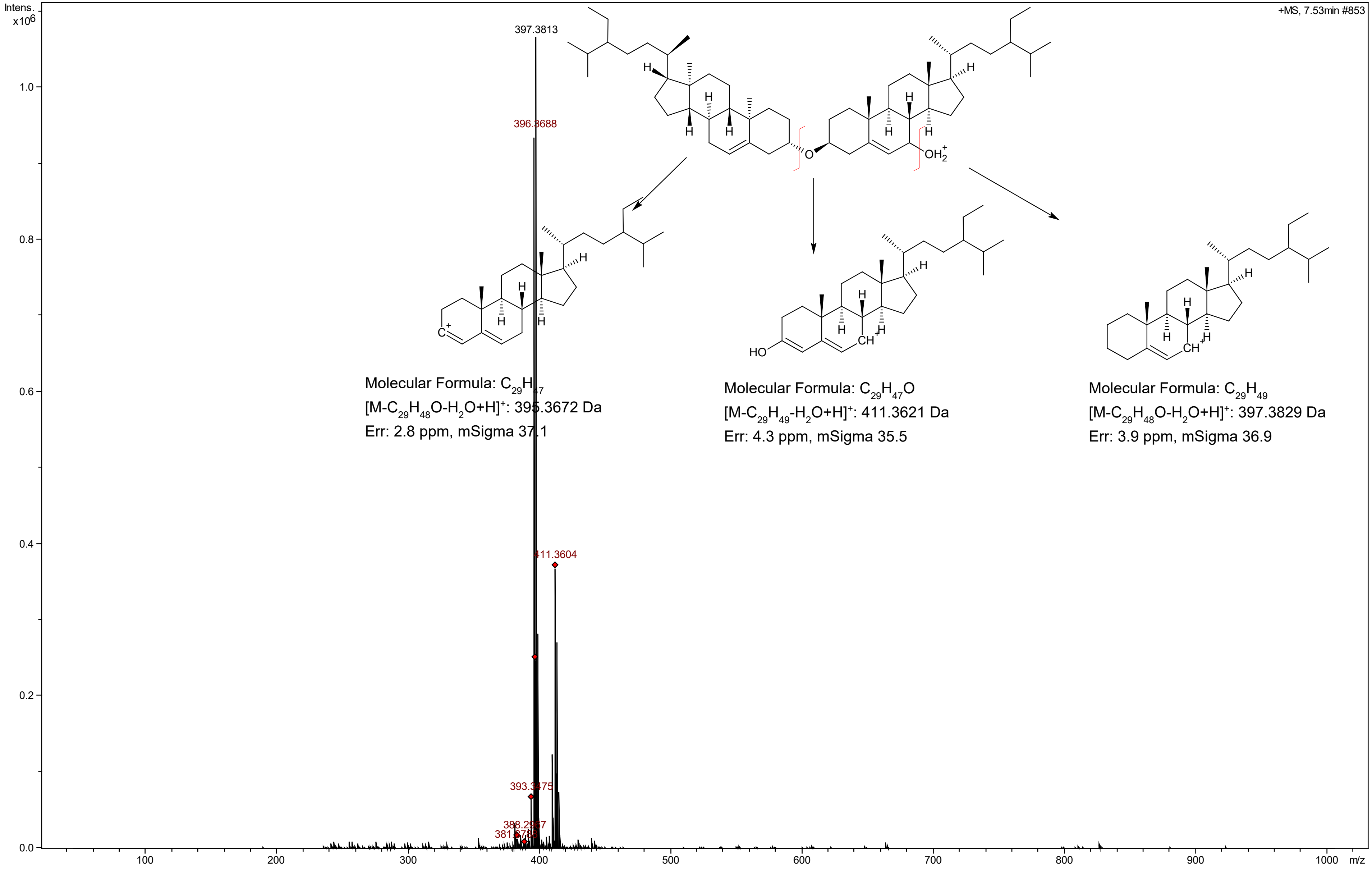

Figure S42. Mass spectrum of sito-5-en-3β,7β-diol-sitosterol ether (7β-hydroxysitosteryl-sitosterol ether) (**14**).

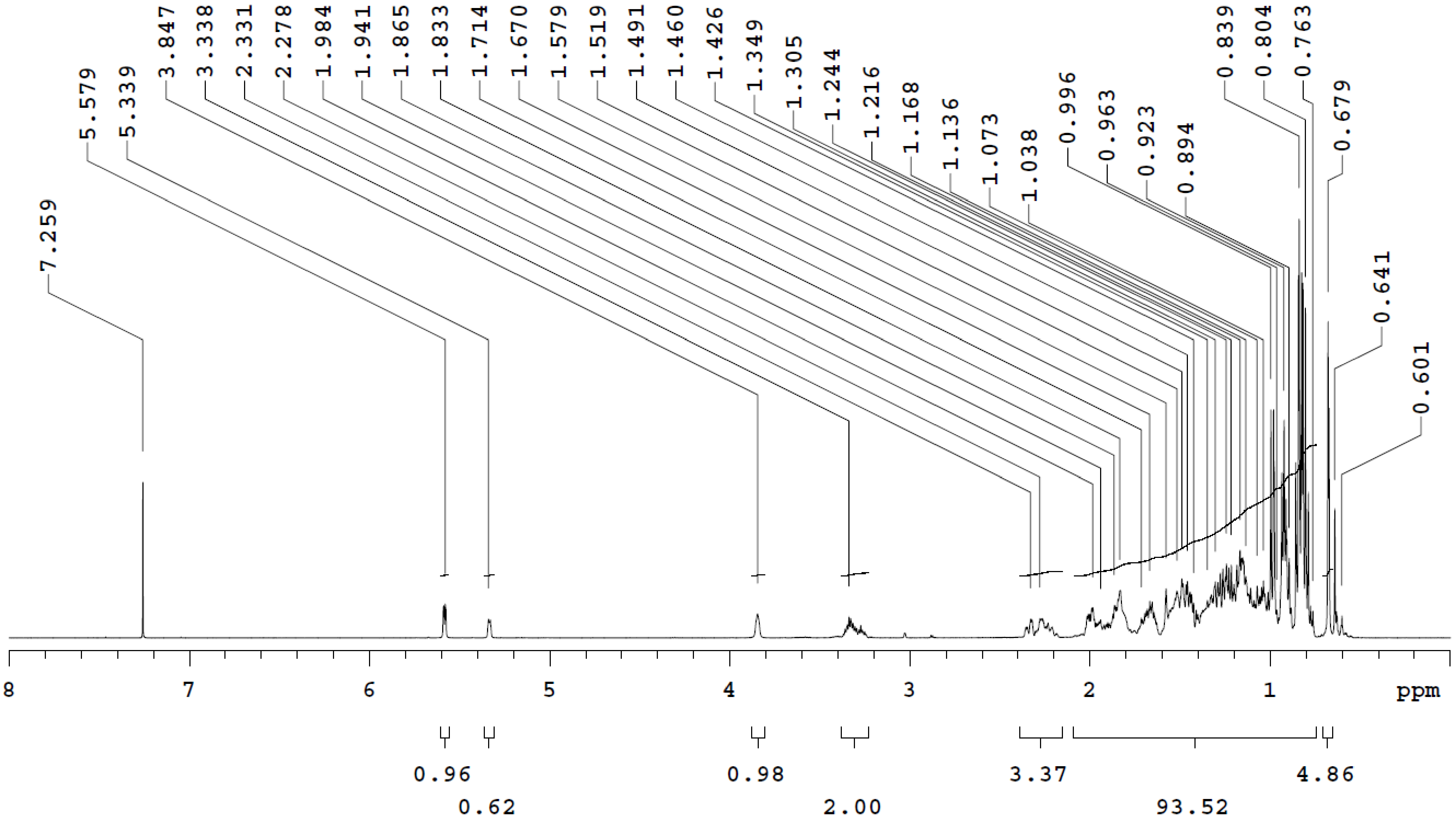

Figure S43. ^{1}H spectrum of sito-5-en-3β,7β-diol-sitosterol ether (7β-hydroxysitosteryl-sitosterol ether) (**14**) in $CDCl_3$.

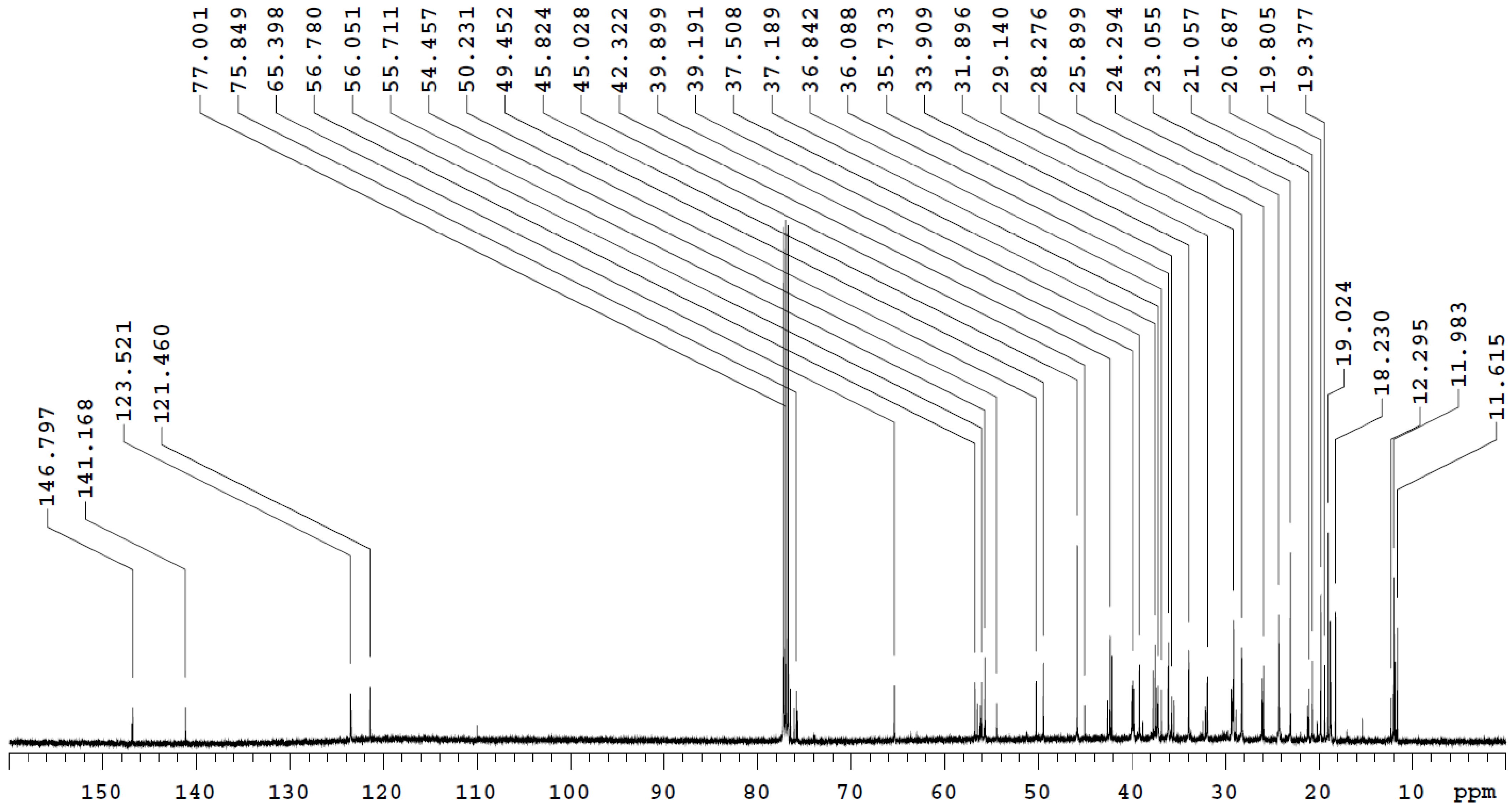

Figure S44. ^{13}C spectrum of sito-5-en-3β,7β-diol-sitosterol ether (7β-hydroxysitosteryl-sitosterol ether) (**14**) in $CDCl_3$.

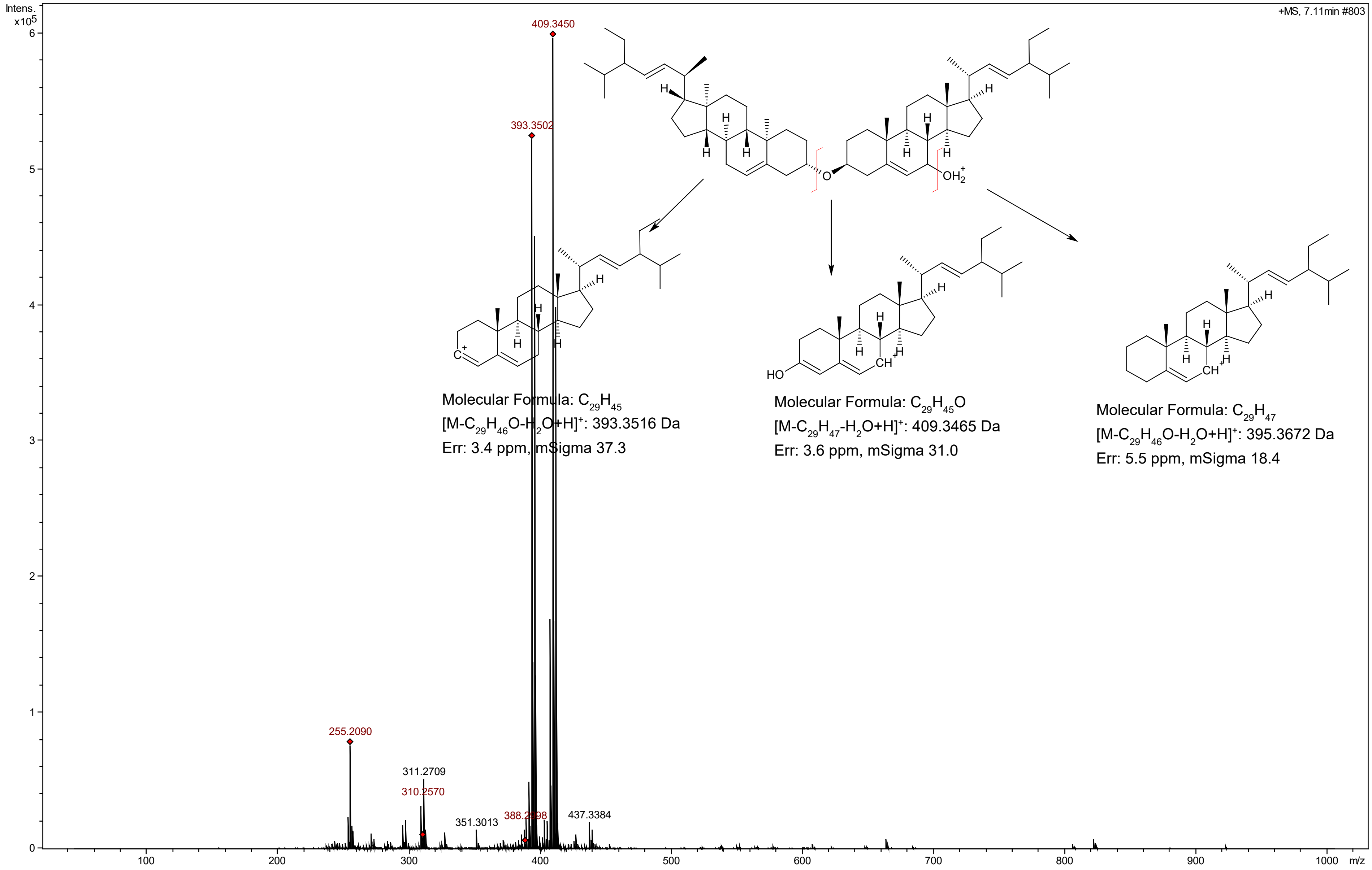

Figure S45. Mass spectrum of stigma-5-en-3β,7β-diol-stigmasterol ether (7β-hydroxystigmasteryl-stigmasterol ether) (**15**).

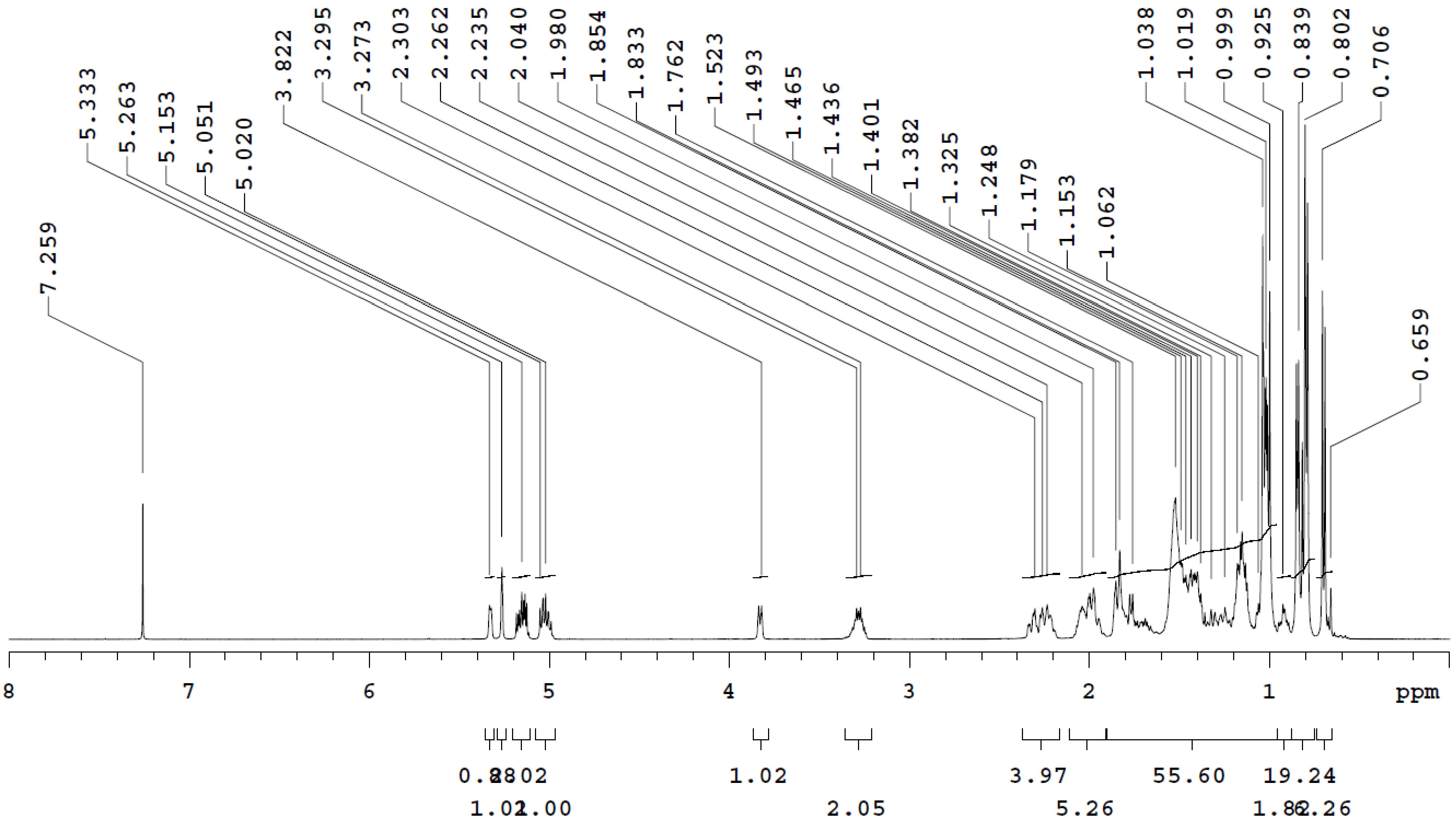

Figure S46. ^{1}H spectrum of stigma-5-en-3β,7β-diol-stigmasterol ether (7β-hydroxystigmasteryl-stigmasterol ether) (**15**) in $CDCl_3$.

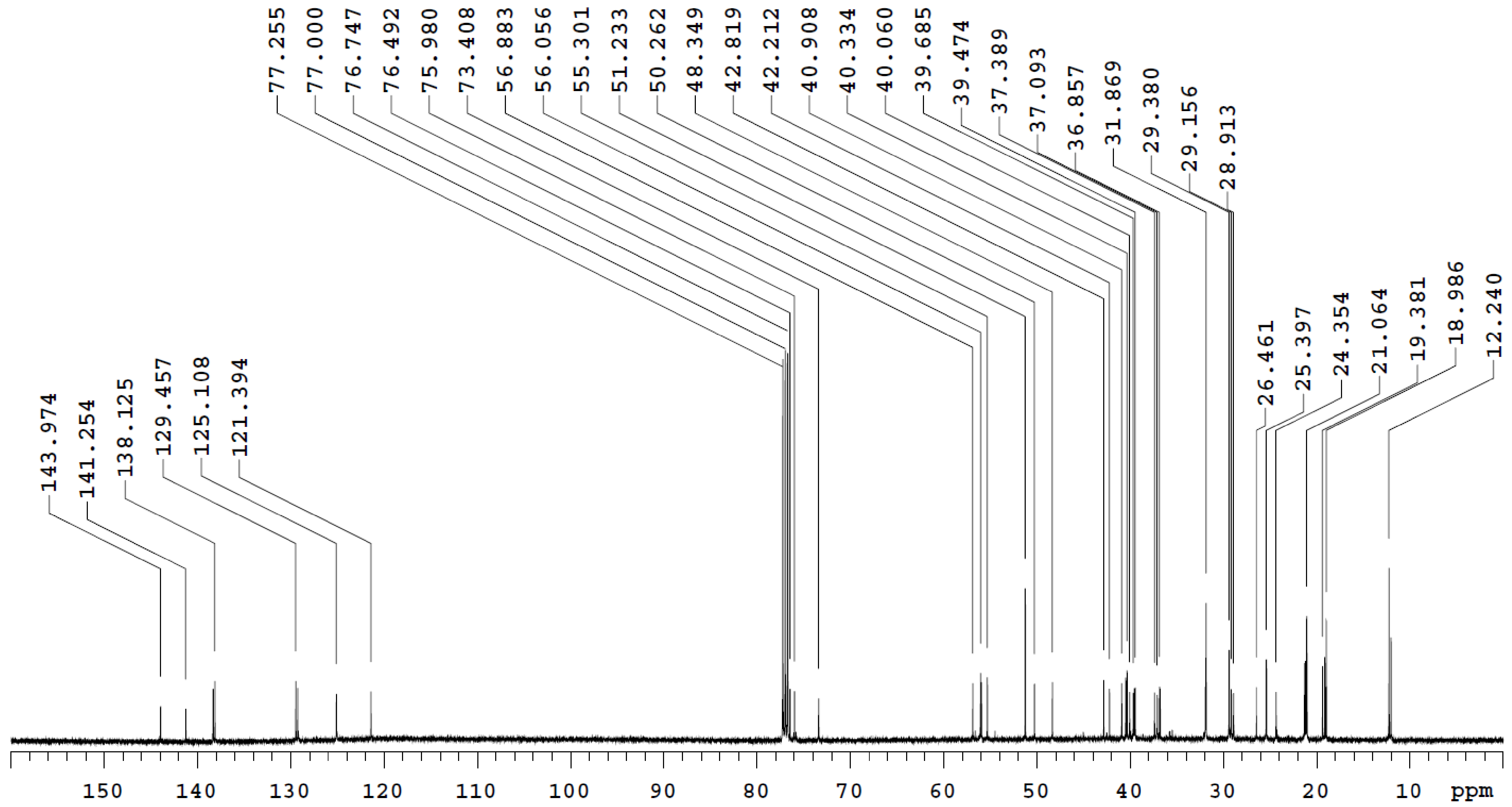

Figure S47. ^{13}C spectrum of stigma-5-en-3β,7β-diol-stigmasterol ether (7β-hydroxystigmasteryl-stigmasterol ether) (**15**) in $CDCl_3$.

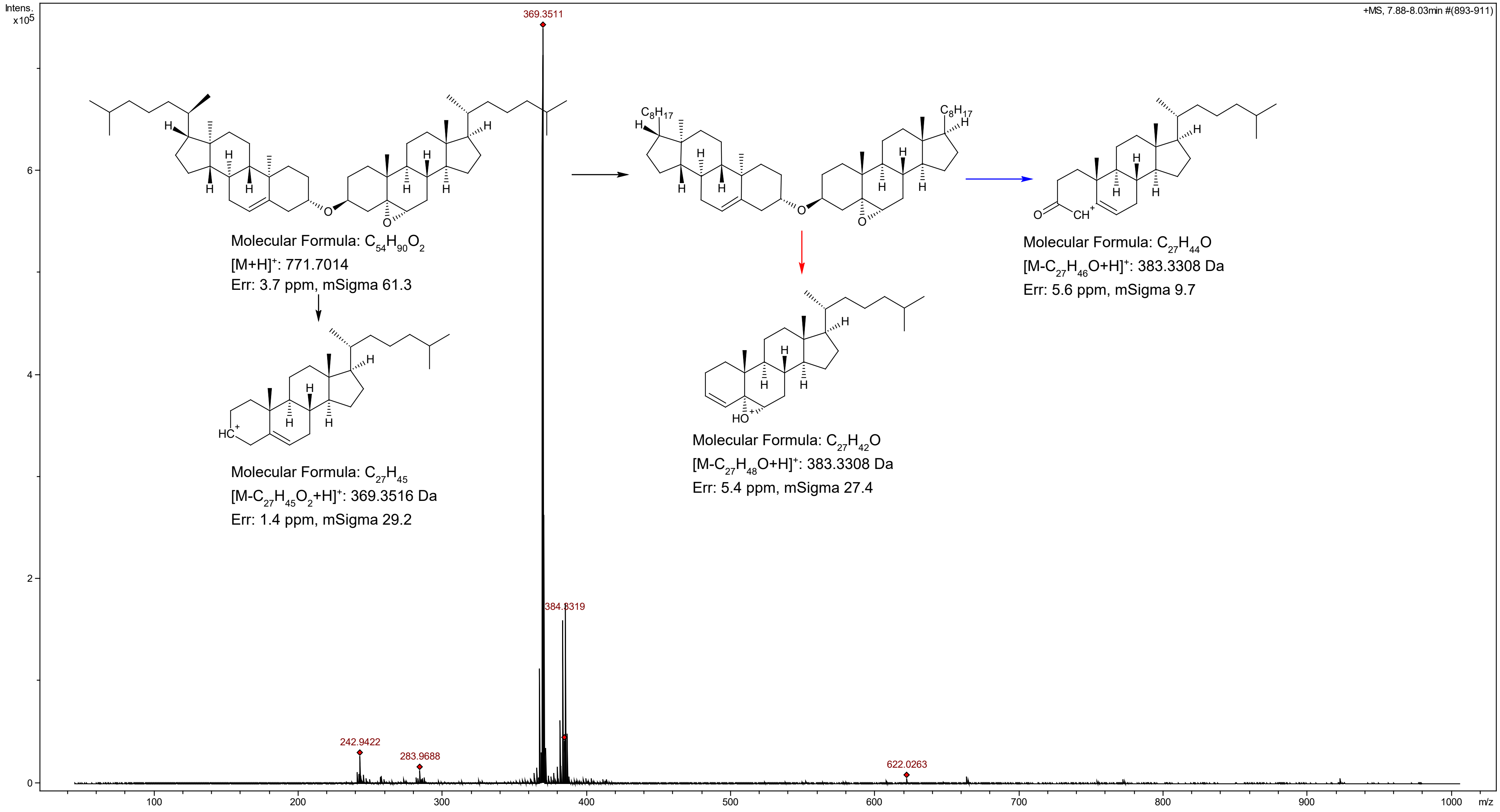

Figure S48. Mass spectrum of (5,6α-epoxycholesteryl)-cholesterol ether (**16**).

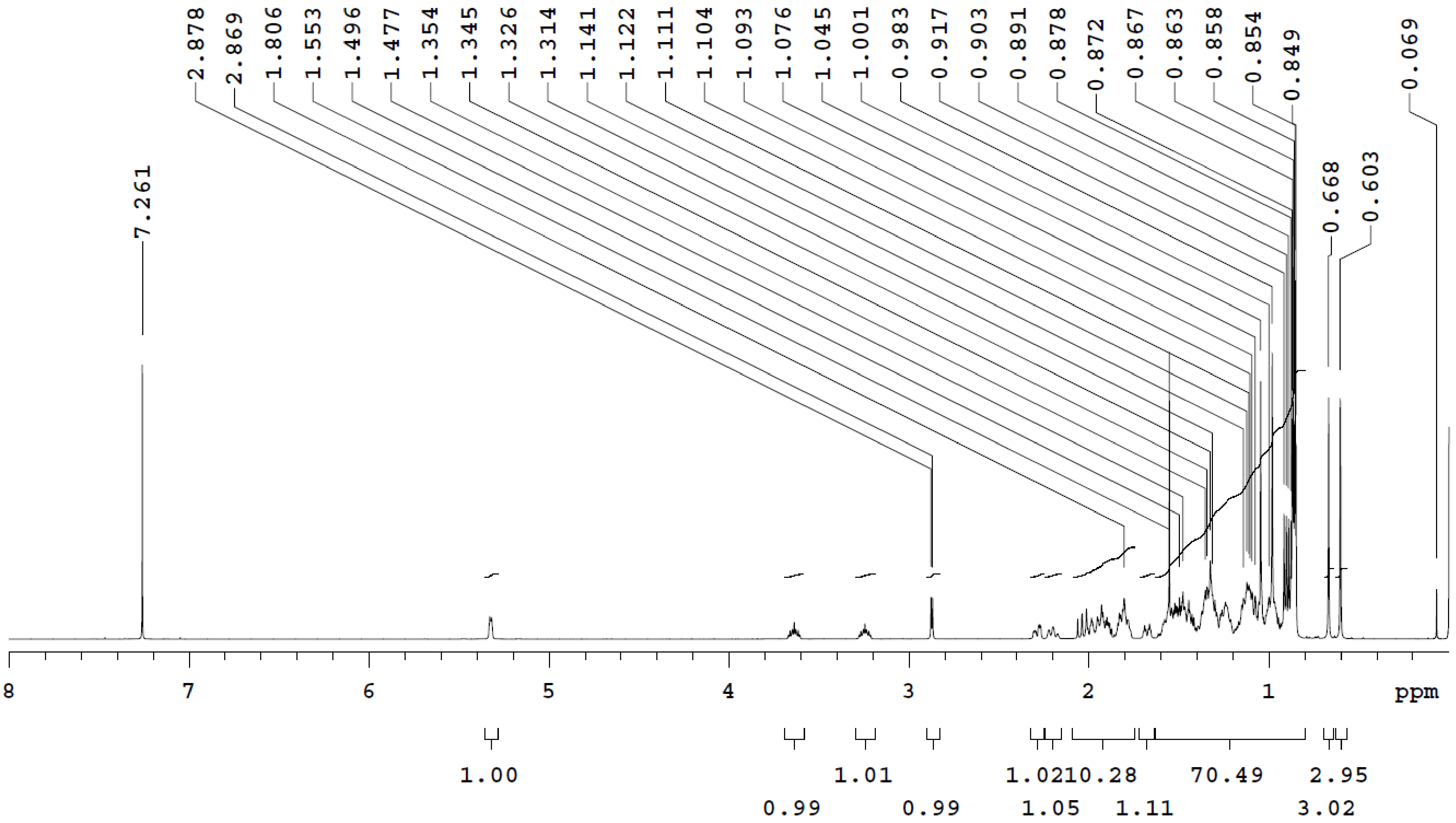

Figure S49. ^{1}H spectrum of (5,6α-epoxycholesteryl)-cholesterol ether (**16**) in $CDCl_3$.

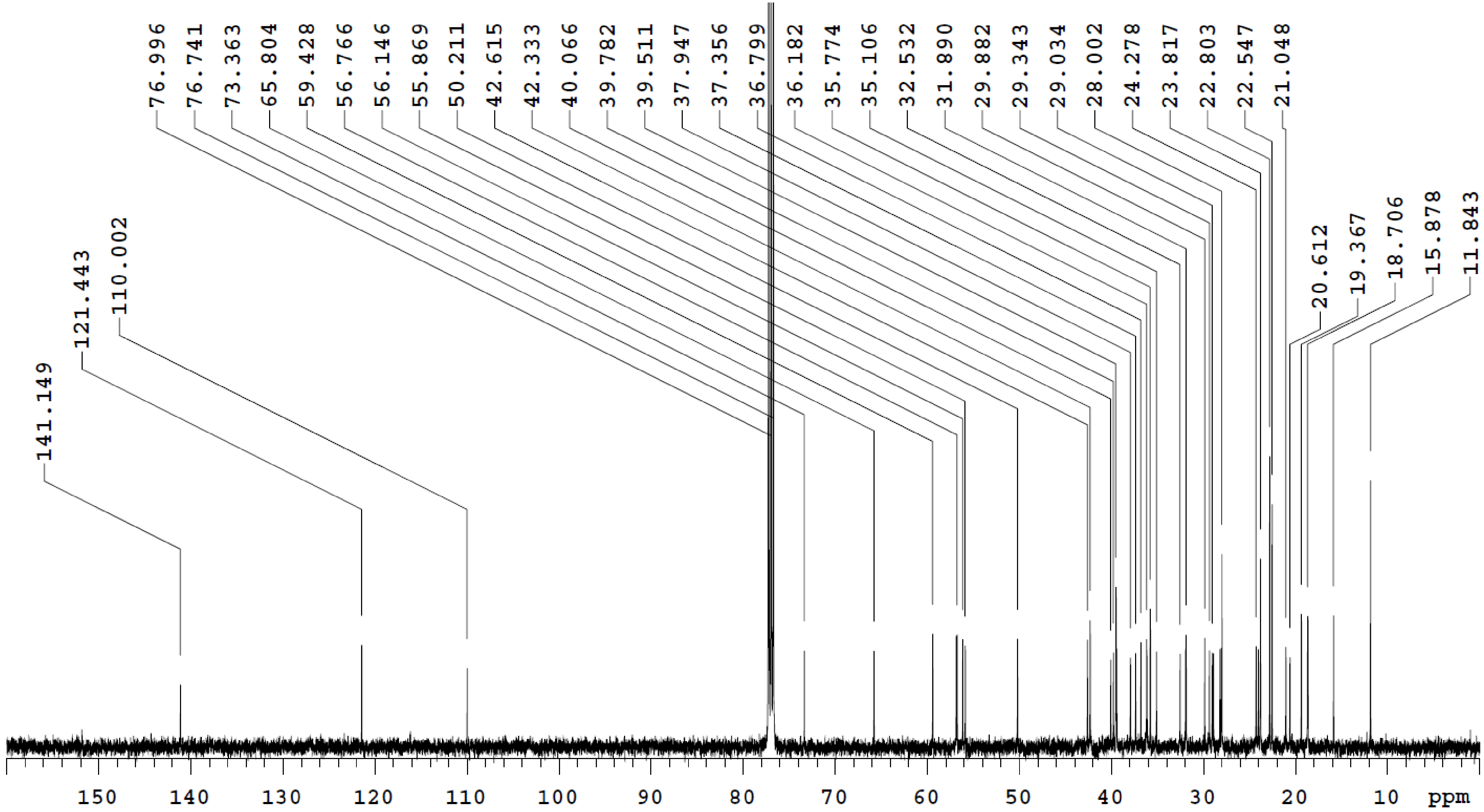

Figure S50. ^{13}C spectrum of (5,6α-epoxycholesteryl)-cholesterol ether (**16**) in $CDCl_3$.

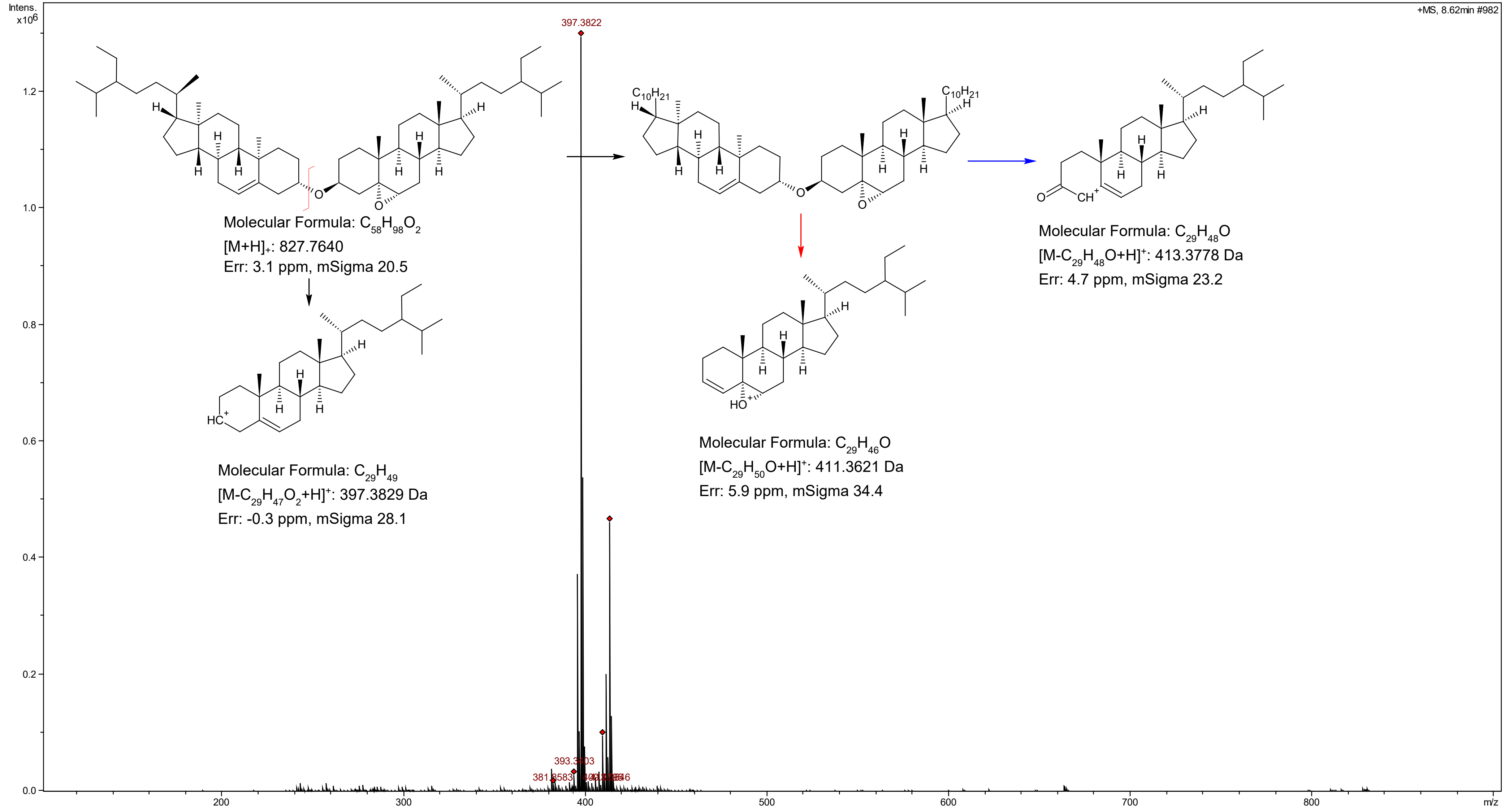

Figure S51. Mass spectrum of (5,6α-epoxysitosteryl)-sitosterol ether (**17**)

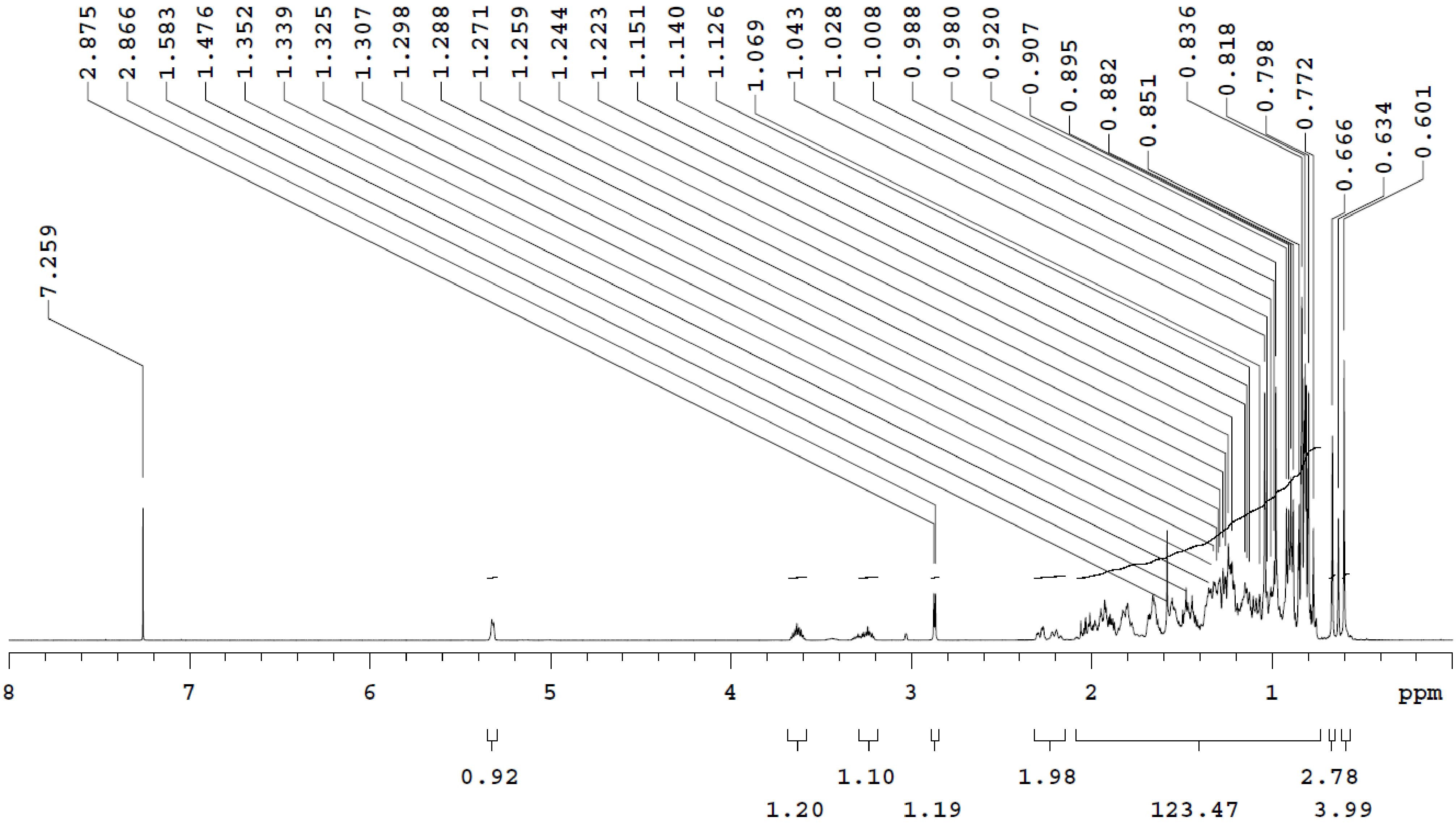

Figure S52. ^{1}H spectrum of (5,6α-epoxysitosteryl)-sitosterol ether (**17**) in $CDCl_3$.

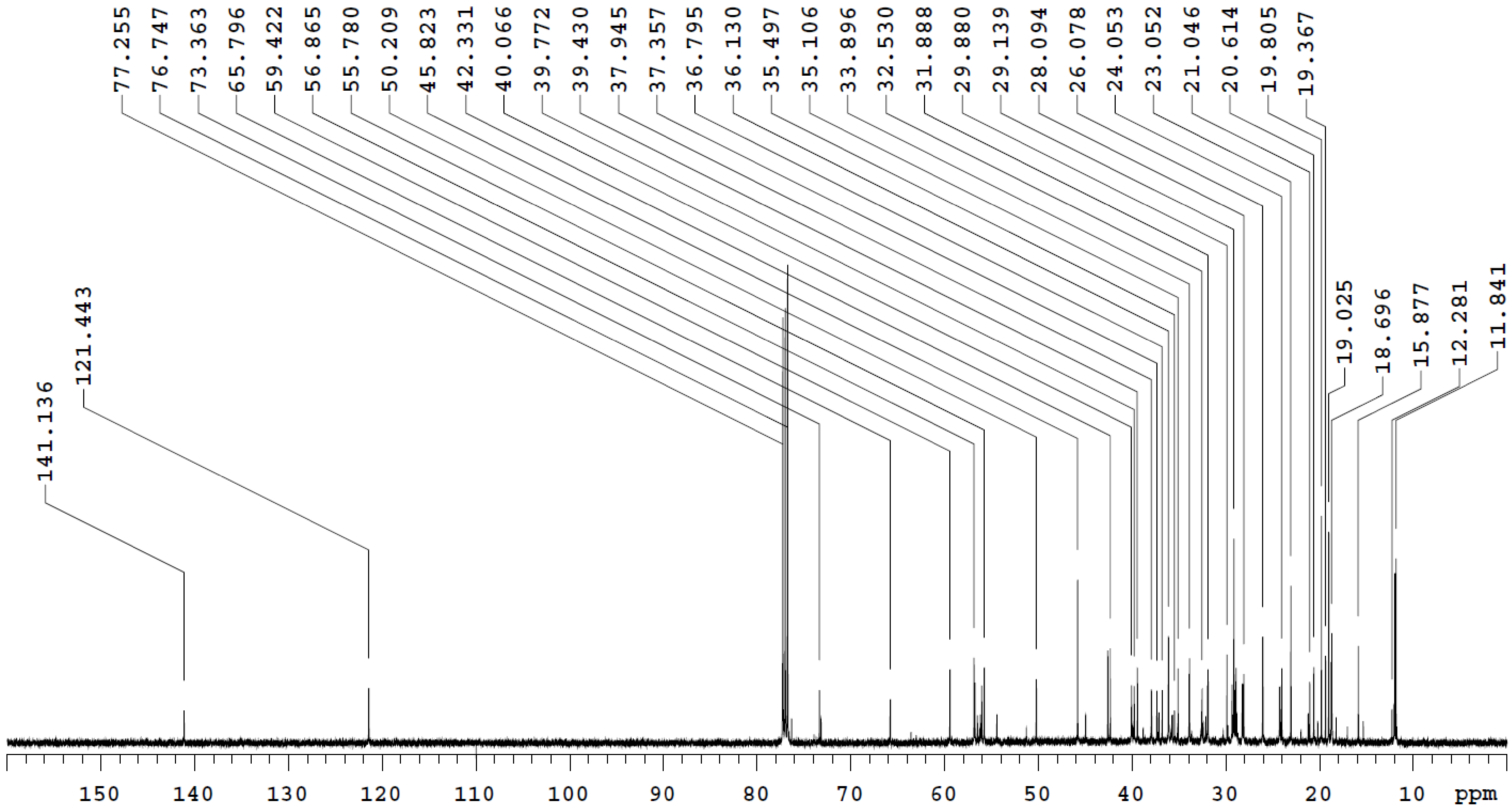

Figure S53. ^{13}C spectrum of (5,6α-epoxysitosteryl)-sitosterol ether (**17**) in $CDCl_3$.

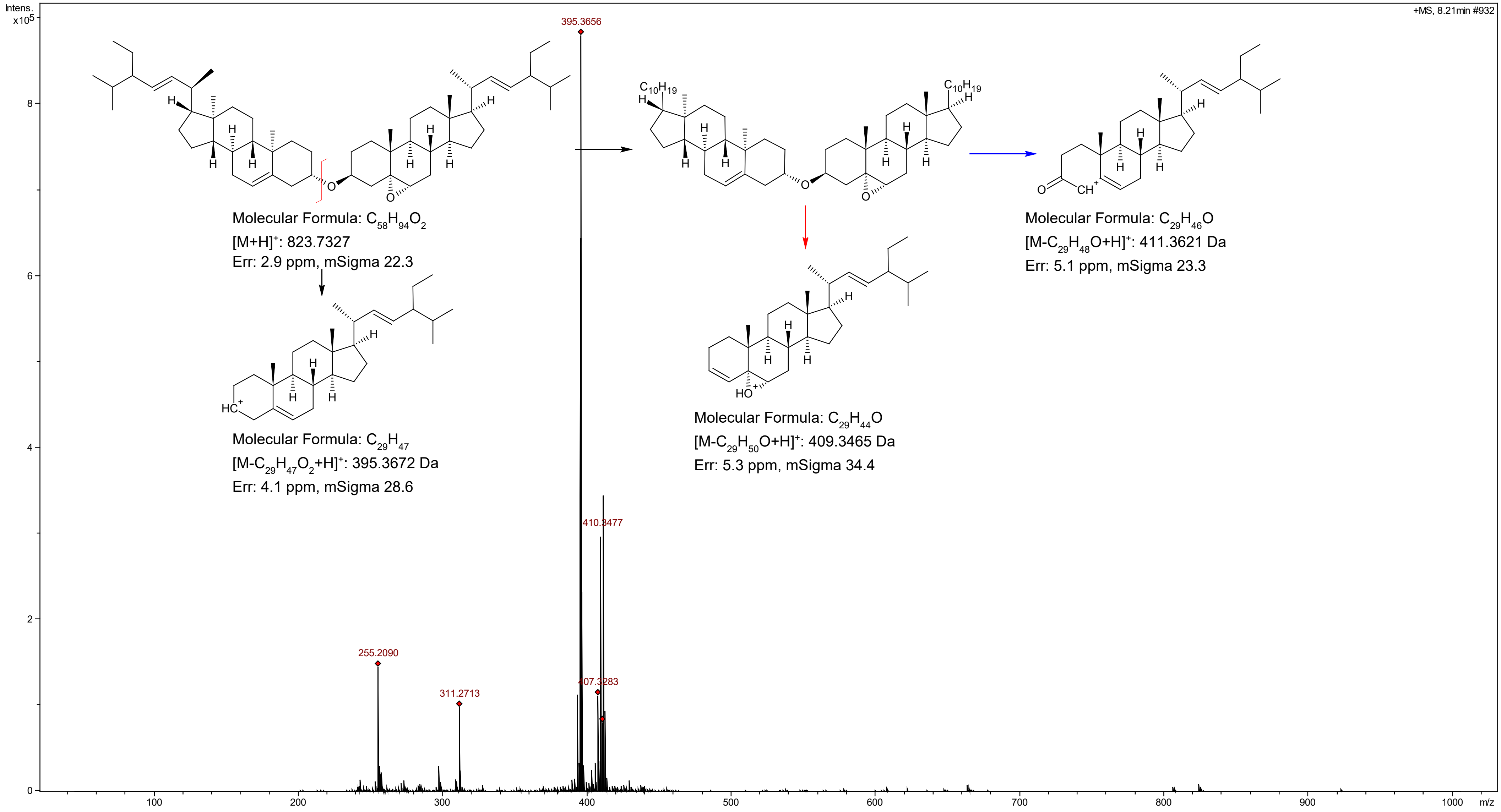

Figure S54. Mass spectrum of (5,6α-epoxysitgmasteryl)-stigmasterol ether (**18**).

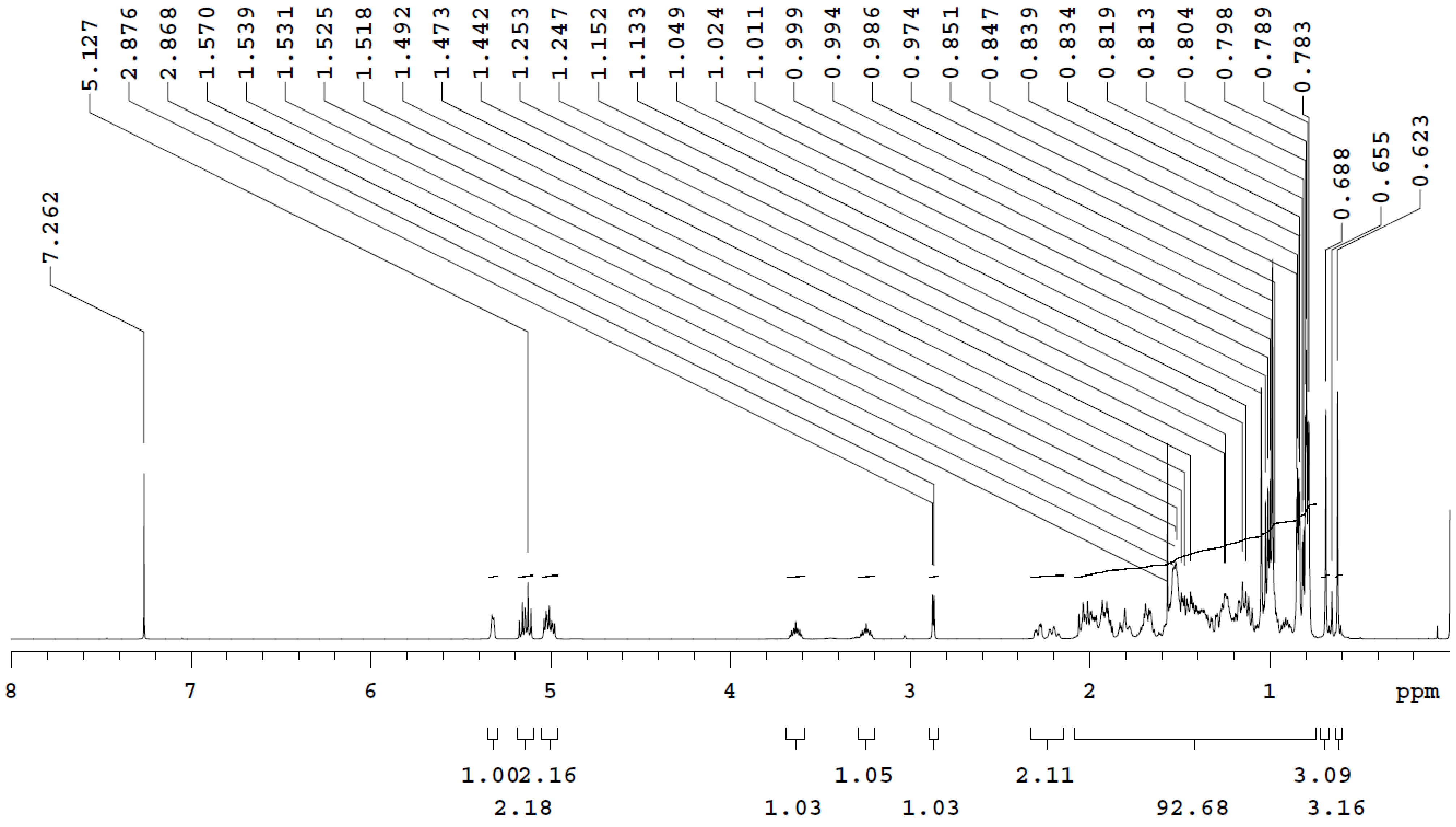

Figure S55. ^{1}H spectrum of (5,6α-epoxysitgmasteryl)-stigmasterol ether (**18**) in $CDCl_3$.

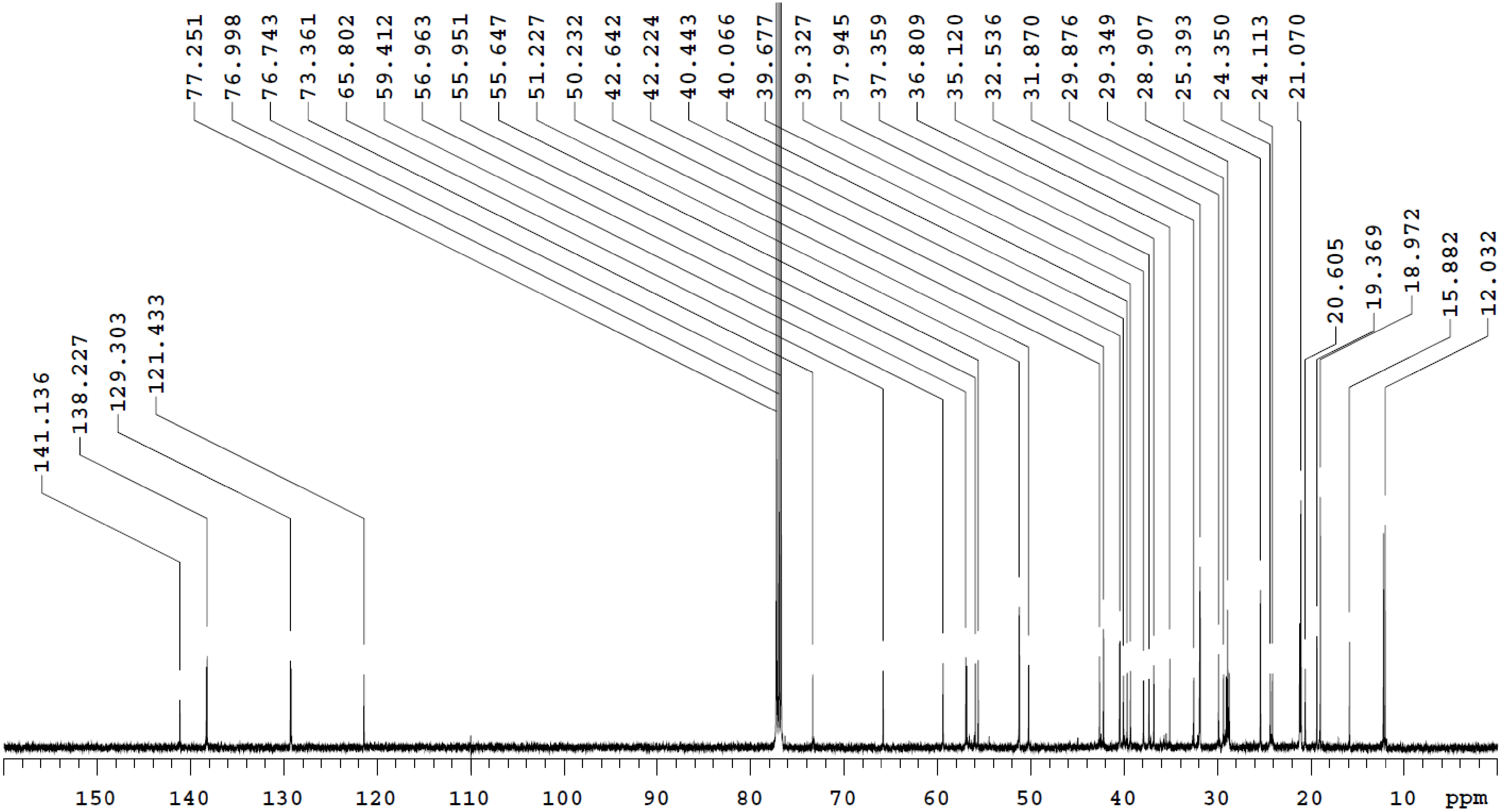

Figure S56. ^{13}C spectrum of (5,6α-epoxysitgmasteryl)-stigmasterol ether (**18**) in $CDCl_3$.

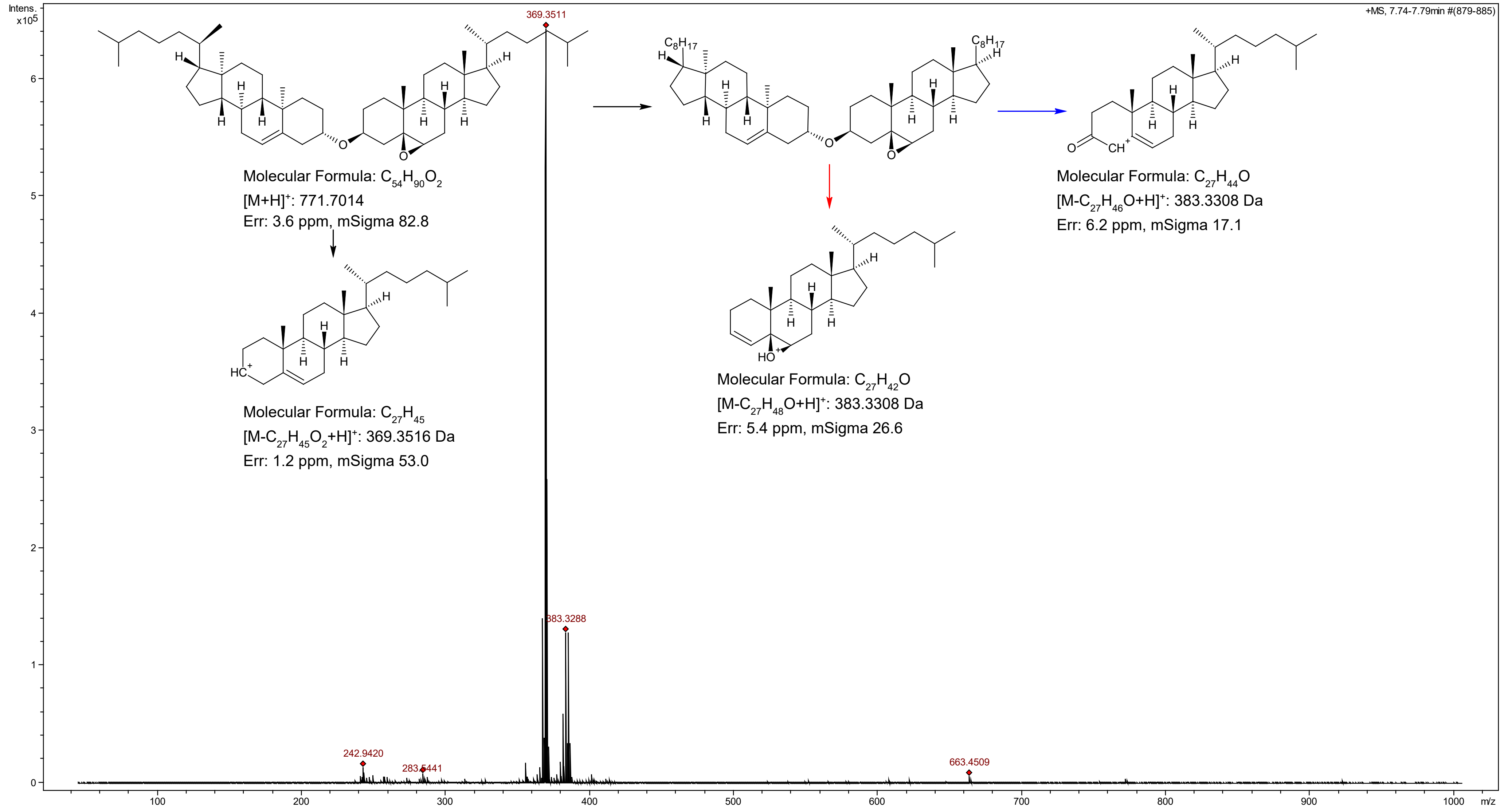

Figure S57. Mass spectrum of (5,6β-epoxycholesteryl)-cholesterol ether (**19**).

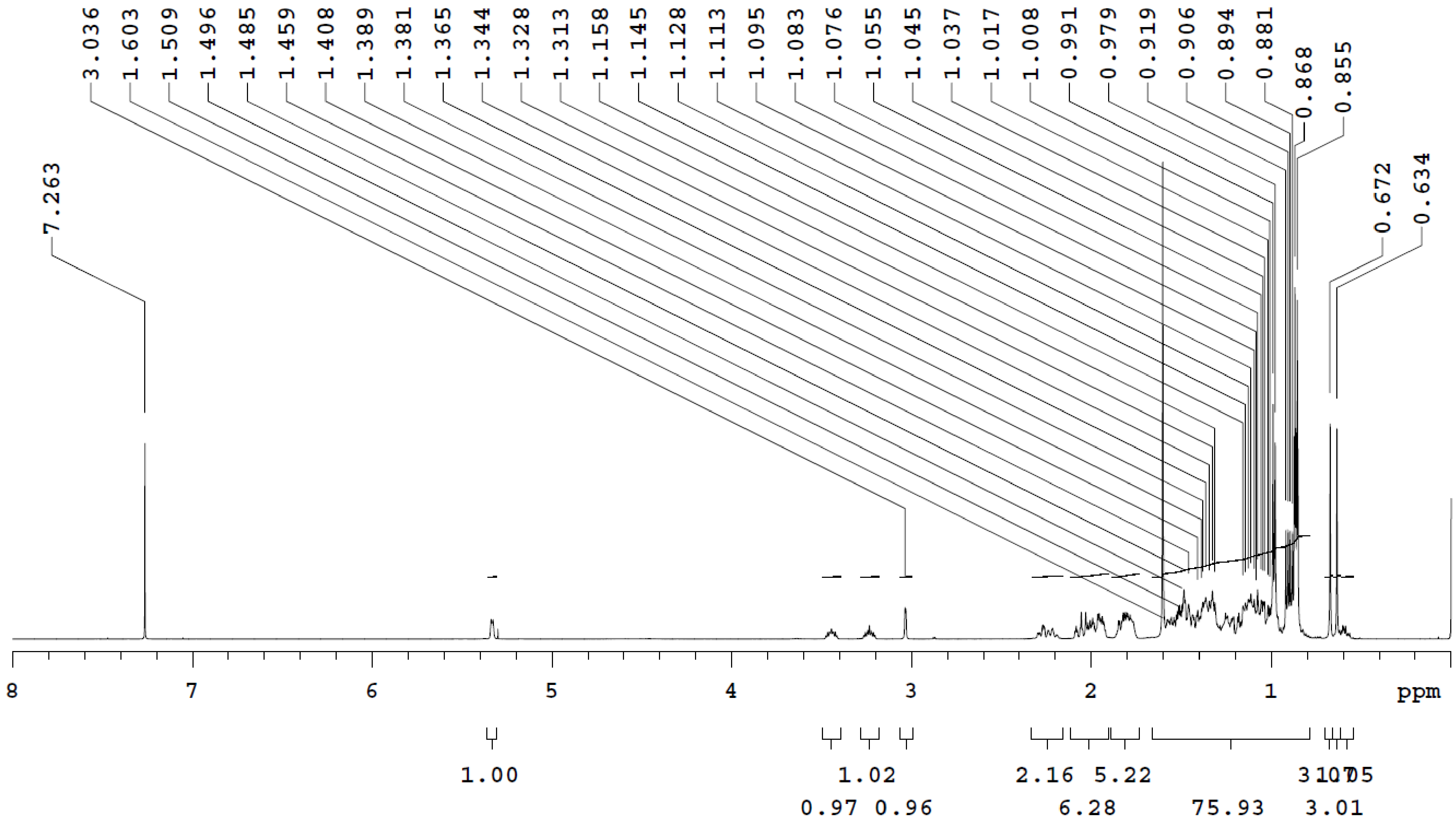

Figure S58. ^{1}H spectrum of (5,6β-epoxycholesteryl)-cholesterol ether (**19**) in $CDCl_3$.

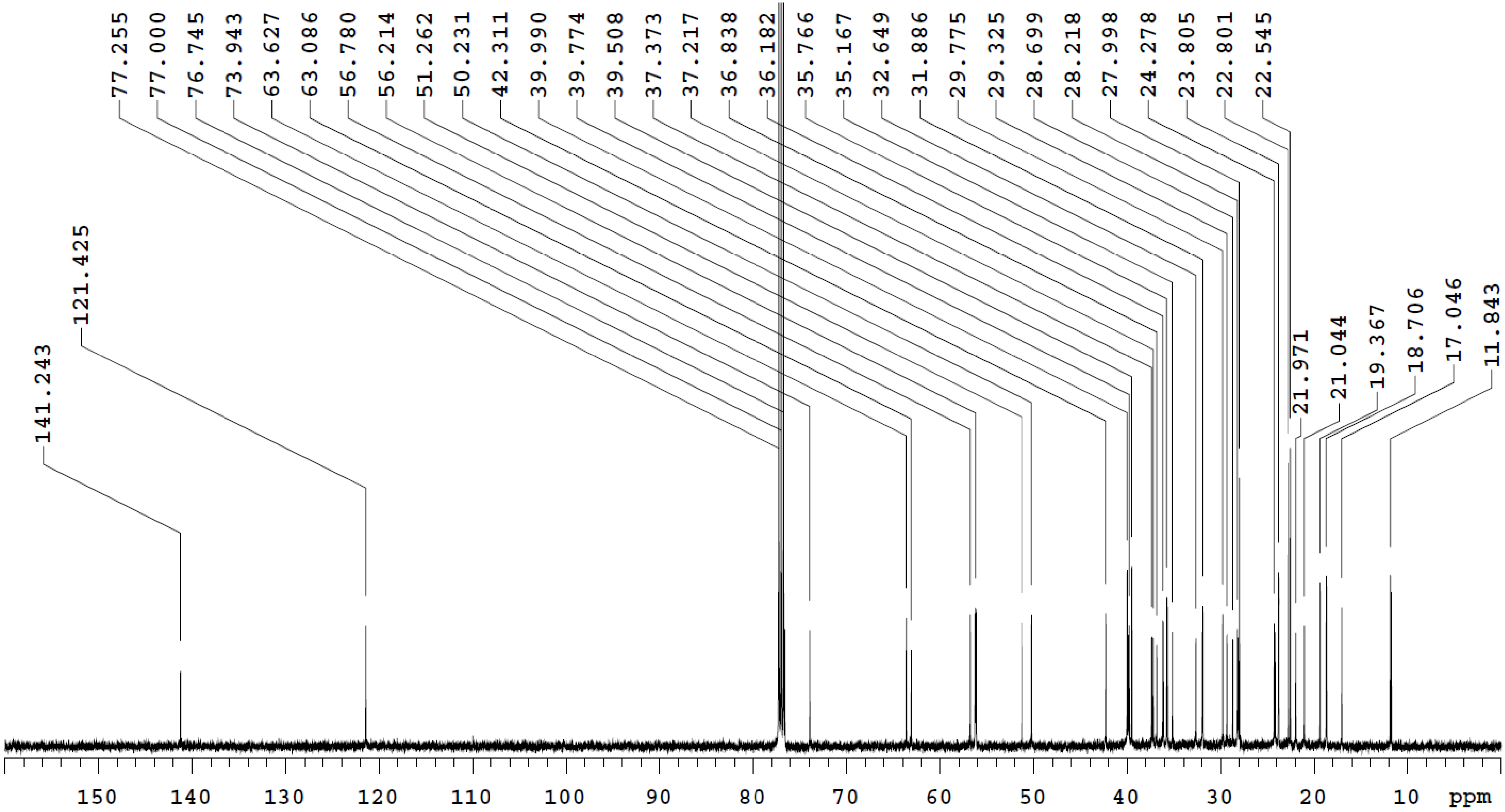

Figure S59. ^{13}C spectrum of (5,6β-epoxycholesteryl)-cholesterol ether (**19**) in $CDCl_3$.

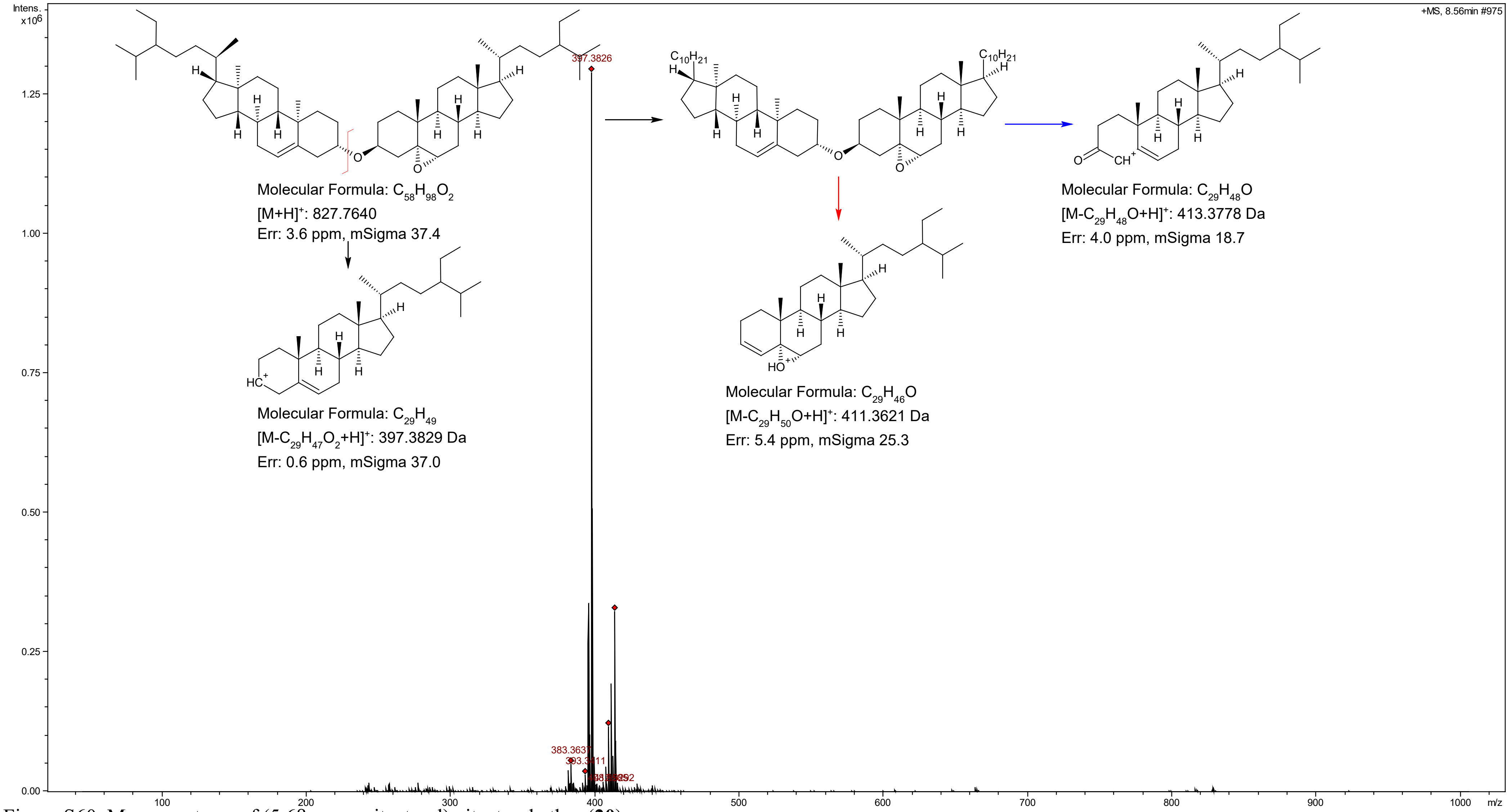

Figure S60. Mass spectrum of (5,6β-epoxysitosteryl)-sitosterol ether (**20**).

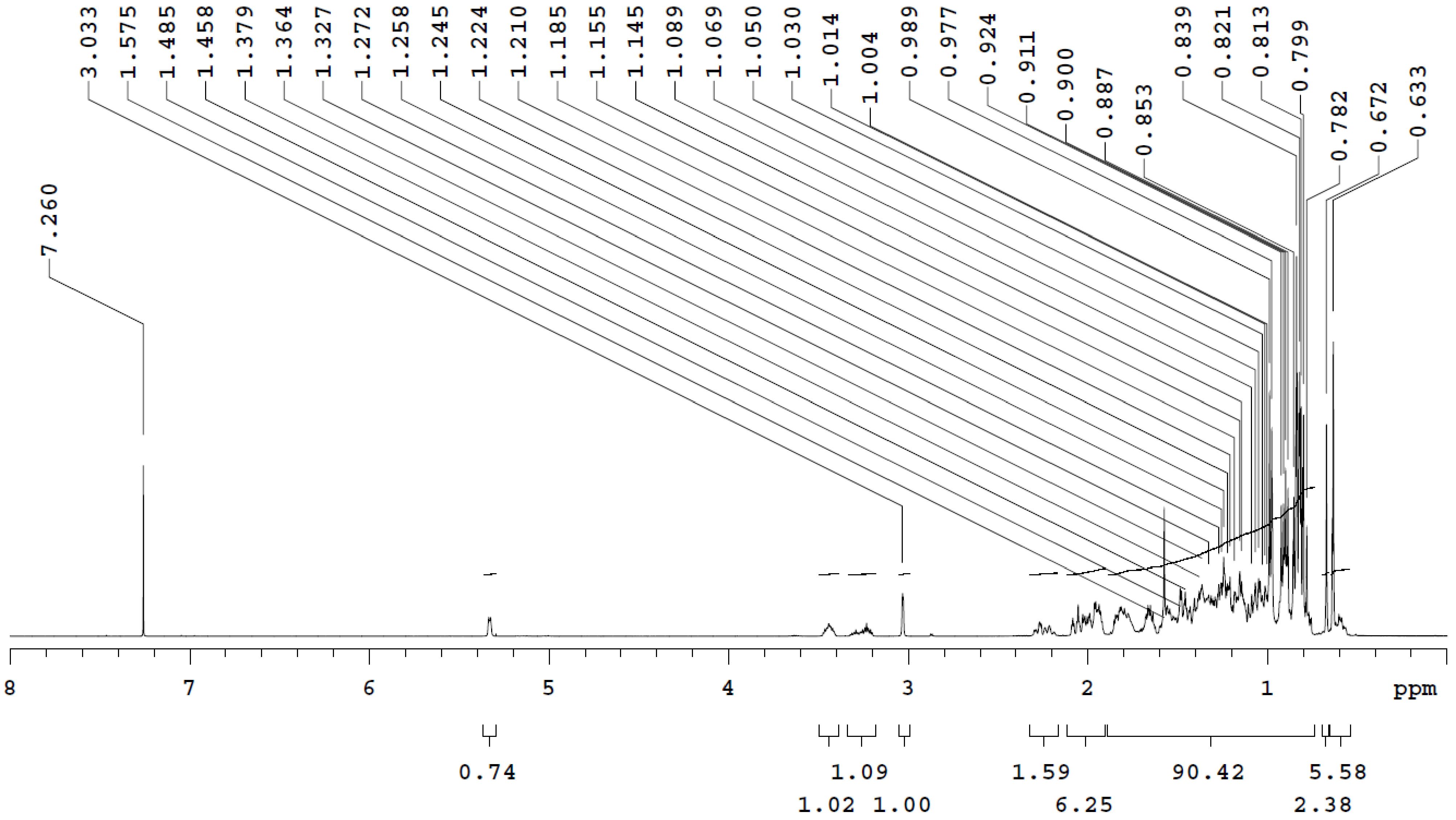

Figure S61. ^{1}H spectrum of (5,6β-epoxysitosteryl)-sitosterol ether (**20**) in $CDCl_3$.

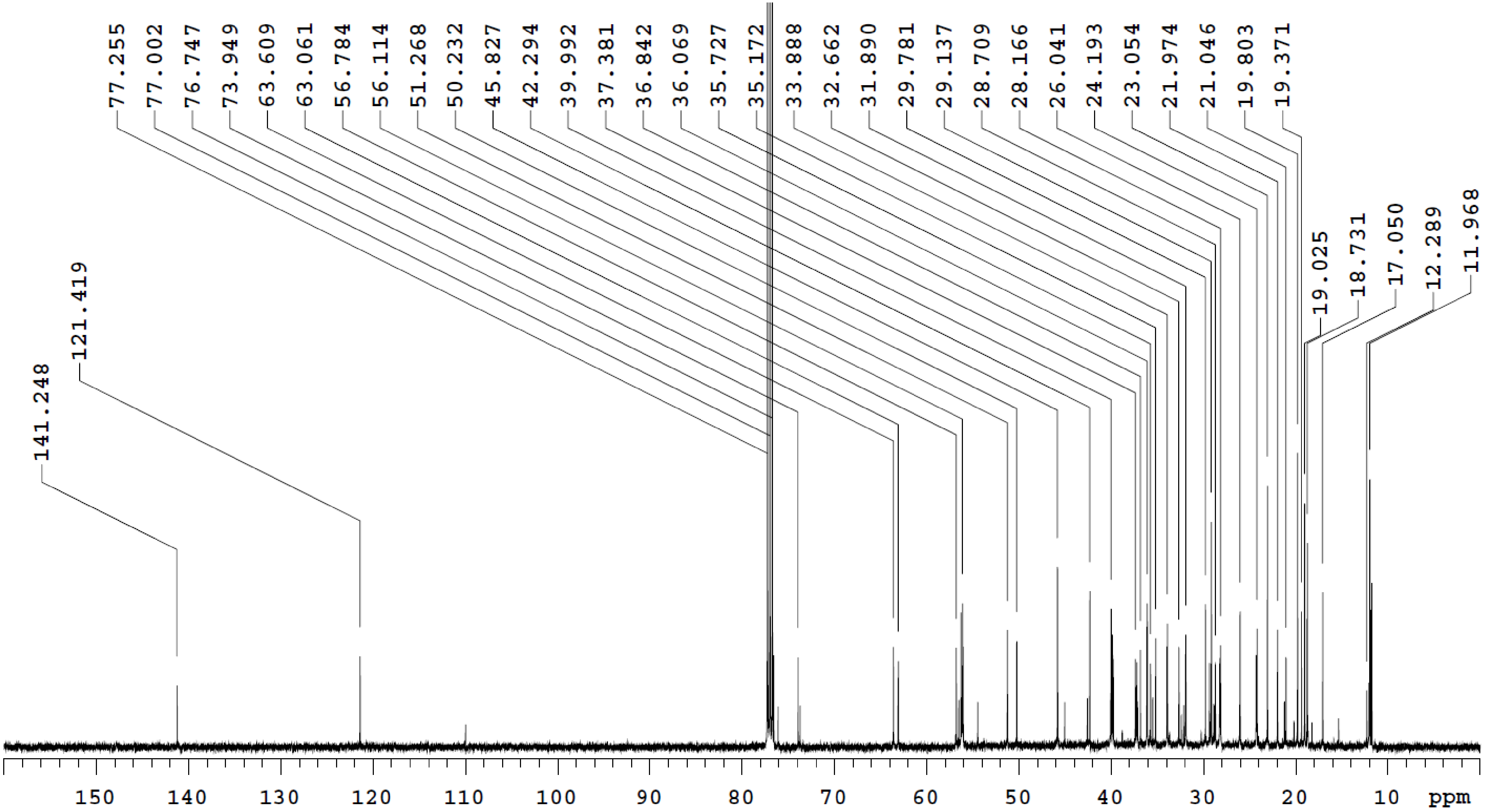

Figure S62. ^{13}C spectrum of (5,6β-epoxysitosteryl)-sitosterol ether (**20**) in $CDCl_3$.

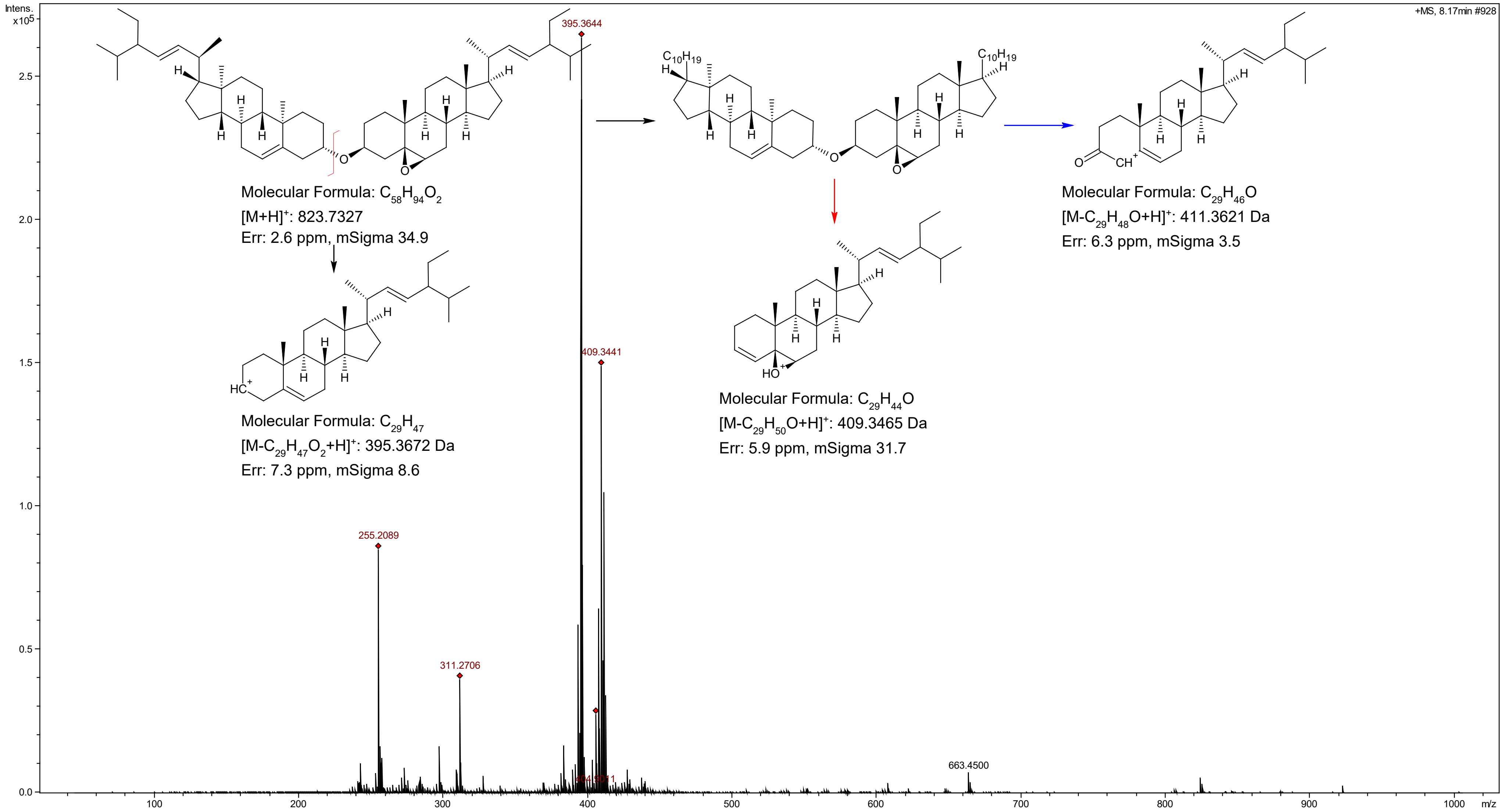

Figure S63. Mass spectrum of (5,6β-epoxystigmasteryl)-stigmasterol ether (**21**).

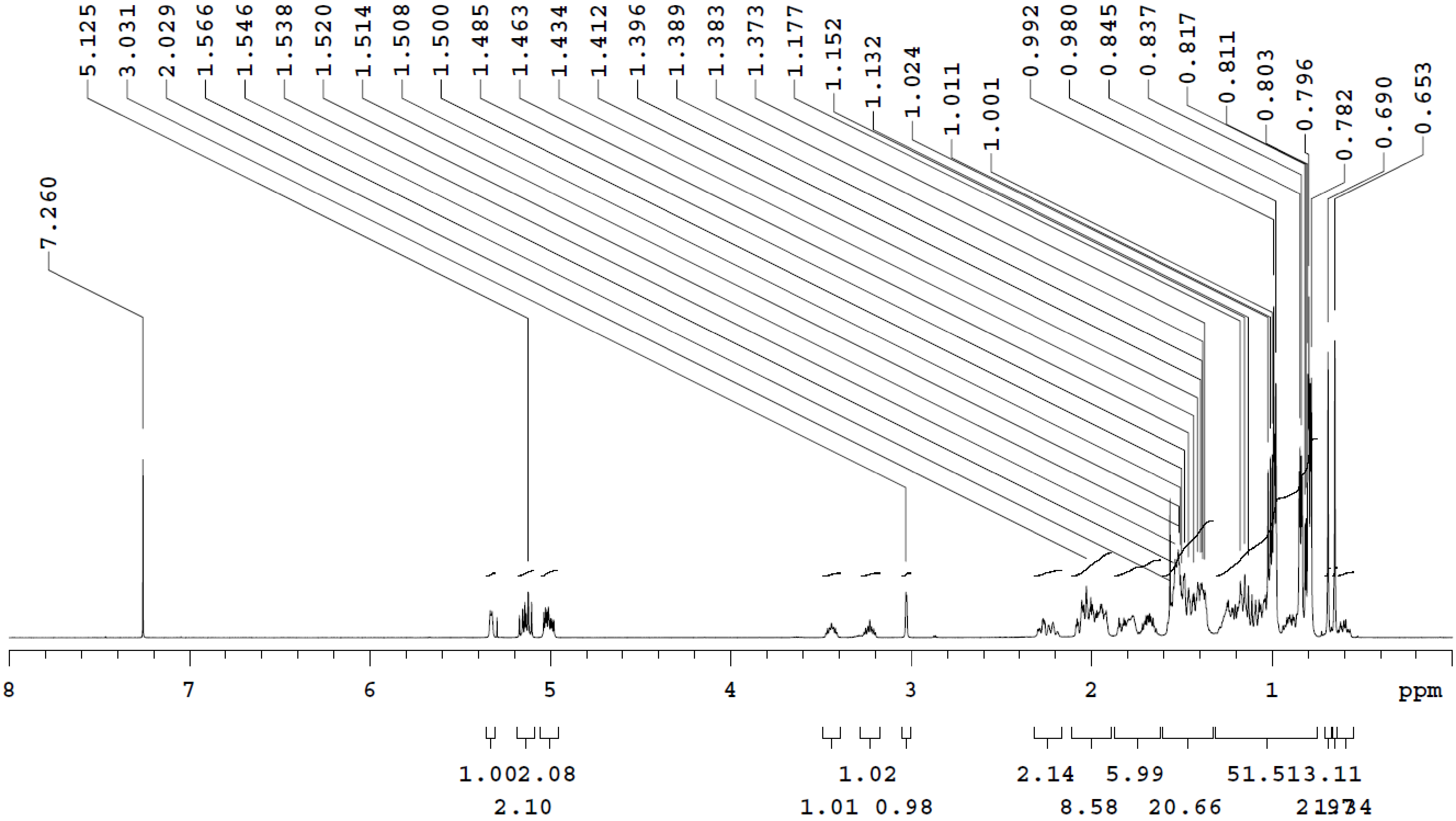

Figure S64. ^{1}H spectrum of (5,6β-epoxystigmasteryl)-stigmasterol ether (**21**) in $CDCl_3$.

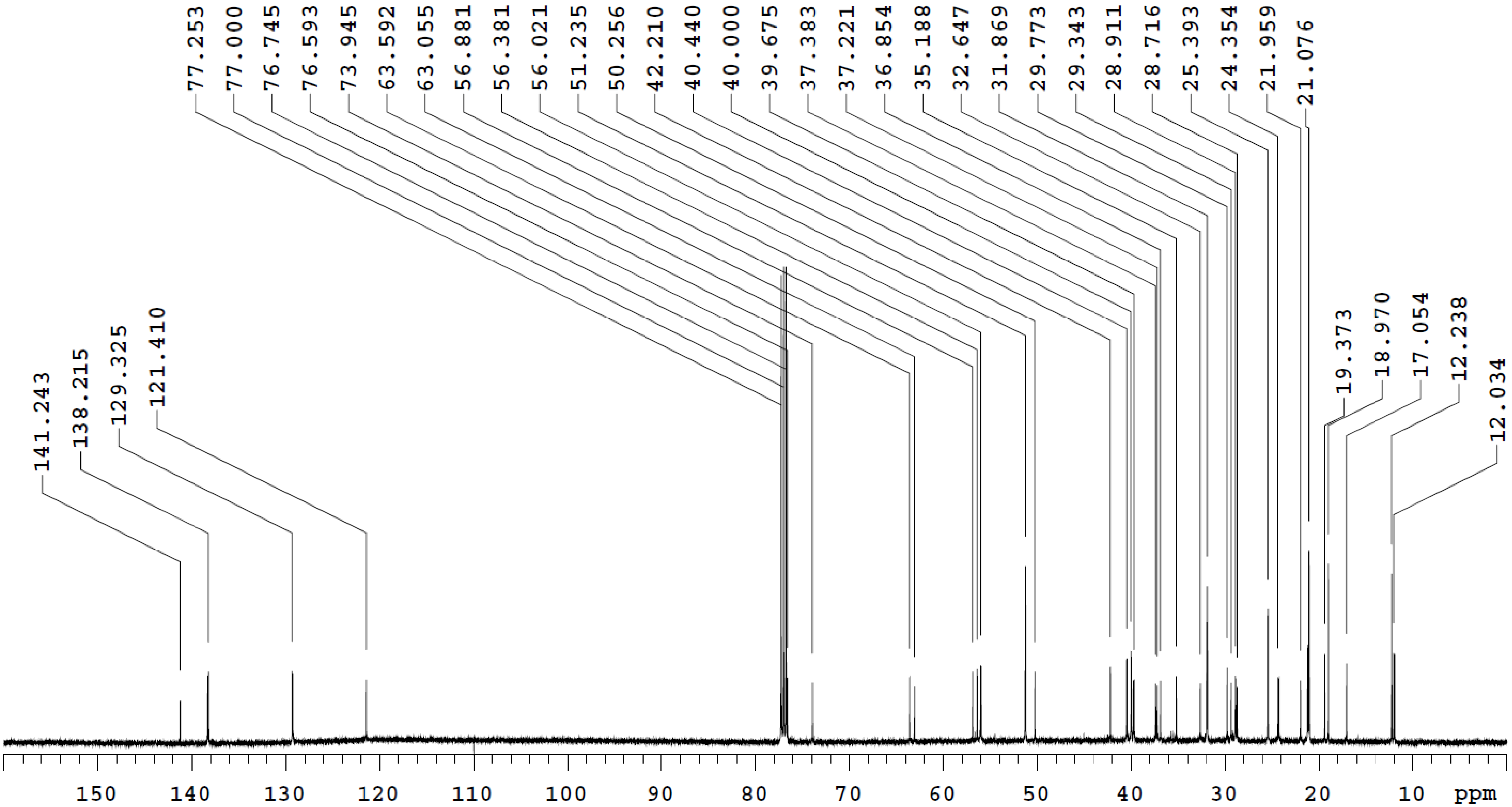

Figure S65. ^{13}C spectrum of (5,6β-epoxystigmasteryl)-stigmasterol ether (**21**) in $CDCl_3$.

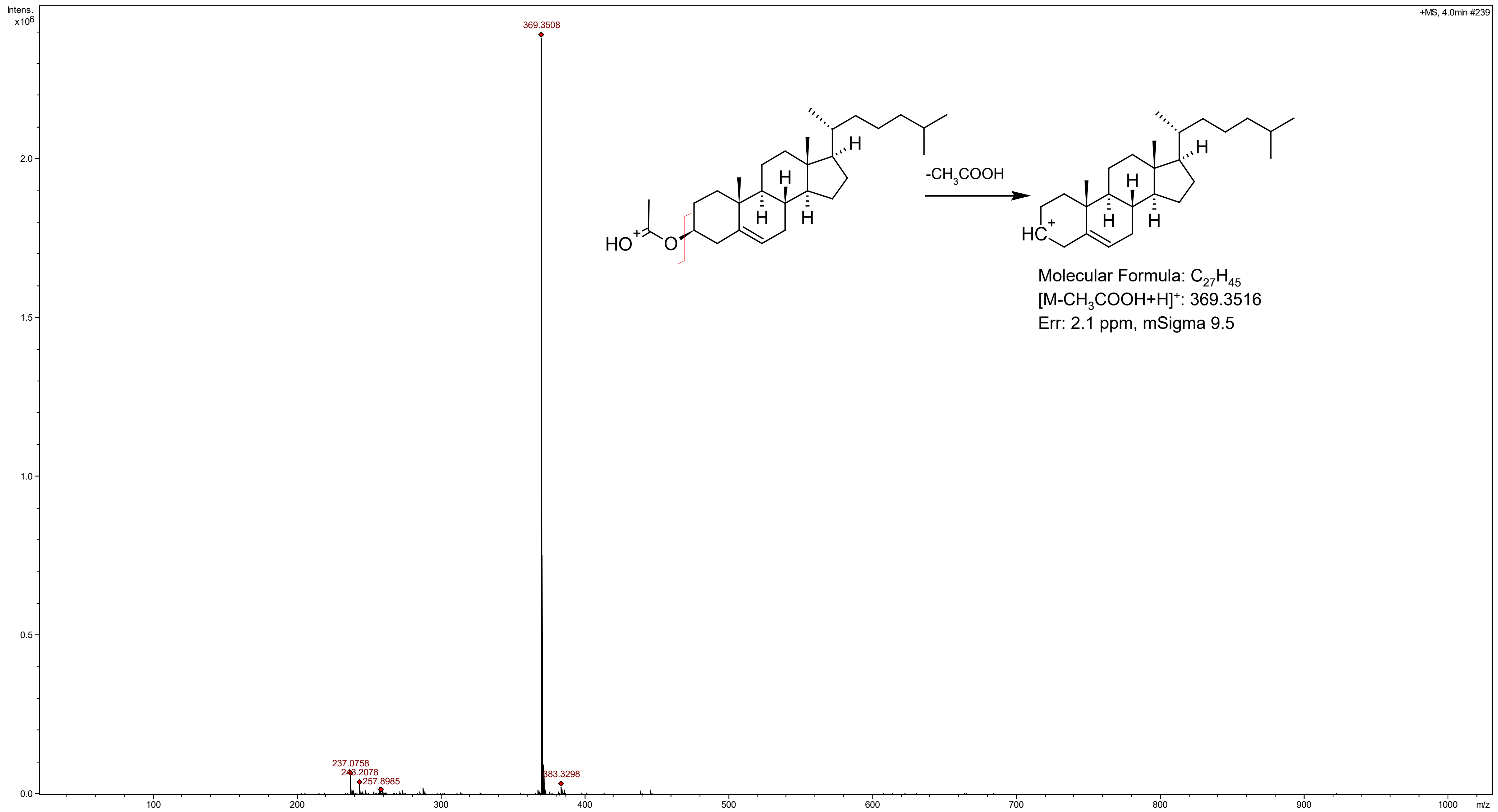

Figure S66. Mass spectrum of cholest-5-en-3β-yl acetate (Cholesteryl acetate) (**22**)

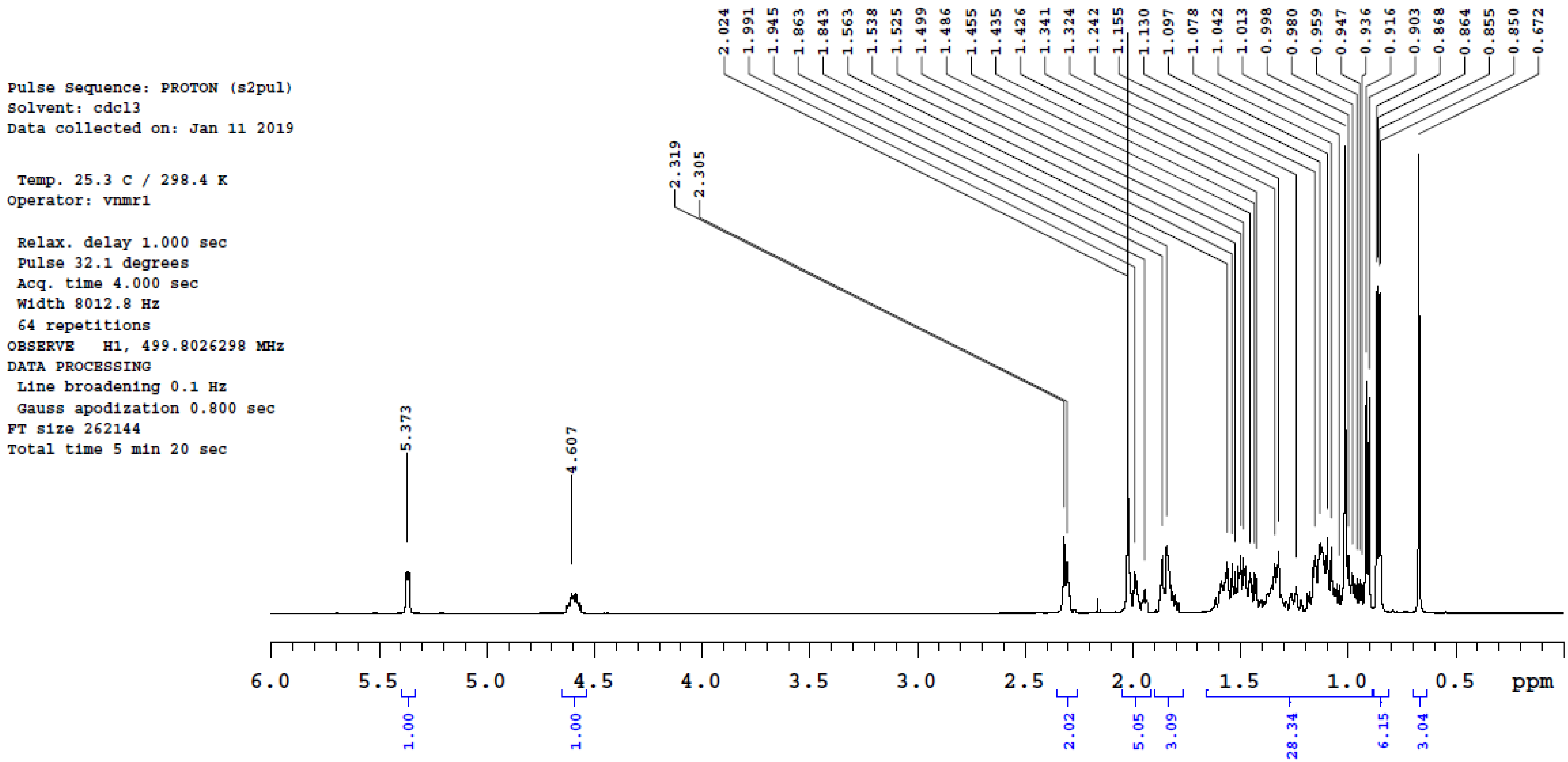

Figure S67. ^{1}H spectrum of cholest-5-en-3β-yl acetate (Cholesteryl acetate) (**22**) in $CDCl_3$

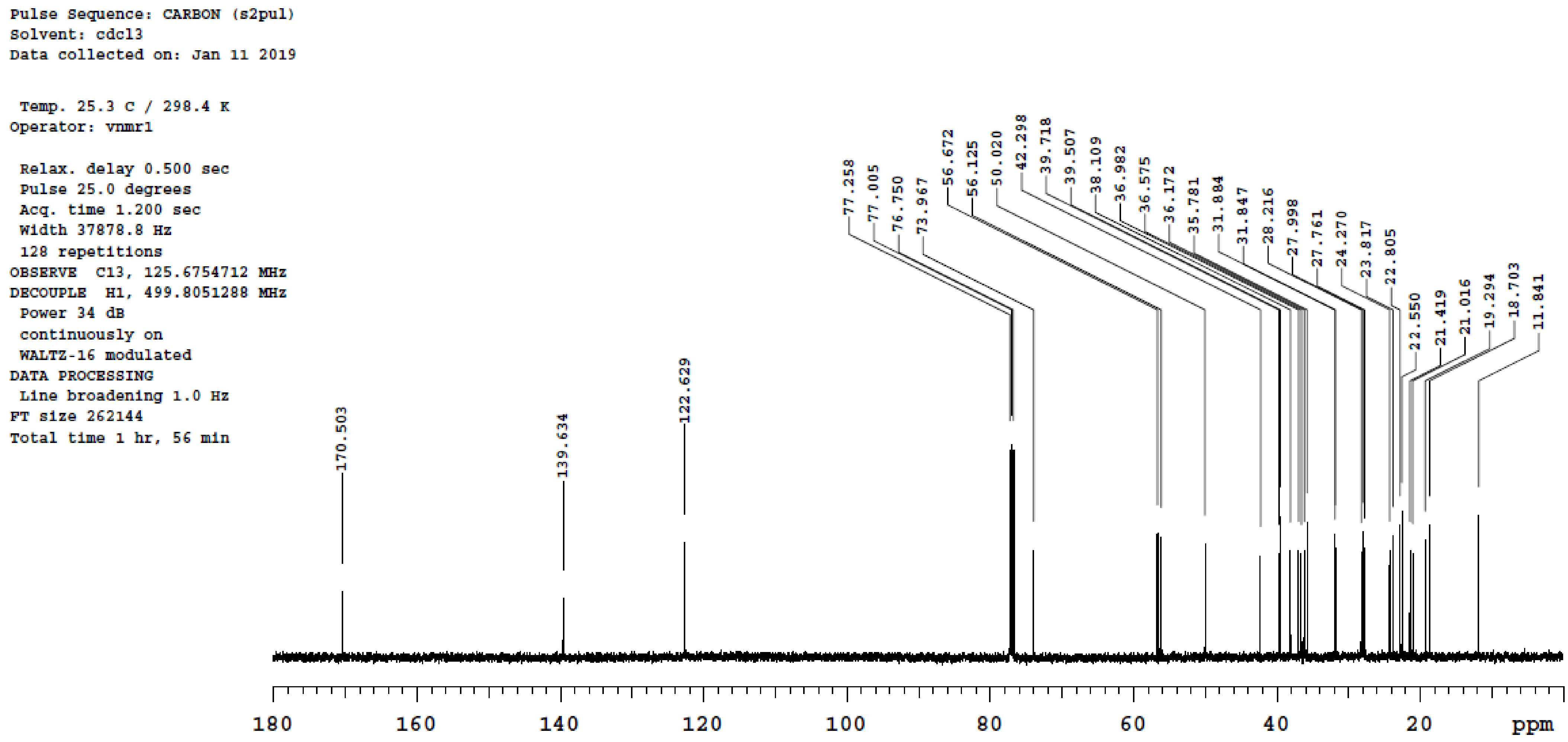

Figure S68. ^{13}C spectrum of cholest-5-en-3β-yl acetate (Cholesteryl acetate) (**22**) in $CDCl_3$

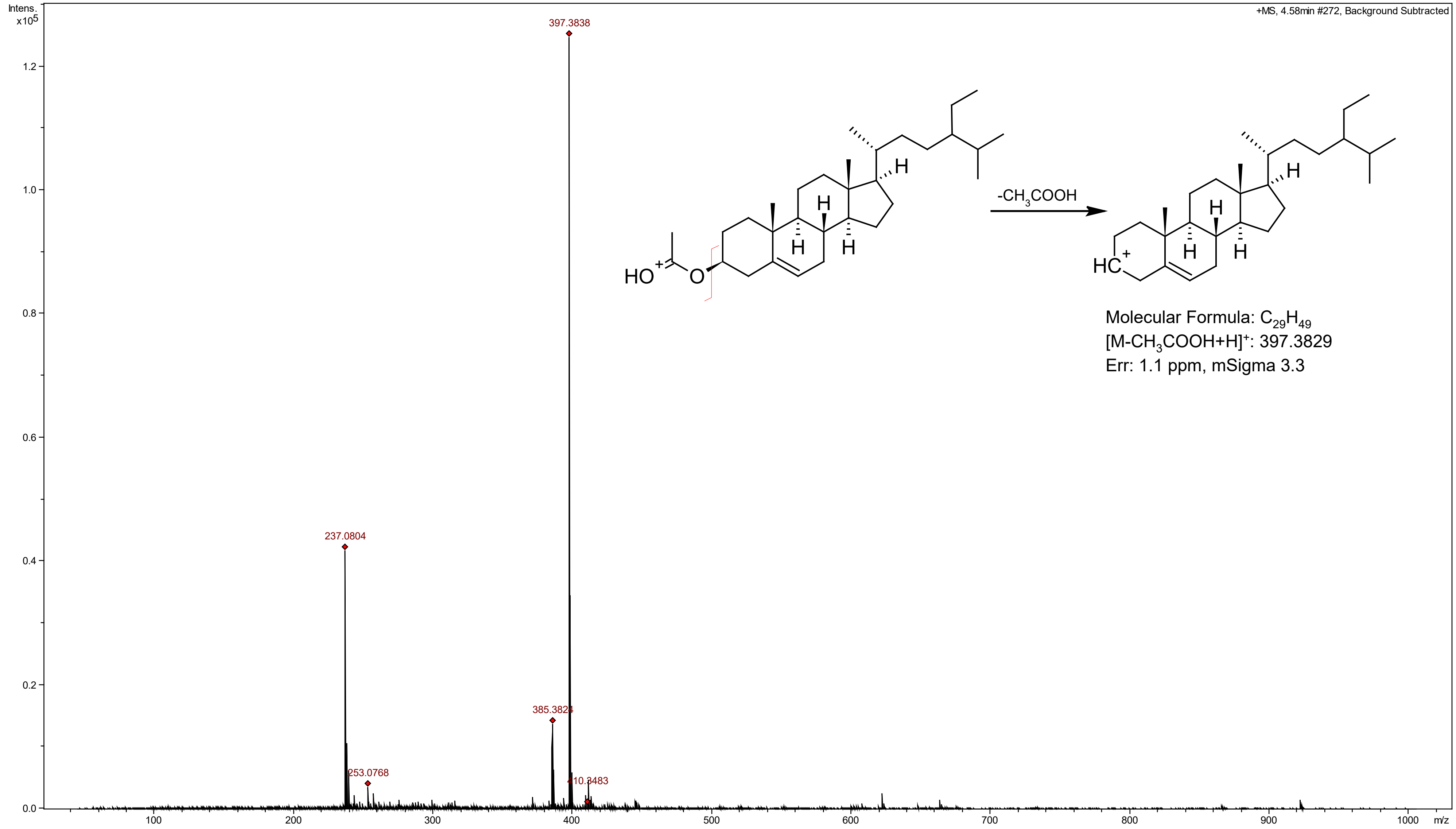

Figure S69. Mass spectrum of sitost-5-en-3β-yl acetate (β-Sitosteryl acetate) (**23**)

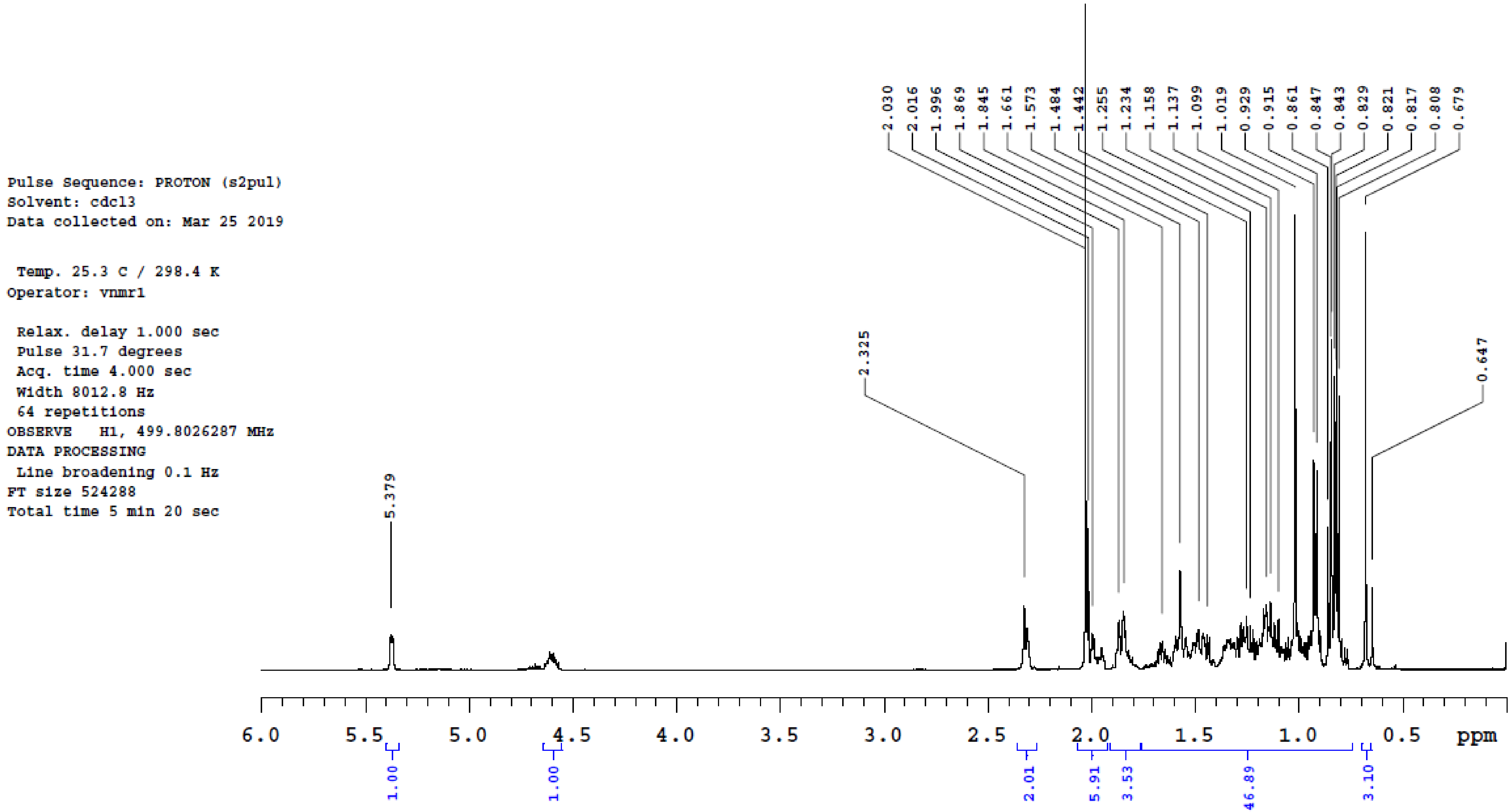

Figure S70. ^{1}H spectrum of sitost-5-en-3β-yl acetate (β-Sitosteryl acetate) (**23**) in $CDCl_3$

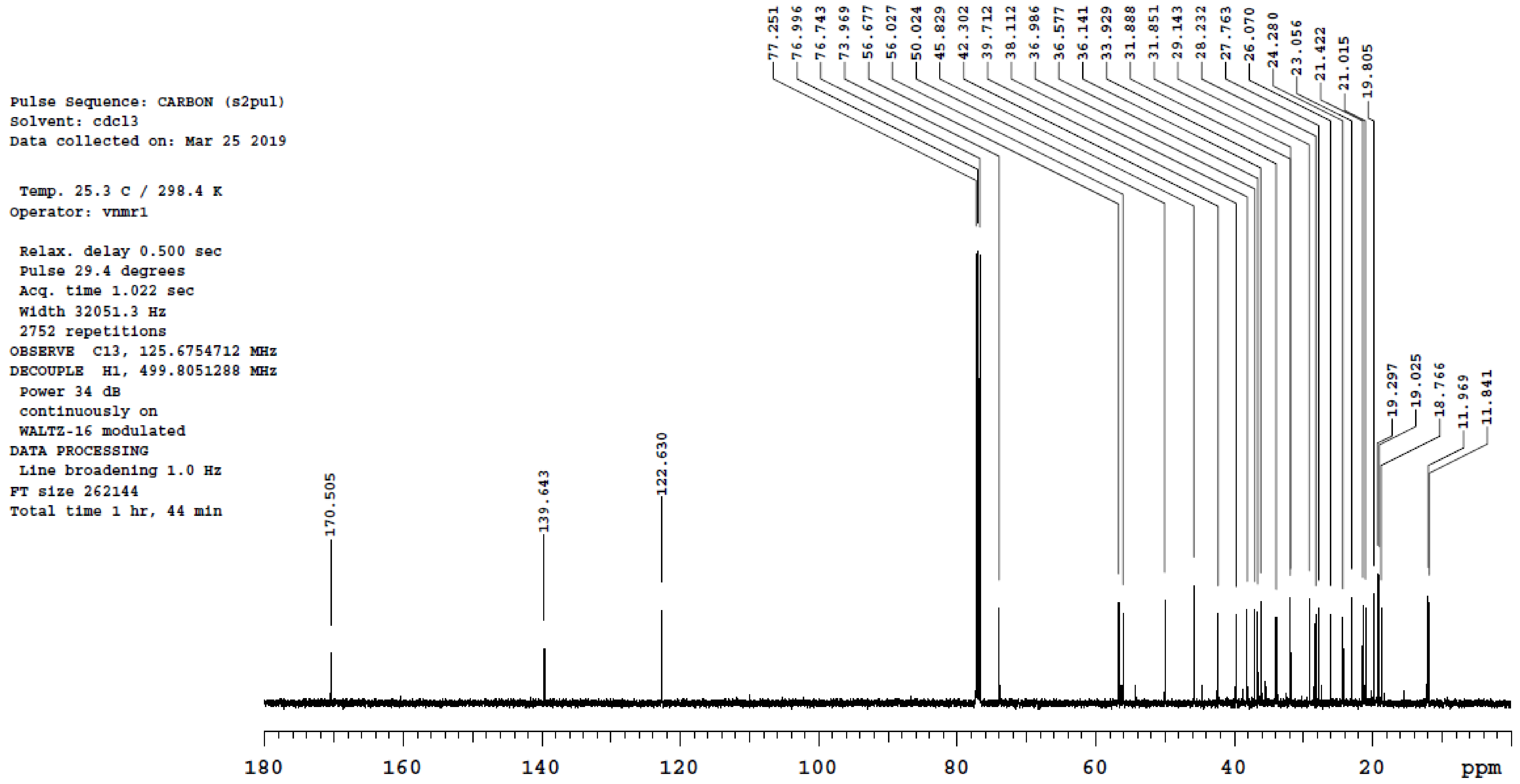

Figure S71. ^{13}C spectrum of sitost-5-en-3β-yl acetate (β-Sitosteryl acetate) (**23**) in $CDCl_3$

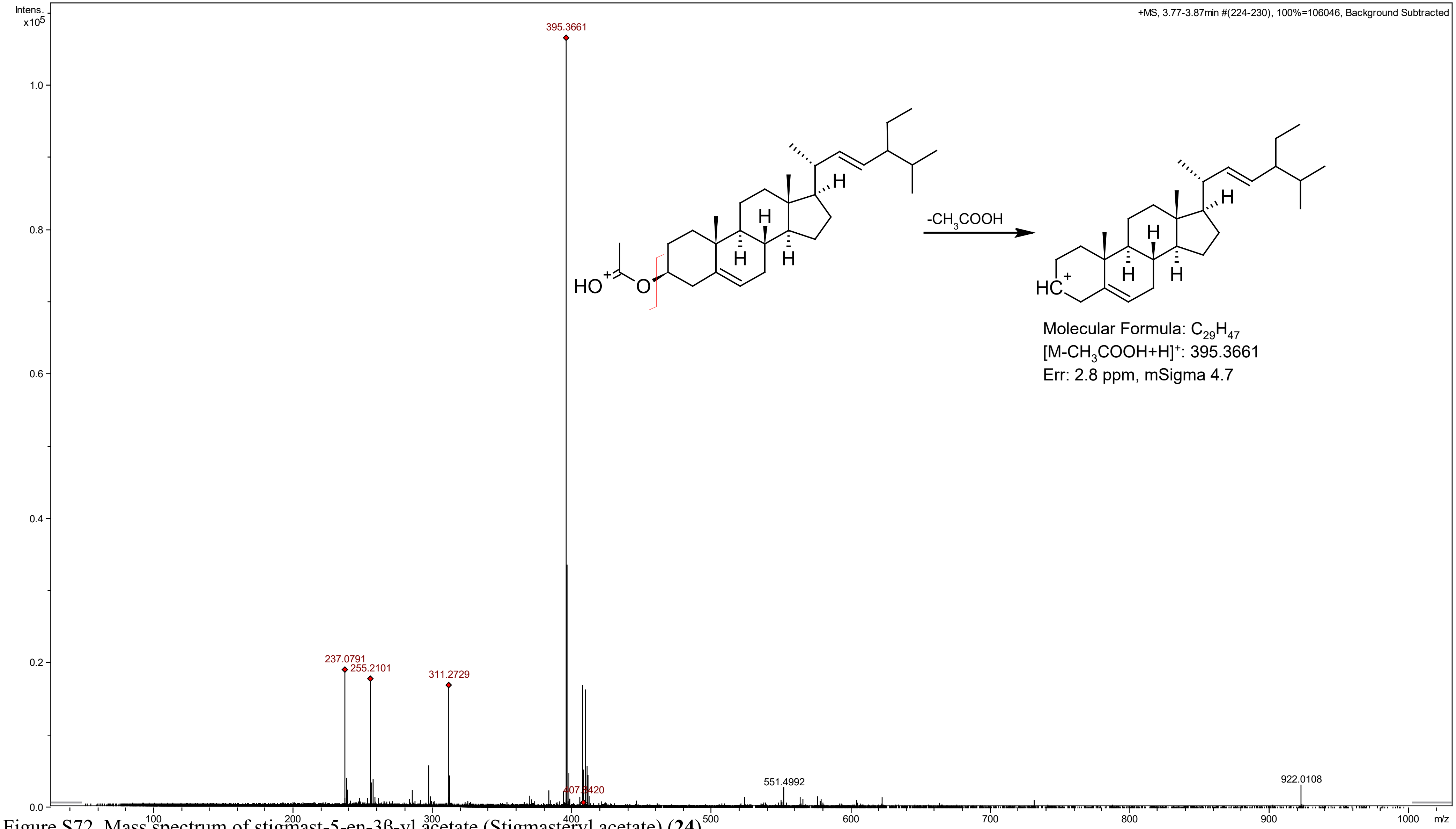

Figure S72. Mass spectrum of stigmast-5-en-3β-yl acetate (Stigmasteryl acetate) (**24**)

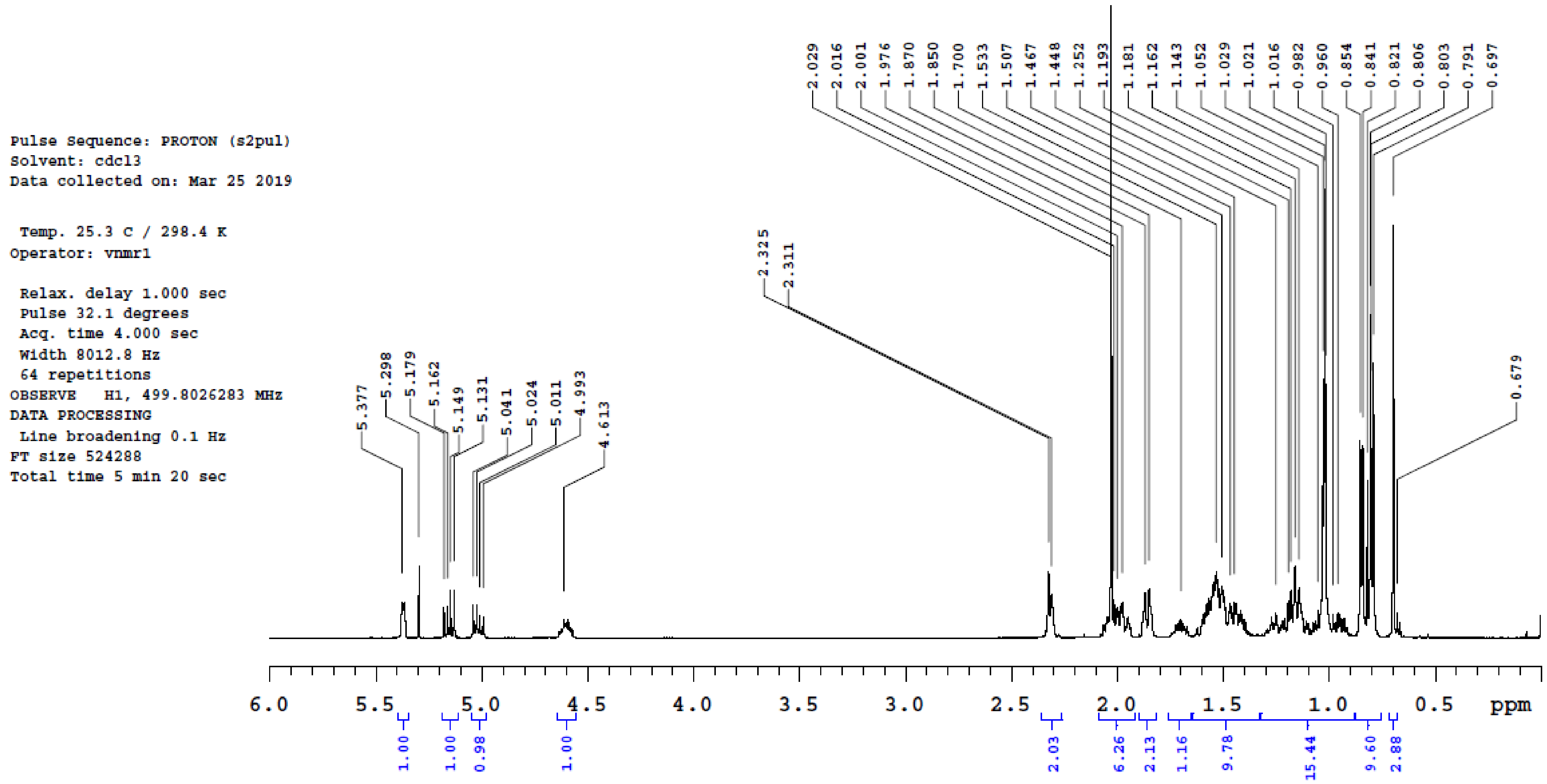

Figure S73. ^{1}H spectrum of stigmast-5-en-3β-yl acetate (Stigmasteryl acetate) (**24**) in $CDCl_3$

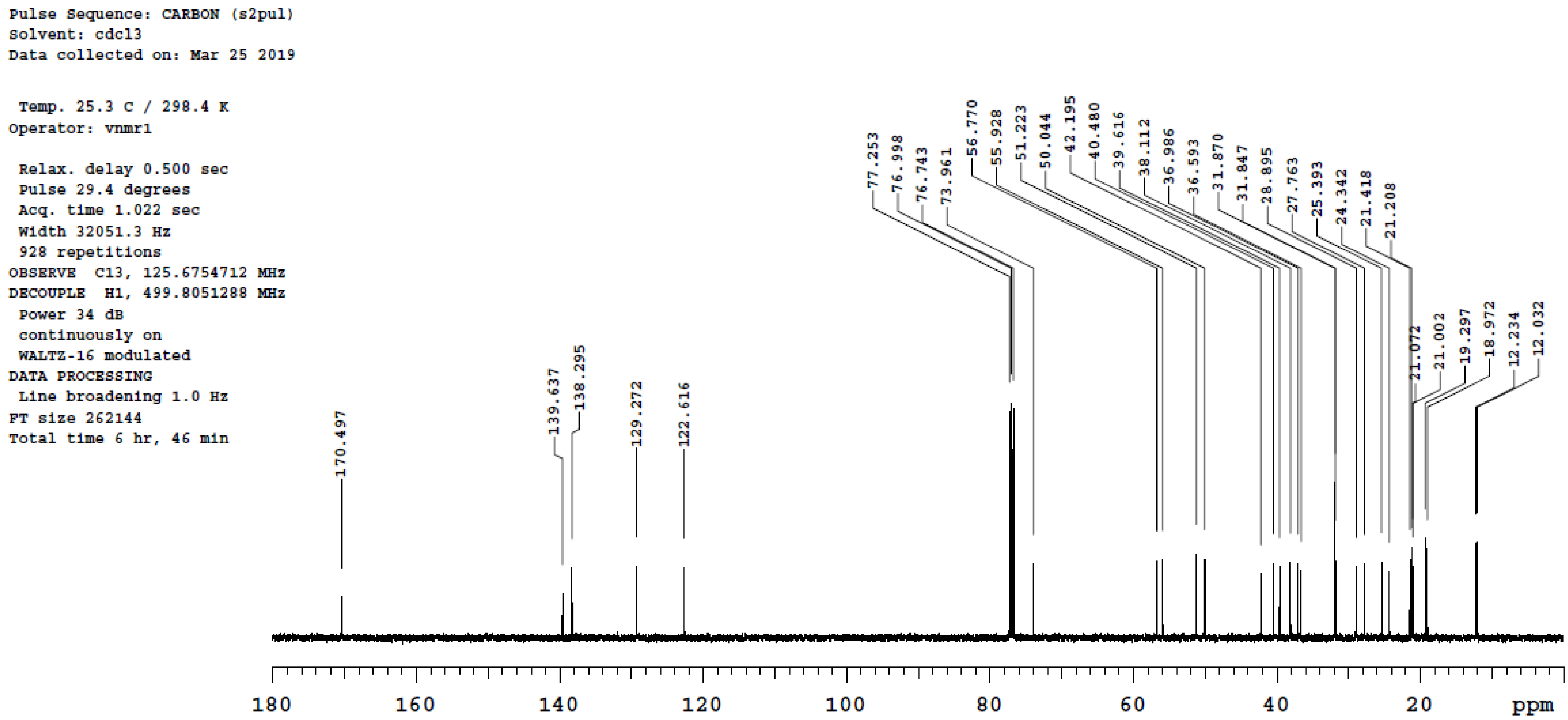

Figure S74. ^{13}C spectrum of stigmast-5-en-3β-yl acetate (Stigmasteryl acetate) (**24**) in $CDCl_3$

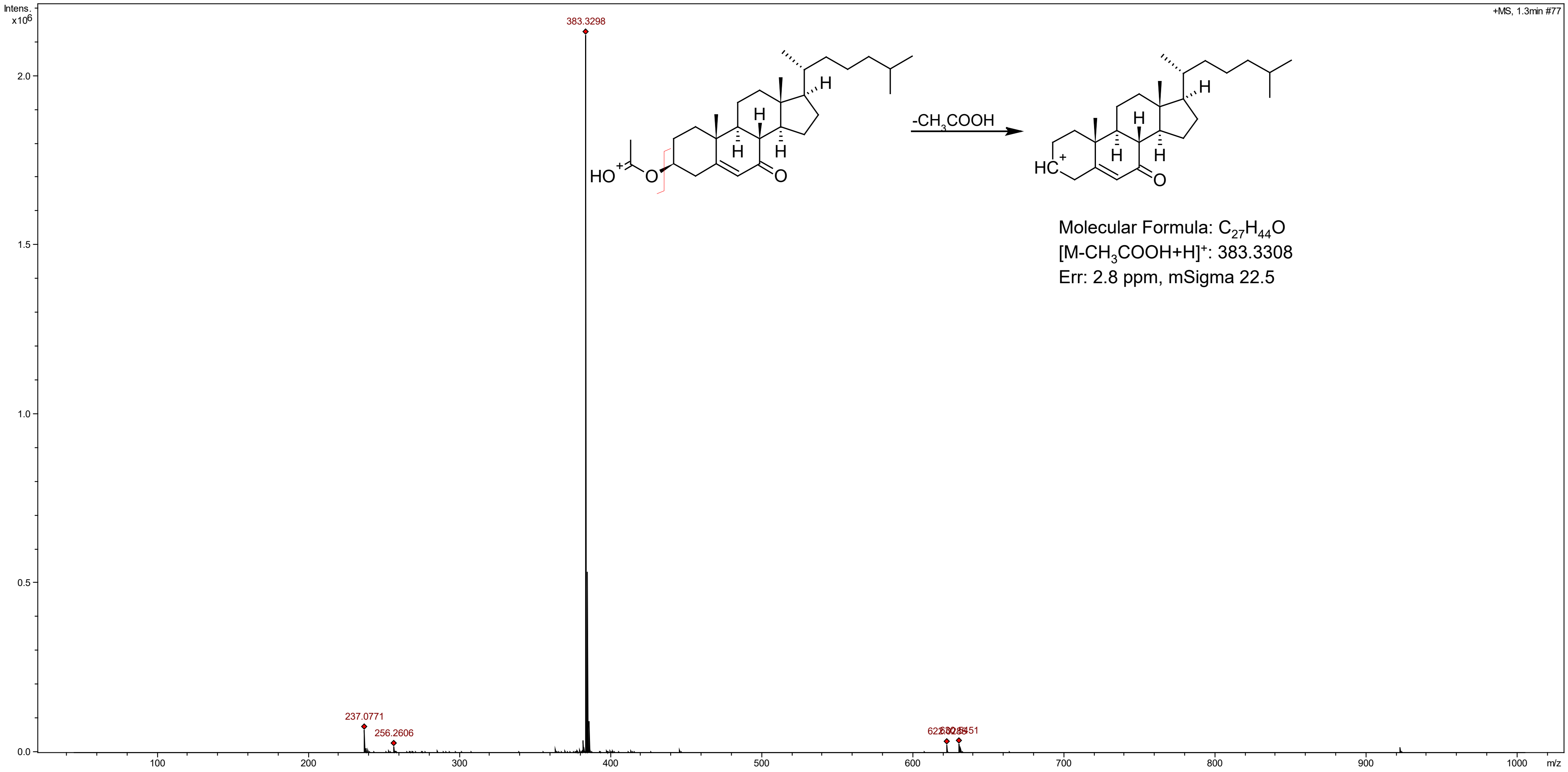

Figure S75. Mass spectrum of cholest-5-en-7-one-3β-yl acetate (7-ketocholesterol acetate) (**25**)

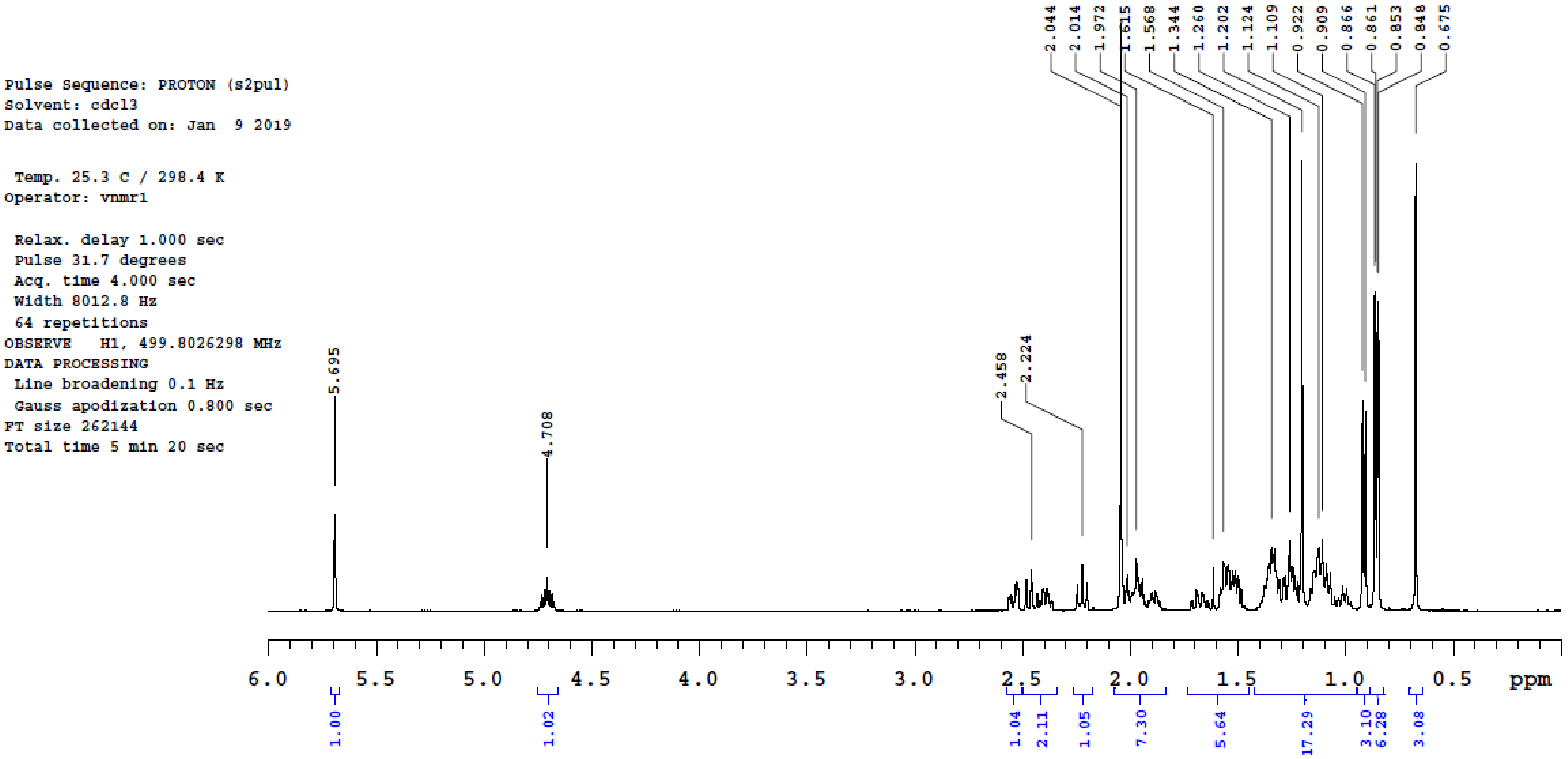

Figure S76. ^{1}H spectrum of cholest-5-en-7-one-3β-yl acetate (7-ketocholesterol acetate) (**25**) in $CDCl_3$

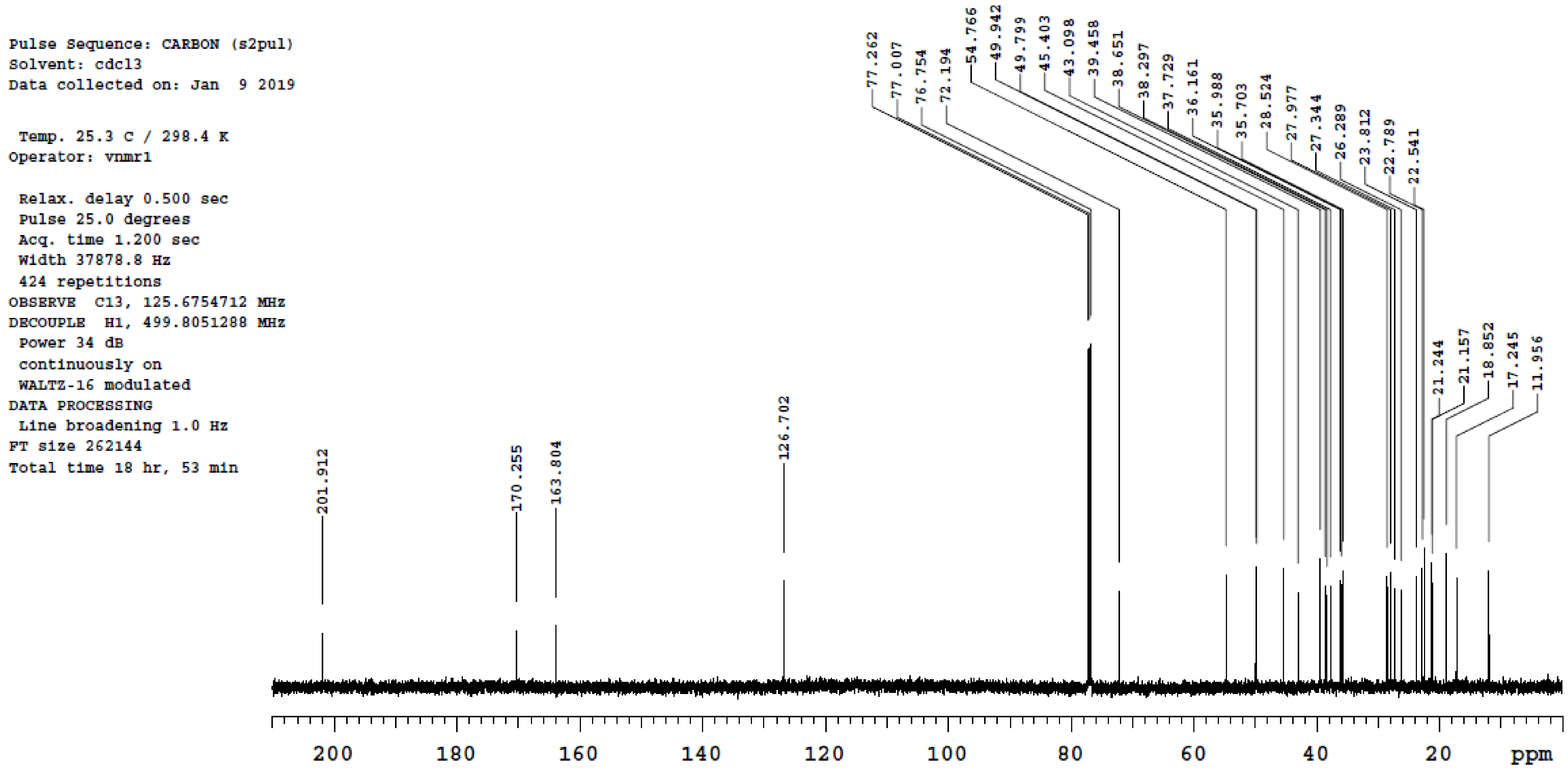

Figure S77. ^{13}C spectrum of cholest-5-en-7-one-3β-yl acetate (7-ketocholesterol acetate) (**25**) in $CDCl_3$

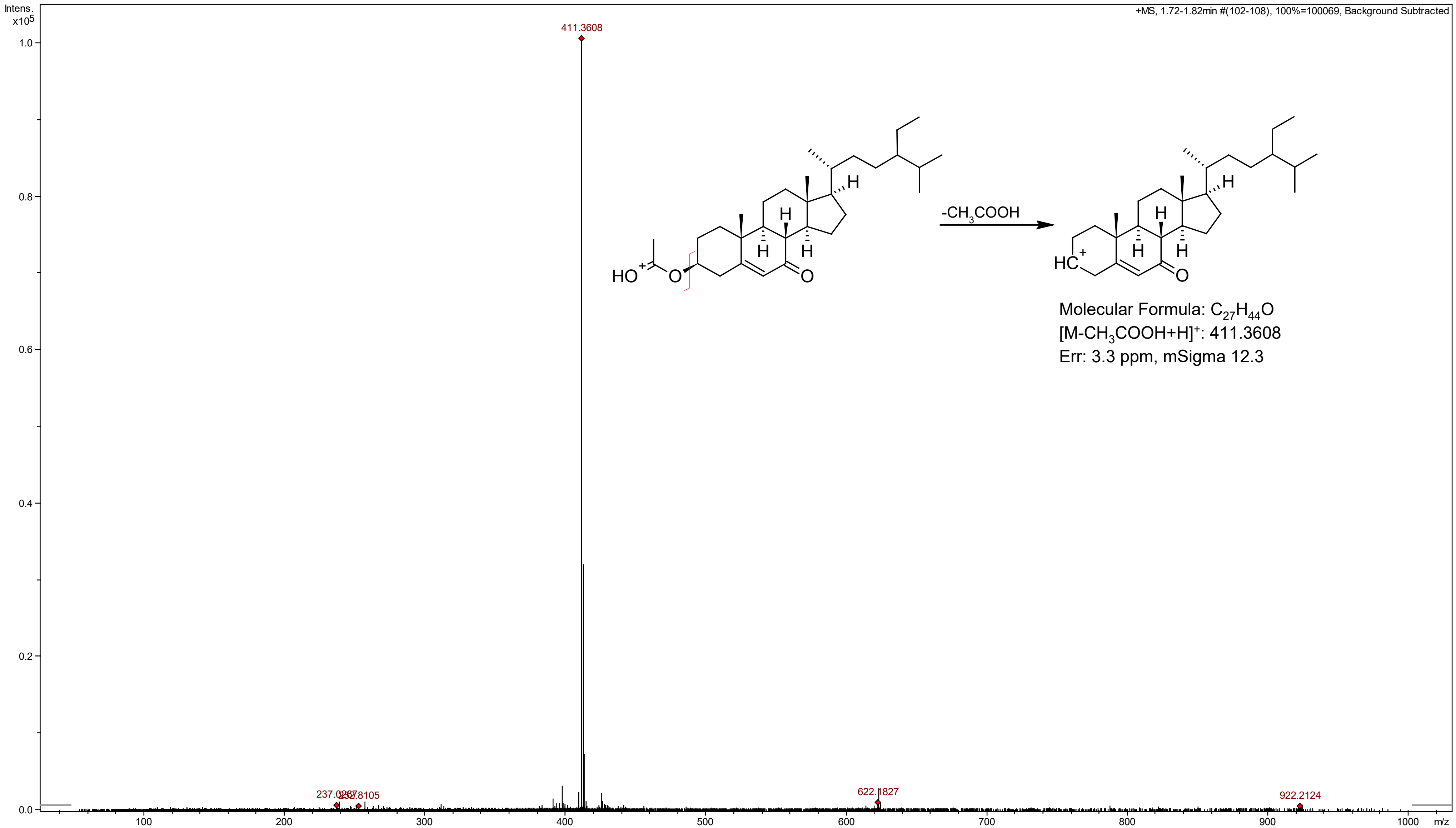

Figure S78. Mass spectrum of sitost-5-en-7-one-3β-yl acetate (7-ketositosteryl acetate) (**26**)

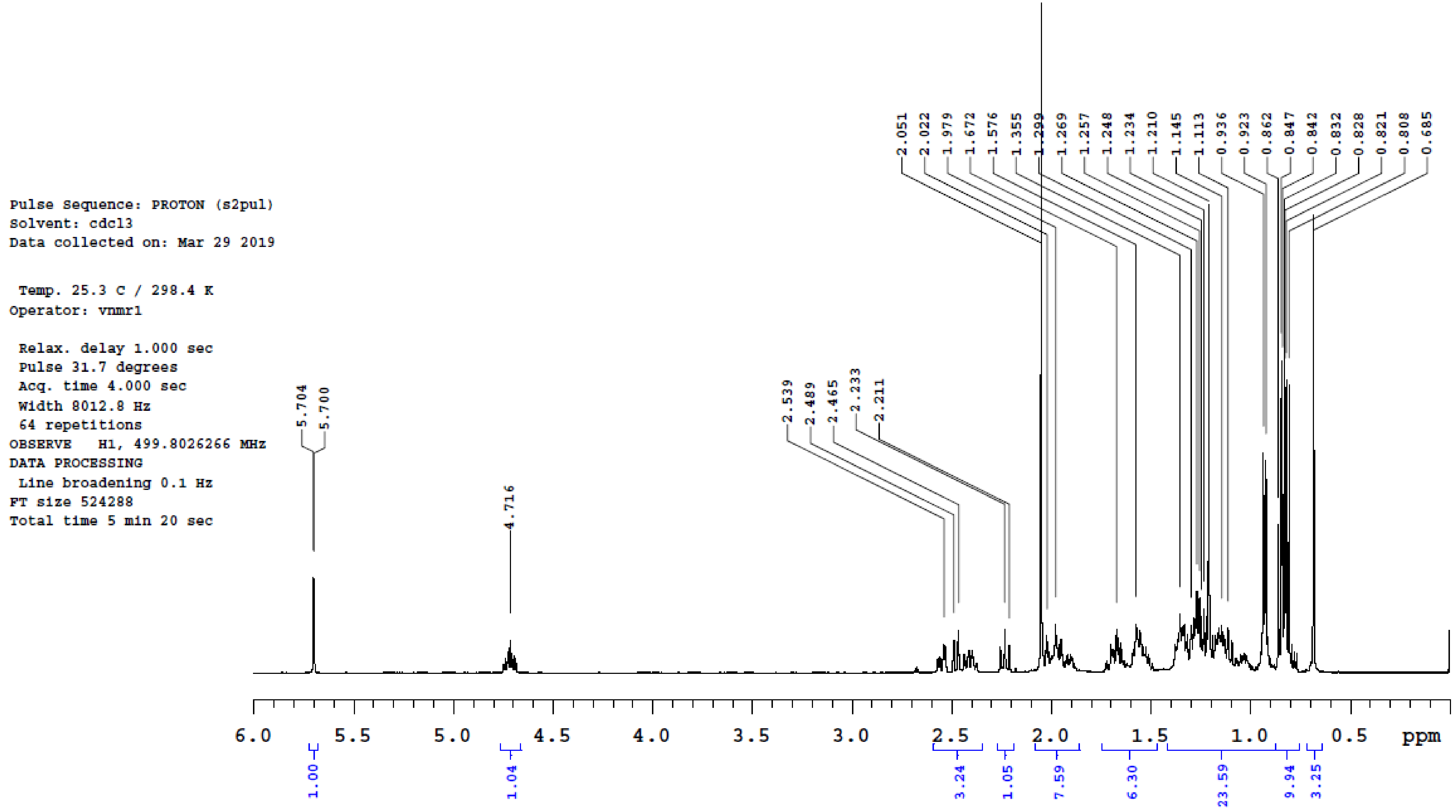

Figure S79. ^{1}H spectrum of sitost-5-en-7-one-3β-yl acetate (7-ketositosteryl acetate) (**26**) in $CDCl_3$

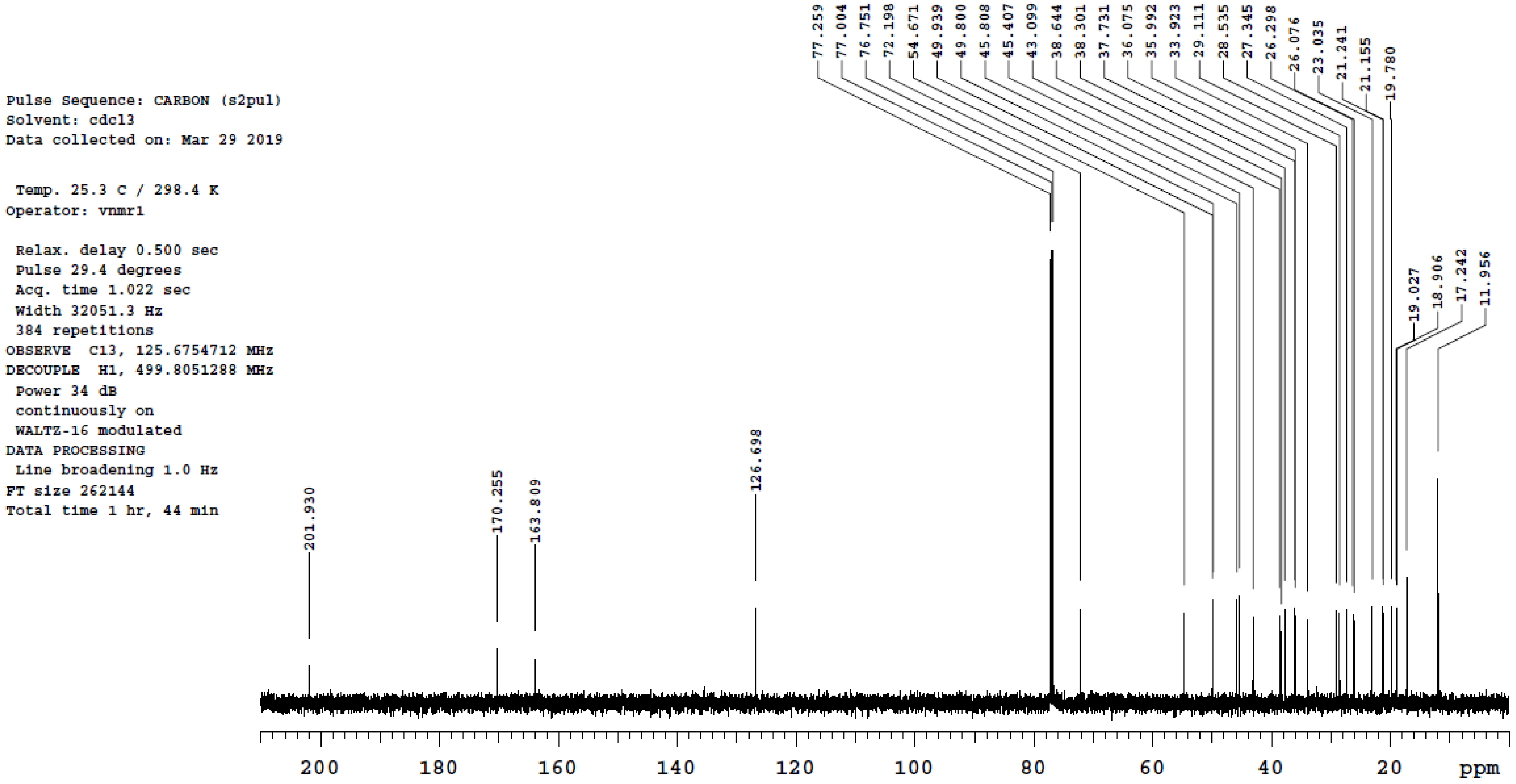

Figure S80. ^{13}C spectrum of sitost-5-en-7-one-3β-yl acetate (7-ketositosteryl acetate) (**26**) in $CDCl_3$

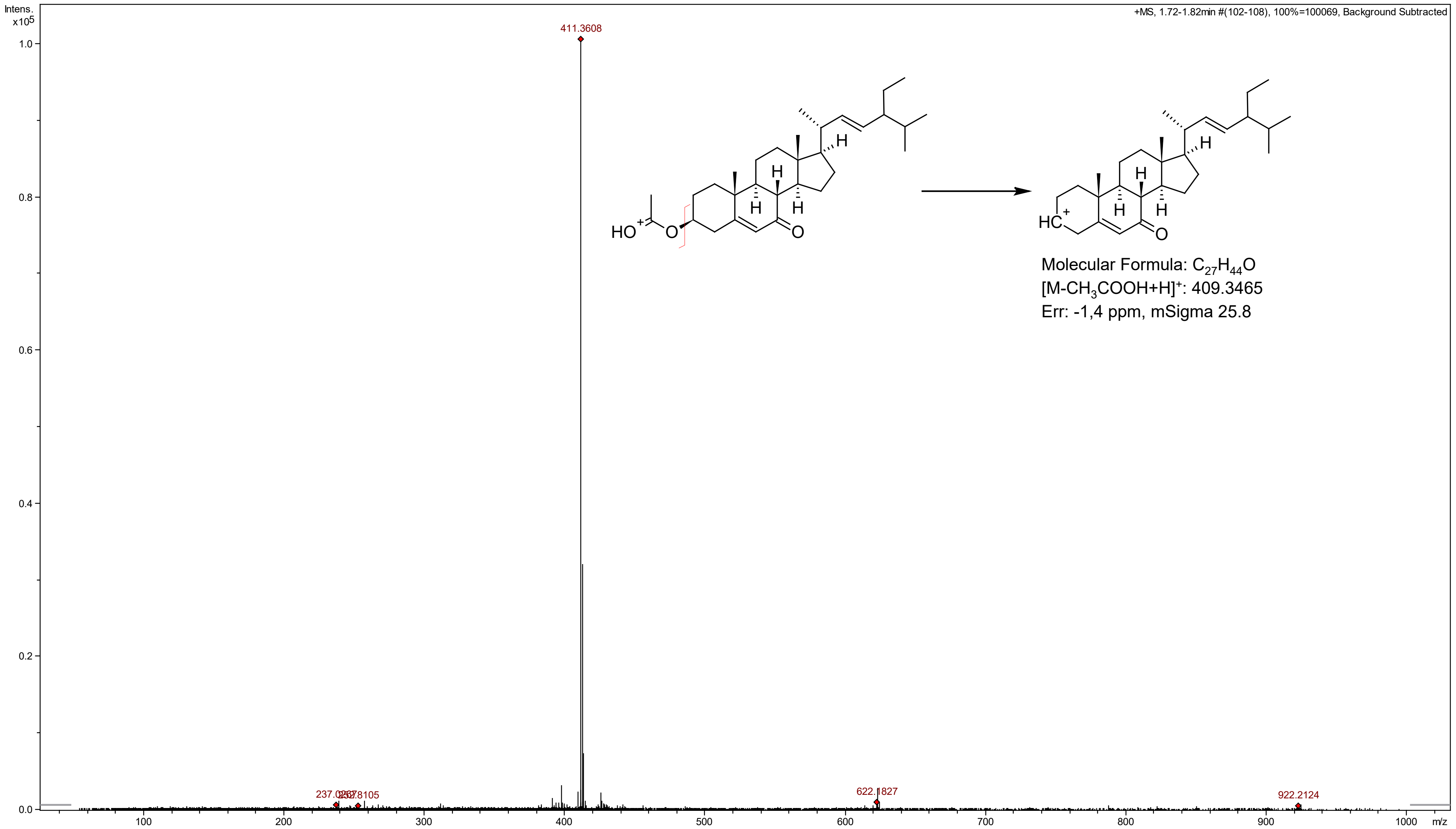

Figure S81. Mass spectrum of stigmast-5-en-7-one-3β-yl acetate (7-ketostigmasteryl acetate) (**27**)

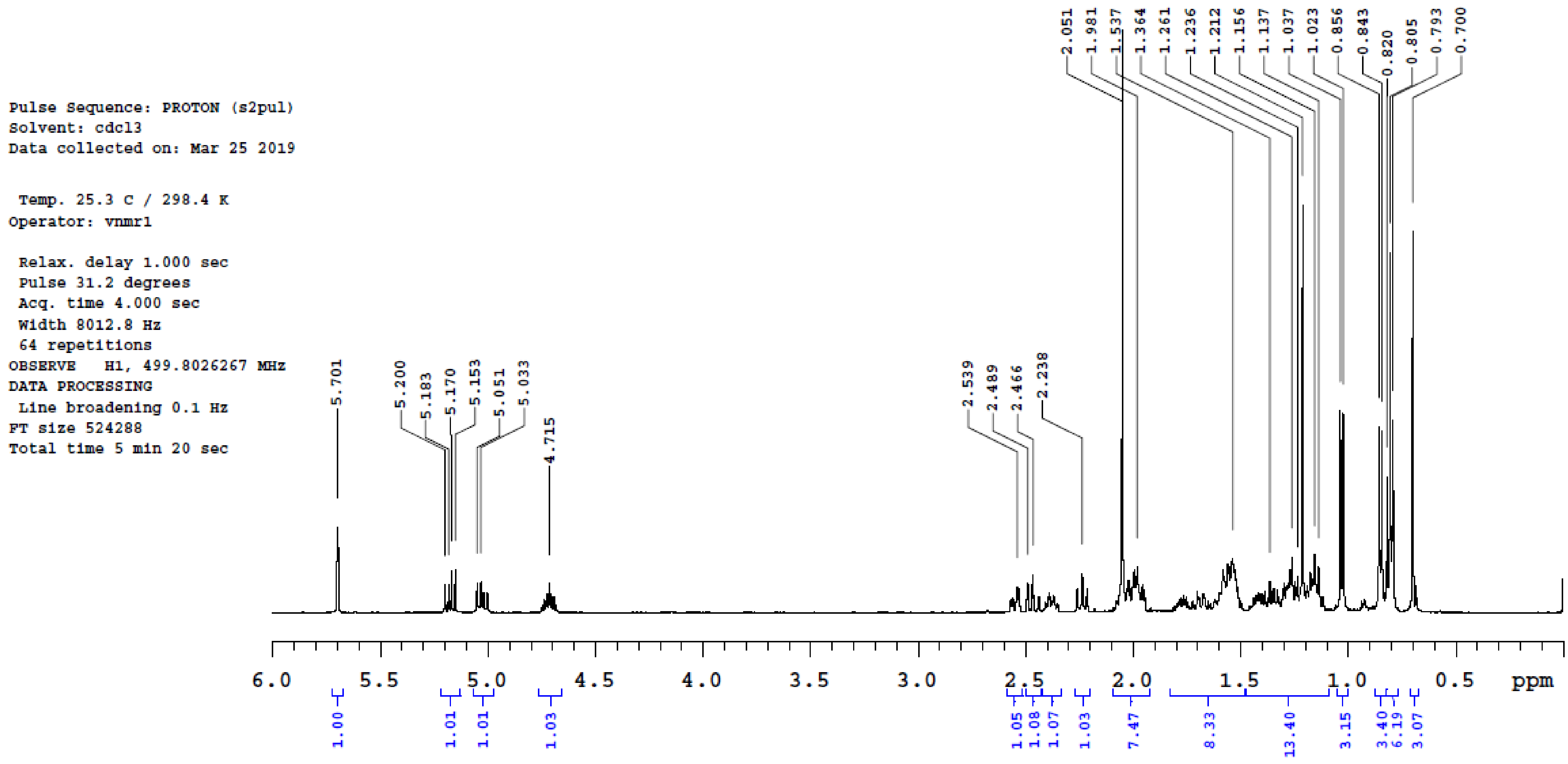

Figure S82. ^{1}H spectrum of stigmast-5-en-7-one-3β-yl acetate (7-ketostigmasteryl acetate) (**27**) in $CDCl_3$

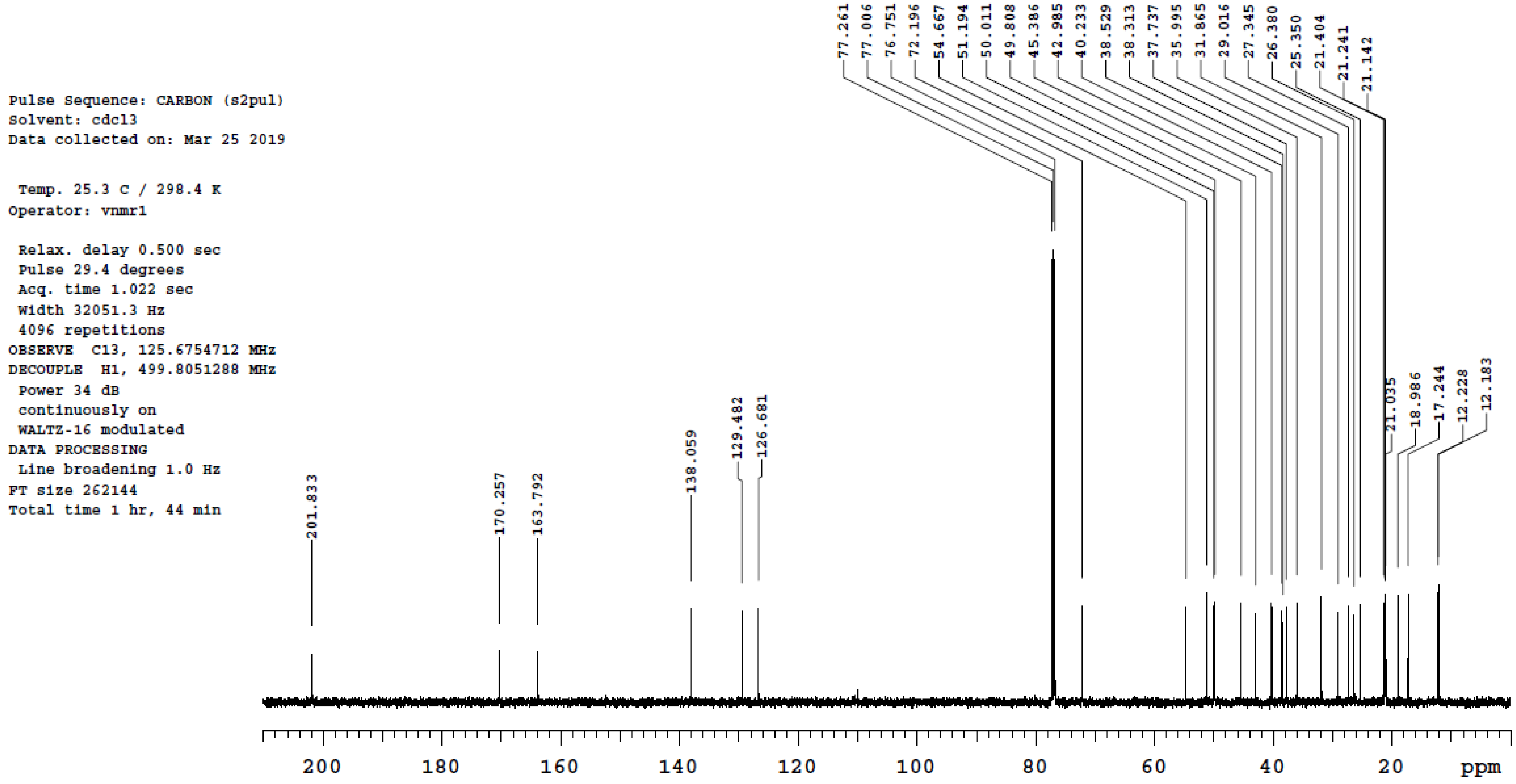

Figure S83. ^{13}C spectrum of stigmast-5-en-7-one-3β-yl acetate (7-ketostigmasteryl acetate) (**27**) in $CDCl_3$

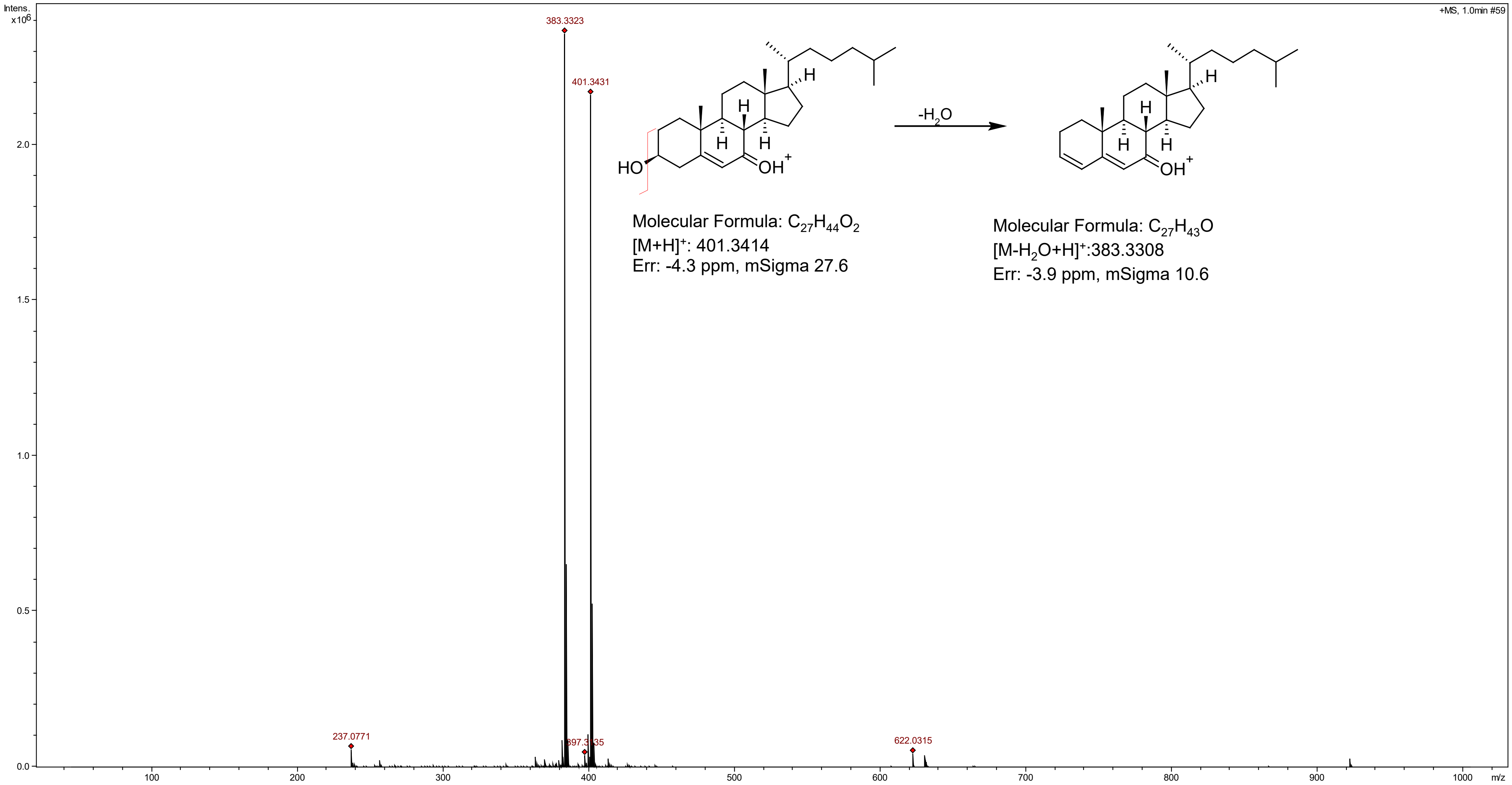

Figure S84. Mass spectrum of 5-cholesten-3β-ol-7-one (7-ketocholesterol) (**28**)

Pulse Sequence: PROTON (s2pul)
Solvent: cdcl3
Data collected on: Jan 10 2019

Temp. 25.0 C / 298.1 K
Operator: vnmr1

Relax. delay 0.001 sec
Pulse 33.8 degrees
Acq. time 4.000 sec
Width 5000.0 Hz
Single scan
OBSERVE H1, 599.8350651 MHz
DATA PROCESSING
Line broadening 0.1 Hz
FT size 131072
Total time 0 min 4 sec

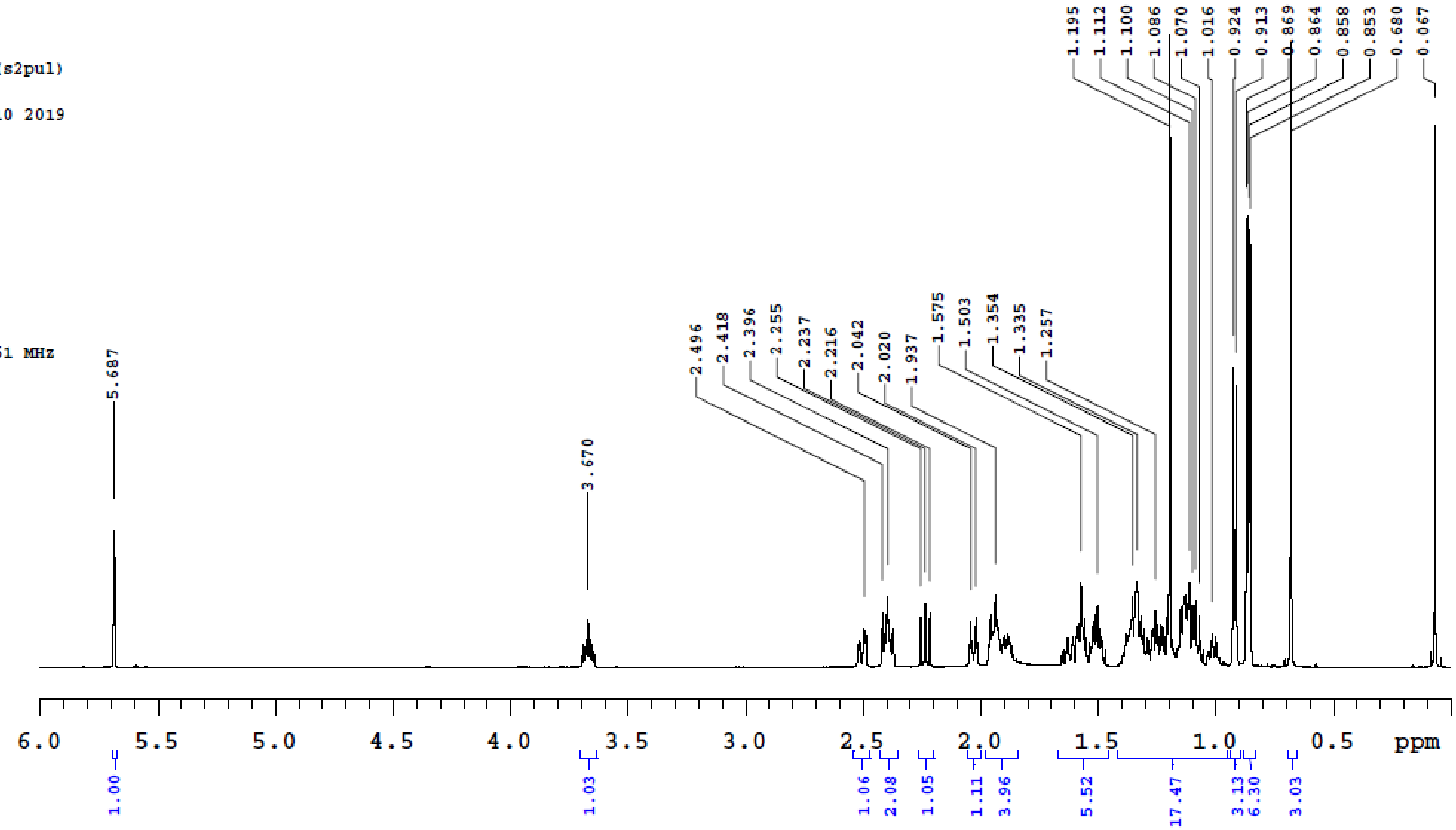

Figure S85. ^{1}H spectrum of 5-cholesten-3β-ol-7-one (7-ketocholesterol) (**28**) in $CDCl_3$

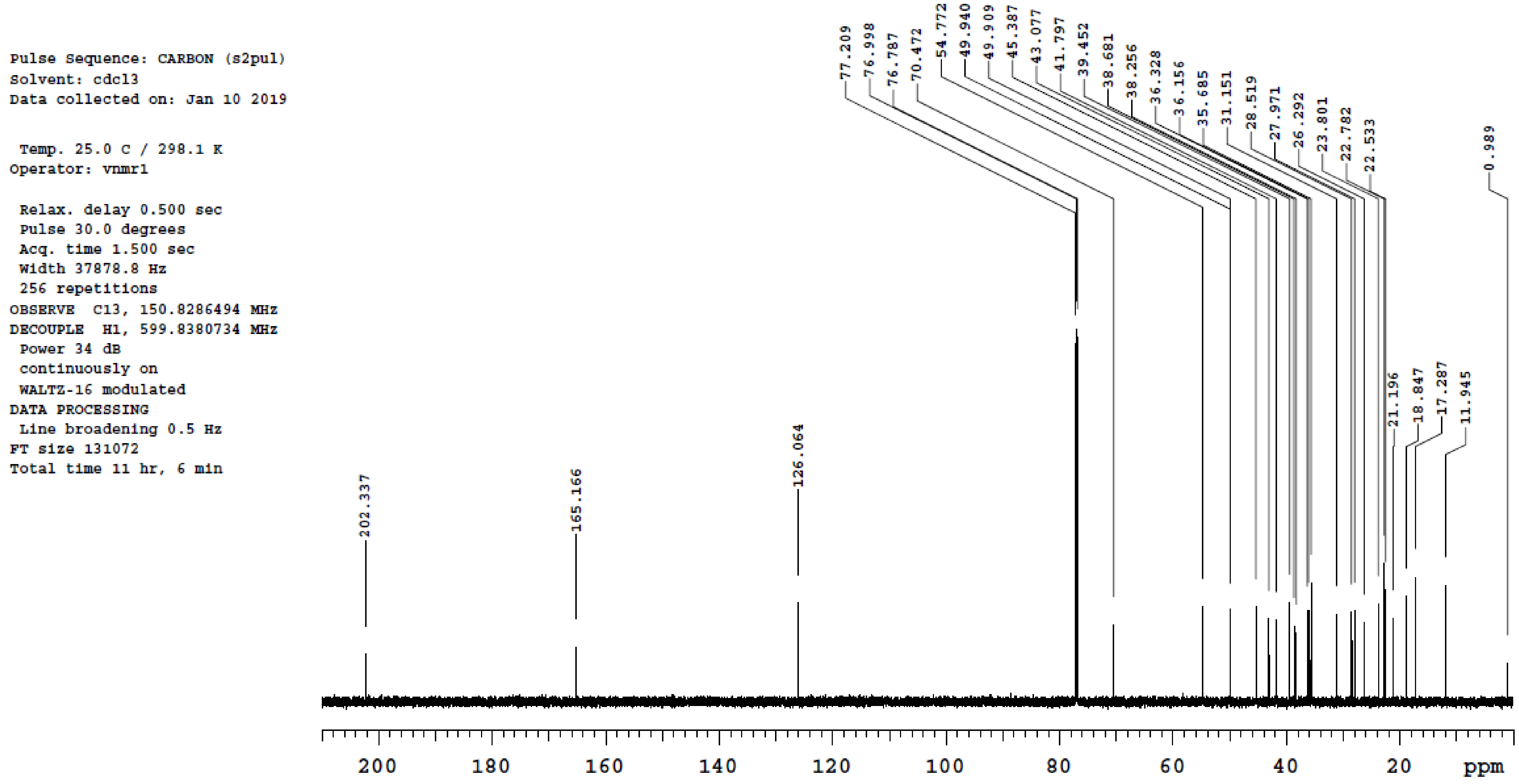

Figure S86. ^{13}C spectrum of 5-cholesten-3β-ol-7-one (7-ketocholesterol) (**28**) in $CDCl_3$

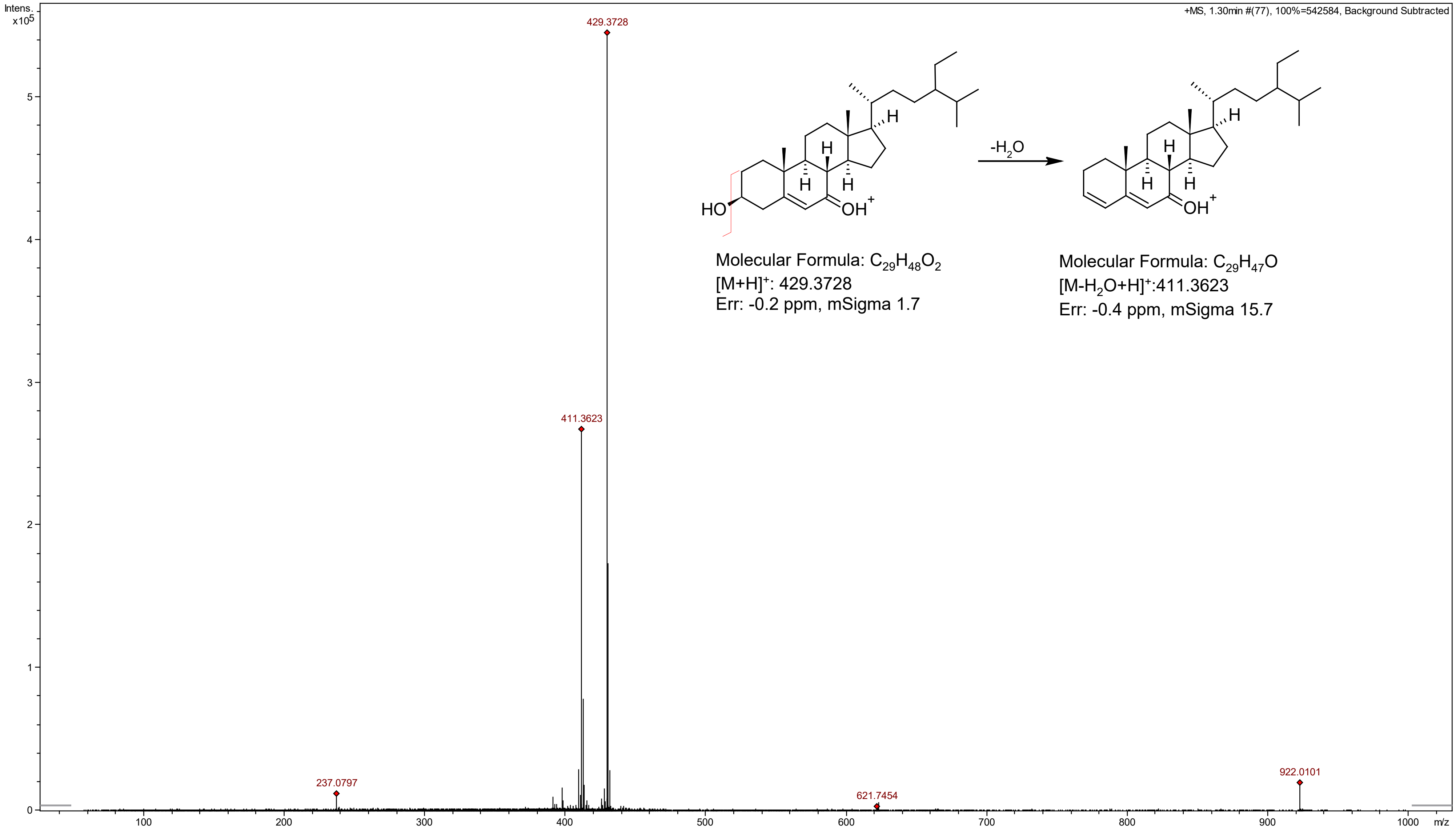

Figure S87. Mass spectrum of 5-sitosten-3β-ol-7-one (7-ketositosterol) (**29**)

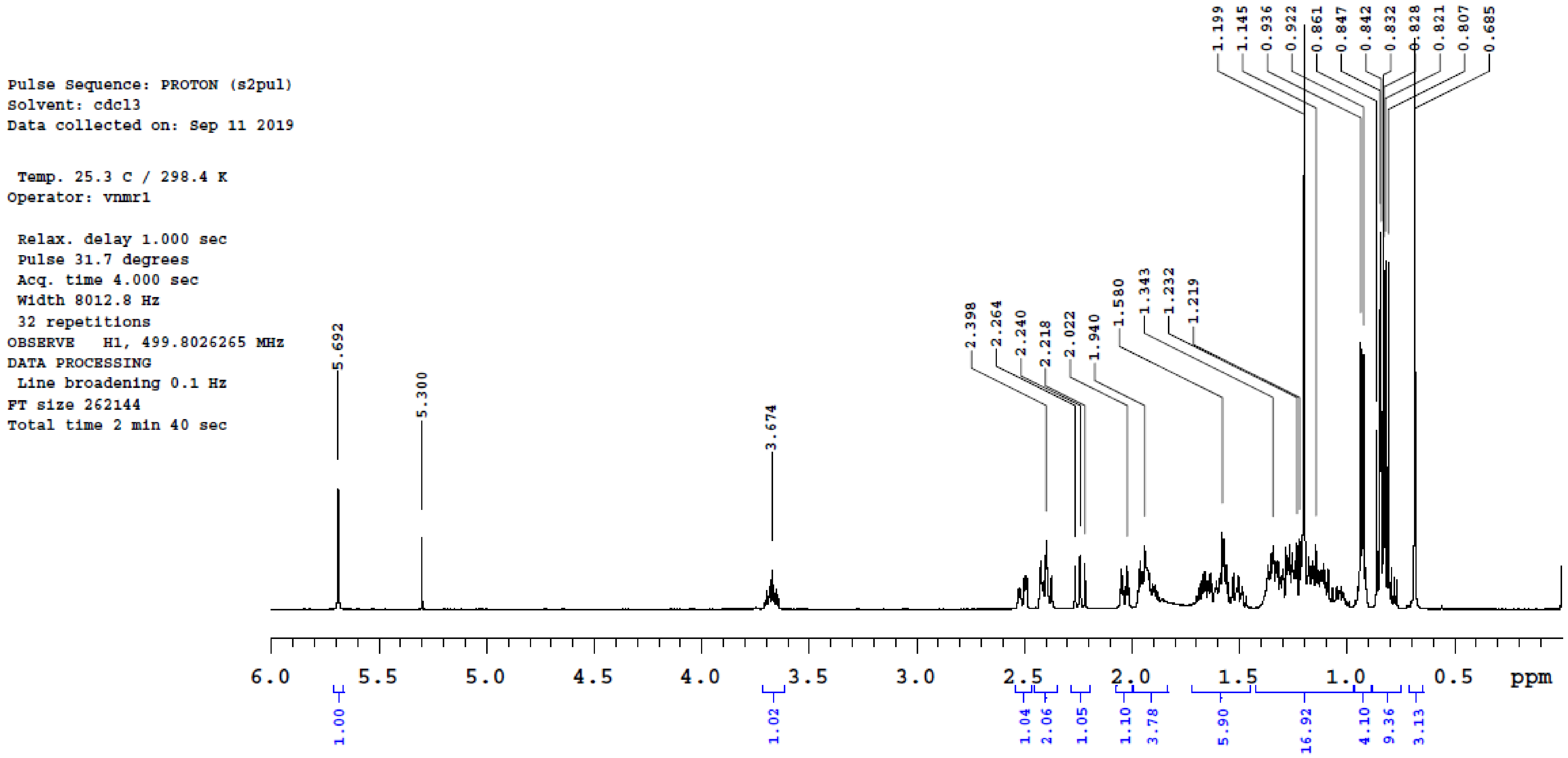

Figure S88. ^{1}H spectrum of 5-sitosten-3β-ol-7-one (7-ketositosterol) (**29**) in $CDCl_3$

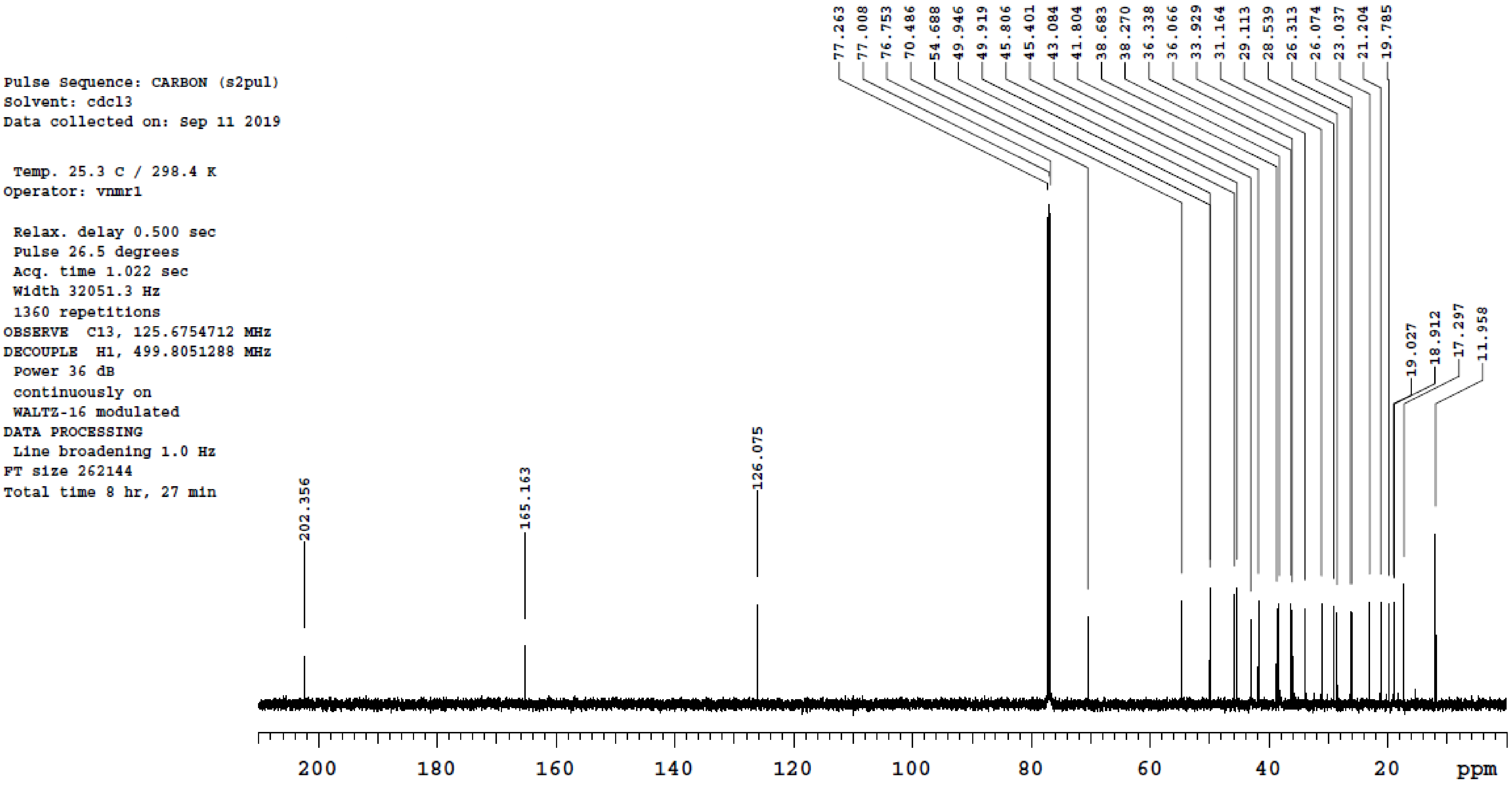

Figure S89. ^{13}C spectrum of 5-sitosten-3β-ol-7-one (7-ketositosterol) (**29**) in $CDCl_3$

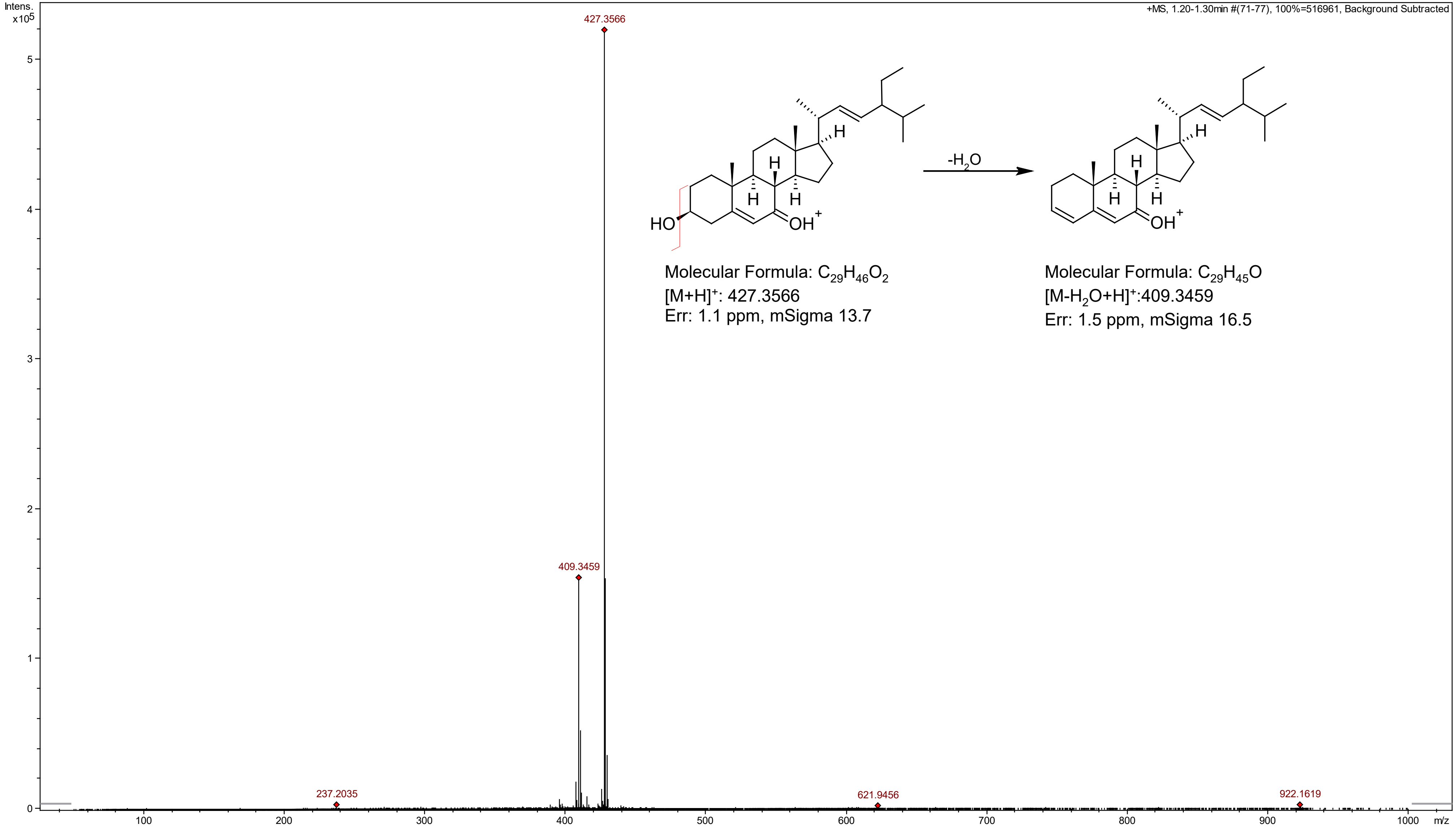

Figure S90. Mass spectrum of 5-stigmasten-3β-ol-7-one (7-ketostigmasterol) (**30**)

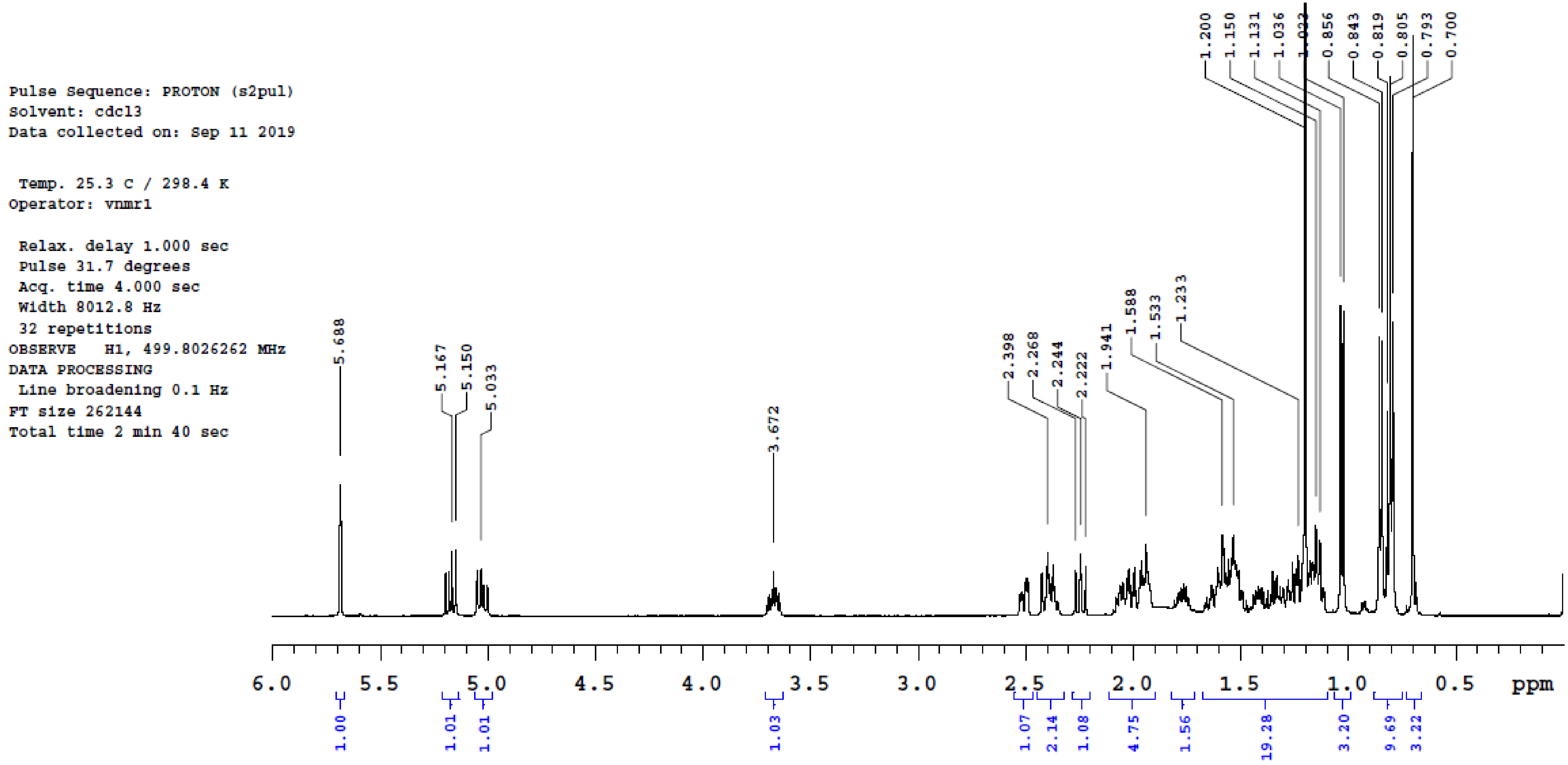

Figure S91. ^{1}H spectrum of 5-stigmasten-3β-ol-7-one (7-ketostigmasterol) (**30**) in $CDCl_3$

Pulse Sequence: CARBON (s2pul)
Solvent: cdcl3
Data collected on: Sep 11 2019

Temp. 25.3 C / 298.4 K
Operator: vnmr1

Relax. delay 0.500 sec
Pulse 26.5 degrees
Acq. time 1.022 sec
Width 32051.3 Hz
960 repetitions
OBSERVE C13, 125.6754712 MHz
DECOUPLE H1, 499.8051288 MHz
Power 36 dB
continuously on
WALTZ-16 modulated
DATA PROCESSING
Line broadening 1.0 Hz
FT size 262144
Total time 8 hr, 27 min

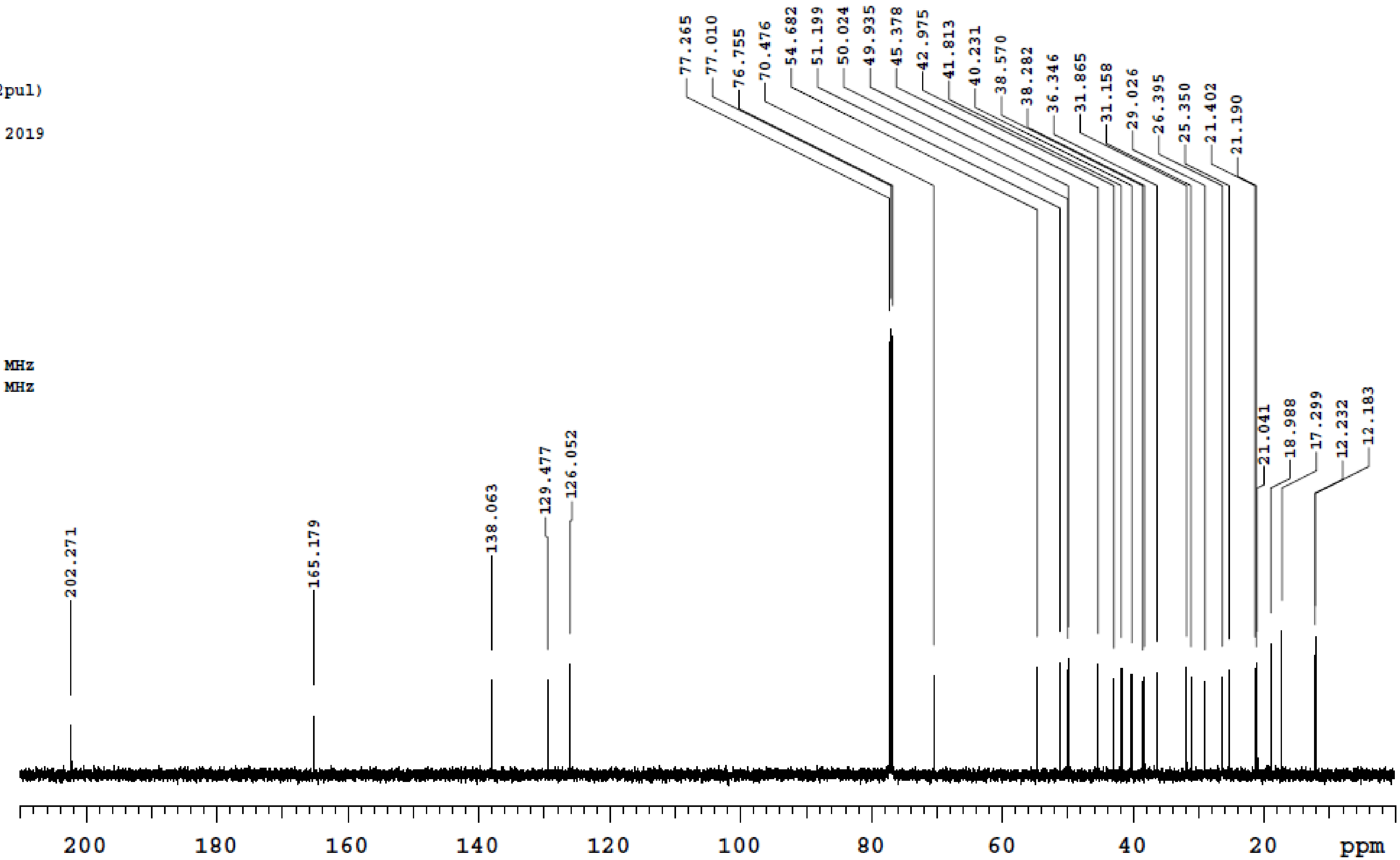

Figure S92. ^{13}C spectrum of 5-stigmasten-3β-ol-7-one (24) (7-ketostigmasterol) (**30**) in $CDCl_3$

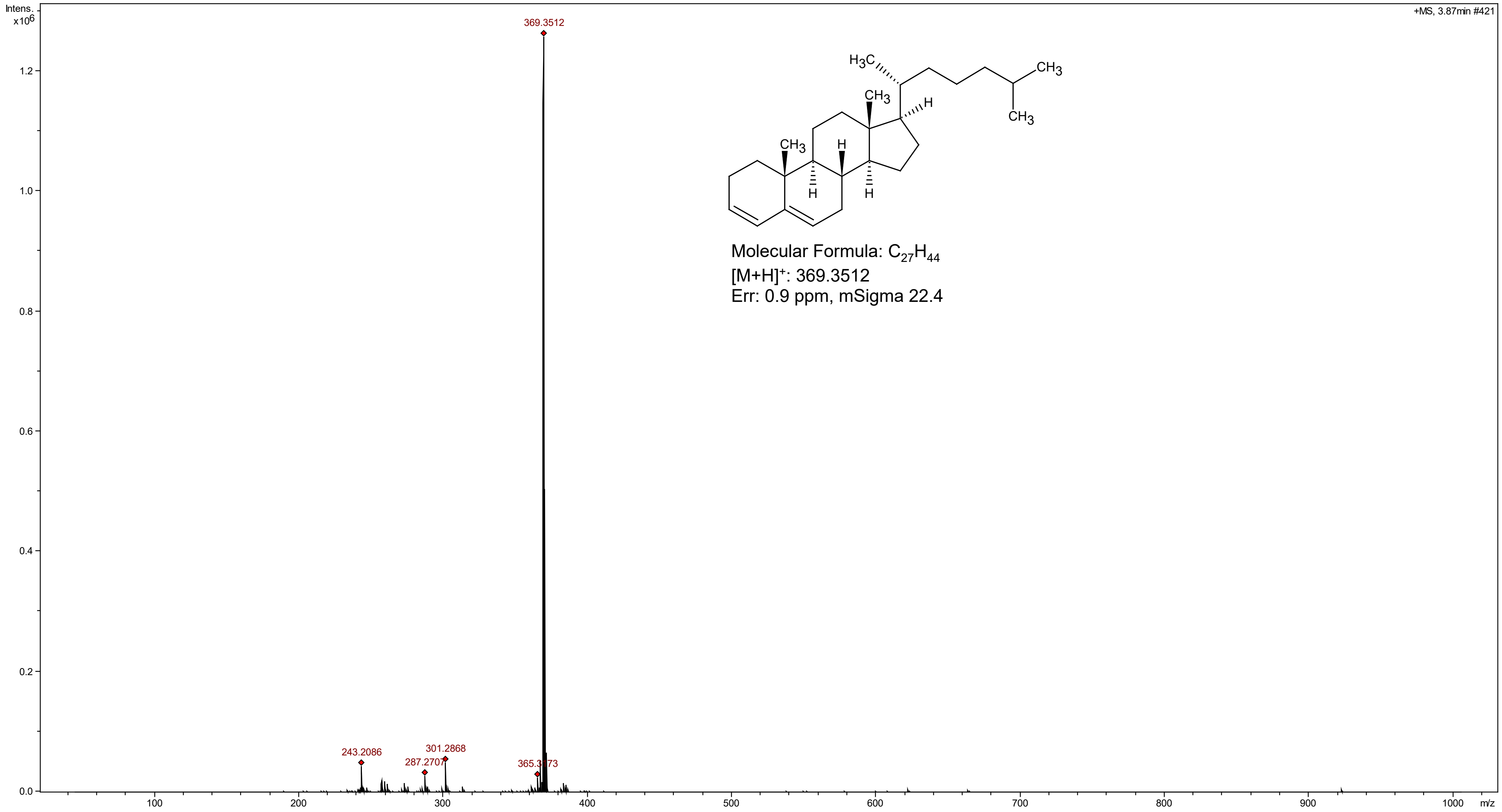

Figure S93. Mass spectrum of cholesta-3,5-diene (cholestadiene) (**31**)

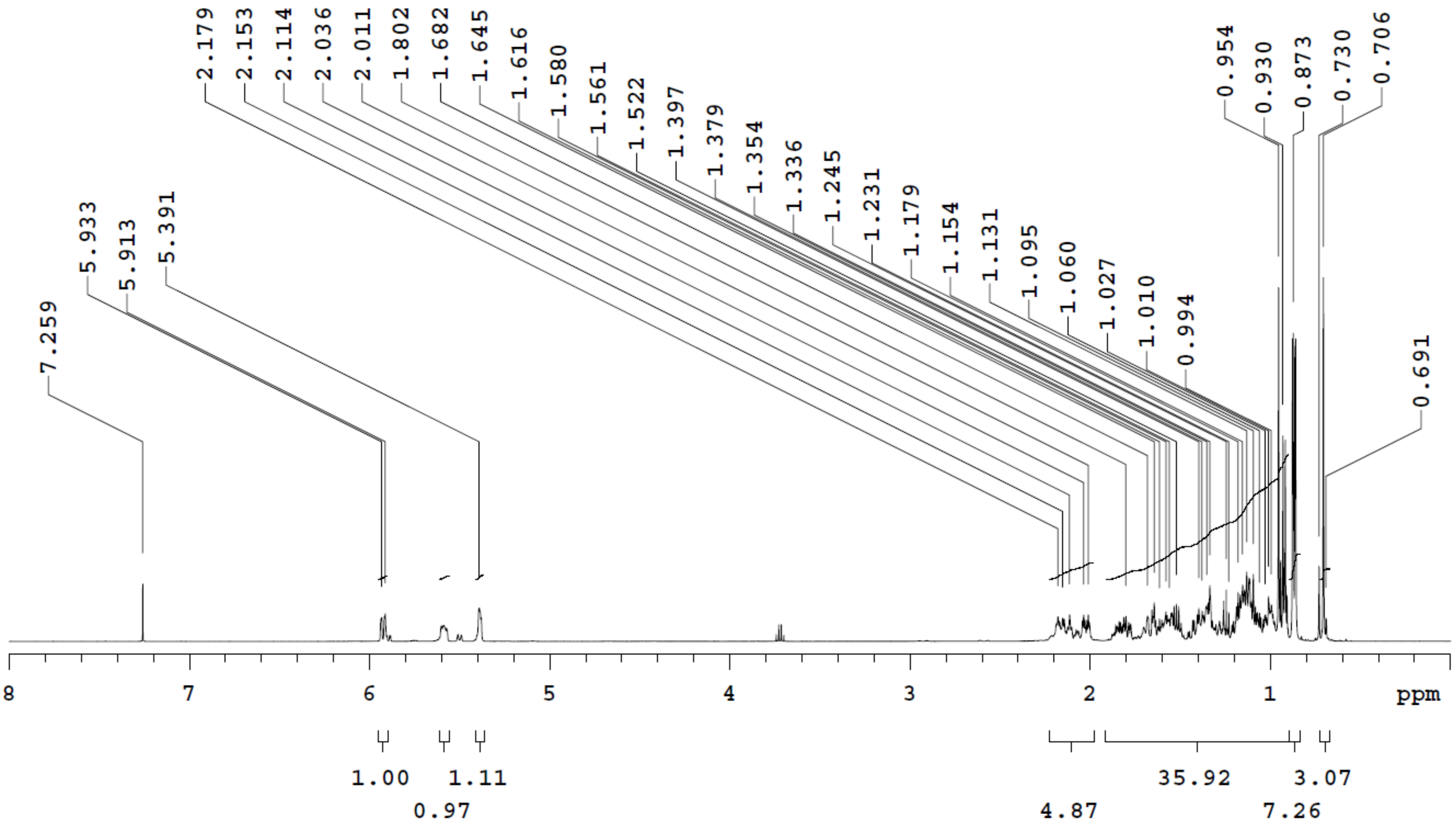

Figure S94. ^{1}H spectrum of cholesta-3,5-diene (cholestadiene) (**31**) in $CDCl_3$

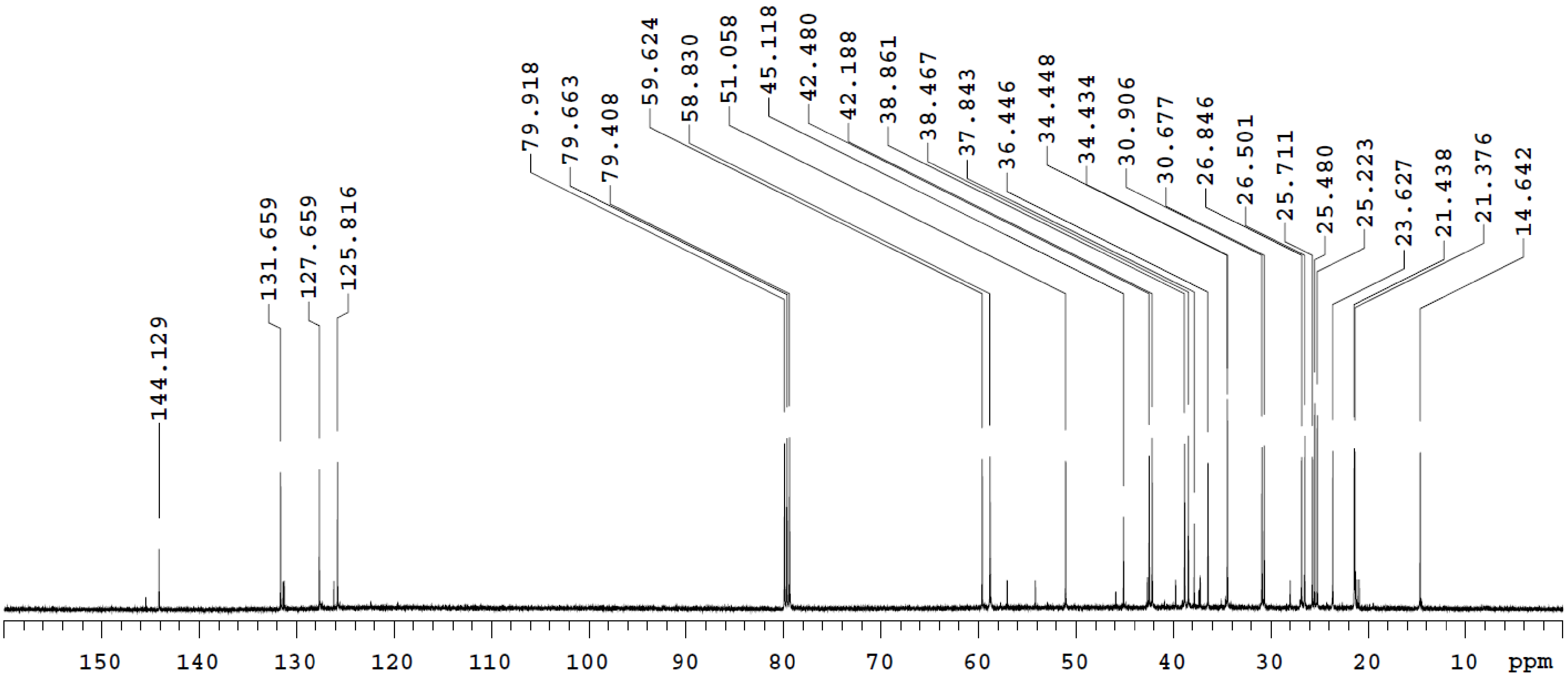

Figure S95. ^{1}H spectrum of cholesta-3,5-diene (cholestadiene) (**31**) in $CDCl_3$
